# Antisense, but not sense, repeat expanded RNAs activate PKR/eIF2α-dependent ISR in *C9ORF72* FTD/ALS

**DOI:** 10.7554/eLife.85902

**Published:** 2023-04-19

**Authors:** Janani Parameswaran, Nancy Zhang, Elke Braems, Kedamawit Tilahun, Devesh C Pant, Keena Yin, Seneshaw Asress, Kara Heeren, Anwesha Banerjee, Emma Davis, Samantha L Schwartz, Graeme L Conn, Gary J Bassell, Ludo Van Den Bosch, Jie Jiang

**Affiliations:** 1 Department of Cell Biology, Emory University Atlanta United States; 2 Department of Neurosciences, Experimental Neurology and Leuven Brain Institute, KU Leuven Leuven Belgium; 3 Center for Brain & Disease Research, Laboratory of Neurobiology, VIB, Campus Gasthuisberg Leuven Belgium; 4 Department of Neurology, Emory University Atlanta United States; 5 Department of Biochemistry, Emory University Atlanta United States; Baylor College of Medicine United States; Stanford University United States

**Keywords:** C9ORF72, PKR, antisense RNA, FTD/ALS, integrated stress response, Zebrafish

## Abstract

GGGGCC (G_4_C_2_) hexanucleotide repeat expansion in the *C9ORF72* gene is the most common genetic cause of frontotemporal dementia (FTD) and amyotrophic lateral sclerosis (ALS). The repeat is bidirectionally transcribed and confers gain of toxicity. However, the underlying toxic species is debated, and it is not clear whether antisense CCCCGG (C_4_G_2_) repeat expanded RNAs contribute to disease pathogenesis. Our study shows that *C9ORF72* antisense C_4_G_2_ repeat expanded RNAs trigger the activation of the PKR/eIF2α-dependent integrated stress response independent of dipeptide repeat proteins that are produced through repeat-associated non-AUG-initiated translation, leading to global translation inhibition and stress granule formation. Reducing PKR levels with either siRNA or morpholinos mitigates integrated stress response and toxicity caused by the antisense C_4_G_2_ RNAs in cell lines, primary neurons, and zebrafish. Increased phosphorylation of PKR/eIF2α is also observed in the frontal cortex of *C9ORF72* FTD/ALS patients. Finally, only antisense C_4_G_2_, but not sense G_4_C_2_, repeat expanded RNAs robustly activate the PKR/eIF2α pathway and induce aberrant stress granule formation. These results provide a mechanism by which antisense C_4_G_2_ repeat expanded RNAs elicit neuronal toxicity in FTD/ALS caused by *C9ORF72* repeat expansions.

## Introduction

In 2011, GGGGCC (G_4_C_2_) hexanucleotide repeat expansion in the first intron of the chromosome 9 open-reading frame 72 (*C9ORF72*) gene was identified as the most common genetic cause of frontotemporal dementia (FTD) and amyotrophic lateral sclerosis (ALS), two neurodegenerative diseases that are now believed to belong to a continuous disease spectrum with clinical, pathological, and genetic overlaps (DeJesus-Hernandez et al., 2011; Renton et al., 2011). In normal populations, the G_4_C_2_ repeat size is between 2 and 30, whereas it expands to hundreds or thousands in FTD/ALS patients (referred to hereafter as C9FTD/ALS). C9FTD/ALS thus joins an increasing number of repeat expansion disorders, including Huntington’s disease, myotonic dystrophy, and several spinocerebellar ataxias (La Spada and Taylor, 2010). Based on the initial pathological assessment of C9FTD/ALS patient postmortem tissues and lessons learned from other repeat expansion disorders, several pathogenic mechanisms by which the expanded *C9ORF72* repeats can exert toxicity were proposed (Jiang and Ravits, 2019). First, expanded G_4_C_2_ repeats inhibit *C9ORF72* mRNA transcription, leading to haploinsufficiency of the C9ORF72 protein (Braems et al., 2020; Gijselinck et al., 2012; van Blitterswijk et al., 2015); second, *C9ORF72* repeats are bidirectionally transcribed into sense G_4_C_2_ and antisense CCCCGG (C_4_G_2_) RNAs. These repeat expanded RNAs may cause gain of toxicity by sequestering essential RNA-binding proteins (RBPs) into RNA foci and/or by the production of toxic dipeptide repeat (DPR) proteins via non-canonical repeat-associated non-AUG-dependent (RAN) translation from all reading frames. More specifically, translating from sense G_4_C_2_ RNAs produces GA (glycine-alanine), GP (glycine-proline), and GR (glycine-arginine) DPR proteins, and translating from antisense C_4_G_2_ RNAs produces GP (glycine-proline), PA (proline-alanine), and PR (proline-arginine) DPR proteins (Ash et al., 2013). In addition to these pure dimeric DPR proteins, there is also evidence of chimeric DPR proteins in both model systems and patients (Gao et al., 2017; McEachin et al., 2020a; Tabet et al., 2018).

How *C9ORF72* repeat expansions cause FTD/ALS has been extensively explored. Although reducing C9ORF72 in zebrafish or *Caenorhabditis elegans* can cause motor deficits (Swinnen et al., 2018; Therrien et al., 2013), reduced or even complete deletion of C9ORF72 in mice does not lead to FTD/ALS-like abnormalities, suggesting that loss of C9ORF72 is not the main disease driver (Atanasio et al., 2016; Burberry et al., 2016; Koppers et al., 2015; O’Rourke et al., 2016; Sudria-Lopez et al., 2016; Sullivan et al., 2016; Ugolino et al., 2016). Supporting this, no missense or truncation mutations in *C9ORF72* are yet found in FTD/ALS patients (Harms et al., 2013). On the other hand, several lines of studies, by expressing either G_4_C_2_ repeats (Chew et al., 2015; Jiang et al., 2016) or individual codon-optimized, ATG-driven DPR proteins (Choi et al., 2019; Mizielinska et al., 2014; Zhang et al., 2019), support that gain of toxicity from the repeat expanded RNAs plays a central role in disease pathogenesis. Finally, loss of C9ORF72, which plays a role in autophagy/lysosomal functions, can exacerbate toxicity from the repeat expanded RNAs (Boivin et al., 2020; Zhu et al., 2020).

The underlying toxic species arising from *C9ORF72* repeat expanded RNAs that drive disease is still debated. Several RBPs have been suggested to interact with G_4_C_2_ or C_4_G_2_ repeat RNAs and to co-localize with RNA foci (Celona et al., 2017; Conlon et al., 2016; Donnelly et al., 2013; Haeusler et al., 2014; Lee et al., 2013; Mori et al., 2013; Sareen et al., 2013; Xu et al., 2013). However, strong evidence supporting that loss of any proposed RBPs drives C9FTD/ALS is lacking. In contrast, ectopic expression of individual DPR proteins, especially GR and PR, causes toxicity in various model systems (Boeynaems et al., 2016; Choi et al., 2019; Freibaum et al., 2015; Hao et al., 2019; Kanekura et al., 2016; Lee et al., 2016; May et al., 2014; Mizielinska et al., 2014; Schludi et al., 2017; Tao et al., 2015; Wen et al., 2014; Yamakawa et al., 2015; Yang et al., 2015; Zhang et al., 2018; Zhang et al., 2016; Zhang et al., 2019; Zhang et al., 2014; Zu et al., 2011). To determine the relative contributions of RNA foci- and DPR protein-mediated toxicity, two studies employed interrupted repeats with stop codons in all reading frames to prevent DPR protein production and concluded that neither sense or antisense repeat expanded RNAs are toxic in *Drosophila* (Moens et al., 2018; Tran et al., 2015). This was challenged by another study showing both sense and antisense repeat expanded RNAs can cause motor axonopathy in zebrafish independent of DPR proteins (Swinnen et al., 2018). Irrespective of RNA foci and DPR proteins, studies using antisense oligonucleotide (ASOs) to selectively degrade sense G_4_C_2_ repeat expanded RNAs strongly support its role in C9FTD/ALS pathogenesis. These sense strand-specific ASOs not only mitigate toxicity from *C9ORF72* repeat expansions in both transgenic mice expressing G_4_C_2_ repeats (Jiang et al., 2016) and patient IPSC-derived neurons (Donnelly et al., 2013), but also reverse downstream cellular and molecular alterations such as nucleocytoplasmic transport deficits (Zhang et al., 2015). However, whether antisense C_4_G_2_ repeat expanded RNAs contribute to C9FTD/ALS and thus are targets of intervention is less clear. Although PR translated from the antisense strand is extremely toxic in model systems, PR or its aggregates are rare in postmortem tissues. Antisense RNA transcripts are also hard to detect. Surprisingly, several studies showed that antisense RNA foci are as abundant as sense RNA foci in multiple brain regions (DeJesus-Hernandez et al., 2017; Mizielinska et al., 2013; Vatsavayai et al., 2019), raising a possibility that antisense C_4_G_2_ repeat expanded RNAs also contribute to C9FTD/ALS (McEachin et al., 2020b). In this study, we show that antisense *C9ORF72* C_4_G_2_ repeat expanded RNAs are neurotoxic independent of RAN translated DPR proteins. Antisense C_4_G_2_, but not sense G_4_C_2_, repeat expanded RNAs activate PKR/eIF2α-dependent integrated stress response, leading to global protein synthesis inhibition and stress granules formation. Moreover, the phosphorylation of PKR/eIF2α is significantly increased in C9FTD/ALS patients, suggesting that antisense C_4_G_2_ repeat expanded RNAs contribute to disease pathogenesis.

## Results

### *C9ORF72* antisense C_4_G_2_ expanded repeats are neurotoxic

**Figure 1.**
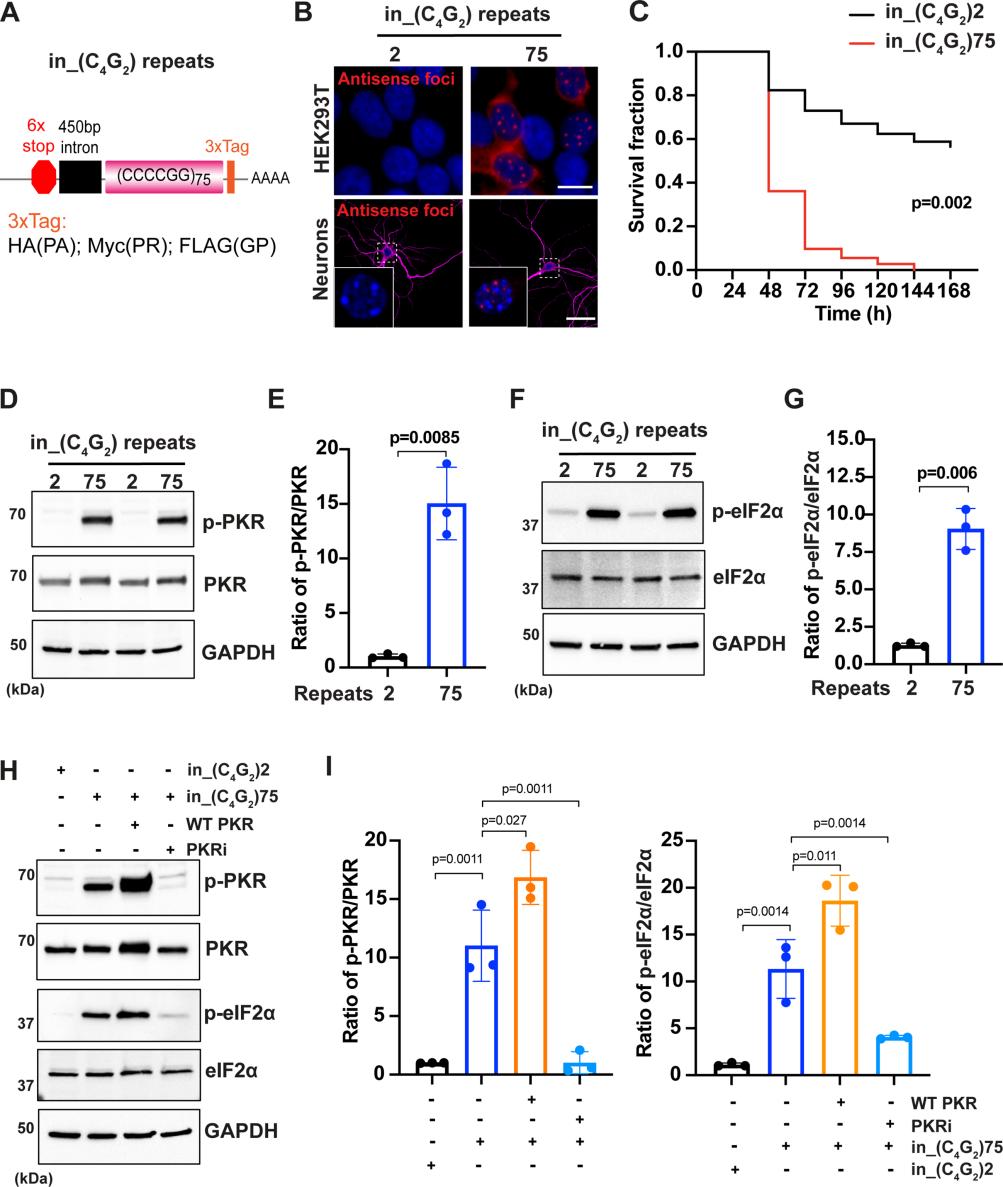
*C9ORF72* antisense C_4_G_2_ expanded repeats activate PKR/eIF2α-dependent integrated stress response and cause neuronal toxicity. (**A**) Schematic illustration of the in_(C_4_G_2_)75 repeat construct including 6× stop codons, 450 bp of human intronic sequences at the N-terminus and 3× protein tags at the C- terminus of the repeats to monitor the DPR proteins in each frame. (**B**) Representative images of antisense RNA foci in HEK293T cells and in primary cortical neurons expressing in_(C_4_G_2_)75 detected by RNA FISH. Red, foci; blue, DAPI; magenta, MAP2. (**C**) Kaplan–Meier curves showing increased risk of cell death in in_(C_4_G_2_)75-expressing primary cortical neurons compared with neurons expressing 2 repeats. Statistical analyses were performed using Mantel–Cox test (replicated three times with similar results). (**D, E**) Immunoblotting analysis of phosphorylated PKR (p-PKR) and total PKR in HEK293T cells expressing in_(C_4_G_2_)75 or 2 repeats. p-PKR levels were detected using anti-p-PKR (T446) and normalized to total PKR. GAPDH was used as a loading control. Error bars represent SD (n = 3 independent experiments). Statistical analyses were performed using Student’s *t*-test. (**F, G**) Immunoblotting analysis of phosphorylated eIF2α (p-eIF2α) and total eIF2α in HEK293T cells expressing in_(C_4_G_2_)75 or 2 repeats. p-eIF2α levels were detected using anti-phosphor eIF2α (Ser51) and normalized to total eIF2α. GAPDH was used as a loading control. Error bars represent SD (n = 3 independent experiments). Statistical analyses were performed using Student’s *t*-test. (**H, I**) Immunoblotting analysis of p-PKR and p-eIF2α in HEK293T cells expressing in_(C_4_G_2_)75, with or without co-expression of wild type PKR, or treatment of a PKR inhibitor, C16. Error bars represent SD (n = 3 independent experiments). Statistical analyses were performed using one-way ANOVA with Tukey’s post hoc test. Scale bars: 10 µm (neurons), 20 µm (HEK293T). Figure 1—source data 1.Original western blot results for Figure 1D and F. Figure 1—source data 2.Original western blot results for Figure 1H.

To determine the contribution of *C9ORF72* antisense repeat expanded RNAs in FTD/ALS pathogenesis, we first generated a construct containing 75 C_4_G_2_ repeats using recursive directional ligation as previously described (Mizielinska et al., 2014). We included six stop codons (two every frame) at the N-terminus to prevent unwarranted translation initiation and three protein tags in frame with individual DPR proteins at the C-terminus (Figure 1A). Recent studies have shown that the nucleotide sequences at 5′- and 3′- regions of expanded repeats regulate toxicity (He et al., 2020; Sellier et al., 2017). Although the molecular mechanism of *C9ORF72* antisense transcription initiation is unknown, it has been shown that transcription can start from at least 450 bp nucleotides upstream (Zu et al., 2013). We therefore added 450 bp of human sequence at the 5′- region of the antisense C_4_G_2_ repeats and termed this construct as ‘in_(C_4_G_2_)75’. When expressed in HEK293T cells, we detected an abundant accumulation of antisense RNA foci, but not in control cells expressing 2 C_4_G_2_ repeats (Figure 1B). Using antibodies against individual DPR proteins RAN translated from C_4_G_2_ expanded repeat RNAs or the protein tags in frame, we also observed production of GP, PR, and PA DPR proteins only in cells expressing in_(C_4_G_2_)75 but not 2 repeats (Figure 1—figure supplement 1A and B). Antisense RNA foci and DPR proteins were also observed in mouse primary cortical neurons expressing in_(C_4_G_2_)75, but not in neurons expressing 2 repeats (Figure 1B and Figure 1—figure supplement 1C). Thus, in_(C_4_G_2_)75 produces antisense RNA foci and DPR proteins, two cellular pathological hallmarks observed in C9FTD/ALS patients.

**Figure 1—figure supplement 1.**
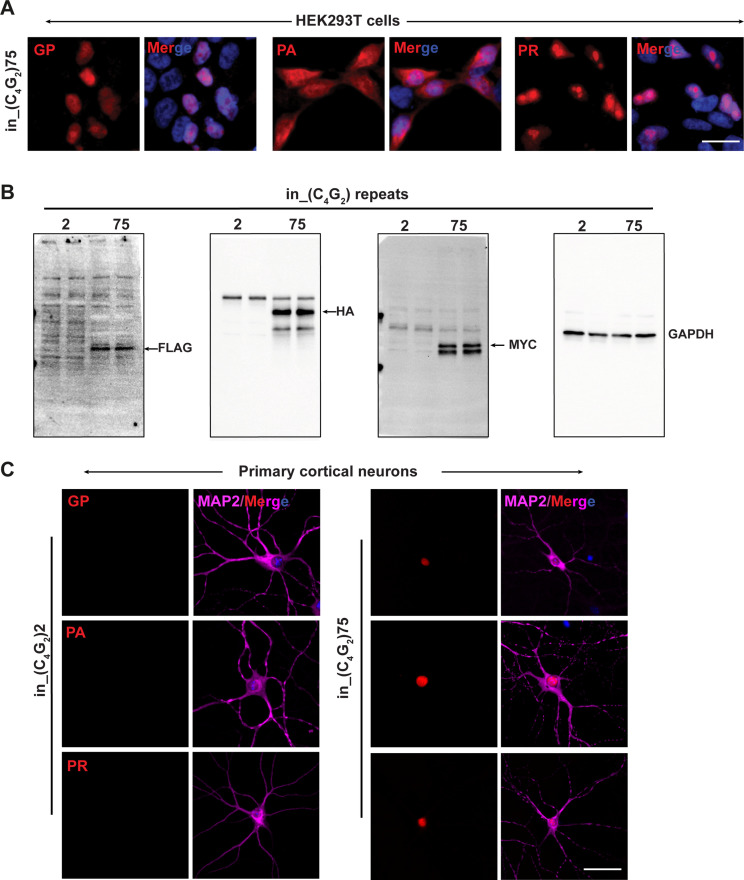
*C9ORF72* C_4_G_2_ repeat expanded repeats produce antisense DPR proteins in HEK293T cells and primary neurons. (**A**) Representative images of DPR protein staining in HEK293T expressing in_(C_4_G_2_)75 repeats using DPR antibodies. Red, GP, PA, and PR; blue, DAPI. (**B**) Immunoblotting of DPR proteins in HEK293T expressing in_(C_4_G_2_)75 repeats using TAG antibodies. GAPDH was used as a loading control. (**C**) Representative images of DPR protein staining in primary neurons expressing in_(C_4_G_2_)75 repeats using DPR antibodies. Red, GP, PA, and PR; blue, DAPI; MAP2, magenta. Scale bars, 10 µm (neurons), 20 µm (HEK293T). Figure 1—figure supplement 1—source data 1.Original western blot results for Figure 1—figure supplement 1B.

**Figure 1—figure supplement 2.**
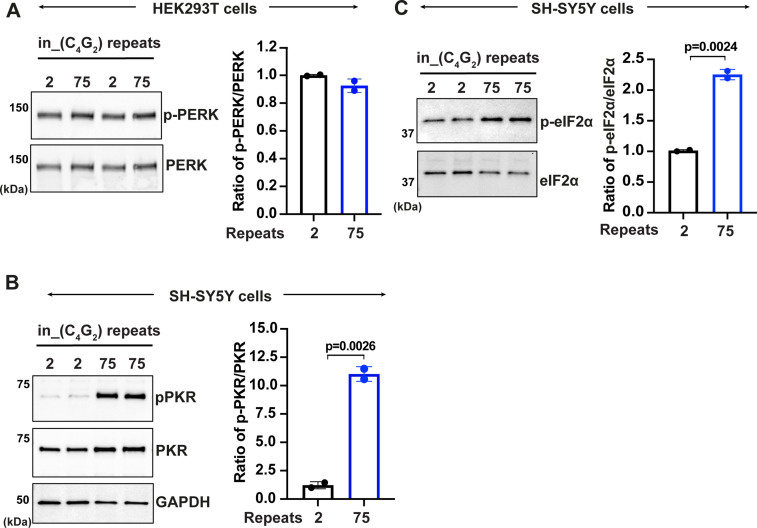
*C9ORF72* C_4_G_2_ repeat expanded repeats activate PKR/eIF2α-dependent integrated stress response in SH-SY5Y cells. (**A**) Immunoblotting of p-PERK in HEK293T cells expressing in_(C_4_G_2_)75 or (C_4_G_2_)2 repeats. Phosphorylated PERK levels were quantified and normalized to total PERK. GAPDH was used as a loading control. Error bars represent SD (n = 2 independent experiments). Statistical analyses were performed using Student’s *t*-test. (**B, C**) Immunoblotting of p-PKR and p-eIF2α in SH-SY5Y cells expressing (C_4_G_2_)75 or (C_4_G_2_)2 repeats. p-PKR (T446) and p-eIF2α (Ser51) were normalized to total PKR and eIF2α, respectively. GAPDH was used as a loading control. Error bars represent SD (n = 2 independent experiments). Statistical analyses were performed using Student’s *t*-test. Figure 1—figure supplement 2—source data 1.Original western blot results for Figure 1—figure supplement 2B and C.

To determine whether *C9ORF72* antisense C_4_G_2_ expanded repeats can cause neuronal toxicity, we co-transfected in_(C_4_G_2_)75 or control 2 repeats together with mApple in mouse primary cortical neurons at 4 days in vitro (DIV4) and used automated longitudinal microscopy to track over days the survival of hundreds of neurons as indicated by the mApple fluorescence. Neurons expressing in_(C_4_G_2_)75 die much faster than those expressing control 2 repeats, suggesting that *C9ORF72* antisense C_4_G_2_ expanded repeats are neurotoxic (Figure 1C).

### *C9ORF72* antisense C_4_G_2_ expanded repeats activate PKR/eIF2α-dependent integrated stress response

We next investigated the molecular mechanism underlying toxicity caused by *C9ORF72* antisense C_4_G_2_ expanded repeats. More than 50 neurological diseases are genetically associated with microsatellite repeat expansions. Repeat expanded RNAs, including CAG, CUG, CCUG, CAGG, and G_4_C_2_, have been shown to activate the double-stranded RNA-dependent protein kinase (PKR) (Handa et al., 2003; Tian et al., 2000). We hypothesized that *C9ORF72* antisense C_4_G_2_ expanded RNAs can also activate PKR. HEK293T cells expressing in_(C_4_G_2_)75 show a significant increase in the level of phosphorylated PKR compared to cells expressing 2 repeats, while the total level of PKR remains unchanged (Figure 1D and E). PKR is one of four kinases that are activated during the integrated stress response (ISR), an evolutionarily conserved stress signaling pathway that adjusts cellular biosynthetic capacity according to need (Martinez et al., 2021). The four ISR kinases, including PKR, PKR-like ER kinase (PERK), heme-regulated eIF2α kinase (HRI), and general control non-derepressible 2 (GCN2), respond to distinct environmental and physiological stresses by phosphorylating the eukaryotic translation initiation factor eIF2α to cause a temporary shutdown of global protein synthesis and upregulation of specific stress-responsive genes. Accompanying PKR activation, in_(C_4_G_2_)75 significantly increases the phosphorylation of eIF2α without affecting its total level (Figure 1F and G). in_(C_4_G_2_)75 activates eIF2α mainly by the phosphorylation of PKR as other ISR kinases such as PERK phosphorylation are not altered (Figure 1—figure supplement 2A). Consistent with this, overexpressing wild type (WT) PKR further increases the phosphorylation of eIF2α induced by in_(C_4_G_2_)75, whereas treatment with a specific PKR inhibitor C16 reduces the phosphorylation of both PKR and eIF2α to a level comparable to that of cells expressing 2 repeats (Figure 1H and I). We further expressed in_(C_4_G_2_)75 in a neuronal cell line SH-SY5Y that is commonly used to study neurodegeneration and observed similar activation of PKR and eIF2α by the antisense C_4_G_2_ expanded repeats (Figure 1—figure supplement 2B and C).

**Figure 2.**
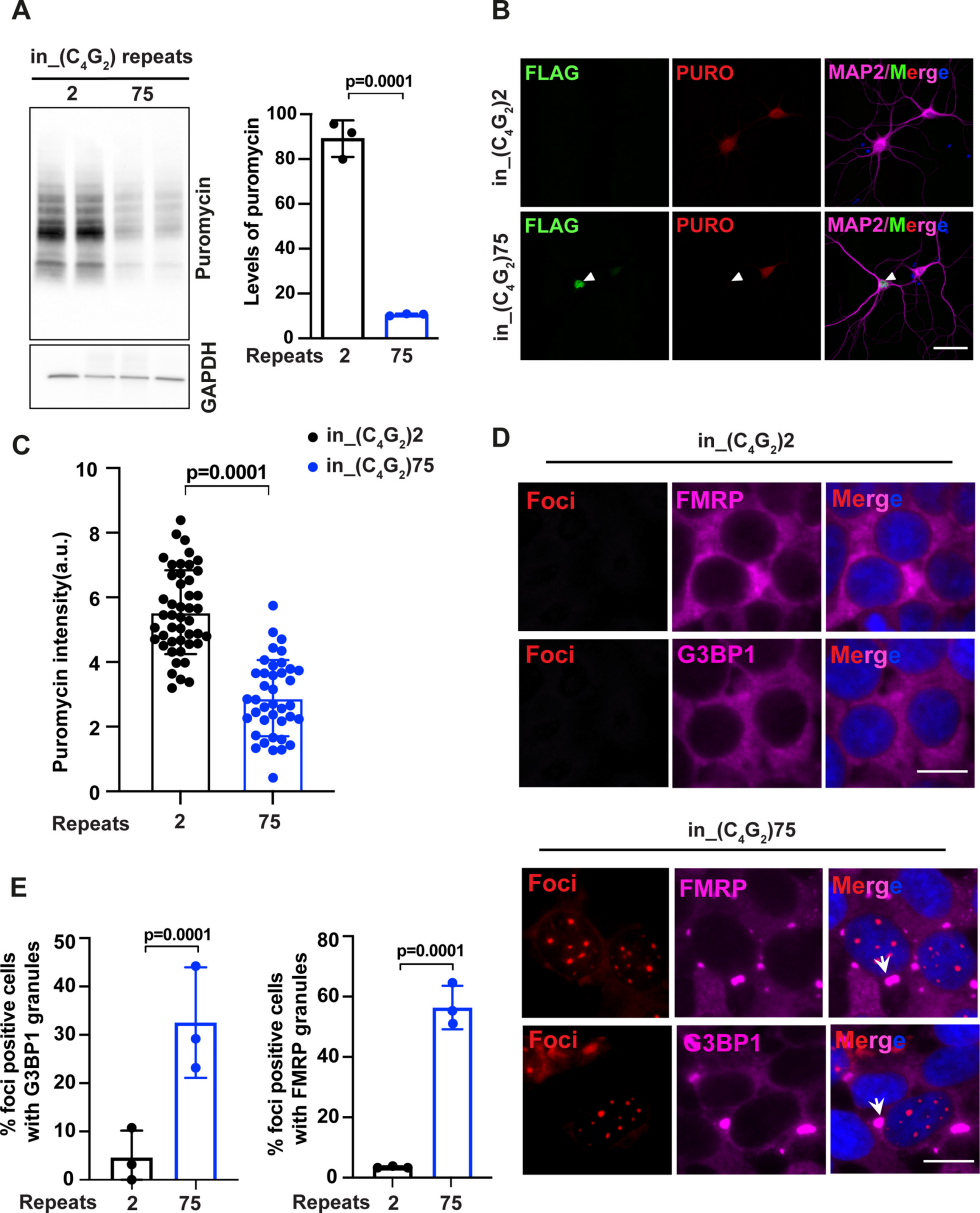
*C9ORF72* antisense C_4_G_2_ expanded repeats inhibit global protein synthesis and induce stress granule assembly. (**A**) Immunoblotting of puromycin in HEK293T cells expressing in_(C_4_G_2_)75 or 2 repeats. Cells were incubated with puromycin for 30 min before harvesting. The level of puromycin was normalized to GAPDH. Error bars represent SD (n = 3 independent experiments). Statistical analyses were performed using Student’s *t*-test (**B**) Representative images and (**C**) quantification of primary neurons expressing either (C_4_G_2_)75 or 2 repeats stained with anti-puromycin (red), anti-FLAG (green), DAPI (blue), and MAP2 (magenta). The puromycin intensity was quantified using ImageJ. Error bars represent SD (n = 40–50 neurons/group; similar results were obtained from two independent experiments). Statistical analyses were performed using Student’s *t*-test. (**D**) Representative images of G3BP1 and FMRP staining in HEK293T cells expressing in_(C_4_G_2_)75 and in_(C_4_G_2_)2 repeats. (**E**) Quantification of antisense foci-positive cells with G3BP1 and FMRP granules. Error bars represent SD (n = 150 cells/condition and three independent experiments). Statistical analyses were performed using Student’s *t*-test. Scale bars, 10 µm (neurons), 20 µm (HEK293T). Figure 2—source data 1.Original western blot results for Figure 1A.

To determine whether the activation of PKR/eIF2α leads to a global mRNA translation inhibition, we employed a puromycin-based, nonradioactive method to monitor protein synthesis (Schmidt et al., 2009). Puromycin is a structural analog of aminoacyl-tRNA that incorporates into nascent polypeptide chains and prevents elongation. The amount of incorporated puromycin detected by antibodies reflects global translation efficacy. HEK293T cells expressing in_(C_4_G_2_)75 show a significantly reduced amount of incorporated puromycin compared to those expressing 2 repeats (Figure 2A). Similarly, neurons expressing 75 antisense C_4_G_2_ repeats, as identified by GP DPR protein accumulation, have a robust global translation inhibition (Figure 2B and C). We also observed an abundant accumulation of stress granules in response to stress-induced translation inhibition. Approximately 32% of cells expressing in_(C_4_G_2_)75, identified by the presence of antisense RNA foci, show G3BP1-positive stress granules, and ~55% of foci-positive cells stain for FMRP, another commonly used marker for stress granules (Figure 2D and E). These results support that *C9ORF72* antisense C_4_G_2_ expanded repeats activate the PKR/eIF2α-dependent integrated stress response, leading to global translation inhibition and stress granule formation.

### Antisense C_4_G_2_ repeat expanded RNAs activate the PKR/eIF2α pathway independent of DPR proteins

**Figure 3.**
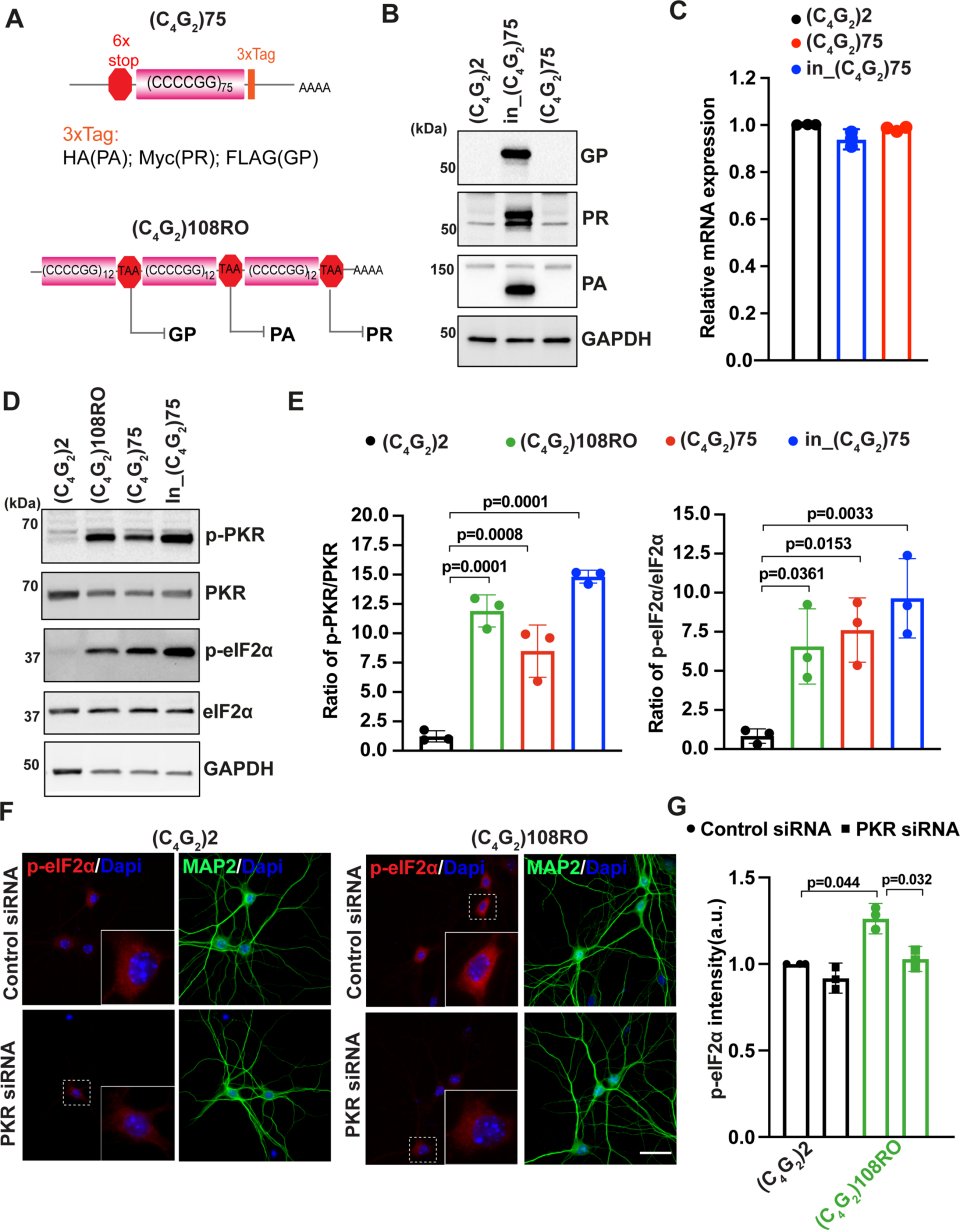
Antisense C_4_G_2_ repeat expanded RNAs activate the PKR/eIF2α pathway independent of DPR proteins. (**A**) (Top) Schematic illustration of (C_4_G_2_)75 repeats without the human intronic sequences. 3× protein tags were included at the C- terminus of the repeats to monitor the DPR proteins in each frame. (Bottom) Schematic illustration of antisense (C_4_G_2_)108RO repeats with stop codons inserted in every 12 repeats to prevent the translation of DPR proteins from all reading frames. (**B**) Immunoblotting of DPR proteins in HEK293T cells expressing in_(C_4_G_2_)75, (C_4_G_2_)75, or 2 repeats. DPR protein levels were detected using anti-FLAG (frame with GP), anti-MYC (frame with PR), and anti-HA (frame with PA). GAPDH was used as a loading control. (**C**) mRNA levels were measured by quantitative qPCR in cell expressing in_(C_4_G_2_)75, (C_4_G_2_)75, or 2 repeats. Error bars represent SD (n = 3). (**D, E**) Immunoblotting of p-PKR and p-eIF2α in HEK293T cells expressing in_(C_4_G_2_)75, (C_4_G_2_)75, (C_4_G_2_)108RO, or 2 repeats. p-PKR (T446) and p-eIF2α (Ser51) were normalized to total PKR and eIF2α, respectively. GAPDH was used as a loading control. Error bars represent SD (n = 3 independent experiments). Statistical analyses were performed using one-way ANOVA with Tukey’s post hoc test. (**F, G**) Representative images (**F**) and quantitation (**G**) of p-eIF2α in primary neurons expressing antisense (C_4_G_2_)108RO or 2 repeats in the presence and absence of PKR siRNA. Scale bars, 10 µm. Figure 3—source data 1.Original western blot results for Figure 3B. Figure 3—source data 2.Original western blot results for Figure 3D.

We next determined whether the activation of PKR/eIF2α-dependent integrated stress response is driven by repeat RNA themselves and/or by DPR proteins. We first expressed individual codon-optimized, ATG-driven DPR proteins. Neither PR50, PA50, nor GP80 activates the phosphorylation of eIF2α, suggesting that the activation of the PKR/eIF2α pathway by *C9ORF72* antisense C_4_G_2_ expanded repeats is unlikely due to the DPR proteins produced from RAN translation (Figure 3—figure supplement 1A and B). To obtain direct evidence that *C9ORF72* antisense repeat expanded RNAs themselves activate PKR/eIF2α, we used two strategies to reduce/inhibit DPR proteins without affecting the RNA. First, recent studies have shown that *C9ORF72* sense G_4_C_2_ repeat expanded RNAs initiate RAN translation at a near-cognate CUG codon in the intronic region 24 nucleotides upstream of the repeat sequence (Green et al., 2017; Tabet et al., 2018). We thus hypothesized that RAN translation from C_4_G_2_ antisense repeat expanded RNAs might similarly depend on the intronic sequence at the 5′ region. We generated a new construct (C_4_G_2_)75 without including the 450 bp human intronic sequence (Figure 3A). Supporting our hypothesis, cells expressing (C_4_G_2_)75 do not accumulate any detectable GP, PA, or PR DPR proteins, which is strikingly different compared to those expressing in_(C_4_G_2_)75 with the 450 bp human intronic sequence (Figure 3B). The reduced/abolished DPR proteins by (C_4_G_2_)75 are not due to altered RNA expressions since the levels of RNA transcripts and antisense foci are comparable to those of in_(C_4_G_2_)75 (Figure 3C and Figure 3—figure supplement 1C). Second, we obtained a previously reported stop codon-interrupted 108 antisense repeat construct, designated as RNA only (RO) [(C_4_G_2_)108RO] (Figure 3A). It has been shown that this construct is not RAN translated to produce DPR proteins, while still adopting similar stable conformations as the uninterrupted repeat RNAs (Moens et al., 2018). As expected, no detectable antisense DPR proteins are observed in cells expressing (C_4_G_2_)108RO, despite an abundant accumulation of antisense foci (Figure 3—figure supplement 1C and D). Interestingly, expression of either (C_4_G_2_)75 or (C_4_G_2_)108RO leads to the robust activation of PKR and eIF2α at a comparable level as seen for in_(C_4_G_2_)75 (Figure 3D and E). To further validate the above results in a disease-relevant cell type, we made lentivirus to express (C_4_G_2_)108RO in primary cortical neurons. We observed the presence of antisense foci in nearly 100% of (C_4_G_2_)108RO-expressing neurons (Figure 3—figure supplement 1E) together with increased levels of phosphorylated eIF2α (Figure 3F and G). Since the available commercial antibodies against mouse phosphorylated PKR do not work in our hands, we designed siRNAs targeting mouse PKR. Reducing PKR in (C_4_G_2_)108RO-expressing neurons mitigates the elevated levels of phosphorylated eIF2α (Figure 3F and G and Figure 3—figure supplement 1F and G). These results support that *C9ORF72* antisense C_4_G_2_ repeat expanded RNAs activate the PKR/eIF2α pathway independent of DPR proteins.

**Figure 3—figure supplement 1.**
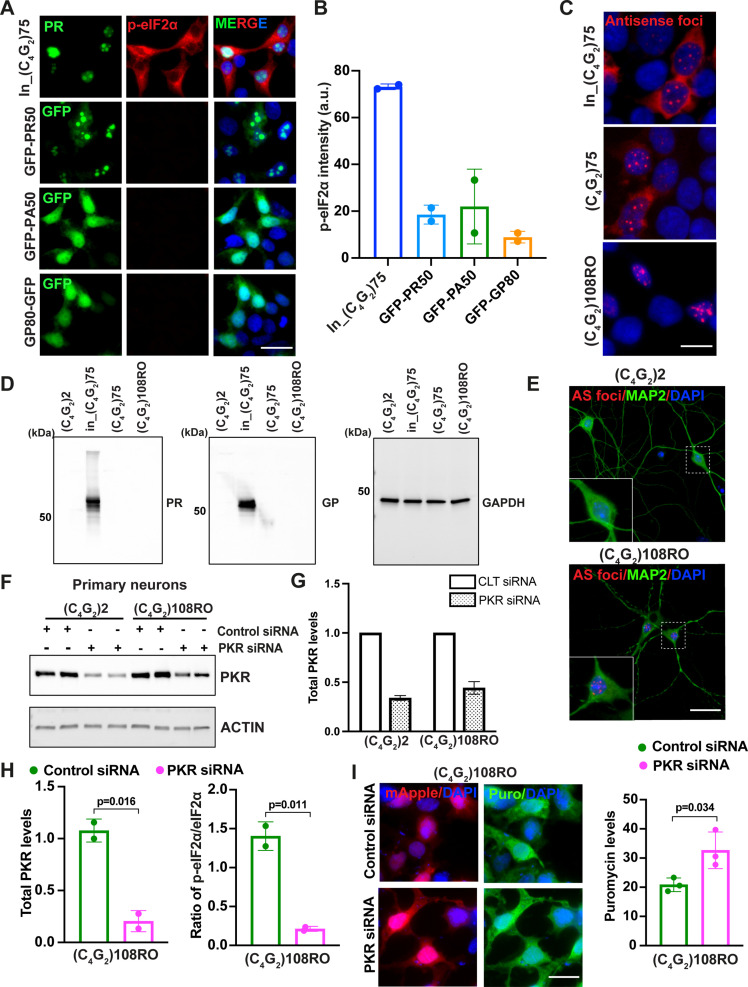
Antisense DPR proteins do not activate PKR/eIF2α-dependent integrated stress response. (**A**) Representative images and (**B**) quantification of p-eIF2α staining in HEK293T cells expressing in_(C_4_G_2_)75, PR50, PA50, or GP80. Green, GP, PA, or PR; red, p-eIF2α; blue, DAPI. Error bars represent SD (n = 2 independent experiments). (**C**) Representative images of antisense RNA foci in HEK293T cells expressing in_(C_4_G_2_)75, (C_4_G_2_)75 or (C_4_G_2_)108RO repeats. Foci were detected by RNA FISH. Red, foci; blue, DAPI. (**D**) Immunoblotting of DPR proteins in HEK293T cells expressing in_(C_4_G_2_)75, (C_4_G_2_)75 and (C_4_G_2_)108RO repeats. DPR protein levels were detected using anti-PR and anti-GP antibodies. GAPDH was used as a loading control. (**E**) Representative images of antisense RNA foci in primary cortical neurons expressing (C_4_G_2_)108 detected by RNA FISH. Red, foci; blue, DAPI; Green, MAP2. (**F**) Immunoblotting and (**G**) quantification of PKR in primary neurons expressing (C_4_G_2_)2 and (C_4_G_2_)108RO together with control or PKR siRNA. PKR were normalized to GADPH. Error bars represent SD. (**H**) The levels of PKR and p-eIF2α (Ser51) in HEK293T cells expressing (C_4_G_2_)108RO together with control or PKR siRNA. PKR and p-eIF2α (Ser51) were normalized to GADPH and total eIF2α, respectively. Error bars represent SD (n = 2 independent experiments). Statistical analyses were performed using Student’s *t*-test. (**I**) Representative images and quantification of puromycin staining in HEK293T cells expressing (C_4_G_2_)108RO together with control or PKR siRNA. Error bars represent SD (n = 3 independent experiments). mApple was co-transfected to identify cells with (C_4_G_2_)108RO expression. Statistical analyses were performed using Student’s *t*-test. Scale bars, 20 µm (HEK293T), 10 µm (neurons). Figure 3—figure supplement 1—source data 1.Original western blot results for Figure 3—figure supplement 1D. Figure 3—figure supplement 1—source data 2.Original western blot results for Figure 3—figure supplement 1F.

### Antisense C_4_G_2_ repeat expanded RNAs themselves induce stress granules and lead to neuronal toxicity

**Figure 4.**
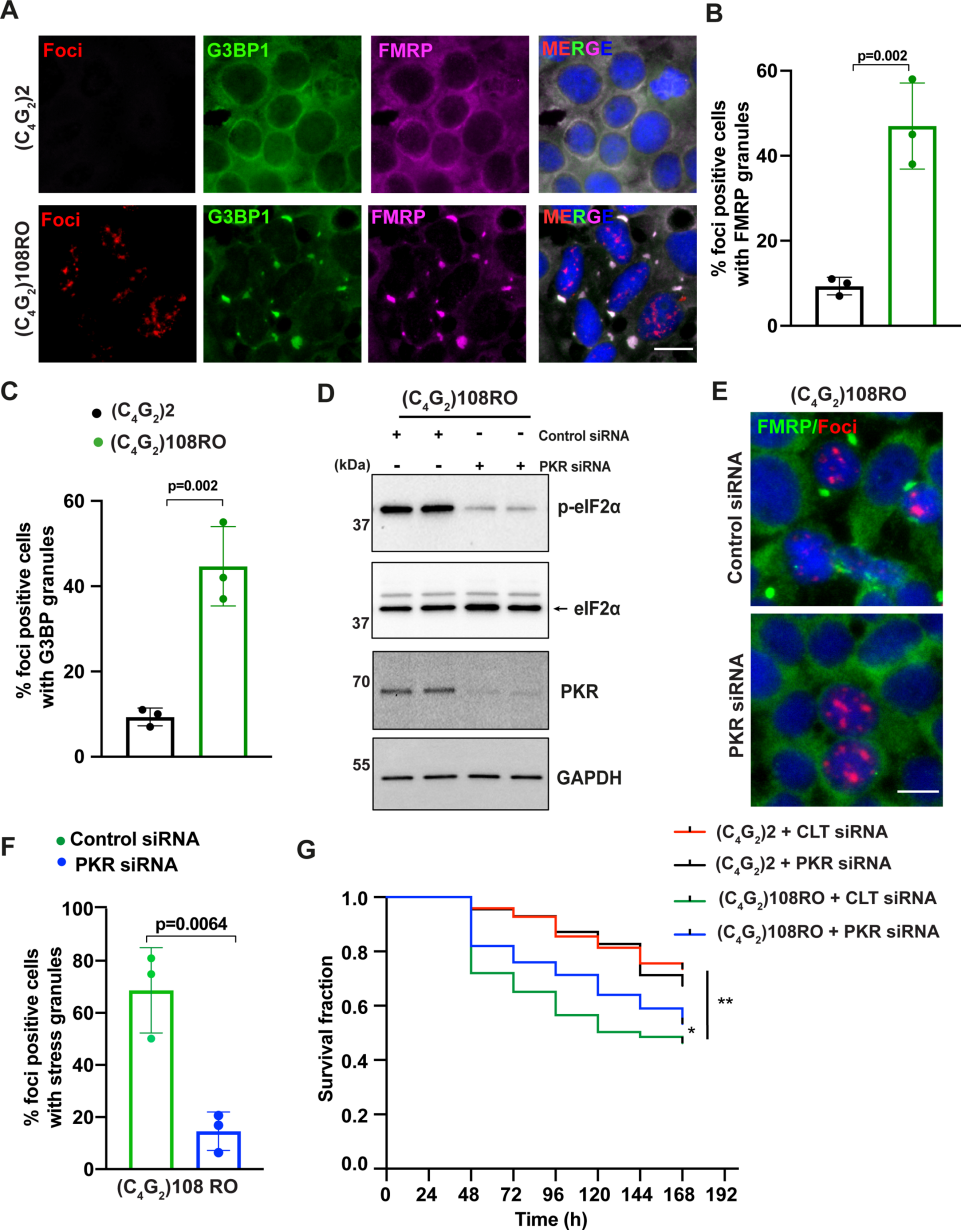
Antisense C_4_G_2_ repeat expanded RNAs themselves induce stress granules and lead to neuronal toxicity. (**A**) Representative images of FMRP and G3BP1 staining in HEK293T cells expressing (C_4_G_2_)108RO or 2 repeats. (**B, C**) Quantification of antisense foci-positive cells with FMRP and G3BP1 granules. Error bars represent SD (n = 180 cells/condition and three independent experiments). Statistical analyses were performed using Student’s *t*-test. (**D**) Immunoblotting of PKR and p-eIF2α (Ser51) in HEK293T cells expressing (C_4_G_2_)108RO together with control or PKR siRNA. GAPDH was used as a loading control. (**E**) Representative images of FMRP staining in HEK293T cells expressing (C_4_G_2_)108 repeats together with either control or PKR siRNA. (**F**) Quantification of antisense foci-positive cells with FMRP granules. Error bars represent SD (n = 150 cells/condition and three independent experiments). (**F**) Kaplan–Meier curves showing the risk of cell death in (C_4_G_2_)108RO-expressing neurons compared with 2 repeats in the presence and absence of PKR siRNA (replicated three times with similar results). Statistical analyses were performed using Mantel–Cox test (*p<0.05, **p<0.01). Scale bars, 20 µm. Figure 4—source data 1.Original western blot results for Figure 4D.

Given the conflicting reports of whether *C9ORF72* antisense RNAs themselves are toxic independent of DPR proteins (Moens et al., 2018; Swinnen et al., 2018; Tran et al., 2015), we next focused on (C_4_G_2_)108RO, which does not produce DPR proteins but activates the PKR/eIF2α-dependent ISR pathway. We first determined whether the interrupted repeats are sufficient to induce stress granules, which is one of the downstream pathways initiated by phosphorylated eIF2α. We found that FMRP and G3BP1 are mainly diffused in the cytoplasm of cells expressing 2 C_4_G_2_ repeats as expected, but they rapidly assemble into stress granules in cells expressing (C_4_G_2_)108RO (Figure 4A–C). These data suggest that antisense C_4_G_2_ repeat expanded RNAs themselves can trigger stress granule formation in the absence of DPR proteins. To determine the role of PKR activation in stress granule formation by (C_4_G_2_)108RO, we knocked down *PKR* using siRNAs. siRNAs targeting *PKR* reduce its protein level by 80% compared to control siRNAs (Figure 4D and Figure 3—figure supplement 1H). Consequently, the phosphorylation of eIF2α by (C_4_G_2_)108RO is almost inhibited (Figure 4D and Figure 3—figure supplement 1H) and the percentage of foci-positive cells with stress granules is significantly reduced (Figure 4E and F). Furthermore, a slight but significant increase in the global protein synthesis is also observed (Figure 3—figure supplement 1I). Thus, *C9ORF72* antisense C_4_G_2_ repeat expanded RNAs themselves induce stress granules by activating PKR/eIF2α.

We further utilized the unbiased longitudinal microscopy assay to determine the risk of death in neurons expressing (C_4_G_2_)108RO. Rodent primary cortical neurons were transfected with mApple and (C_4_G_2_)108RO or 2 repeats and imaged at 24 hr intervals for 7 d. Neurons expressing (C_4_G_2_)108RO show a significant decrease in survival compared to control neurons expressing 2 repeats (Figure 4G). Importantly, knockdown of PKR partially rescues the (C_4_G_2_)108RO-mediated toxicity (Figure 4G). These data suggest that *C9ORF72* antisense repeat expanded RNAs themselves are neurotoxic via PKR activation.

### Increased levels of phosphorylated PKR and eIF2α in C9FTD/ALS patients

**Figure 5.**
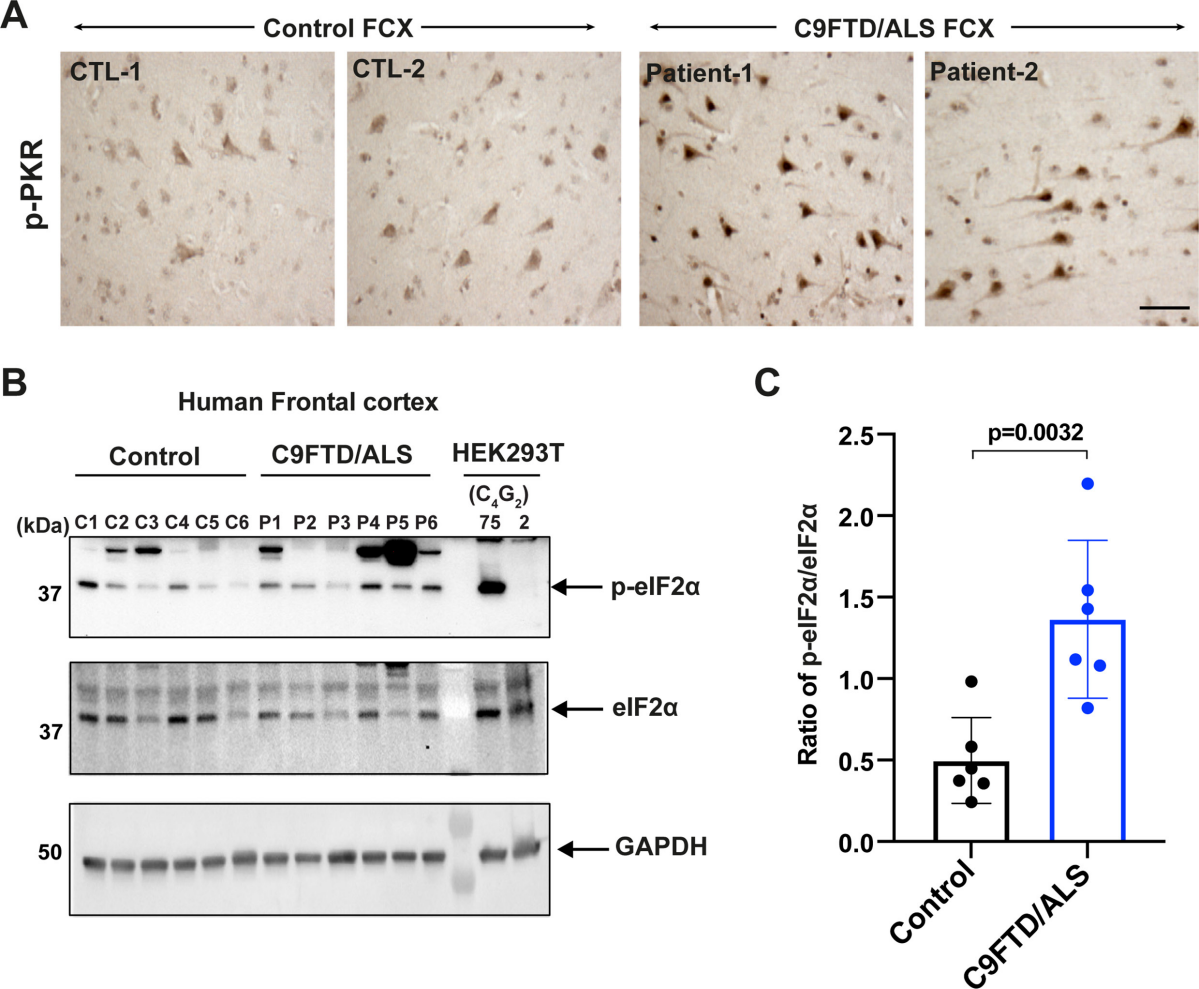
Increased levels of phosphorylated PKR and eIF2α in C9FTD/ALS patients. (**A**) Representative immunohistochemistry images of phosphorylated PKR staining in control and C9FTD/ALS patient’s frontal cortex (FCX) using anti-p-PKR (T446) (n = 4 per genotype). (**B, C**) Immunoblotting of p-eIF2α in proteins extracted from control (C1–C6) and C9FTD/ALS patient’s frontal cortex (P1–P6). p-eIF2α (Ser51) was normalized to total eIF2α. GAPDH was used as a loading control. Error bars represent SD (control n = 6 and C9FTD/ALS n = 6). Statistical analyses were performed using unpaired Student’s *t*-test. Scale bars, 10 µm. Figure 5—source data 1.Original western blot results for Figure 5B.

To study disease relevance, we next determined the levels of phosphorylated PKR and eIF2α in C9FTD/ALS patient postmortem tissues and in patient-derived iPSCs motor neurons. Immunohistochemistry staining showed that the level of phosphorylated PKR is increased in the frontal cortex, especially in the large pyramidal neurons, of patients carrying *C9ORF72* repeat expansions compared to age-matched non-disease controls (Figure 5A). This data is consistent with two recent studies showing increased PKR phosphorylation in *C9ORF72* patients (Rodriguez et al., 2021; Zu et al., 2020). In addition, the level of phosphorylated eIF2α after normalizing to the total eIF2α level is also significantly increased, despite the heterogeneity of eIF2α protein levels in patients (Figure 5B and C, Table 1). These results suggest that the PKR/eIF2α pathway is activated in C9FTD/ALS patients. We also obtained protein extracts from two lines of *C9ORF72* patient-derived motor neurons and their isogenic controls (Braems et al., 2022). However, we did not observe any differences in the levels of either phosphorylated PKR or phosphorylated eIF2α of 38-day-old neurons (Figure 5—figure supplement 1A and B), suggesting that PKR/ eIF2α-dependent ISR pathway possibly gets activated in *C9ORF72* patients in a later stage of disease pathogenesis.

**Figure 5—figure supplement 1.**
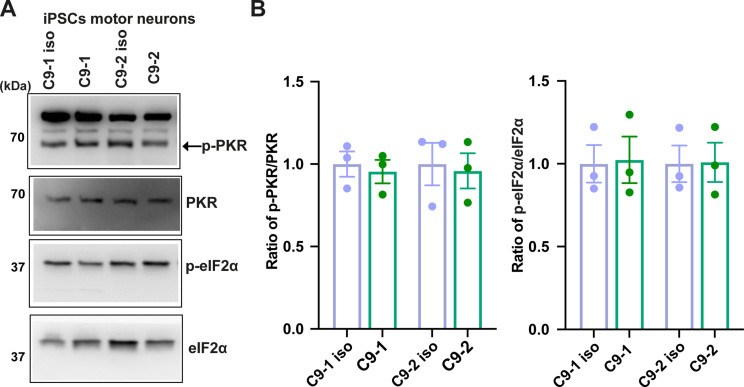
Phosphorylated PKR and eIF2α levels are unchanged in C9FTD/ALS iPSC-derived motor neurons. (**A**) Immunoblotting and (**B**) quantification of p-PKR and p-eIF2α in two different lines of iPSC-derived motor neurons of C9FTD/ALS patient and their isogenic controls (38 d post differentiation). P-PKR levels were detected using anti-p-PKR (T446) and normalized to total PKR. P-eIF2α levels were detected using anti-phosphor eIF2α (Ser51) and normalized to total eIF2α. Error bars represent SD (n = 3). Figure 5—figure supplement 1—source data 1.Original western blot results for Figure 5—figure supplement 1A.

**Table 1.** List of controls (C1–C6) and C9FTD/ALS patients (P1–P6) postmortem tissues used in this study.

ID	Primary neuropathological diagnosis	Age atonset (years)	Age atdeath (years)	Sex
C1	Control	-	53	F
C2	Control	-	77	NA
C3	Control	-	43	F
C4	Control	-	57	M
C5	Control	-	72	F
C6	Control	-	57	F
P1	FTLD (C9 expansion)	58	71	M
P2	ALS (C9 expansion)	54	57	F
P3	FTLD (C9 expansion)	57	66	M
P4	FTLD (C9 expansion)	58	67	M
P5	FTLD (C9 expansion)	62	66	M
P6	ALS (C9 expansion)	-	69	NA

ALS: amyotrophic lateral sclerosis.

### Sense G_4_C_2_ repeat expanded RNAs cannot activate the PKR/eIF2α pathway

**Figure 6.**
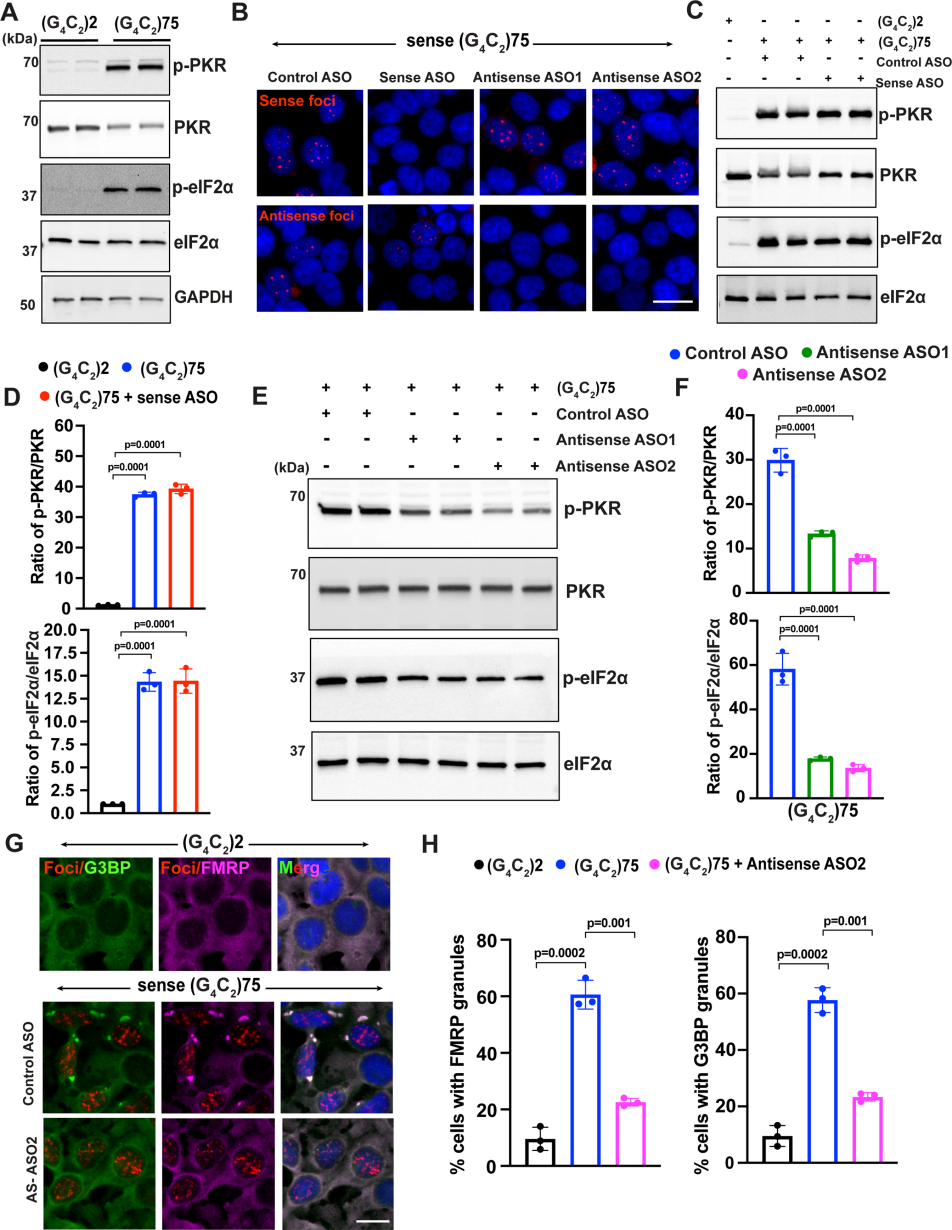
Sense G_4_C_2_ repeat expanded RNAs cannot activate the PKR/eIF2α pathway. (**A**) Immunoblotting of p-PKR and p-eIF2α in HEK293T cells expressing (G_4_C_2_)75 or (G_4_C_2_)2 repeats. (**B**) Representative images of sense and antisense RNA foci in HEK293T expressing (G_4_C_2_)75 repeats together with either control antisense oligonucleotides (ASOs), ASOs targeting sense G_4_C_2_RNAs or ASOs targeting antisense C_4_G_2_ repeat expanded RNAs. Foci were detected by RNA FISH. Red, foci; blue, DAPI. (**C**) Immunoblotting and (**D**) quantification of p-PKR and p-eIF2α in HEK293T cells expressing (G_4_C_2_)75 or (G_4_C_2_)2 repeats together with either control ASO or ASOs targeting sense G_4_C_2_ repeat expanded RNAs. Phosphorylated PKR levels were detected using anti-p-PKR (phosphor T446) and normalized to total PKR. Phosphorylated eIF2α levels were detected using anti-peIF2α (Ser51) and normalized to total eIF2α. GAPDH was used as a loading control. Error bars represent SD (n = 3 independent experiments). Statistical analyses were performed using unpaired Student’s *t*-test. (**E, F**) Immunoblotting of p-PKR and p-eIF2α in HEK293T cells expressing (G_4_C_2_)75 or (G_4_C_2_)2 repeats together with either control ASO or ASOs targeting antisense G_4_C_2_ repeat expanded RNAs. P-PKR (T446) and p-eIF2α (Ser51) were normalized to total PKR and eIF2α, respectively. GAPDH was used as a loading control. Error bars represent SD (n = 3 independent experiments). Statistical analyses were performed using one-way ANOVA with Tukey’s post hoc test. (**G**) Representative images and (**H**) quantification of FMRP and G3BP1 staining in HEK293T cells expressing (G_4_C_2_)75 or (G_4_C_2_)2 repeats in the presence and absence of antisense ASO2. Error bars represent SD (n = 150 cells/condition and three independent experiments). Statistical analyses were performed using one-way ANOVA with Tukey’s post hoc test. Scale bars, 20 µm. Figure 6—source data 1.Original western blot results for Figure 6A. Figure 6—source data 2.Original western blot results for Figure 6C. Figure 6—source data 3.Original western blot results for Figure 6E.

Recently, Zu et al. showed that expressing a construct containing (G_4_C_2_)120 also activates PKR and increases DPR protein translation in HEK293T cells (Zu et al., 2020). However, it is not known whether this activation is due to sense or antisense transcripts. Therefore, we generated a sense repeat construct (G_4_C_2_)75 that has a similar repeat size as our antisense construct (Figure 6—figure supplement 1A). Consistent with the earlier findings by Zu et al., expression of sense (G_4_C_2_)75 in HEK293T cells significantly increases phosphorylation of both PKR and eIF2α (Figure 6A and Figure 6—figure supplement 1B). However, we detected abundant accumulation of both sense and antisense RNA foci in cells expressing sense (G_4_C_2_)75 but not in those expressing 2 repeats (Figure 6—figure supplement 1C). Interestingly, we also detected accumulation of sense foci in cells expressing antisense (C_4_G_2_) expanded repeats in such model system (Figure 6—figure supplement 1C). We therefore aimed to determine the relative contribution of sense G_4_C_2_ and antisense C_4_G_2_ repeat expanded RNAs in activating the PKR/eIF2α pathway by focusing the expanded (G_4_C_2_)75 repeats which resemble C9FTD/ALS patients. We first used previously published ASOs that specifically degrade sense G_4_C_2_ RNAs (Jiang et al., 2016). As expected, ASOs targeting sense G_4_C_2_ repeat expanded RNAs significantly reduce the accumulation of sense RNA foci but have little effect on antisense RNA foci (Figure 6B and Figure 6—figure supplement 1D). However, reducing sense G_4_C_2_ RNA transcripts/foci does not alter the activation of PKR/eIF2α by (G_4_C_2_)75 (Figure 6C and D). We next designed two ASOs specifically targeting antisense repeat RNAs. Both ASO1 and ASO2 targeting antisense C_4_G_2_ repeat expanded RNAs significantly reduce the abundance of antisense RNA foci without affecting sense RNA foci (Figure 6B and Figure 6—figure supplement 1E). Consequently, both ASOs significantly inhibit the activation of PKR and eIF2α by (G_4_C_2_)75 (Figure 6E and F). We further obtained an interrupted sense (G_4_C_2_)108RO construct that has the similar repeat size as the antisense (C_4_G_2_)108RO (Moens et al., 2018). Expression of sense (G_4_C_2_)108RO also significantly increases the phosphorylation of PKR and eIF2α, which is mitigated by ASOs targeting antisense RNAs, but not sense RNAs (Figure 6—figure supplement 1F). These results suggest that antisense C_4_G_2_, but not sense G_4_C_2_, repeat expanded RNAs (either pure repeats or interrupted) activate the PKR/eIF2α-mediated ISR pathway. It has been shown previously that expressing sense repeats can induce stress granules (Green et al., 2017), but the underlying molecular mechanisms are not explored. We observed a significant increase in the accumulation of FMRP and G3BP1 granules in (G_4_C_2_)75 repeats expressing cells compared to those expressing the control 2 repeats. Interestingly, such aberrant accumulation of stress granules is drastically reduced after treatment with ASOs targeting the antisense G_4_C_2_ repeat RNAs (Figure 6G and H). Thus, antisense C_4_G_2_, but not sense G_4_C_2_, repeat expanded RNAs activate the PKR/eIF2α pathway, leading to toxicity.

**Figure 6—figure supplement 1.**
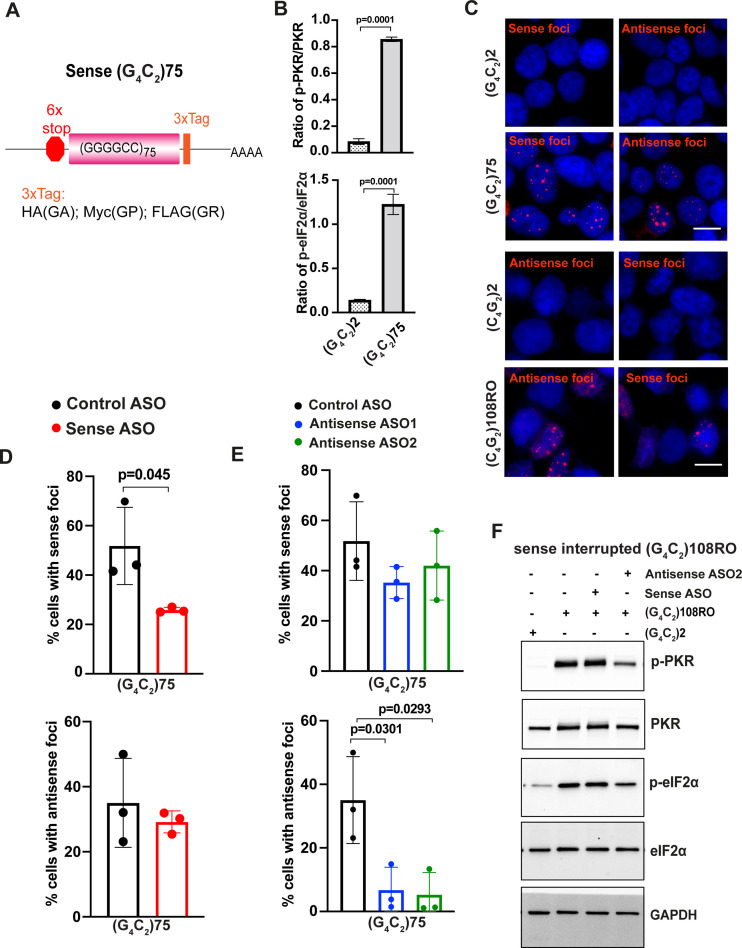
Sense G_4_C_2_ repeat expanded RNAs cannot activate the PKR/eIF2α pathway. (**A**) Schematic illustration of the sense (G_4_C_2_)75 repeat construct. (**B**) Quantification of p-PKR and p-eIF2α in HEK293T cells expressing (G_4_C_2_)75 or (G_4_C_2_)2 repeats. p-PKR (T446) and p-eIF2α (Ser51) were normalized to total PKR and eIF2α, respectively. GAPDH was used as a loading control. Error bars represent SD (n = 2 independent experiments). Statistical analyses were performed using Student’s *t*-test. (**C**) Representative images of sense and antisense RNA foci in HEK293T cells expressing sense (G_4_C_2_)75 and interrupted antisense (C_4_G_2_)108RO repeats. Foci were detected by RNA FISH. Red, foci; blue, DAPI. (**D, E**) Quantification of RNA foci in HEK293T expressing (G_4_C_2_)75 together with either control antisense oligonucleotide (ASO) or ASOs targeting sense G_4_C_2_ and antisense C_4_G_2_ repeat expanded RNAs. Error bars represent SD (n = 80–100 cells/condition from three independent experiments). (**F**) Immunoblotting of p-PKR and p-eIF2α in HEK293T cells expressing interrupted sense (G_4_C_2_)108RO and control repeats. p-PKR levels were detected using anti-p-PKR (T446) and normalized to total PKR. P-eIF2α levels were detected using anti-phosphor eIF2α (Ser51) and normalized to total eIF2α. Error bars represent. GAPDH was used as a loading control. Error bars represent SD Scale bars, 20 µm (HEK293T). Figure 6—figure supplement 1—source data 1.Original western blot results for Figure 6—figure supplement 1F.

### Reduction of PKR mitigates antisense C_4_G_2_, but not sense G_4_C_2_, RNA- mediated toxicity in zebrafish

**Figure 7.**
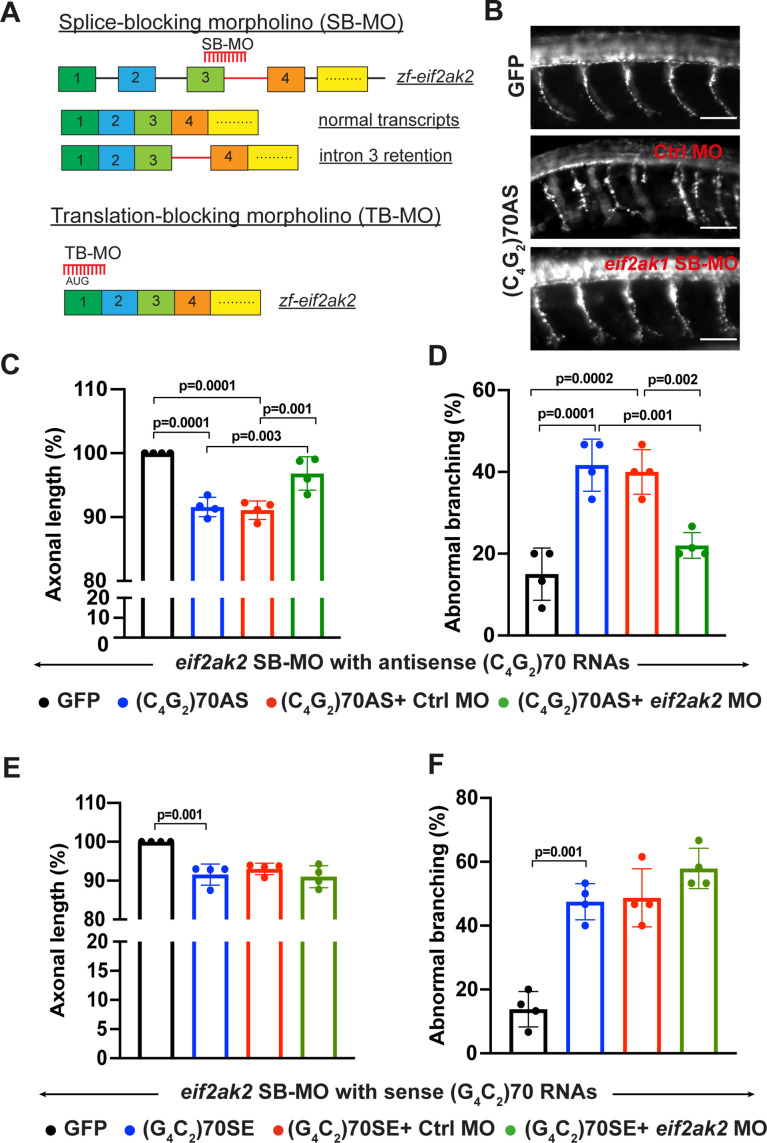
Reduction of PKR mitigates antisense C_4_G_2_, but not sense G_4_C_2_, RNA- mediated toxicity in zebrafish. (**A**) Schematic illustration of splice-blocking morpholino (SB-MO) targeting the exon 3/intron 3 junction in zebrafish *eif2ak2* pre-mRNA. The correctly spliced (wild type transcripts) and intron 3 retained transcripts are shown. The translation-blocking morpholino targets the AUG start codon in post-spliced *eif2ak2* mRNA. (**B**) SV2 immunostaining of motor axons in 30 hpf zebrafish embryos injected with GFP control mRNA and antisense (C_4_G_2_)70 RNAs. Scale bar = 50 µm. (**C, D**) Effects of SB-MO on axonal length (**C**) and branching (**D**) of zebrafish expressing antisense (C_4_G_2_)70 RNAs. Embryos were injected with 0.844 µM GFP mRNA, 0.844 µM antisense (C_4_G_2_)70 RNAs, 0.844 µM antisense (C_4_G_2_)70 RNAs plus 0.25 mM control morpholino, or 0.844 µM antisense (C_4_G_2_)70 RNAs plus 0.25 mM *eif2ak2* morpholino. Error bars represent SD (n = 4 independent experiments). Statistical analyses were performed using one-way ANOVA with Tukey’s post hoc test. (**E, F**) Effects of SB-MO on axonal length and branching of zebrafish expressing sense (G_4_C_2_)70 RNAs. Embryos were injected with 0.844 µM GFP mRNA, 0.844 µc sense (G_4_C_2_)70 RNAs, 0.844 µM sense (G_4_C_2_)70 RNAs plus 0.25 mM control morpholino or 0.844 µM sense (G_4_C_2_)70 RNAs plus 0.25 mM *eif2ak2* morpholino. Error bars represent SD (n = 4 independent experiments). Statistical analyses were performed using one-way ANOVA with Tukey’s post hoc test.

To validate our findings in an in vivo model, we performed a morpholino-mediated knockdown of the zebrafish ortholog of human PKR (i.e. Eif2ak2) in zebrafish expressing *C9ORF72* repeat expanded RNAs. We previously demonstrated that micro-injection of RNAs containing either sense (G_4_C_2_)70 or antisense (C_4_G_2_)70 repeats results in motor axonopathy including reduced axonal length and aberrant branching in zebrafish embryos (Swinnen et al., 2018). As no DPR proteins are detected, this phenotype is specifically caused by sense or antisense RNA themselves. We first designed a splice-blocking morpholino (SB-MO) targeting the exon 3–intron 3 junction of zebrafish *eif2ak2*. SB-MO caused the retention of intron 3 in a dose–response manner, resulting in a reduction of the WT *eif2ak2* transcripts (Figure 7A and Figure 7—figure supplement 1A). Co-injection of antisense (C_4_G_2_)70 RNAs with *eif2ak2* SB-MO significantly mitigates the abnormalities of axonal length and branching compared to zebrafish injected with (C_4_G_2_)70 RNAs and a control morpholino (Figure 7B–D). Consistent with this observation, Eif2ak2 reduction with a translation-blocking morpholino increases axonal length (Figure 7A and Figure 7—figure supplement 1B and C) and significantly ameliorates the abnormal branching phenotype caused by antisense (C_4_G_2_)70 RNAs. On the other hand, no protective effects were observed when co-injecting sense (G_4_C_2_)70 RNAs with *eif2ak2* SB-MO (Figure 7E and F). Overall, these data indicate that PKR is an important player contributing to toxicity induced by antisense C_4_G_2_ but not sense G_4_C_2_ repeat expanded RNAs.

**Figure 7—figure supplement 1.**
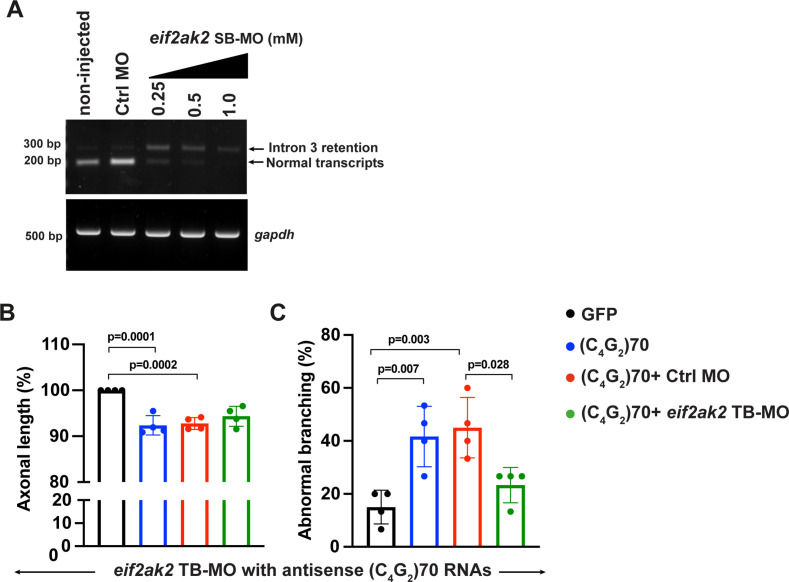
Translation-blocking morpholino of PKR mitigates antisense C_4_G_2_ repeat RNA-induced axonopathy in zebrafish. (**A**) Dose-dependent knockdown of WT transcripts and increase of transcripts with intron 3 retention as determined by RT-PCR analysis. *Eif2ak2* splice variants in non-injected, standard control and *eif2ak2* targeting MO-injected embryos 30 hpf are shown. (**B, C**) Effect of translation-blocking morpholino (TB-MO)-mediated reduction of Eif2ak2 on axonal length (**B**) and branching (**C**), using a dose of 0.25 mM for TB-MO and standard control MO in GFP and antisense (C_4_G_2_)70 RNAs expressing zebrafish. Error bars represent SD (n = 4 independent experiments). Statistical analyses were performed using one-way ANOVA with Tukey’s post hoc test. Figure 7—figure supplement 1—source data 1.Original agarose gel results for Figure 7—figure supplement 1A.

## Discussion

**Figure 8.**
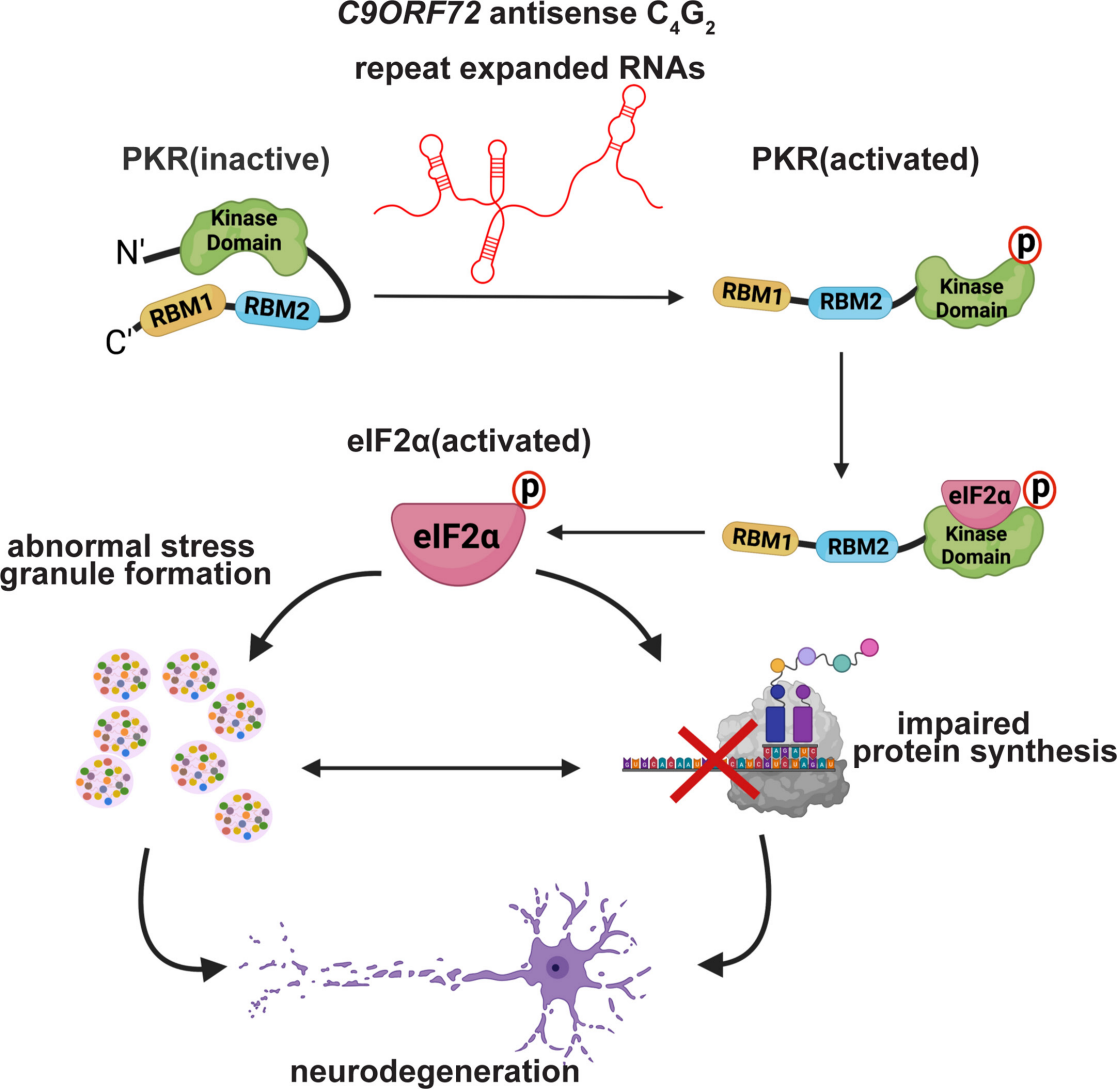
Proposed pathogenic mechanisms caused by *C9ORF72* antisense C_4_G_2_ repeat expanded RNAs. *C9ORF72* antisense C_4_G_2_ repeat expanded RNAs activate PKR/eIF2α-dependent integrated stress response and lead to neurotoxicity independent of DPR proteins.

It is generally accepted in the field that gain of toxicity from the bidirectionally transcribed repeat expanded RNAs plays important roles in FTD/ALS caused by *C9ORF72* repeat expansions (Jiang and Ravits, 2019). However, the relative contributions of potentially toxic species, including sense and antisense RNAs themselves, RNA foci, and DPR proteins, are largely debated. Our study shows for the first time that *C9ORF72* antisense C_4_G_2_ repeat expanded RNAs activate PKR/eIF2α-dependent integrated stress response and lead to neurotoxicity independent of DPR proteins in model systems (Figure 8). Although most C9FTD/ALS patients carry significantly longer G_4_G_2_ repeats and may produce antisense C_4_G_2_ repeat expanded RNAs of different sizes and expression levels, we also detected increased activation of PKR/eIF2α in patient frontal cortex. Consistent with our observations, increased phosphorylation of PKR has also been reported in BAC transgenic mice expressing 500 G_4_C_2_ repeats and in *C9ORF72* patients by two other studies (Rodriguez et al., 2021; Zu et al., 2020). Importantly, using strand-specific ASOs we showed the previously reported increased phosphorylation of PKR/eIF2α and stress granules when expressing G_4_C_2_ repeats are mainly driven by the antisense transcripts (Figures 6 and 7), highlighting the significance of antisense C_4_G_2_ repeat expanded RNAs in disease pathogenesis.

Several studies argue against the toxicity from either sense G_4_C_2_ or antisense C_4_G_2_ repeat expanded RNAs and associated RNA foci. In one study, *Drosophila* expressing intronic (G_4_C_2_)160 repeats show abundant sense RNA foci in the nucleus but have little DPR proteins and no neurodegeneration, suggesting that sense RNA foci is insufficient to cause toxicity in this model (Tran et al., 2015). Two other elegant studies generated *Drosophila* expressing interrupted G_4_C_2_ repeats by inserting stop codons every 12 repeats in all reading frames to prevent RAN translation. These *Drosophila* do not show any toxicity, whereas those expressing pure G_4_C_2_ repeats of similar sizes do despite comparable sense RNA foci accumulation. Similarly, *Drosophila* expressing interrupted antisense repeat expanded RNAs do not show any deficits, suggesting that antisense C_4_G_2_ RNAs are not toxic in this model system (Mizielinska et al., 2014; Moens et al., 2018). However, expressing the same antisense interrupted repeat construct (C_4_G_2_)108RO causes motor axonopathy in zebrafish (Swinnen et al., 2018). Consistent with this, we show that (C_4_G_2_)108RO is toxic to primary cortical neurons, which is partially rescued by the reduction of PKR (Figure 4G). The PKR-dependent toxicity is also observed in zebrafish expressing antisense (C_4_G_2_)70 RNAs (Figure 7B–D and Figure 7—figure supplement 1B and C). It is interesting to note that PKR, constitutively and ubiquitously expressed in vertebrate cells including zebrafish, is not found in plants, fungi, protists, or invertebrates such as *Drosophila* (Martinez et al., 2021). This may partially explain the difference in toxicities caused by the antisense C_4_G_2_ repeat expanded RNAs in *Drosophila*, zebrafish, and primary neurons.

The contribution of antisense repeat expanded RNAs to C9FTD/ALS pathogenesis is understudied, although overexpression of PR DPR proteins as those RAN translated from the antisense RNAs has been shown to be toxic in various model systems (Jovičić et al., 2015; Wen et al., 2014; Zhang et al., 2019). Aggregates of PR DPR proteins are rare in C9FTD/ALS postmortem tissues, whereas antisense RNA foci are as abundant as sense RNA foci in multiple CNS regions despite the scarcity of antisense RNA transcripts. One possibility of such discrepancy between RNA transcripts and foci levels is that antisense RNA foci are extraordinarily stable and rarely turn over once formed along the life span of patients. Several neuropathological studies have attempted to correlate the abundance and distribution of antisense RNA foci with C9FTD/ALS clinical features. Mizielinska et al., 2013 showed that patients with more antisense RNA foci tend to have an earlier age of symptom onset and, more intriguingly, antisense RNA foci are shown to be associated with nucleoli and mislocalization of TDP-43 in two different studies (Aladesuyi Arogundade et al., 2019; Cooper-Knock et al., 2015). These results highlight the disease relevance of antisense repeat expanded RNAs in C9FTD/ALS and the significance of our work. Our study, however, does not differentiate antisense RNA foci from RNAs themselves since it is technically challenging given that RNA foci inevitably form with the expression of repeat expanded RNAs. The proposed mechanisms of RNA foci-mediated toxicity are via sequestration of critical RBPs. Several RBPs have been proposed to interact with and/or are sequestered into sense RNA foci, yet those interacting with antisense RNA foci have not been well characterized and are worth exploring especially in correlation with PKR/eIF2α activation.

How is PKR specifically activated by *C9ORF72* antisense but not sense repeat expanded RNAs? PKR is a stress sensor first identified as a kinase responding to viral infections by directly binding to viral double-stranded RNAs (dsRNAs) (Martinez et al., 2021). Several disease-relevant repeats expanded RNAs, such as CUG and CGG, have been shown to form stable hairpins and directly bind to PKR, leading to its activation (Handa et al., 2003; Tian et al., 2000). It has been shown that antisense C_4_G_2_ DNAs form i-motifs consisting of two parallel duplexes in a head to tail orientation as well as protonated hairpins under near-physiological conditions, thus posing a possibility of direct activation of PKR by antisense RNA (Kovanda et al., 2015). In contrast, sense G_4_C_2_ RNAs exist in an equilibrium between two folded states, hairpin and G-quadruplex (Wang et al., 2019), and tend to form more stable unimolecular and multimolecular G-quadruplexes (Fratta et al., 2012; Reddy et al., 2013). Indeed, Rodriguez et al., 2021 showed dsRNAs, derived at least in part from G4C2 repeats, colocalize with phosphorylated TDP-43 in the cerebellum and frontal cortex of C9FTD/ALS patients. In addition to *C9ORF72* antisense repeat expanded RNAs, short and long interspersed retrotransposable elements (SINEs and LINEs) and endogenous retroviruses (ERVs) represent other main sources of endogenous dsRNAs (Sadeq et al., 2021). In *C9ORF72* patients, transcripts from multiple classes of repetitive elements are significantly elevated (Prudencio et al., 2017). Other PKR activators include cellular stresses such as oxidative stress, intracellular calcium increase or ER stress, as well as interferon-gamma (IFNγ), tumor necrosis factor α (TNFα), heparin, and platelet-derived growth factor (Martinez et al., 2021). Whether *C9ORF72* antisense C_4_G_2_ expanded RNAs activate PKR directly or indirectly via increased transcription of RNAs with repetitive elements or other PKR activators warrants additional studies.

Our study shows that *C9ORF72* antisense C_4_G_2_ expanded repeat RNAs promote robust global translation inhibition and stress granule formation independent of DPR proteins via the activation of the PKR/eIF2α pathway. Stress granules are dynamic structures that form and disperse rapidly with acute stress. However, chronic stress during aging or under pathological conditions leads to altered stress granule dynamics and persistent stress granules, which have been implicated in the aggregation of RBPs such as TDP-43 and contribute to the pathogenesis of FTD and ALS (Li et al., 2013). Previous studies have shown that expressing sense (G_4_C_2_) repeats can activate the PKR/eIF2a pathway (Zu et al., 2020) and induce stress granules (Green et al., 2017), which is replicated in our study (Figure 6). Interestingly, we detected abundant accumulation of both sense and antisense RNA foci in cells expressing sense (G_4_C_2_) expanded repeats. How such bidirectional transcription for *C9ORF72*-associated expanded repeats initiates is not known. Of note, we also detected sense foci accumulation in HEK293T cells expressing antisense (C_4_G_2_) expanded repeats. Nevertheless, using strand-specific ASOs to selectively degrade sense/antisense RNAs (Figure 6) or by directly injecting sense/antisense RNAs in zebrafish (Figure 7), our data clearly show that antisense C_4_G_2_, but not sense G_4_C_2_, repeat expanded RNAs activate the PKR/eIF2α pathway, leading to toxicity. It has been suggested the RAN translation of DPR proteins is increased explicitly by the integrated stress response via eIF2α phosphorylation (Cheng et al., 2018; Green et al., 2017) and several DPR proteins such as GR, PR, and GA are toxic (Boeynaems et al., 2016; Choi et al., 2019; Freibaum et al., 2015; Hao et al., 2019; Kanekura et al., 2016; Lee et al., 2016; May et al., 2014; Mizielinska et al., 2014; Schludi et al., 2017; Tao et al., 2015; Wen et al., 2014; Yamakawa et al., 2015; Yang et al., 2015; Zhang et al., 2018; Zhang et al., 2016; Zhang et al., 2019; Zhang et al., 2014; Zu et al., 2011). Thus, activation of PKR/eIF2α by *C9ORF72* antisense repeat expanded RNAs may lead to additional accumulation of DPR proteins and toxicity. Future studies will determine the relative contributions and interplay between repeat containing RNAs from either direction and DPRs proteins to disease pathogenesis.

From the therapeutic development point of view, several approaches have been explored to mitigate the gain of toxicity from *C9ORF72* repeat expanded RNAs (Jiang and Ravits, 2019). Our previous work with ASOs specifically targeting *C9ORF72* sense G_4_C_2_ repeat expanded RNAs showed great promise in a preclinical mouse model expressing 450 G_4_C_2_ repeats (Jiang et al., 2016). Unfortunately, there was a recent setback in a clinical trial using these ASOs to treat C9ALS patients (Mar 2022, news release by Biogen Inc). Although many confounding reasons may cause drug failures, further understanding of disease mechanisms is required to develop successful therapies for C9FTD/ALS. On this note, Zu et al. recently showed that metformin, an FDA-approved drug widely used for treating type 2 diabetes, inhibits PKR activation, reduces DPR proteins RAN translated from the sense strand, and improves behavioral and pathological deficits in BAC transgenic mice expressing G_4_C_2_ repeats (Zu et al., 2020). Our study now suggests that the activation of PKR in these BAC transgenic mice and in *C9ORF72* patients might result from the antisense repeat expanded RNAs. Future therapies targeting *C9ORF72* antisense RNAs and/or altered downstream molecular pathways hold great promise for these devastating neurodegenerative diseases.

## Materials and methods

### Plasmids and siRNAs

A construct containing 10 GGGGCC repeats, flanked 5′ by BbsI and 3′ by BsmBI recognition sites, was synthesized by GENEWIZ and used to generate antisense C_4_G_2_ repeats using recursive directional ligation as previously described (Mizielinska et al., 2014). The repeat-containing plasmids were amplified using recombination-deficient Stbl3 *Escherichia coli* (Life Technologies) at 32°C to minimize retraction of repeats. Human PKR cDNA was a gift from Dr. Thomas Dever (NIH, USA) and (C_4_G_2_)108RO was gifted by Dr. Adrian Isaacs (Moens et al., 2018). For longitudinal fluorescence microscopy, pGW1-mApple was used. All plasmids were verified by Sanger sequencing (Genewiz, USA). All MOE-Gapmer ASOs were synthesized by Integrated DNA Technologies, USA. The ASO sequences are sense ASO (GCCCCTAGCGCGCGACTC) (Jiang et al., 2016), AS ASO1 (GGCCGGGGCCGGGGCCGGGG), and AS ASO2 (CGGGGCCG GGGCCGGGGC). siRNAs against PKR and control siRNAs were purchased from Horizon Discovery, USA.

### Human tissues

Postmortem brain tissues from C9FTD/ALS patients (n = 6) and controls (n = 6) were obtained from the Emory Neuropathology Core. Patient information is provided in Table 1.

### Cell culture, transfection, and treatment

Human embryonic kidney (HEK293T) and human neuroblastoma (SH-SY5Y) cells from ATCC were cultured in high-glucose DMEM (Invitrogen) and DMEM-F12 (Invitrogen), respectively, supplemented with 10% fetal bovine serum (Corning), 4 mM GlutaMAX (Invitrogen), penicillin (100 U/mL), streptomycin (100 μg/mL), and non-essential amino acids (1%). All parental cell lines were obtained from ATCC (HEK293T, CRL-3216; SH-SY5Y, CRL-2266) and are mycoplasma negative (abcam ab289834). Cells were grown at 37°C in a humidified atmosphere with 5% CO_2_. Cells were transiently transfected using polyethyleneimine or Lipofectamine 2000. Experiments were performed 24 or 48 hr after transfection. For puromycin labeling, HEK293T and primary neurons were incubated with puromycin (10 ug/mL) for 30 and 5 min, respectively. For PKR inhibition, cells were treated with PKR inhibitor (C16) at 5 nM for 12 hr followed by transfection with repeats RNAs.

### Primary cortical neuronal culture and transfection

Primary cortical neurons were prepared from C57BL/6J mouse embryos (Charles River) of either sex on embryonic day 17. Cerebral cortices were dissected and enzymatically dissociated using trypsin with EDTA (Thermo Fisher Scientific; 10 min), mechanically dissociated in Minimum Essential Media (MEM; Fisher) supplemented with 0.6% glucose (Sigma) and 10% FBS (Hyclone) and stained to assess viability using Trypan Blue (Sigma). Neurons were plated on coverslips (Matsunami Inc, 22 mm). A total of 50,000 neurons were plated as a ‘spot’ on the center of the coverslip to create a small, high-density network. Neurons were cultured in standard growth medium (glial conditioned neurobasal plus medium [Fisher] supplemented with GlutaMAX [Gibco], and B27 plus [Invitrogen]), and half of the media was exchanged 2–3 times a week until the experiment endpoints. No antibiotics or antimycotics were used. Cultures were maintained in an incubator regulated at 37^ο^C, 5% CO_2_, and 95% relative humidity as described (Valdez-Sinon et al., 2020). Cells were transiently transfected using Lipofectamine 2000 (Invitrogen) according to the manufacturer’s instructions.

### Longitudinal fluorescence microscopy

Mouse primary cortical neurons were transfected with mApple and repeat expanded constructs and imaged by fluorescence microscopy at 24 hr intervals for 7–10 d as described (Weskamp et al., 2019). Time of death was determined based on rounding of the soma, retraction of neurites, or loss of fluorescence. The time of death for individual neurons was used to calculate the risk of death in each population relative to a reference group. Images were acquired using Keyence BZ-X810 microscope with a ×10 objective and analyzed using ImageJ. The images were stitched and stacked, and cell death was scored using the criteria mentioned above.

### RNA fluorescence in situ hybridization

LNA DNA probes were used against the sense and antisense hexanucleotide repeat expanded RNAs (Exiqon, Inc). The probe sequence for detecting sense RNA foci: TYE563-CCCCGGCCCCGGCCCC; and that for antisense RNA foci is: TYE563-GGGGCCGGGGCCGGGG. All hybridization steps were performed under RNase-free conditions. Cells were fixed in 4% paraformaldehyde (Electron Microscopy Sciences) for 20 min, washed three times for 5 min with phosphate buffered saline (DEPC 1× PBS, Corning) followed by permeabilization with 0.2% Triton-X 100 (Sigma) for 10 min and then incubated with 2× SSC buffer for 10 min. Cells were hybridized (50% formamide, 2× SCC, 50 mM sodium phosphate [pH 7], 10% dextran sulfate, and 2 mM vanadyl sulfate ribonucleosides) with denatured probes (final concentration of 40 nM) at 66°C for 2 hr. After hybridization, slides were washed at room temperature in 0.1% Tween-20/2× SCC for 10 min twice and in stringency washes in 0.1× SCC at 65°C for 10 min. Cell nuclei were stained with DAPI. Three to six random pictures were taken by Keyence BZ-X810 microscope with a ×60 oil objective and analyzed using ImageJ.

### Immunofluorescence

Cells were fixed in 4% paraformaldehyde (Electron Microscopy Sciences) for 20 min, washed three times for 5 min with phosphate buffered saline (1×PBS, Corning), and treated with 0.2% Triton-X 100 (Sigma) in PBS for 10 min. Cells were blocked for 30 min in a blocking solution consisting of 4% bovine serum albumin (Sigma) in PBS. Cells were incubated overnight in primary antibodies diluted in blocking solution. The next day, cells were washed three times for 5 min in PBS and incubated in secondary antibodies in blocking solution for 1 hr at room temperature (dark). After washing three times for 5 min, coverslips with the cells were mounted using Prolong Gold Antifade mounting media (Invitrogen). Images were acquired with Keyence BZ-X810 microscope with a ×60 oil objective and analyzed using ImageJ.

### Immunohistochemistry

Postmortem brain tissues were obtained from the brain bank maintained by the Emory Alzheimer Disease Research Center under proper Institutional Review Board protocols. Paraffin-embedded sections from frontal cortex (8 μm thickness) were deparaffinized by incubation at 60°C for 30 min and rehydrated by immersion in graded ethanol solutions. Antigen retrieval was done by microwaving in a 10 mM citrate buffer (pH 6.0) for 5 min followed by allowing slides to cool to room temperature for 30 min. Endogenous peroxidase activity was eliminated by incubating slides with hydrogen peroxide block solution (Fisher) for 10 min at room temperature followed by rinsing in phosphate buffered saline. Nonspecific binding was reduced by blocking in ultra-Vision Block (Fisher) for 5 min at room temperature. Sections were then incubated overnight with primary antibodies diluted in 1% BSA in phosphate buffered saline for 30 min at room temperature or incubated without primary antibody as a negative control. Sections were rinsed in phosphate buffered saline and incubated in labeled ultra-Vision LP detection system horseradish peroxidase-polymer secondary antibody (Fisher) for 15 min at room temperature. Slides were imaged for analysis using an Aperio Digital Pathology Slide Scanner (Leica Biosystems). For IHC, rabbit anti-p-PKR, Millipore 07-532 (1:100 dilution) antibody was used.

### Protein lysate preparation

Whole cell/tissue extracts were lysed using RIPA Lysis Buffer pH 7.4 (Bio-world, USA) supplemented with Halt protease and phosphatase inhibitor cocktail (Thermo Fisher Scientific). Lysates were sonicated at 25% amplitude for three cycles for 15 s with 5 s intervals. Supernatant was collected after centrifuging at max speed for 15 min at 4°C. The concentration of the isolated proteins was determined using BCA Protein Assay Reagent (Pierce, USA).

### Immunoblotting assay

For western blotting, 20–30 μg of proteins were prepared in 4× Laemmli sample buffer and heat-denatured at 95°C for 5 min. Samples were resolved on 4–20% gradient gels (Bio-Rad). Proteins were transferred to nitrocellulose membranes (0.2 μm, Bio-Rad). The membrane was blocked in 5% milk and incubated overnight at 4°C with primary antibodies diluted in blocking buffer. Secondary antibodies HRP-conjugated secondary antibodies (ABclonal) or IRDye secondary antibodies (LI-COR) were diluted in blocking buffer and applied to the membrane for 1 hr at room temperature. Primary antibodies used mouse anti-FLAG (1:1000; Sigma # F1804), rabbit anti-HA (1:1000; Cell Signaling # 3724S), mouse anti-MYC (1:1000; Sigma # C3956), rabbit anti-PKR (1:1000; abcam # ab184257), rabbit anti-phospho-PKR (1:1000; abcam # ab32036), rabbit anti-eIF2α (1:1000; Cell Signaling # 3398S), rabbit anti-phospho-eIF2α (1:1000; CST# 9722S), rabbit anti-PERK (1:1000; CST # 3192S), rabbit anti-phospho-PERK (1:1000; abcam # ab192591), and rabbit anti-GAPDH (1:5000; ABclonal # ac001). Antibodies against PR, GP, and PA have been previously reported (Jiang et al., 2016). Super Signal West Pico (Pierce, USA) was used for detection of peroxidase activity. Molecular masses were determined by comparison to protein standards (Thermo Scientific). The immunoreactive bands were detected by ChemiDoc Image System (Bio-Rad, USA).

### Quantitative real-time PCR

Total RNAs were extracted using a RNeasy kit as instructed by the manufacturer (QIAGEN). cDNA was prepared using High-Capacity cDNA Reverse Transcription Kit from applied biosystem. Quantitative RT-PCR reactions were conducted and analyzed on a StepOnePlus Real-Time PCR system (Applied Biosystems). Gene expression levels were measured by SYBR green (Thermo Fisher Scientific) quantitative real-time PCR (PRIMER SEQUENCE).

### Zebrafish microinjections, SV2 immunohistochemistry, and phenotyping

Zebrafish work was performed as previously described (Swinnen et al., 2018). Zebrafish oocytes were injected at the one- to two-cell stage with the indicated amounts of morpholinos. The splice-blocking morpholino targeting exon 3 intron 3 junction of *Danio rerio eif2ak2* (transcript ENSDART00000164338.2; morpholino sequence 5′-AATGTCTTGAATACTGACC GGGTGA-3′), the translation-blocking morpholino targeting AUG start codon (morpholino sequence 5′-TTCCTGACAGAGACTCCATTGCGAA-3′), and the standard control oligo (morpholino sequence 5′-CCTCTTACCTCAGTTACAATTTATA-3′) were designed and generated by Gene Tools (Philomath, USA). Injected oocytes were incubated at 28°C. After 24 hr post fertilization (hpf), the embryos were dechorionated using a forceps. Only morphologically normal embryos were selected for downstream experiments. At 30 hpf, the selected fish were deyolked and subsequently fixed overnight at 4°C in 4% formaldehyde in 1×PBS. Fish were permeabilized with acetone for 1 hr at − 20°C, blocked with 1% BSA/1% DMSO/PBS for 1 hr at room temperature, and immunostained with mouse anti-SV2 primary antibody (AB2315387, Developmental Studies Hybridoma Bank) followed by a secondary antibody.

For phenotyping, 15 embryos per condition were analyzed with imaging (Leica DM 3000 LED microscope; DMK 33UX250 USB3.0 monochrome industrial camera, The Imaging Source, Bremen, Germany) and the Lucia software (version 4.60, Laboratory Imaging, Prague, Czech Republic) by a blinded observer. For the axonal length, a standardized method was used; five predefined and consecutive motor axons (i.e. the 8th up to the 12th axon on one side) were measured in all 15 embryos. Data for axonal length were normalized to the control condition. For the abnormal branching, a predefined set of 20 consecutive motor axons (i.e. the 8th up to the 17th axon on both sides) in the same 15 embryos were analyzed. Motor axons were considered abnormal when axons branched at or before the ventral edge of the notochord. An embryo was considered as having ‘abnormal branching’ when at least two of these 20 axons were abnormal. For each experiment, the standard morpholino was used as control at the same dose of the tested morpholino.

### Statistical analysis

Statistical analyses and graphs were prepared in GraphPad Prism (version 9). Data is expressed as mean ± SD. Student’s *t*-test or one-way ANOVA was used for statistical analysis unless specified in figure legends.

## Data Availability

All data generated or analysed during this study are included in the manuscript.

## Additional files

MDAR checklist

## References

[bib1] Aladesuyi Arogundade O, Stauffer JE, Saberi S, Diaz-Garcia S, Malik S, Basilim H, Rodriguez MJ, Ohkubo T, Ravits J (2019). Antisense RNA foci are associated with nucleoli and TDP-43 mislocalization in c9orf72-ALS/FTD: a quantitative study. Acta Neuropathologica.

[bib2] Ash PEA, Bieniek KF, Gendron TF, Caulfield T, Lin W-L, DeJesus-Hernandez M, van Blitterswijk MM, Jansen-West K, Paul JW, Rademakers R, Boylan KB, Dickson DW, Petrucelli L (2013). Unconventional translation of C9orf72 GGGGCC expansion generates insoluble polypeptides specific to c9FTD/ALS. Neuron.

[bib3] Atanasio A, Decman V, White D, Ramos M, Ikiz B, Lee H-C, Siao C-J, Brydges S, LaRosa E, Bai Y, Fury W, Burfeind P, Zamfirova R, Warshaw G, Orengo J, Oyejide A, Fralish M, Auerbach W, Poueymirou W, Freudenberg J, Gong G, Zambrowicz B, Valenzuela D, Yancopoulos G, Murphy A, Thurston G, Lai K-MV (2016). C9Orf72 ablation causes immune dysregulation characterized by leukocyte expansion, autoantibody production and glomerulonephropathy in mice. Scientific Reports.

[bib4] Boeynaems S, Bogaert E, Michiels E, Gijselinck I, Sieben A, Jovičić A, De Baets G, Scheveneels W, Steyaert J, Cuijt I, Verstrepen KJ, Callaerts P, Rousseau F, Schymkowitz J, Cruts M, Van Broeckhoven C, Van Damme P, Gitler AD, Robberecht W, Van Den Bosch L (2016). *Drosophila* screen connects nuclear transport genes to Dpr pathology in c9als/FTD. Scientific Reports.

[bib5] Boivin M, Pfister V, Gaucherot A, Ruffenach F, Negroni L, Sellier C, Charlet-Berguerand N (2020). Reduced autophagy upon C9orf72 loss synergizes with dipeptide repeat protein toxicity in G4C2 repeat expansion disorders. The EMBO Journal.

[bib6] Braems E, Swinnen B, Van Den Bosch L (2020). C9Orf72 loss-of-function: a trivial, stand-alone or additive mechanism in C9 ALS/FTD?. Acta Neuropathologica.

[bib7] Braems E, Bercier V, Van Schoor E, Heeren K, Beckers J, Fumagalli L, Dedeene L, Moisse M, Geudens I, Hersmus N, Mehta AR, Selvaraj BT, Chandran S, Ho R, Thal DR, Van Damme P, Swinnen B, Van Den Bosch L (2022). Hnrnpk alleviates RNA toxicity by counteracting DNA damage in C9orf72 ALS. Acta Neuropathologica.

[bib8] Burberry A, Suzuki N, Wang JY, Moccia R, Mordes DA, Stewart MH, Suzuki-Uematsu S, Ghosh S, Singh A, Merkle FT, Koszka K, Li QZ, Zon L, Rossi DJ, Trowbridge JJ, Notarangelo LD, Eggan K (2016). Loss-Of-Function mutations in the C9orf72 mouse ortholog cause fatal autoimmune disease. Science Translational Medicine.

[bib9] Celona B, Crinò C, Giudice E, Pietro SD (2017). Evaluation of pericardial effusion in dogs and successful treatment using a hemodialysis fistula needle: a retrospective study. Topics in Companion Animal Medicine.

[bib10] Cheng W, Wang S, Mestre AA, Fu C, Makarem A, Xian F, Hayes LR, Lopez-Gonzalez R, Drenner K, Jiang J, Cleveland DW, Sun S (2018). C9Orf72 GGGGCC repeat-associated non-AUG translation is upregulated by stress through eIF2α phosphorylation. Nature Communications.

[bib11] Chew J, Gendron TF, Prudencio M, Sasaguri H, Zhang YJ, Castanedes-Casey M, Lee CW, Jansen-West K, Kurti A, Murray ME, Bieniek KF, Bauer PO, Whitelaw EC, Rousseau L, Stankowski JN, Stetler C, Daughrity LM, Perkerson EA, Desaro P, Johnston A, Overstreet K, Edbauer D, Rademakers R, Boylan KB, Dickson DW, Fryer JD, Petrucelli L (2015). Neurodegeneration. C9orf72 repeat expansions in mice cause TDP-43 pathology, neuronal loss, and behavioral deficits. Science.

[bib12] Choi SY, Lopez-Gonzalez R, Krishnan G, Phillips HL, Li AN, Seeley WW, Yao WD, Almeida S, Gao FB (2019). C9ORF72-ALS/FTD-associated poly (GR) binds atp5a1 and compromises mitochondrial function in vivo. Nature Neuroscience.

[bib13] Conlon EG, Lu L, Sharma A, Yamazaki T, Tang T, Shneider NA, Manley JL (2016). The C9orf72 GGGGCC expansion forms RNA G-quadruplex inclusions and sequesters hnRNP H to disrupt splicing in ALS brains. eLife.

[bib14] Cooper-Knock J, Higginbottom A, Stopford MJ, Highley JR, Ince PG, Wharton SB, Pickering-Brown S, Kirby J, Hautbergue GM, Shaw PJ (2015). Antisense RNA foci in the motor neurons of C9ORF72-ALS patients are associated with TDP-43 proteinopathy. Acta Neuropathologica.

[bib15] DeJesus-Hernandez M, Mackenzie IR, Boeve BF, Boxer AL, Baker M, Rutherford NJ, Nicholson AM, Finch NA, Flynn H, Adamson J, Kouri N, Wojtas A, Sengdy P, Hsiung GYR, Karydas A, Seeley WW, Josephs KA, Coppola G, Geschwind DH, Wszolek ZK, Feldman H, Knopman DS, Petersen RC, Miller BL, Dickson DW, Boylan KB, Graff-Radford NR, Rademakers R (2011). Expanded GGGGCC hexanucleotide repeat in noncoding region of C9orf72 causes chromosome 9p-linked FTD and ALS. Neuron.

[bib16] DeJesus-Hernandez M, Finch NA, Wang X, Gendron TF, Bieniek KF, Heckman MG, Vasilevich A, Murray ME, Rousseau L, Weesner R, Lucido A, Parsons M, Chew J, Josephs KA, Parisi JE, Knopman DS, Petersen RC, Boeve BF, Graff-Radford NR, de Boer J, Asmann YW, Petrucelli L, Boylan KB, Dickson DW, van Blitterswijk M, Rademakers R (2017). In-Depth clinico-pathological examination of RNA foci in a large cohort of C9orf72 expansion carriers. Acta Neuropathologica.

[bib17] Donnelly CJ, Zhang PW, Pham JT, Haeusler AR, Mistry NA, Vidensky S, Daley EL, Poth EM, Hoover B, Fines DM, Maragakis N, Tienari PJ, Petrucelli L, Traynor BJ, Wang J, Rigo F, Bennett CF, Blackshaw S, Sattler R, Rothstein JD (2013). Rna toxicity from the ALS/FTD C9orf72 expansion is mitigated by antisense intervention. Neuron.

[bib18] Fratta P, Mizielinska S, Nicoll AJ, Zloh M, Fisher EMC, Parkinson G, Isaacs AM (2012). C9Orf72 hexanucleotide repeat associated with amyotrophic lateral sclerosis and frontotemporal dementia forms RNA G-quadruplexes. Scientific Reports.

[bib19] Freibaum BD, Lu Y, Lopez-Gonzalez R, Kim NC, Almeida S, Lee KH, Badders N, Valentine M, Miller BL, Wong PC, Petrucelli L, Kim HJ, Gao FB, Taylor JP (2015). Ggggcc repeat expansion in C9orf72 compromises nucleocytoplasmic transport. Nature.

[bib20] Gao FB, Richter JD, Cleveland DW (2017). Rethinking unconventional translation in neurodegeneration. Cell.

[bib21] Gijselinck I, Van Langenhove T, van der Zee J, Sleegers K, Philtjens S, Kleinberger G, Janssens J, Bettens K, Van Cauwenberghe C, Pereson S, Engelborghs S, Sieben A, De Jonghe P, Vandenberghe R, Santens P, De Bleecker J, Maes G, Bäumer V, Dillen L, Joris G, Cuijt I, Corsmit E, Elinck E, Van Dongen J, Vermeulen S, Van den Broeck M, Vaerenberg C, Mattheijssens M, Peeters K, Robberecht W, Cras P, Martin J-J, De Deyn PP, Cruts M, Van Broeckhoven C (2012). A C9orf72 promoter repeat expansion in a flanders-belgian cohort with disorders of the frontotemporal lobar degeneration-amyotrophic lateral sclerosis spectrum: a gene identification study. The Lancet. Neurology.

[bib22] Green KM, Glineburg MR, Kearse MG, Flores BN, Linsalata AE, Fedak SJ, Goldstrohm AC, Barmada SJ, Todd PK (2017). Ran translation at c9orf72-associated repeat expansions is selectively enhanced by the integrated stress response. Nature Communications.

[bib23] Haeusler AR, Donnelly CJ, Periz G, Simko EAJ, Shaw PG, Kim M-S, Maragakis NJ, Troncoso JC, Pandey A, Sattler R, Rothstein JD, Wang J (2014). C9Orf72 nucleotide repeat structures initiate molecular cascades of disease. Nature.

[bib24] Handa V, Saha T, Usdin K (2003). The fragile X syndrome repeats form RNA hairpins that do not activate the interferon-inducible protein kinase, PKR, but are cut by Dicer. Nucleic Acids Research.

[bib25] Hao Z, Liu L, Tao Z, Wang R, Ren H, Sun H, Lin Z, Zhang Z, Mu C, Zhou J, Wang G (2019). Motor dysfunction and neurodegeneration in a C9orf72 mouse line expressing poly-pr. Nature Communications.

[bib26] Harms MB, Cady J, Zaidman C, Cooper P, Bali T, Allred P, Cruchaga C, Baughn M, Libby RT, Pestronk A, Goate A, Ravits J, Baloh RH (2013). Lack of C9orf72 coding mutations supports a gain of function for repeat expansions in amyotrophic lateral sclerosis. Neurobiology of Aging.

[bib27] He F, Flores BN, Krans A, Frazer M, Natla S, Niraula S, Adefioye O, Barmada SJ, Todd PK (2020). The carboxyl termini of Ran translated GGGGCC nucleotide repeat expansions modulate toxicity in models of ALS/FTD. Acta Neuropathologica Communications.

[bib28] Jiang J, Zhu Q, Gendron TF, Saberi S, McAlonis-Downes M, Seelman A, Stauffer JE, Jafar-Nejad P, Drenner K, Schulte D, Chun S, Sun S, Ling S-C, Myers B, Engelhardt J, Katz M, Baughn M, Platoshyn O, Marsala M, Watt A, Heyser CJ, Ard MC, De Muynck L, Daughrity LM, Swing DA, Tessarollo L, Jung CJ, Delpoux A, Utzschneider DT, Hedrick SM, de Jong PJ, Edbauer D, Van Damme P, Petrucelli L, Shaw CE, Bennett CF, Da Cruz S, Ravits J, Rigo F, Cleveland DW, Lagier-Tourenne C (2016). Gain of toxicity from ALS/FTD-linked repeat expansions in C9orf72 is alleviated by antisense oligonucleotides targeting GGGGCC-containing RNAs. Neuron.

[bib29] Jiang J, Ravits J (2019). Pathogenic mechanisms and therapy development for C9orf72 amyotrophic lateral sclerosis/frontotemporal dementia. Neurotherapeutics.

[bib30] Jovičić A, Mertens J, Boeynaems S, Bogaert E, Chai N, Yamada SB, Paul JW, Sun S, Herdy JR, Bieri G, Kramer NJ, Gage FH, Van Den Bosch L, Robberecht W, Gitler AD (2015). Modifiers of C9orf72 dipeptide repeat toxicity connect nucleocytoplasmic transport defects to FTD/ALS. Nature Neuroscience.

[bib31] Kanekura K, Yagi T, Cammack AJ, Mahadevan J, Kuroda M, Harms MB, Miller TM, Urano F (2016). Poly-dipeptides encoded by the C9orf72 repeats block global protein translation. Human Molecular Genetics.

[bib32] Koppers M, Blokhuis AM, Westeneng H-J, Terpstra ML, Zundel CAC, Vieira de Sá R, Schellevis RD, Waite AJ, Blake DJ, Veldink JH, van den Berg LH, Pasterkamp RJ (2015). C9Orf72 ablation in mice does not cause motor neuron degeneration or motor deficits. Annals of Neurology.

[bib33] Kovanda A, Zalar M, Šket P, Plavec J, Rogelj B (2015). Anti-Sense DNA D (GGCCCC) N expansions in C9orf72 form i-motifs and protonated hairpins. Scientific Reports.

[bib34] La Spada AR, Taylor JP (2010). Repeat expansion disease: progress and puzzles in disease pathogenesis. Nature Reviews. Genetics.

[bib35] Lee YB, Chen HJ, Peres JN, Gomez-Deza J, Attig J, Stalekar M, Troakes C, Nishimura AL, Scotter EL, Vance C, Adachi Y, Sardone V, Miller JW, Smith BN, Gallo JM, Ule J, Hirth F, Rogelj B, Houart C, Shaw CE (2013). Hexanucleotide repeats in ALS/FTD form length-dependent RNA foci, sequester RNA binding proteins, and are neurotoxic. Cell Reports.

[bib36] Lee KH, Zhang P, Kim HJ, Mitrea DM, Sarkar M, Freibaum BD, Cika J, Coughlin M, Messing J, Molliex A, Maxwell BA, Kim NC, Temirov J, Moore J, Kolaitis RM, Shaw TI, Bai B, Peng J, Kriwacki RW, Taylor JP (2016). C9Orf72 dipeptide repeats impair the assembly, dynamics, and function of membrane-less organelles. Cell.

[bib37] Li YR, King OD, Shorter J, Gitler AD (2013). Stress granules as crucibles of ALS pathogenesis. The Journal of Cell Biology.

[bib38] Martinez NW, Gómez FE, Matus S (2021). The potential role of protein kinase R as a regulator of age-related neurodegeneration. Frontiers in Aging Neuroscience.

[bib39] May S, Hornburg D, Schludi MH, Arzberger T, Rentzsch K, Schwenk BM, Grässer FA, Mori K, Kremmer E, Banzhaf-Strathmann J, Mann M, Meissner F, Edbauer D (2014). C9Orf72 FTLD/ALS-associated Gly-Ala dipeptide repeat proteins cause neuronal toxicity and UNC119 sequestration. Acta Neuropathologica.

[bib40] McEachin ZT, Gendron TF, Raj N, García-Murias M, Banerjee A, Purcell RH, Ward PJ, Todd TW, Merritt-Garza ME, Jansen-West K, Hales CM, García-Sobrino T, Quintáns B, Holler CJ, Taylor G, San Millán B, Teijeira S, Yamashita T, Ohkubo R, Boulis NM, Xu C, Wen Z, Streichenberger N, Fogel BL, Kukar T, Abe K, Dickson DW, Arias M, Glass JD, Jiang J, Tansey MG, Sobrido M-J, Petrucelli L, Rossoll W, Bassell GJ, Neuro–CEB Neuropathology Network (2020a). Chimeric peptide species contribute to divergent dipeptide repeat pathology in c9als/FTD and SCA36. Neuron.

[bib41] McEachin ZT, Parameswaran J, Raj N, Bassell GJ, Jiang J (2020b). Rna-Mediated toxicity in C9orf72 ALS and FTD. Neurobiology of Disease.

[bib42] Mizielinska S, Lashley T, Norona FE, Clayton EL, Ridler CE, Fratta P, Isaacs AM (2013). C9Orf72 frontotemporal lobar degeneration is characterised by frequent neuronal sense and antisense RNA foci. Acta Neuropathologica.

[bib43] Mizielinska S, Grönke S, Niccoli T, Ridler CE, Clayton EL, Devoy A, Moens T, Norona FE, Woollacott IOC, Pietrzyk J, Cleverley K, Nicoll AJ, Pickering-Brown S, Dols J, Cabecinha M, Hendrich O, Fratta P, Fisher EMC, Partridge L, Isaacs AM (2014). C9Orf72 repeat expansions cause neurodegeneration in *Drosophila* through arginine-rich proteins. Science.

[bib44] Moens TG, Mizielinska S, Niccoli T, Mitchell JS, Thoeng A, Ridler CE, Grönke S, Esser J, Heslegrave A, Zetterberg H, Partridge L, Isaacs AM (2018). Sense and antisense RNA are not toxic in *Drosophila* models of c9orf72-associated ALS/FTD. Acta Neuropathologica.

[bib45] Mori K, Lammich S, Mackenzie IRA, Forné I, Zilow S, Kretzschmar H, Edbauer D, Janssens J, Kleinberger G, Cruts M, Herms J, Neumann M, Van Broeckhoven C, Arzberger T, Haass C (2013). Hnrnp A3 binds to GGGGCC repeats and is a constituent of p62-positive/TDP43-negative inclusions in the hippocampus of patients with C9orf72 mutations. Acta Neuropathologica.

[bib46] O’Rourke JG, Sahabian A, Wichterle H, Baloh RH, Sareen D, Svendsen CN (2016). Als disrupts spinal motor neuron maturation and aging pathways within gene co-expression networks. Nature Neuroscience.

[bib47] Prudencio M, Gonzales PK, Cook CN, Gendron TF, Daughrity LM, Song Y, Ebbert MTW, van Blitterswijk M, Zhang YJ, Jansen-West K, Baker MC, DeTure M, Rademakers R, Boylan KB, Dickson DW, Petrucelli L, Link CD (2017). Repetitive element transcripts are elevated in the brain of C9orf72 ALS/FTLD patients. Human Molecular Genetics.

[bib48] Reddy K, Zamiri B, Stanley SYR, Macgregor RB, Pearson CE (2013). The disease-associated R (GGGGCC) repeat from the C9orf72 gene forms tract length-dependent uni- and multimolecular RNA G-quadruplex structures. Journal of Biological Chemistry.

[bib49] Renton AE, Majounie E, Waite A, Simón-Sánchez J, Rollinson S, Gibbs JR, Schymick JC, Laaksovirta H, van Swieten JC, Myllykangas L, Kalimo H, Paetau A, Abramzon Y, Remes AM, Kaganovich A, Scholz SW, Duckworth J, Ding J, Harmer DW, Hernandez DG, Johnson JO, Mok K, Ryten M, Trabzuni D, Guerreiro RJ, Orrell RW, Neal J, Murray A, Pearson J, Jansen IE, Sondervan D, Seelaar H, Blake D, Young K, Halliwell N, Callister JB, Toulson G, Richardson A, Gerhard A, Snowden J, Mann D, Neary D, Nalls MA, Peuralinna T, Jansson L, Isoviita V-M, Kaivorinne A-L, Hölttä-Vuori M, Ikonen E, Sulkava R, Benatar M, Wuu J, Chiò A, Restagno G, Borghero G, Sabatelli M, Heckerman D, Rogaeva E, Zinman L, Rothstein JD, Sendtner M, Drepper C, Eichler EE, Alkan C, Abdullaev Z, Pack SD, Dutra A, Pak E, Hardy J, Singleton A, Williams NM, Heutink P, Pickering-Brown S, Morris HR, Tienari PJ, Traynor BJ, ITALSGEN Consortium (2011). A hexanucleotide repeat expansion in C9orf72 is the cause of chromosome 9p21-linked ALS-FTD. Neuron.

[bib50] Rodriguez S, Sahin A, Schrank BR, Al-Lawati H, Costantino I, Benz E, Fard D, Albers AD, Cao L, Gomez AC, Evans K, Ratti E, Cudkowicz M, Frosch MP, Talkowski M, Sorger PK, Hyman BT, Albers MW (2021). Genome-encoded cytoplasmic double-stranded RNAs, found in C9orf72 ALS-FTD brain, propagate neuronal loss. Science Translational Medicine.

[bib51] Sadeq S, Al-Hashimi S, Cusack CM, Werner A (2021). Endogenous double-stranded RNA. Non-Coding RNA.

[bib52] Sareen D, O’Rourke JG, Meera P, Muhammad AKMG, Grant S, Simpkinson M, Bell S, Carmona S, Ornelas L, Sahabian A, Gendron T, Petrucelli L, Baughn M, Ravits J, Harms MB, Rigo F, Bennett CF, Otis TS, Svendsen CN, Baloh RH (2013). Targeting RNA foci in iPSC-derived motor neurons from ALS patients with a C9orf72 repeat expansion. Science Translational Medicine.

[bib53] Schludi MH, Becker L, Garrett L, Gendron TF, Zhou Q, Schreiber F, Popper B, Dimou L, Strom TM, Winkelmann J, von Thaden A, Rentzsch K, May S, Michaelsen M, Schwenk BM, Tan J, Schoser B, Dieterich M, Petrucelli L, Hölter SM, Wurst W, Fuchs H, Gailus-Durner V, de Angelis MH, Klopstock T, Arzberger T, Edbauer D (2017). Spinal poly-GA inclusions in a C9orf72 mouse model trigger motor deficits and inflammation without neuron loss. Acta Neuropathologica.

[bib54] Schmidt EK, Clavarino G, Ceppi M, Pierre P (2009). Sunset, a nonradioactive method to monitor protein synthesis. Nature Methods.

[bib55] Sellier C, Buijsen RAM, He F, Natla S, Jung L, Tropel P, Gaucherot A, Jacobs H, Meziane H, Vincent A, Champy M-F, Sorg T, Pavlovic G, Wattenhofer-Donze M, Birling M-C, Oulad-Abdelghani M, Eberling P, Ruffenach F, Joint M, Anheim M, Martinez-Cerdeno V, Tassone F, Willemsen R, Hukema RK, Viville S, Martinat C, Todd PK, Charlet-Berguerand N (2017). Translation of expanded CGG repeats into fmrpolyg is pathogenic and may contribute to fragile X tremor ataxia syndrome. Neuron.

[bib56] Sudria-Lopez E, Koppers M, de Wit M, van der Meer C, Westeneng H-J, Zundel CAC, Youssef SA, Harkema L, de Bruin A, Veldink JH, van den Berg LH, Pasterkamp RJ (2016). Full ablation of C9orf72 in mice causes immune system-related pathology and neoplastic events but no motor neuron defects. Acta Neuropathologica.

[bib57] Sullivan PM, Zhou X, Robins AM, Paushter DH, Kim D, Smolka MB, Hu F (2016). The ALS/FTLD associated protein C9orf72 associates with SMCR8 and WDR41 to regulate the autophagy-lysosome pathway. Acta Neuropathologica Communications.

[bib58] Swinnen B, Bento-Abreu A, Gendron TF, Boeynaems S, Bogaert E, Nuyts R, Timmers M, Scheveneels W, Hersmus N, Wang J, Mizielinska S, Isaacs AM, Petrucelli L, Lemmens R, Van Damme P, Van Den Bosch L, Robberecht W (2018). A zebrafish model for C9orf72 ALS reveals RNA toxicity as a pathogenic mechanism. Acta Neuropathologica.

[bib59] Tabet R, Schaeffer L, Freyermuth F, Jambeau M, Workman M, Lee C-Z, Lin C-C, Jiang J, Jansen-West K, Abou-Hamdan H, Désaubry L, Gendron T, Petrucelli L, Martin F, Lagier-Tourenne C (2018). Cug initiation and frameshifting enable production of dipeptide repeat proteins from ALS/FTD C9orf72 transcripts. Nature Communications.

[bib60] Tao Z, Wang H, Xia Q, Li K, Li K, Jiang X, Xu G, Wang G, Ying Z (2015). Nucleolar stress and impaired stress granule formation contribute to C9orf72 Ran translation-induced cytotoxicity. Human Molecular Genetics.

[bib61] Therrien M, Rouleau GA, Dion PA, Parker JA, Dupuy D (2013). Deletion of C9orf72 results in motor neuron degeneration and stress sensitivity in *C. elegans*. PLOS ONE.

[bib62] Tian B, White RJ, Xia T, Welle S, Turner DH, Mathews MB, Thornton CA (2000). Expanded CUG repeat RNAs form hairpins that activate the double-stranded RNA-dependent protein kinase PKR. RNA.

[bib63] Tran H, Almeida S, Moore J, Gendron TF, Chalasani U, Lu Y, Du X, Nickerson JA, Petrucelli L, Weng Z, Gao FB (2015). Differential toxicity of nuclear RNA foci versus dipeptide repeat proteins in a *Drosophila* model of C9orf72 FTD/ALS. Neuron.

[bib64] Ugolino J, Ji YJ, Conchina K, Chu J, Nirujogi RS, Pandey A, Brady NR, Hamacher-Brady A, Wang J (2016). Loss of C9orf72 enhances autophagic activity via deregulated mTOR and TFEB signaling. PLOS Genetics.

[bib65] Valdez-Sinon AN, Lai A, Shi L, Lancaster CL, Gokhale A, Faundez V, Bassell GJ (2020). Cdh1-APC regulates protein synthesis and stress granules in neurons through an FMRP-dependent mechanism. IScience.

[bib66] van Blitterswijk M, Gendron TF, Baker MC, DeJesus-Hernandez M, Finch NA, Brown PH, Daughrity LM, Murray ME, Heckman MG, Jiang J, Lagier-Tourenne C, Edbauer D, Cleveland DW, Josephs KA, Parisi JE, Knopman DS, Petersen RC, Petrucelli L, Boeve BF, Graff-Radford NR, Boylan KB, Dickson DW, Rademakers R (2015). Novel clinical associations with specific C9orf72 transcripts in patients with repeat expansions in C9orf72. Acta Neuropathologica.

[bib67] Vatsavayai SC, Nana AL, Yokoyama JS, Seeley WW (2019). C9orf72-FTD/ALS pathogenesis: evidence from human neuropathological studies. Acta Neuropathologica.

[bib68] Wang ZF, Ursu A, Childs-Disney JL, Guertler R, Yang WY, Bernat V, Rzuczek SG, Fuerst R, Zhang YJ, Gendron TF, Yildirim I, Dwyer BG, Rice JE, Petrucelli L, Disney MD (2019). The hairpin form of R (G4C2) EXP in c9als/FTD is repeat-associated non-ATG translated and a target for bioactive small molecules. Cell Chemical Biology.

[bib69] Wen X, Tan W, Westergard T, Krishnamurthy K, Markandaiah SS, Shi Y, Lin S, Shneider NA, Monaghan J, Pandey UB, Pasinelli P, Ichida JK, Trotti D (2014). Antisense proline-arginine Ran dipeptides linked to C9ORF72-ALS/FTD form toxic nuclear aggregates that initiate in vitro and in vivo neuronal death. Neuron.

[bib70] Weskamp K, Safren N, Miguez R, Barmada S (2019). Monitoring neuronal survival via longitudinal fluorescence microscopy. Journal of Visualized Experiments.

[bib71] Xu Z, Poidevin M, Li X, Li Y, Shu L, Nelson DL, Li H, Hales CM, Gearing M, Wingo TS, Jin P (2013). Expanded GGGGCC repeat RNA associated with amyotrophic lateral sclerosis and frontotemporal dementia causes neurodegeneration. PNAS.

[bib72] Yamakawa M, Ito D, Honda T, Kubo K, Noda M, Nakajima K, Suzuki N (2015). Characterization of the dipeptide repeat protein in the molecular pathogenesis of c9FTD/ALS. Human Molecular Genetics.

[bib73] Yang D, Abdallah A, Li Z, Lu Y, Almeida S, Gao FB (2015). Ftd/Als-Associated poly (GR) protein impairs the Notch pathway and is recruited by poly (GA) into cytoplasmic inclusions. Acta Neuropathologica.

[bib74] Zhang Y-J, Jansen-West K, Xu Y-F, Gendron TF, Bieniek KF, Lin W-L, Sasaguri H, Caulfield T, Hubbard J, Daughrity L, Chew J, Belzil VV, Prudencio M, Stankowski JN, Castanedes-Casey M, Whitelaw E, Ash PEA, DeTure M, Rademakers R, Boylan KB, Dickson DW, Petrucelli L (2014). Aggregation-Prone c9FTD/ALS poly (GA) RAN-translated proteins cause neurotoxicity by inducing ER stress. Acta Neuropathologica.

[bib75] Zhang K, Donnelly CJ, Haeusler AR, Grima JC, Machamer JB, Steinwald P, Daley EL, Miller SJ, Cunningham KM, Vidensky S, Gupta S, Thomas MA, Hong I, Chiu SL, Huganir RL, Ostrow LW, Matunis MJ, Wang J, Sattler R, Lloyd TE, Rothstein JD (2015). The C9orf72 repeat expansion disrupts nucleocytoplasmic transport. Nature.

[bib76] Zhang YJ, Gendron TF, Grima JC, Sasaguri H, Jansen-West K, Xu YF, Katzman RB, Gass J, Murray ME, Shinohara M, Lin WL, Garrett A, Stankowski JN, Daughrity L, Tong J, Perkerson EA, Yue M, Chew J, Castanedes-Casey M, Kurti A, Wang ZS, Liesinger AM, Baker JD, Jiang J, Lagier-Tourenne C, Edbauer D, Cleveland DW, Rademakers R, Boylan KB, Bu G, Link CD, Dickey CA, Rothstein JD, Dickson DW, Fryer JD, Petrucelli L (2016). C9Orf72 poly (GA) aggregates sequester and impair HR23 and nucleocytoplasmic transport proteins. Nature Neuroscience.

[bib77] Zhang YJ, Gendron TF, Ebbert MTW, O’Raw AD, Yue M, Jansen-West K, Zhang X, Prudencio M, Chew J, Cook CN, Daughrity LM, Tong J, Song Y, Pickles SR, Castanedes-Casey M, Kurti A, Rademakers R, Oskarsson B, Dickson DW, Hu W, Gitler AD, Fryer JD, Petrucelli L (2018). Poly (GR) impairs protein translation and stress granule dynamics in c9orf72-associated frontotemporal dementia and amyotrophic lateral sclerosis. Nature Medicine.

[bib78] Zhang Y-J, Guo L, Gonzales PK, Gendron TF, Wu Y, Jansen-West K, O’Raw AD, Pickles SR, Prudencio M, Carlomagno Y, Gachechiladze MA, Ludwig C, Tian R, Chew J, DeTure M, Lin W-L, Tong J, Daughrity LM, Yue M, Song Y, Andersen JW, Castanedes-Casey M, Kurti A, Datta A, Antognetti G, McCampbell A, Rademakers R, Oskarsson B, Dickson DW, Kampmann M, Ward ME, Fryer JD, Link CD, Shorter J, Petrucelli L (2019). Heterochromatin anomalies and double-stranded RNA accumulation underlie C9orf72 poly (PR) toxicity. Science.

[bib79] Zhu Q, Jiang J, Gendron TF, McAlonis-Downes M, Jiang L, Taylor A, Diaz Garcia S, Ghosh Dastidar S, Rodriguez MJ, King P, Zhang Y, La Spada AR, Xu H, Petrucelli L, Ravits J, Da Cruz S, Lagier-Tourenne C, Cleveland DW (2020). Reduced C9orf72 function exacerbates gain of toxicity from als/ftd-causing repeat expansion in C9orf72. Nature Neuroscience.

[bib80] Zu T, Gibbens B, Doty NS, Gomes-Pereira M, Huguet A, Stone MD, Margolis J, Peterson M, Markowski TW, Ingram MAC, Nan Z, Forster C, Low WC, Schoser B, Somia NV, Clark HB, Schmechel S, Bitterman PB, Gourdon G, Swanson MS, Moseley M, Ranum LPW (2011). Non-ATG-initiated translation directed by microsatellite expansions. PNAS.

[bib81] Zu T, Liu Y, Bañez-Coronel M, Reid T, Pletnikova O, Lewis J, Miller TM, Harms MB, Falchook AE, Subramony SH, Ostrow LW, Rothstein JD, Troncoso JC, Ranum LPW (2013). RAN proteins and RNA foci from antisense transcripts in C9ORF72 ALS and frontotemporal dementia. PNAS.

[bib82] Zu T, Guo S, Bardhi O, Ryskamp DA, Li J, Khoramian Tusi S, Engelbrecht A, Klippel K, Chakrabarty P, Nguyen L, Golde TE, Sonenberg N, Ranum LPW (2020). Metformin inhibits RAN translation through PKR pathway and mitigates disease in *c9orf72* ALS/FTD mice. PNAS.

