# Supplementary figures and images for: Antisense, but not sense, repeat expanded RNAs activate PKR/eIF2α-dependent ISR in C9ORF72 FTD/ALS
Source: eLife. 2023 Apr 19;12:e85902. doi: 10.7554/eLife.85902 (PMC10188109; doi:10.7554/eLife.85902)

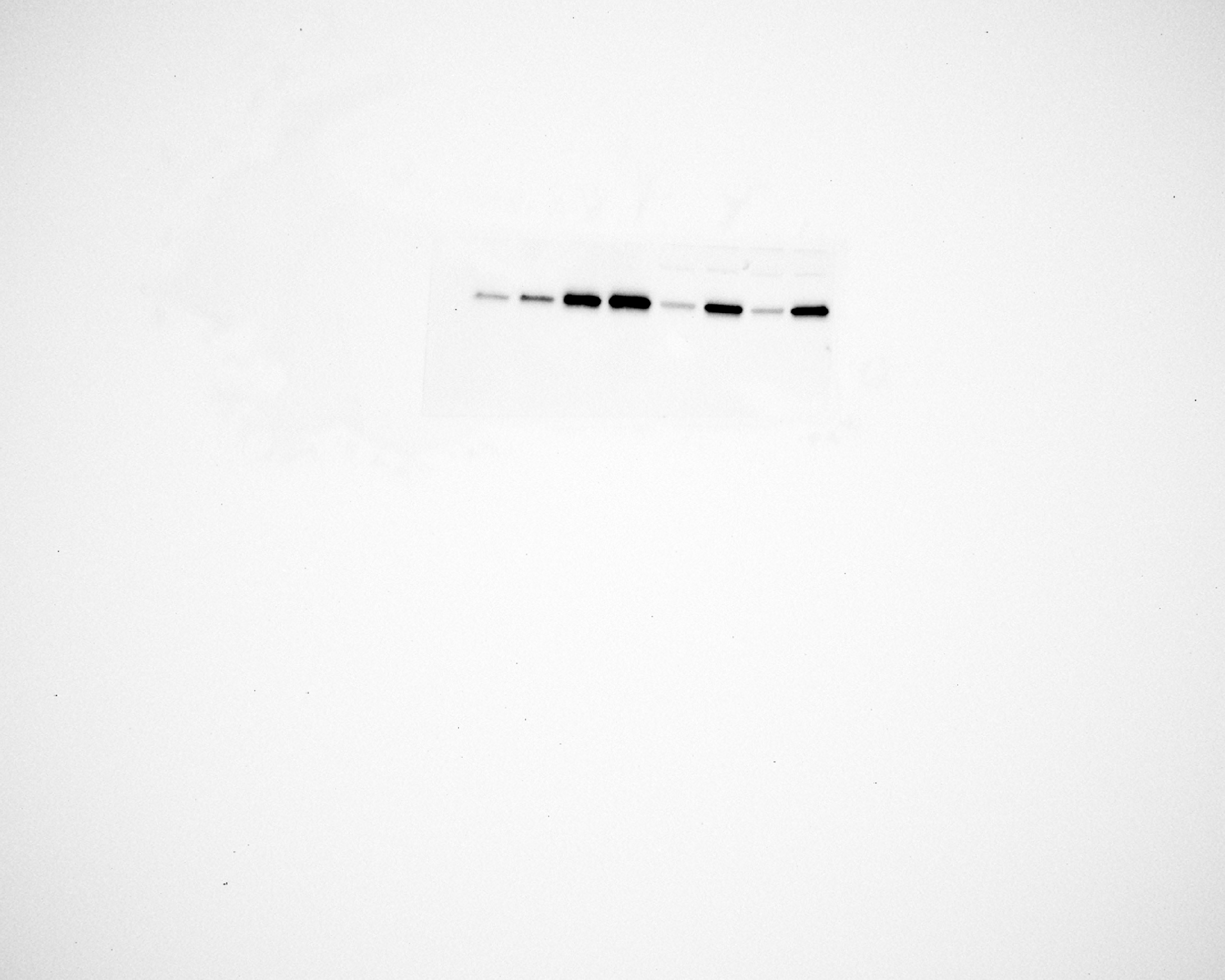

Supplement: Figure 1—source data 1. [file elife-85902-fig1-data1.zip › Figure 1-source data/Figure 1 unlabelled/pEIF2A 1F.tif]

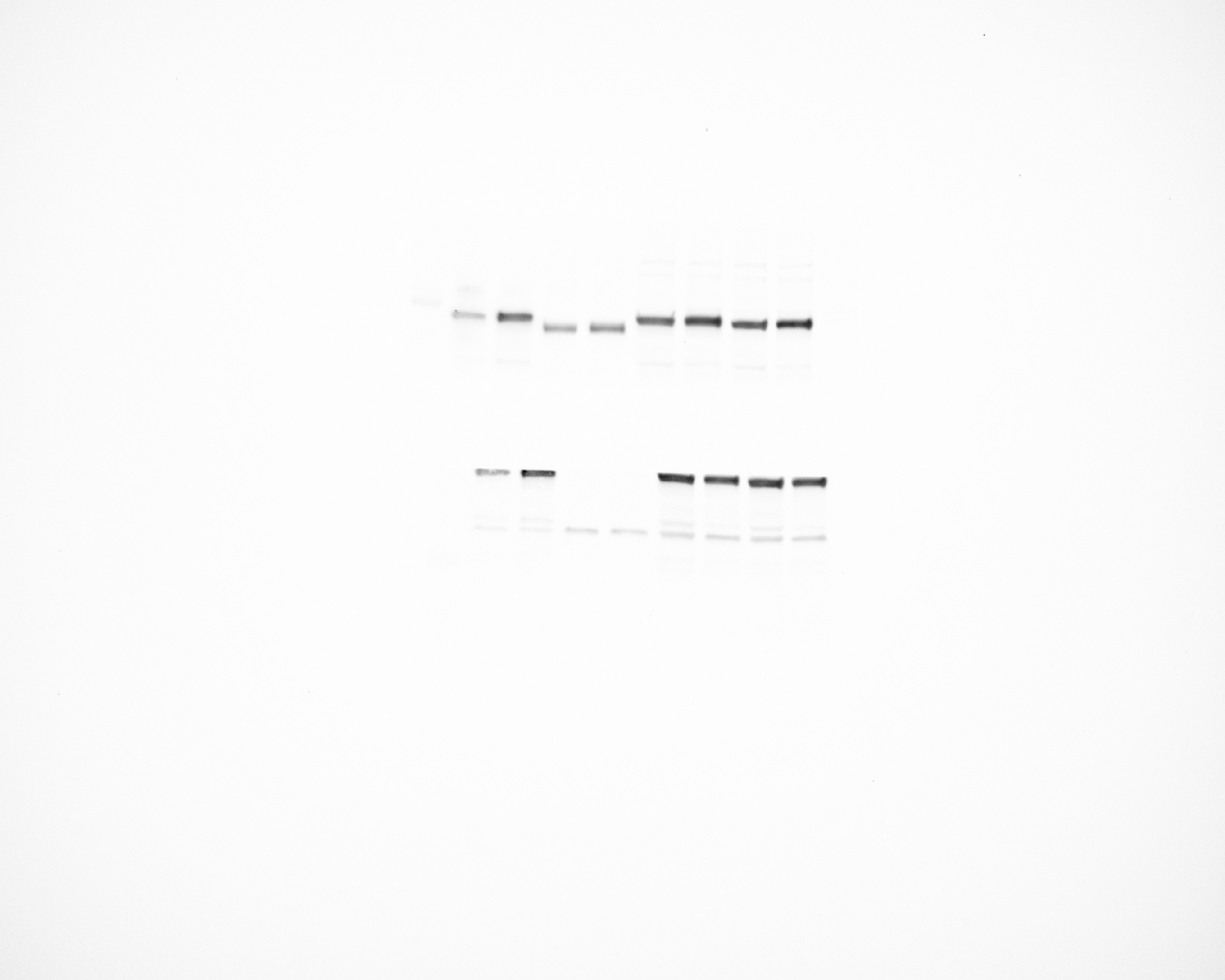

Supplement: Figure 1—source data 1. [file elife-85902-fig1-data1.zip › Figure 1-source data/Figure 1 unlabelled/PKR 1D.tif]

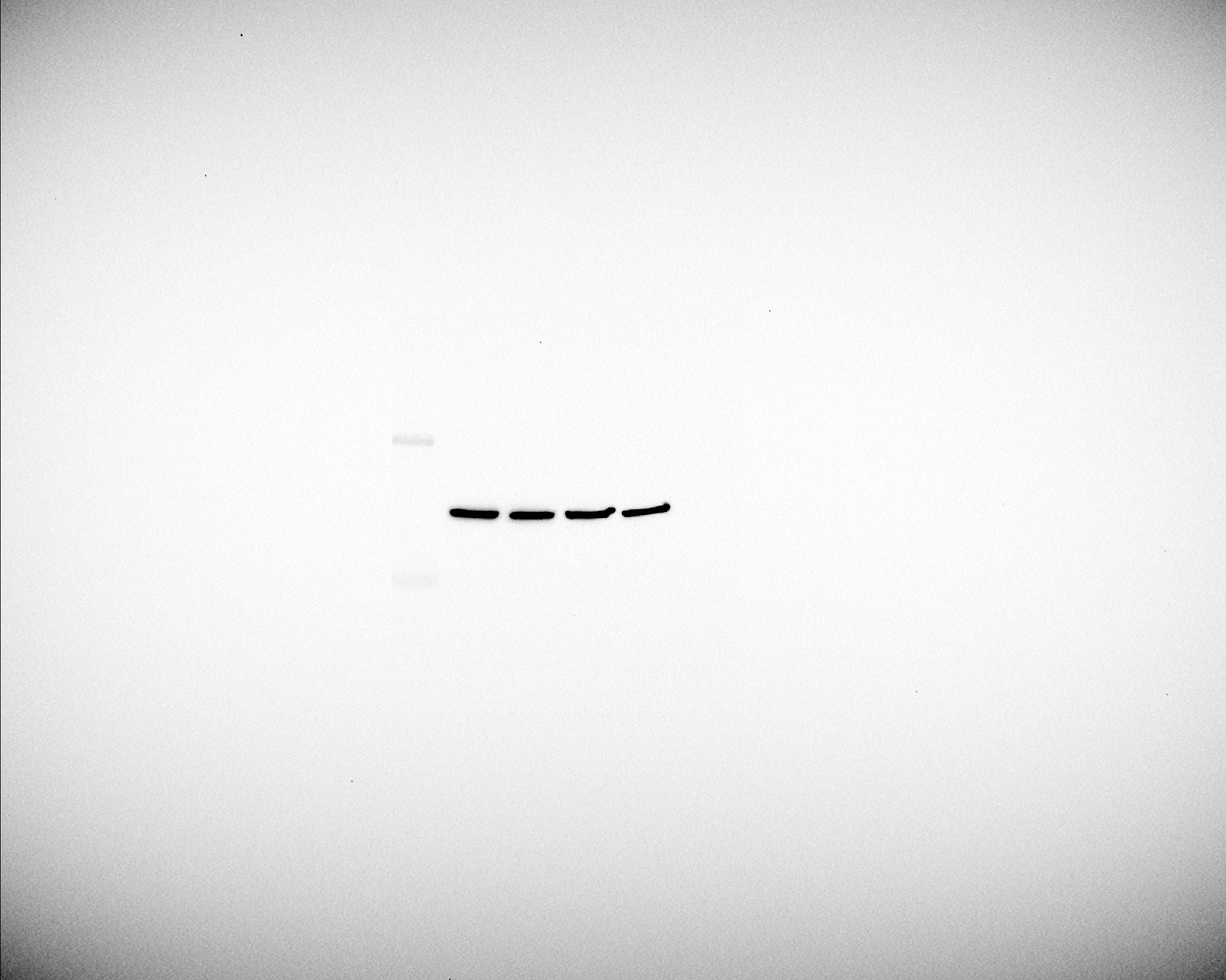

Supplement: Figure 1—source data 1. [file elife-85902-fig1-data1.zip › Figure 1-source data/Figure 1 unlabelled/GAPDH 1H.tif]

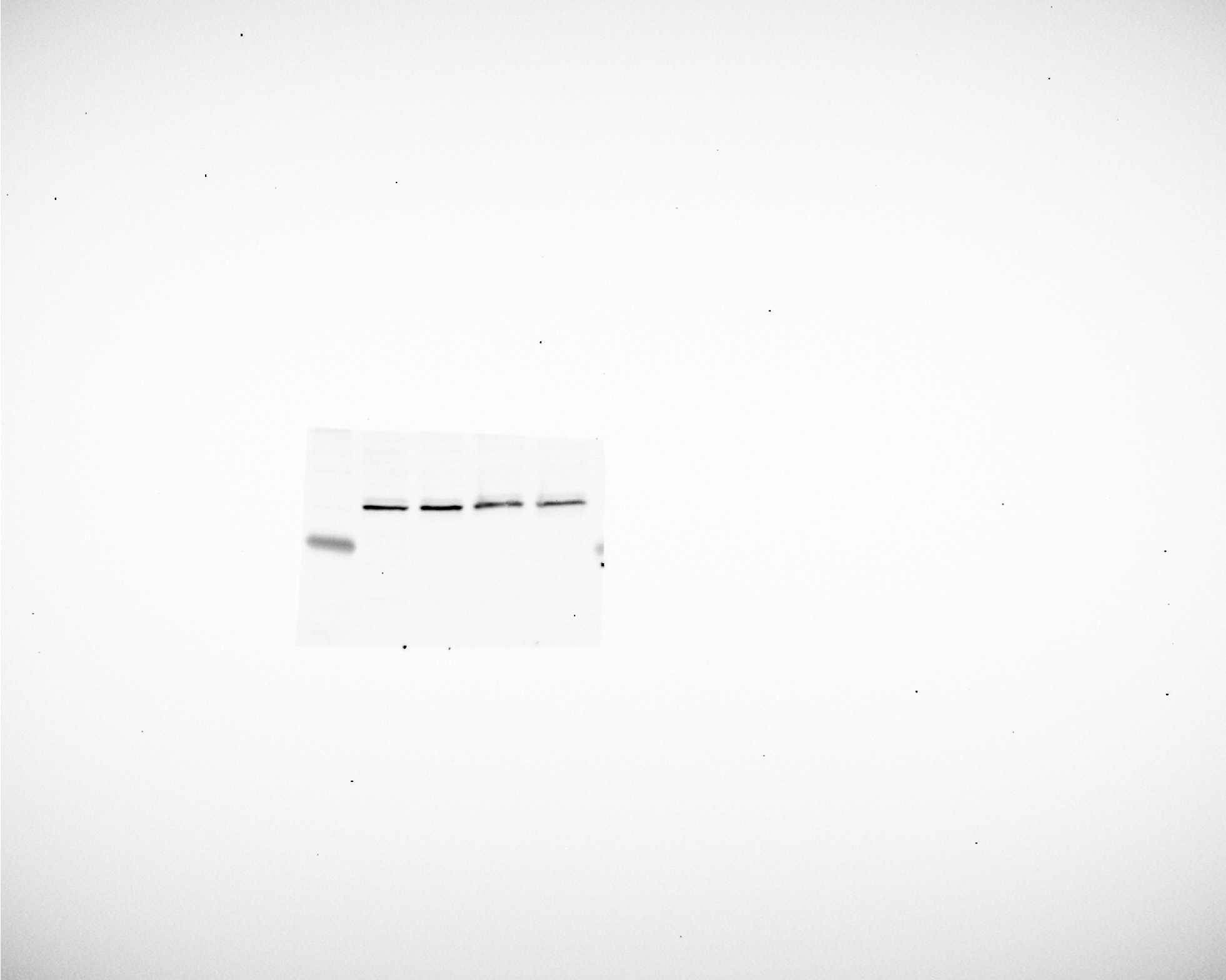

Supplement: Figure 1—source data 1. [file elife-85902-fig1-data1.zip › Figure 1-source data/Figure 1 unlabelled/EIF2A 1H.tif]

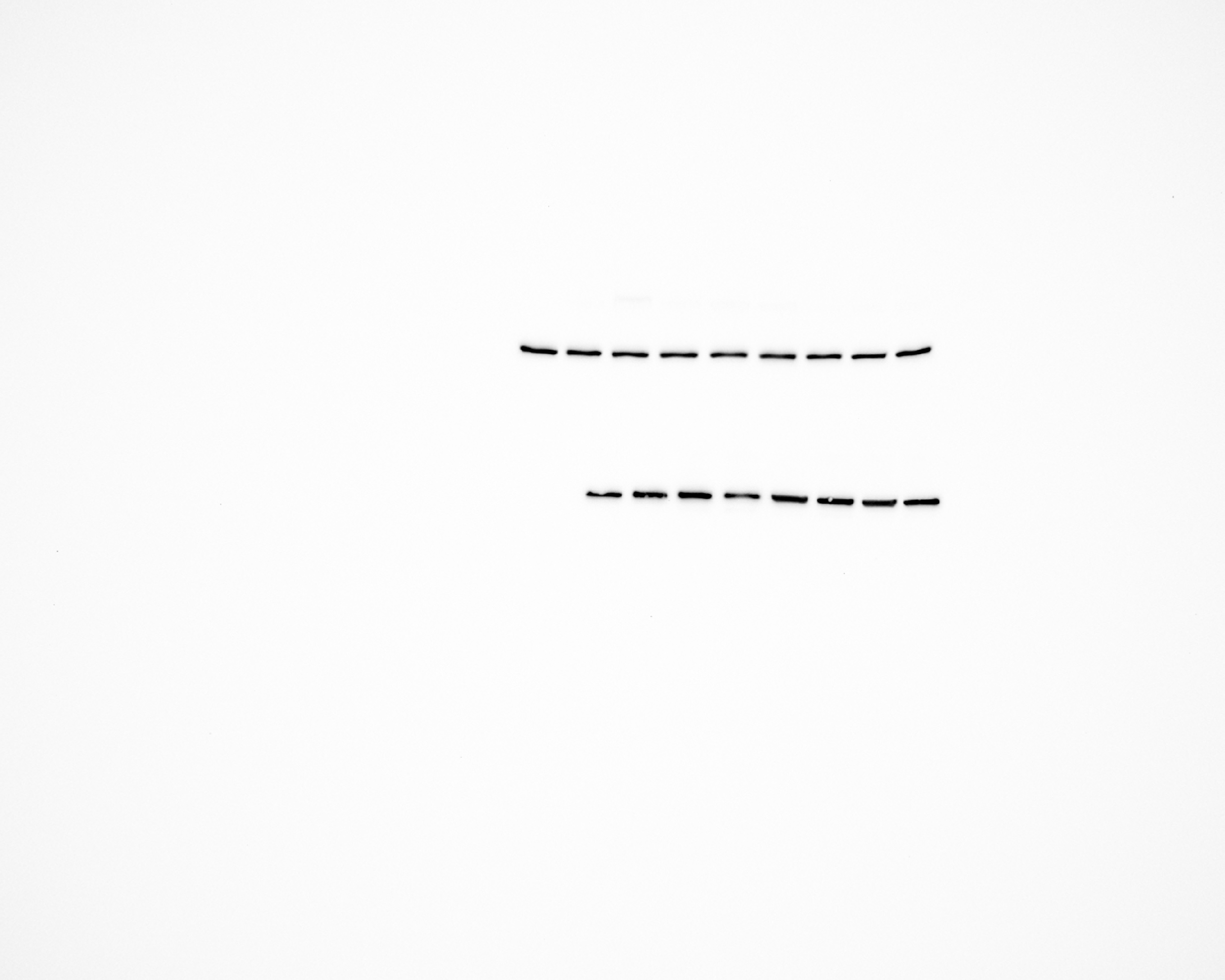

Supplement: Figure 1—source data 1. [file elife-85902-fig1-data1.zip › Figure 1-source data/Figure 1 unlabelled/GAPDH 1D 1F.tif]

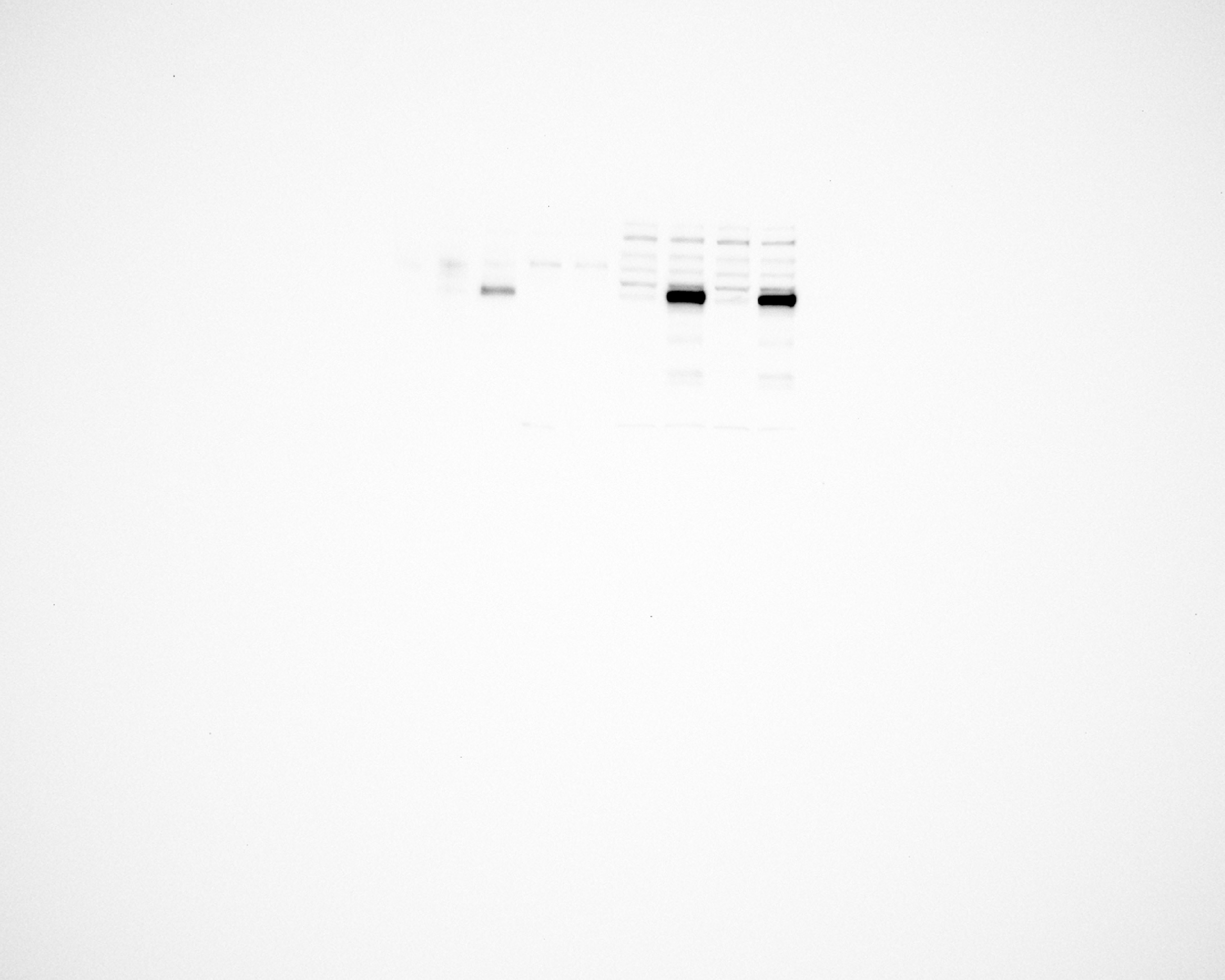

Supplement: Figure 1—source data 1. [file elife-85902-fig1-data1.zip › Figure 1-source data/Figure 1 unlabelled/pPKR 1D.tif]

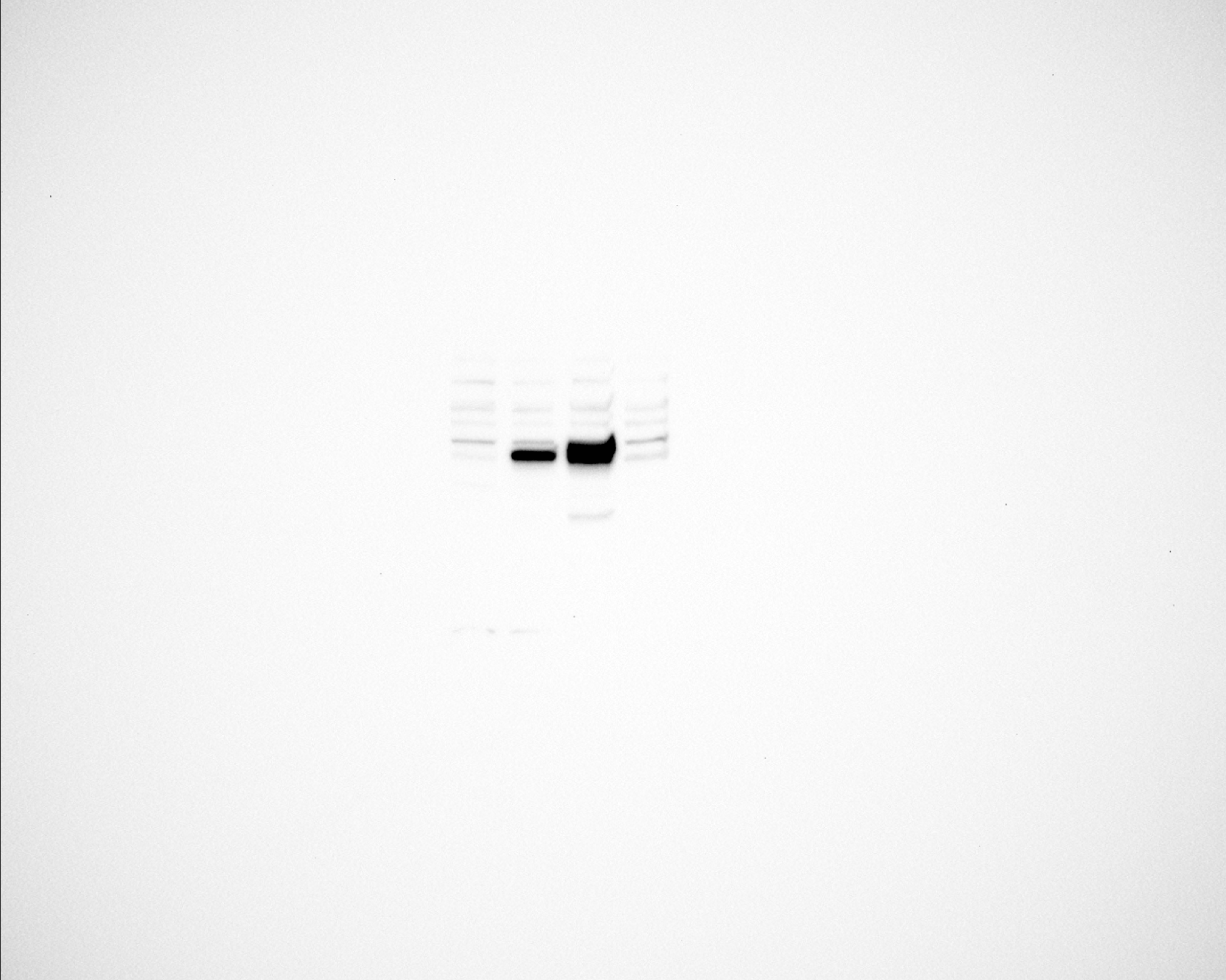

Supplement: Figure 1—source data 1. [file elife-85902-fig1-data1.zip › Figure 1-source data/Figure 1 unlabelled/pPKR 1H.tif]

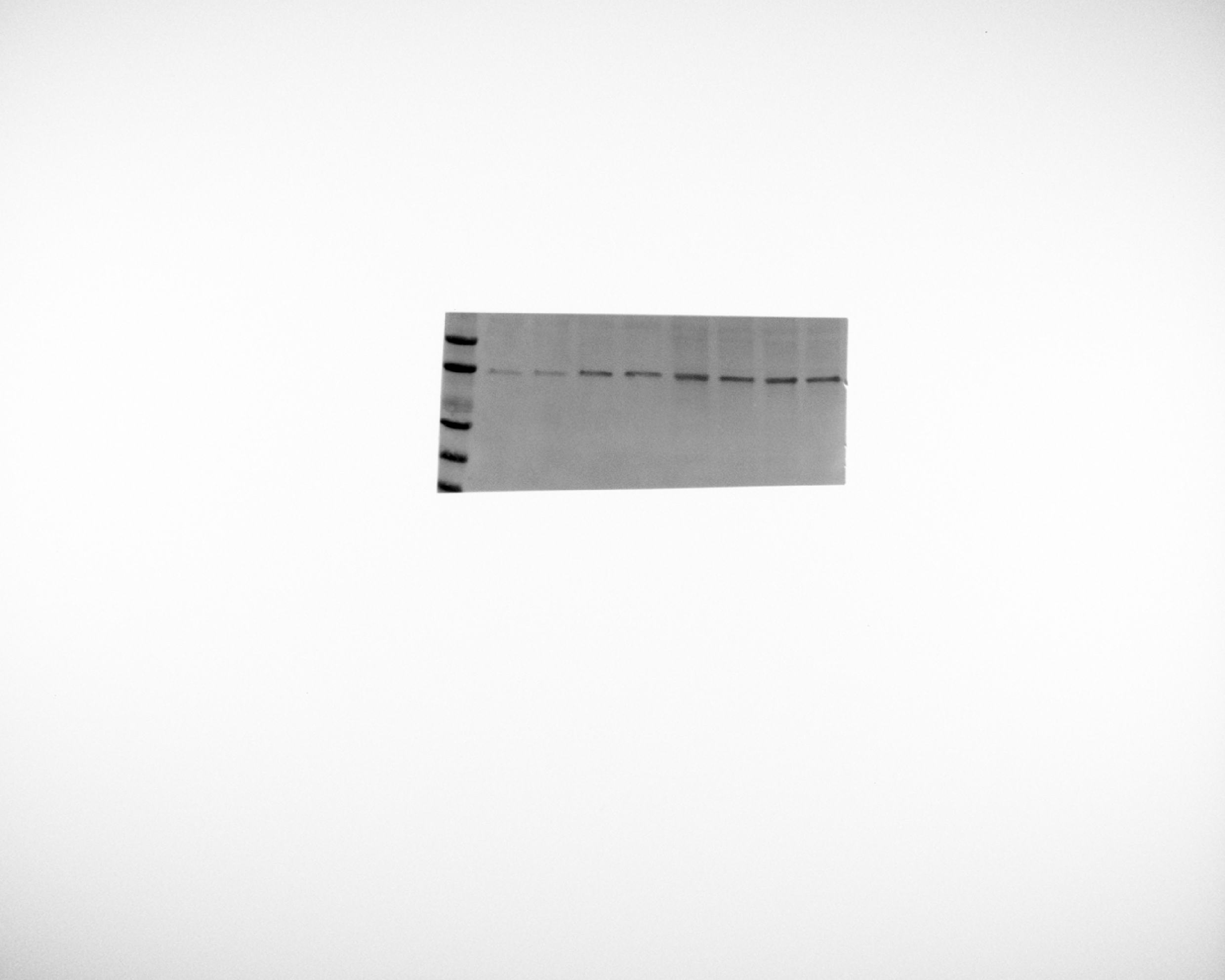

Supplement: Figure 1—source data 1. [file elife-85902-fig1-data1.zip › Figure 1-source data/Figure 1 unlabelled/EIF2A 1F.tif]

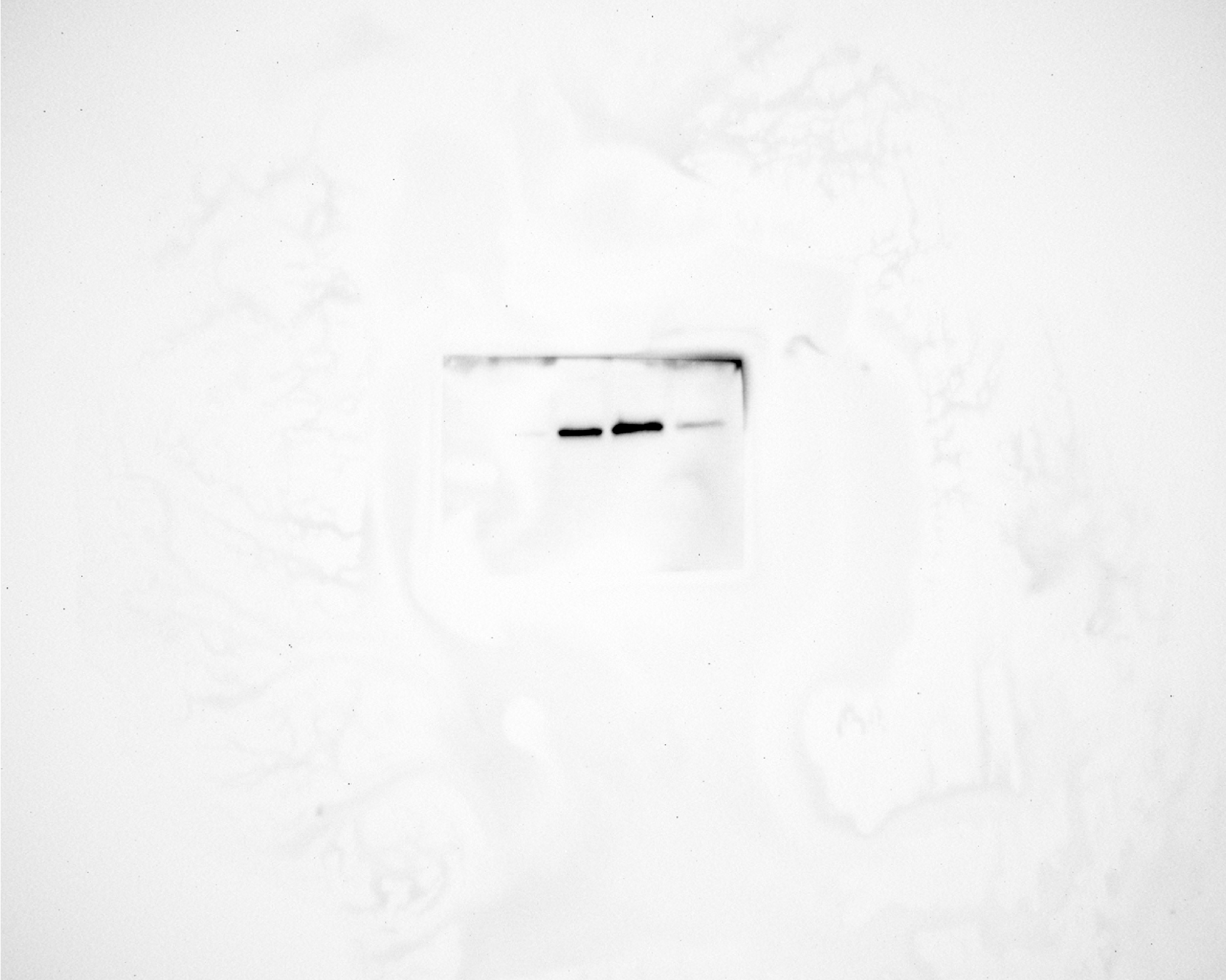

Supplement: Figure 1—source data 1. [file elife-85902-fig1-data1.zip › Figure 1-source data/Figure 1 unlabelled/pEIF2A 1H.tif]

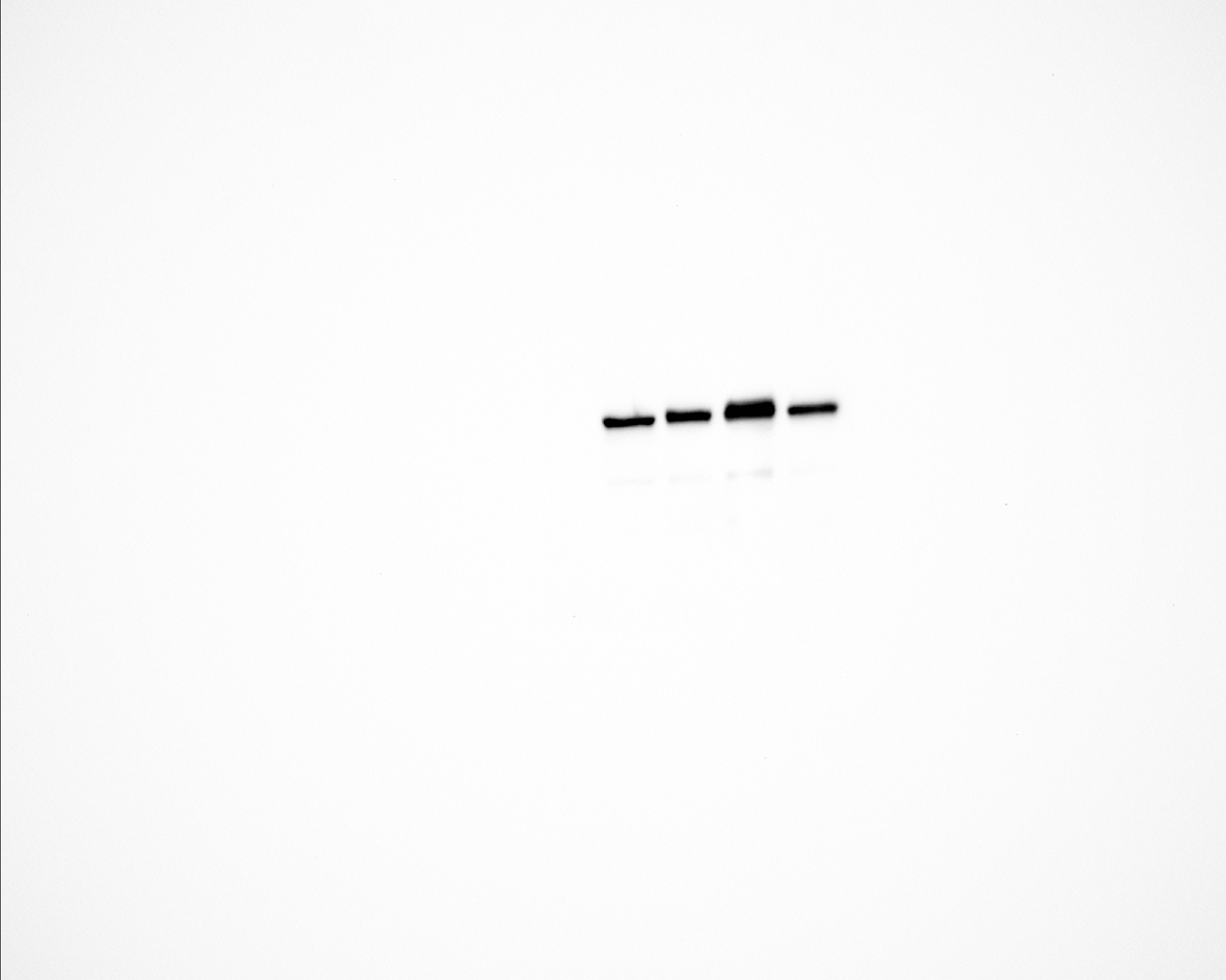

Supplement: Figure 1—source data 1. [file elife-85902-fig1-data1.zip › Figure 1-source data/Figure 1 unlabelled/PKR 1H.tif]

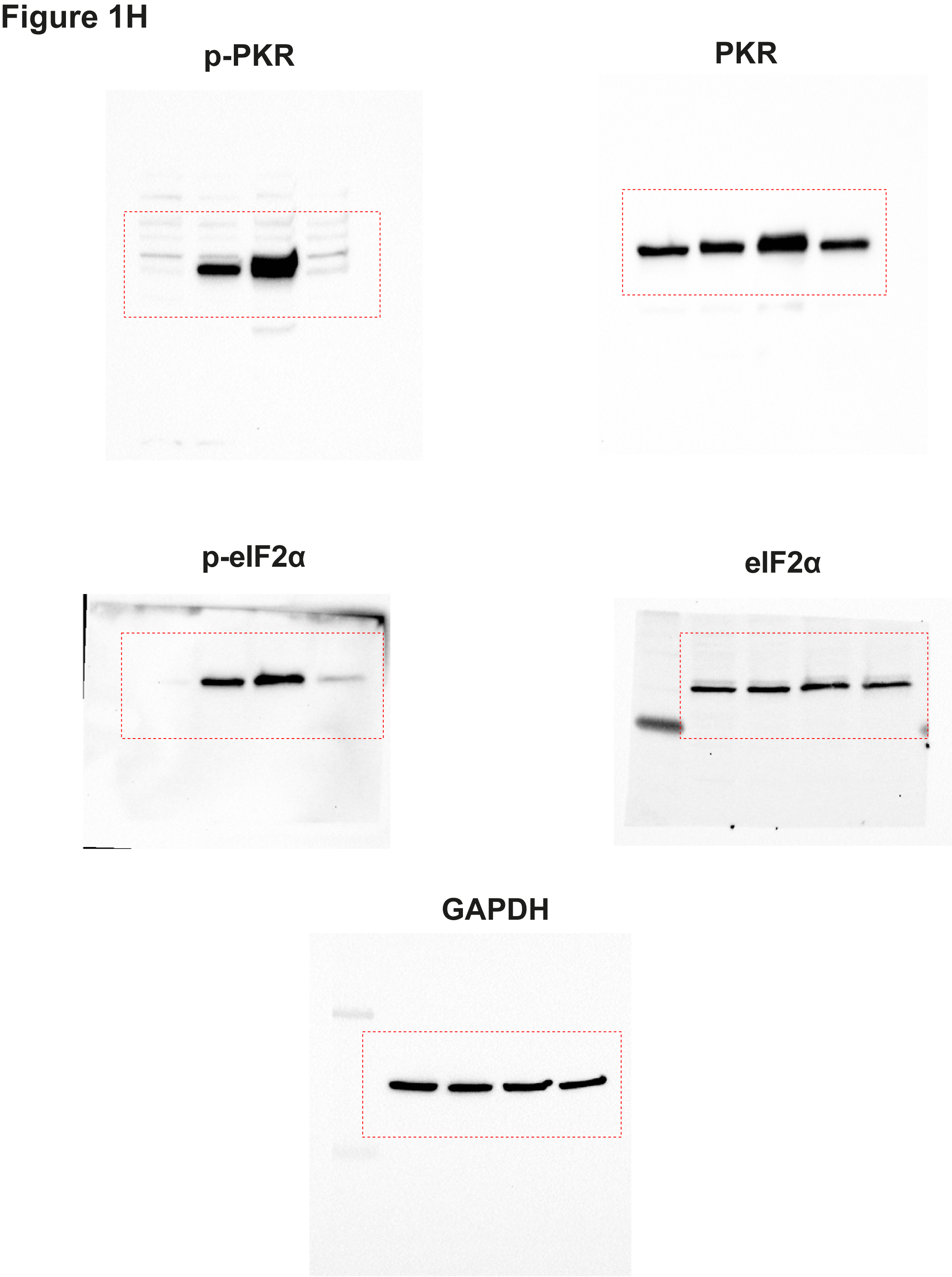

Supplement: Figure 1—source data 1. [file elife-85902-fig1-data1.zip › Figure 1-source data/Labelled/Figure 1-source data 2.tif]

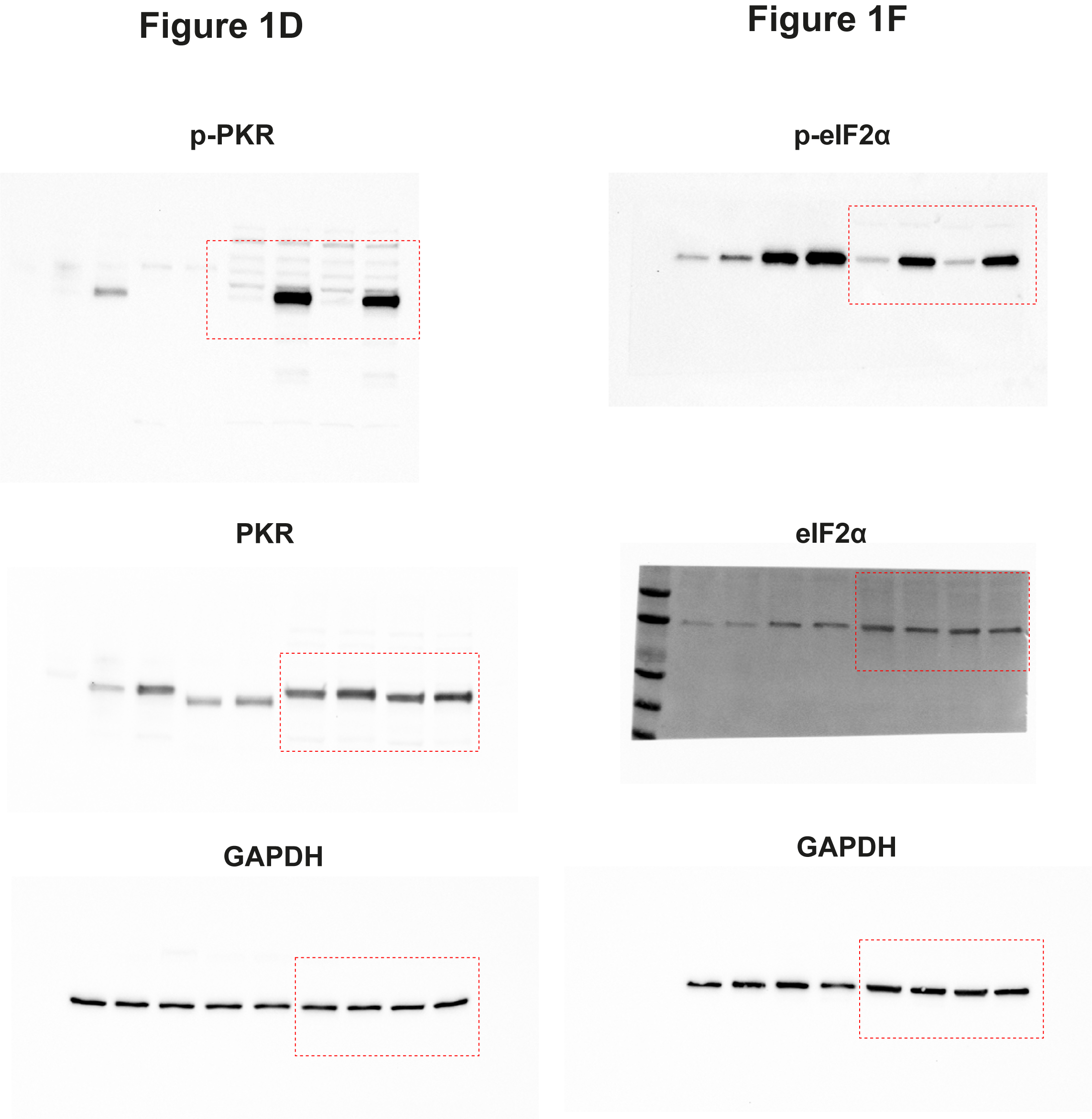

Supplement: Figure 1—source data 1. [file elife-85902-fig1-data1.zip › Figure 1-source data/Labelled/Figure 1-source data 1.tif]

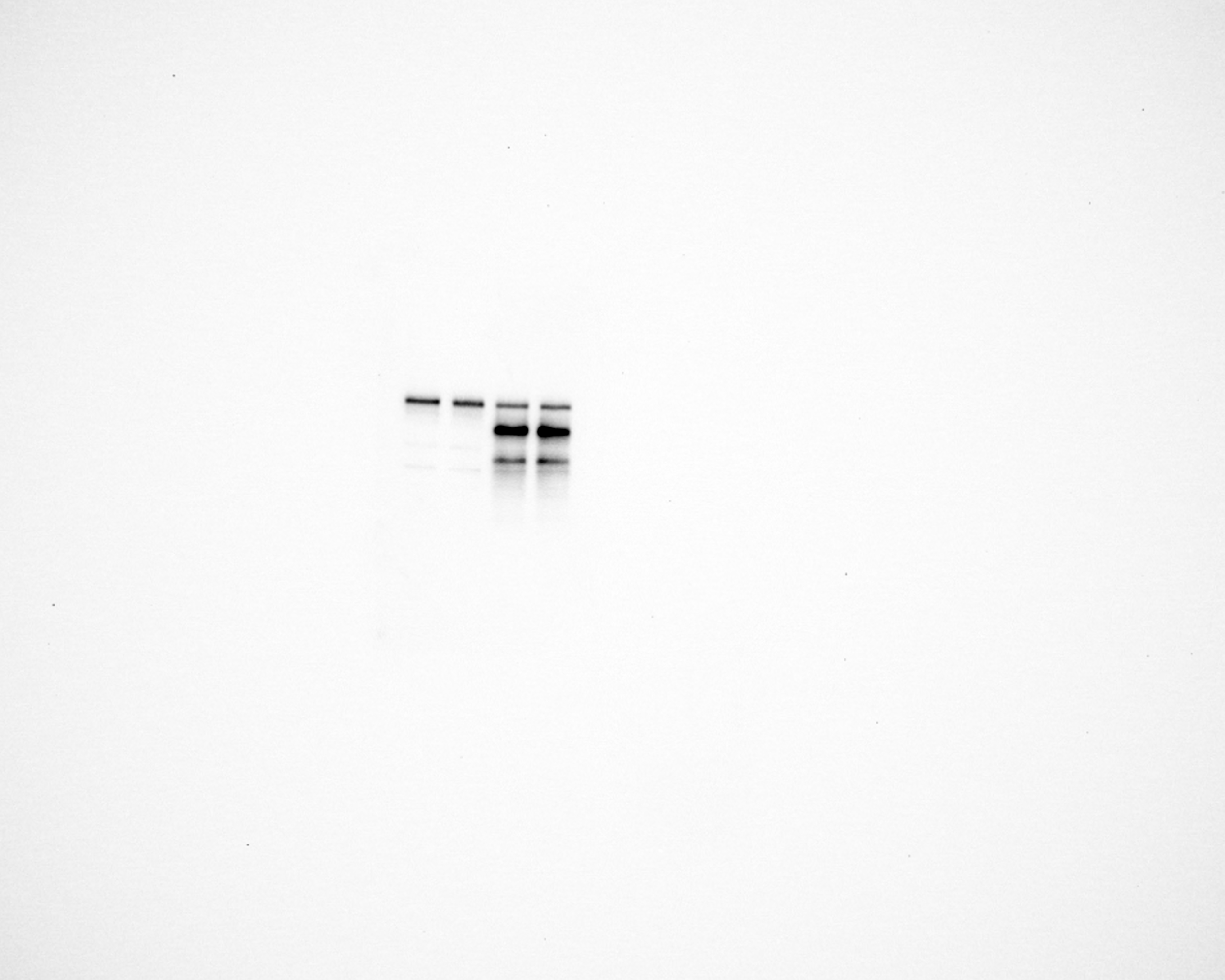

Supplement: Figure 1—source data 2. [file elife-85902-fig1-data2.zip › Figure 1-figure supplement 1-source data_/Unlabelled/B HA.tif]

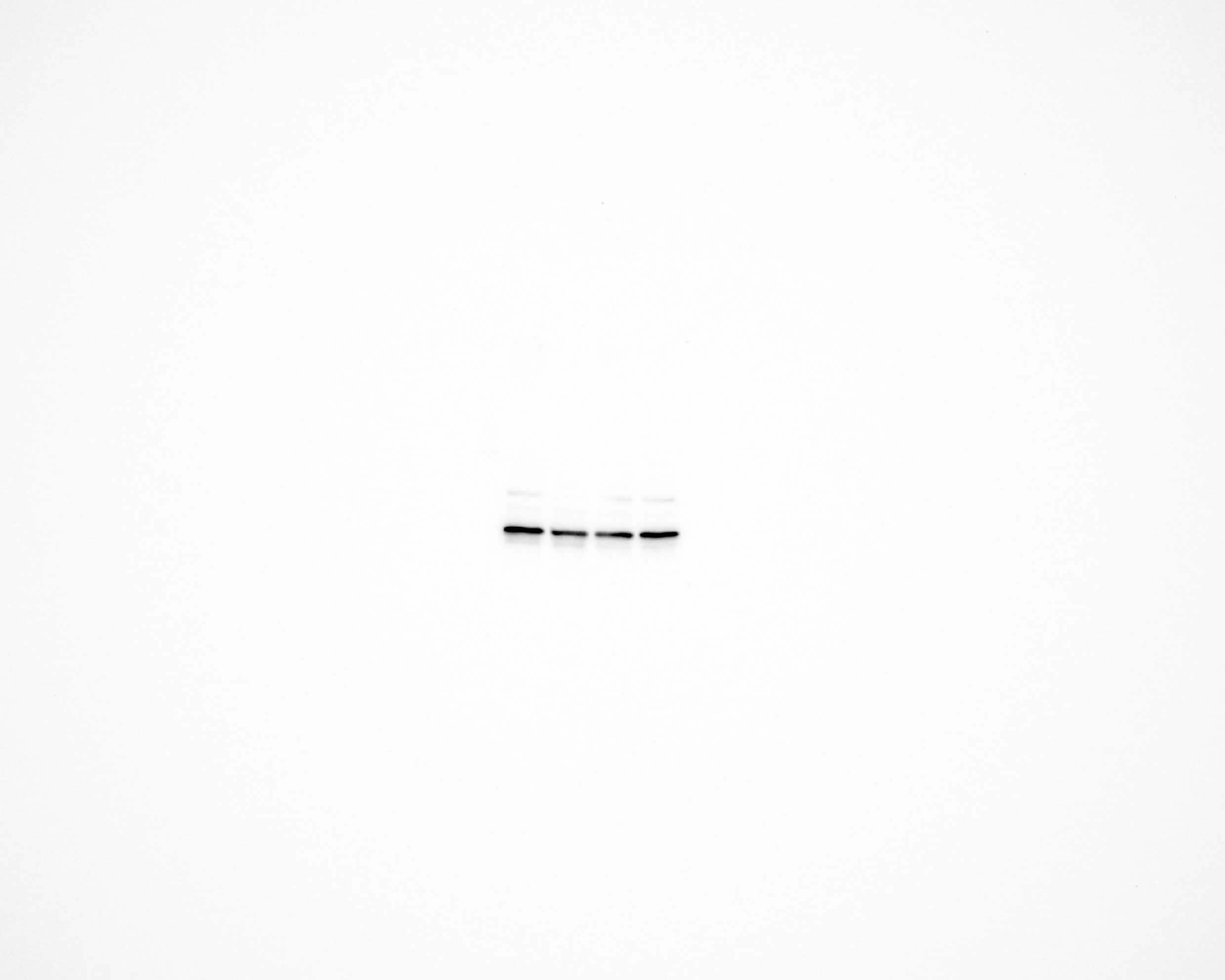

Supplement: Figure 1—source data 2. [file elife-85902-fig1-data2.zip › Figure 1-figure supplement 1-source data_/Unlabelled/B GAPDH.tif]

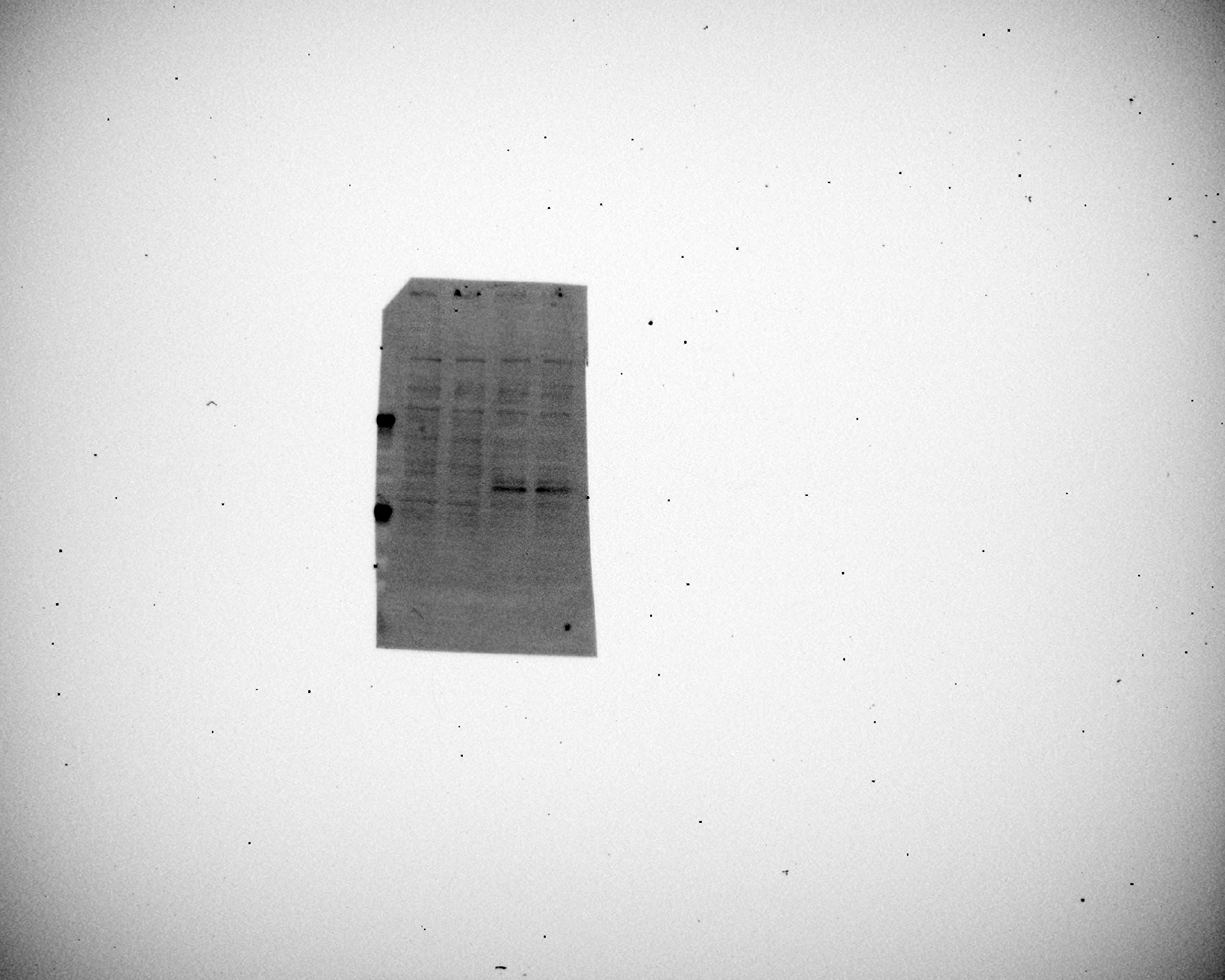

Supplement: Figure 1—source data 2. [file elife-85902-fig1-data2.zip › Figure 1-figure supplement 1-source data_/Unlabelled/B FLAG.tif]

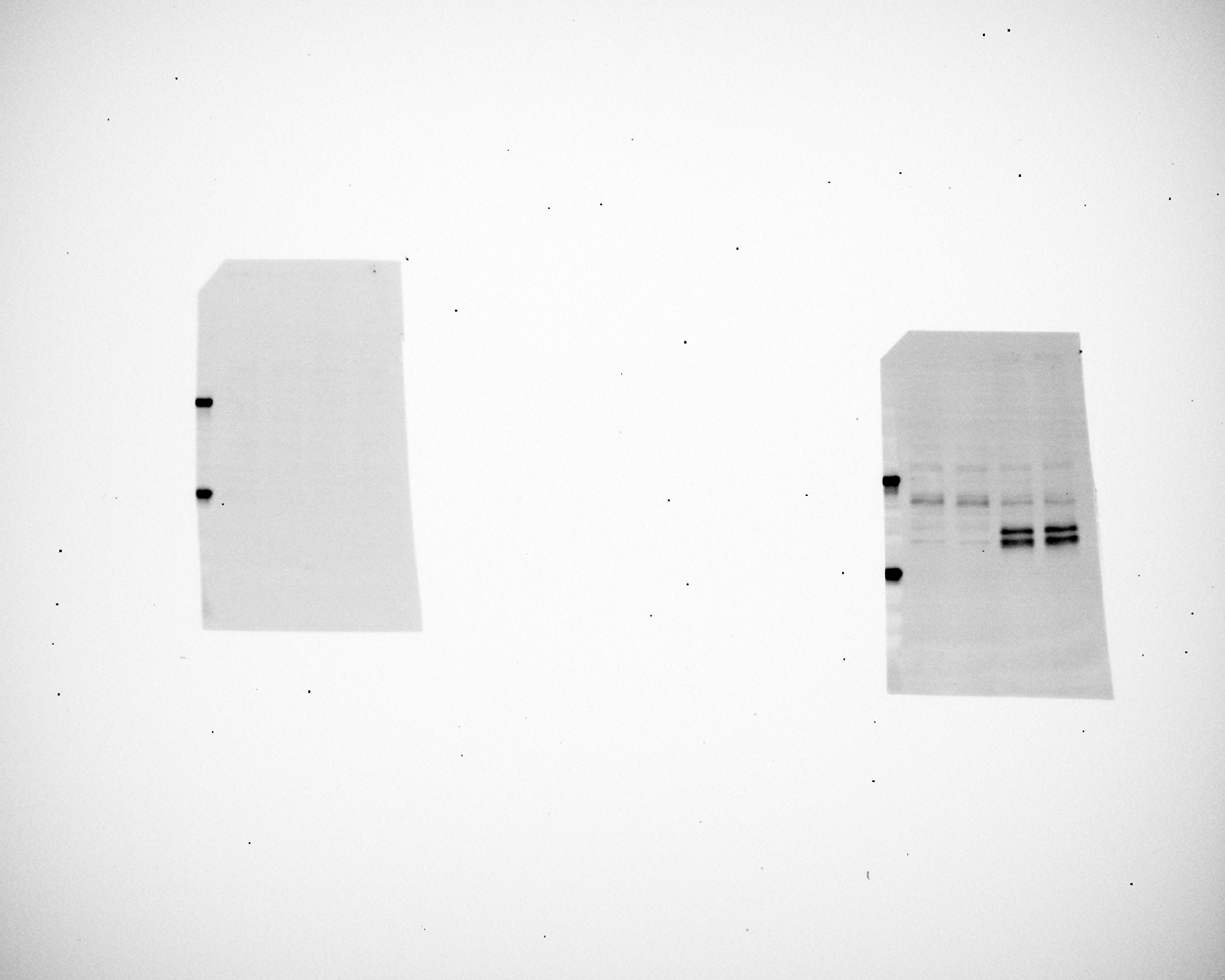

Supplement: Figure 1—source data 2. [file elife-85902-fig1-data2.zip › Figure 1-figure supplement 1-source data_/Unlabelled/B MYC.tif]

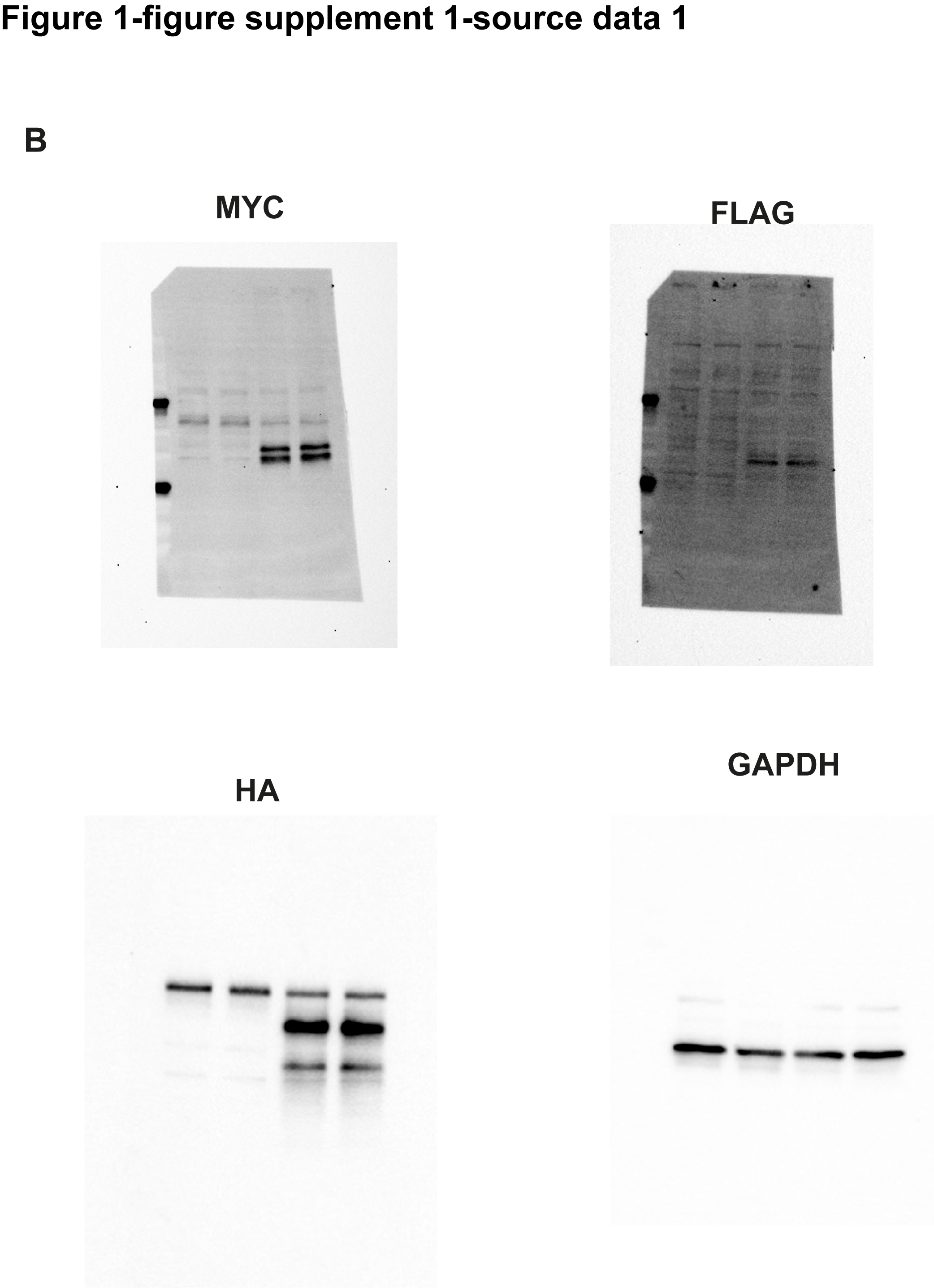

Supplement: Figure 1—source data 2. [file elife-85902-fig1-data2.zip › Figure 1-figure supplement 1-source data_/Labelled/Figure 1-figure supplement 1-source data 1.tif]

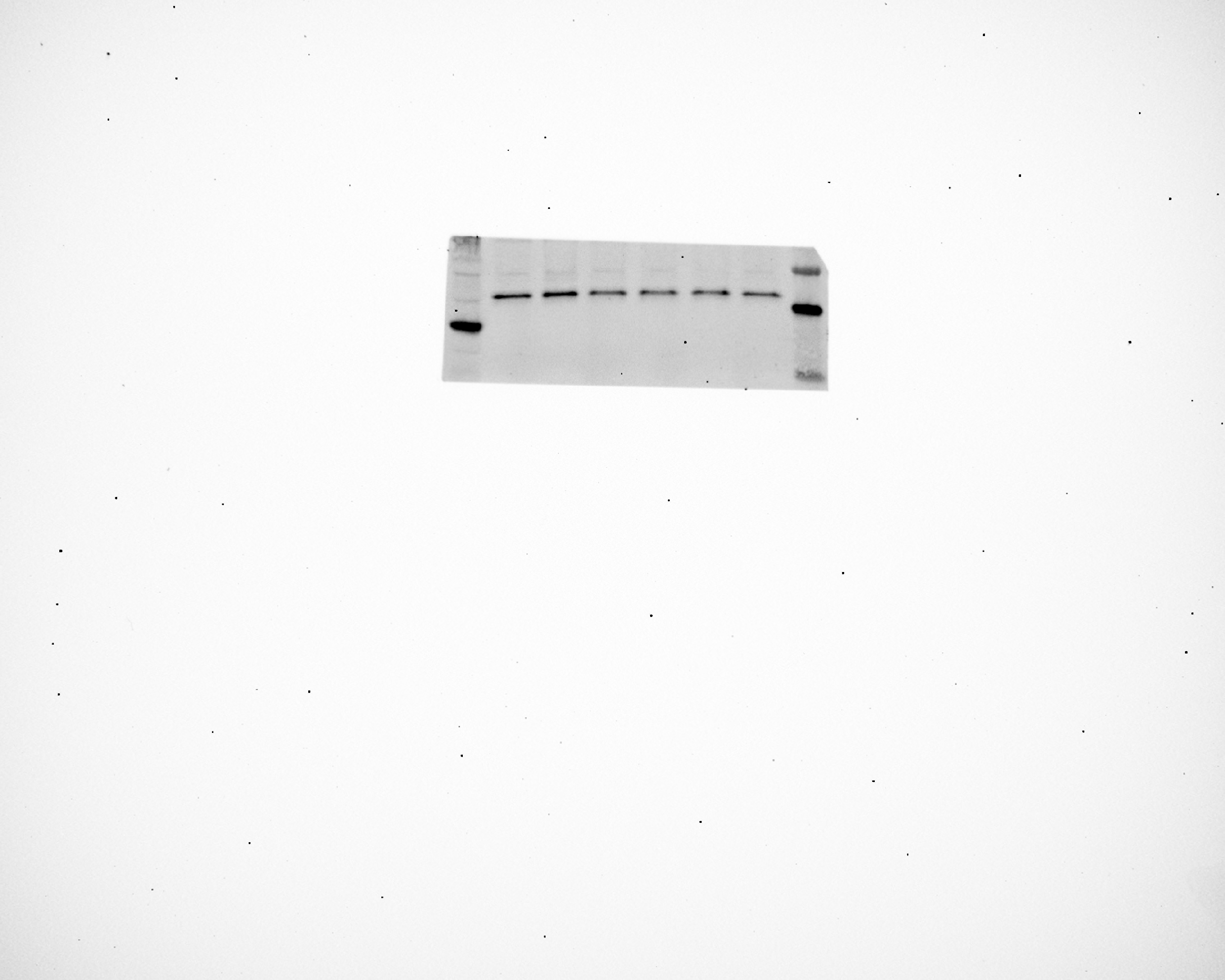

Supplement: Figure 1—figure supplement 2—source data 1. [file elife-85902-fig1-figsupp2-data1.zip › Figure 1-figure supplement 2-source data_/Unlabelled/C EIF2A.tif]

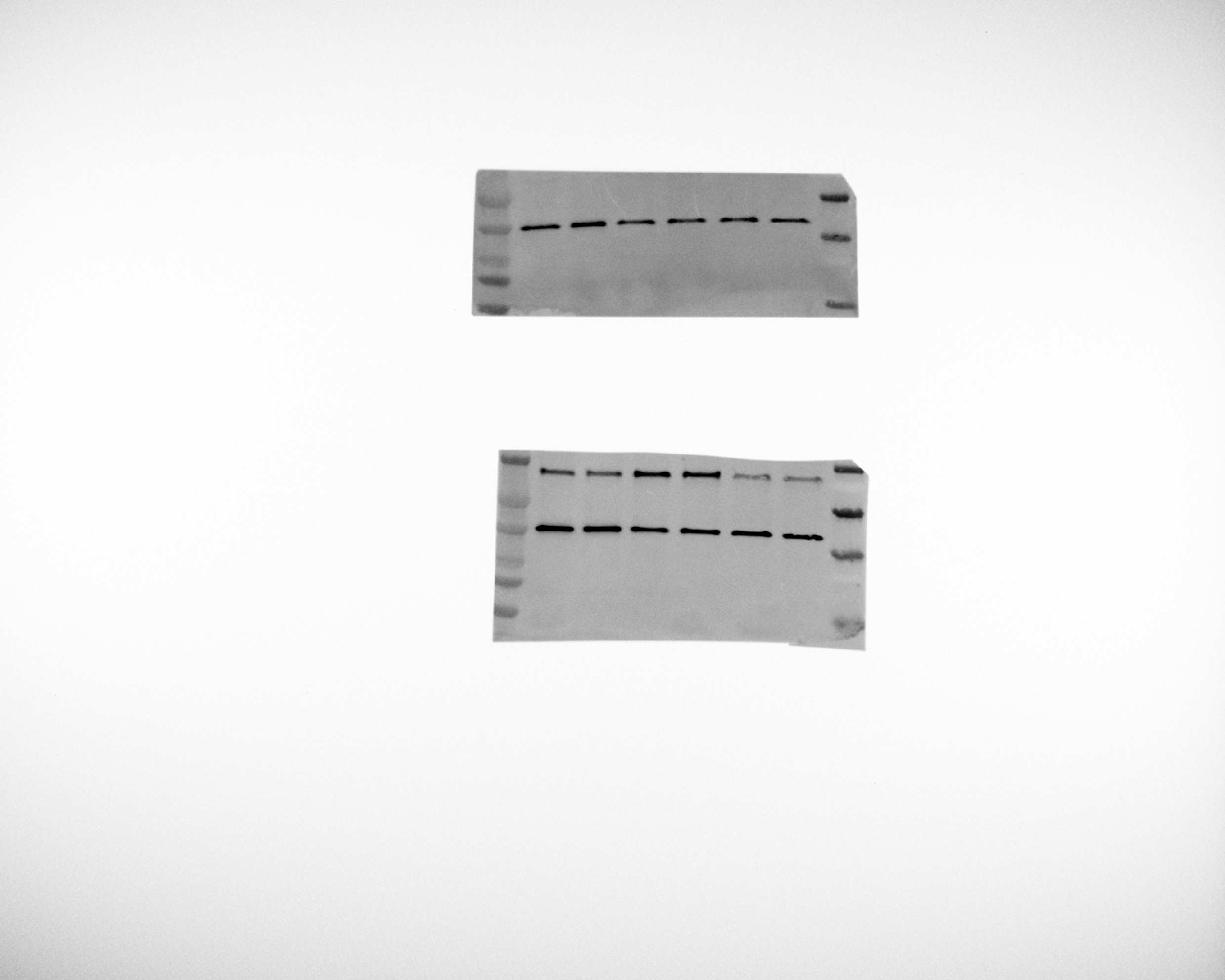

Supplement: Figure 1—figure supplement 2—source data 1. [file elife-85902-fig1-figsupp2-data1.zip › Figure 1-figure supplement 2-source data_/Unlabelled/B GAPDH.tif]

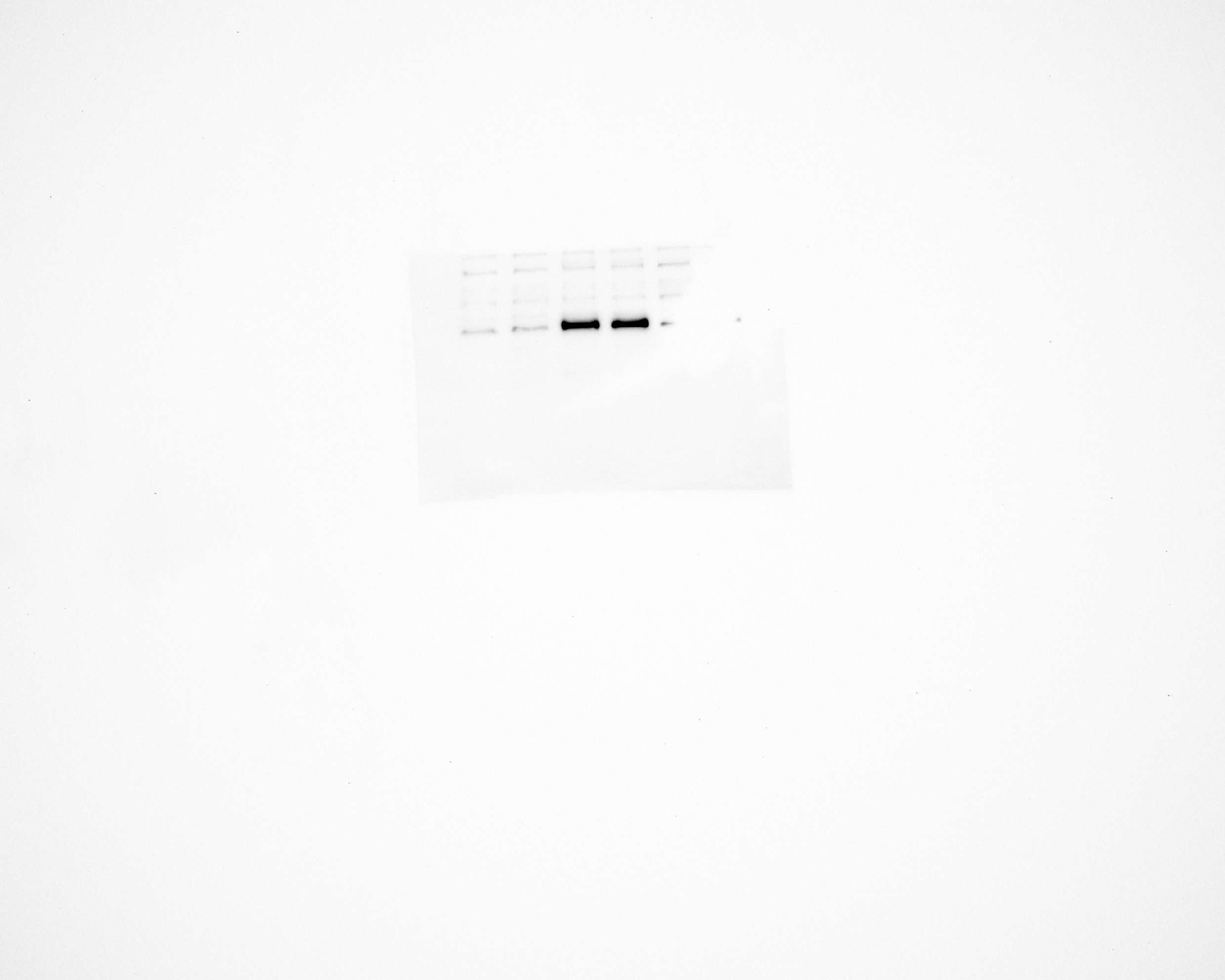

Supplement: Figure 1—figure supplement 2—source data 1. [file elife-85902-fig1-figsupp2-data1.zip › Figure 1-figure supplement 2-source data_/Unlabelled/B pPKR.tif]

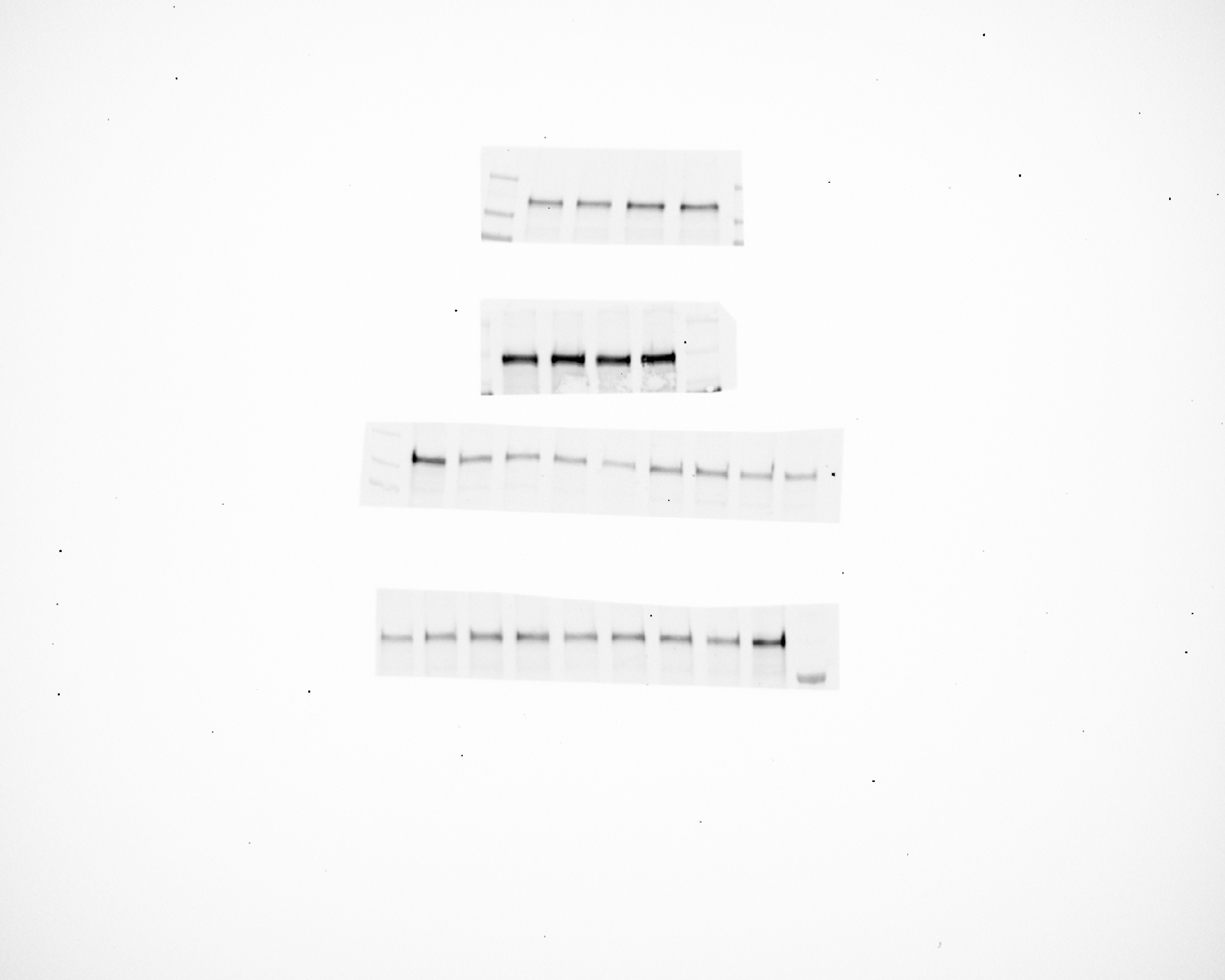

Supplement: Figure 1—figure supplement 2—source data 1. [file elife-85902-fig1-figsupp2-data1.zip › Figure 1-figure supplement 2-source data_/Unlabelled/A pPERK PERK.tif]

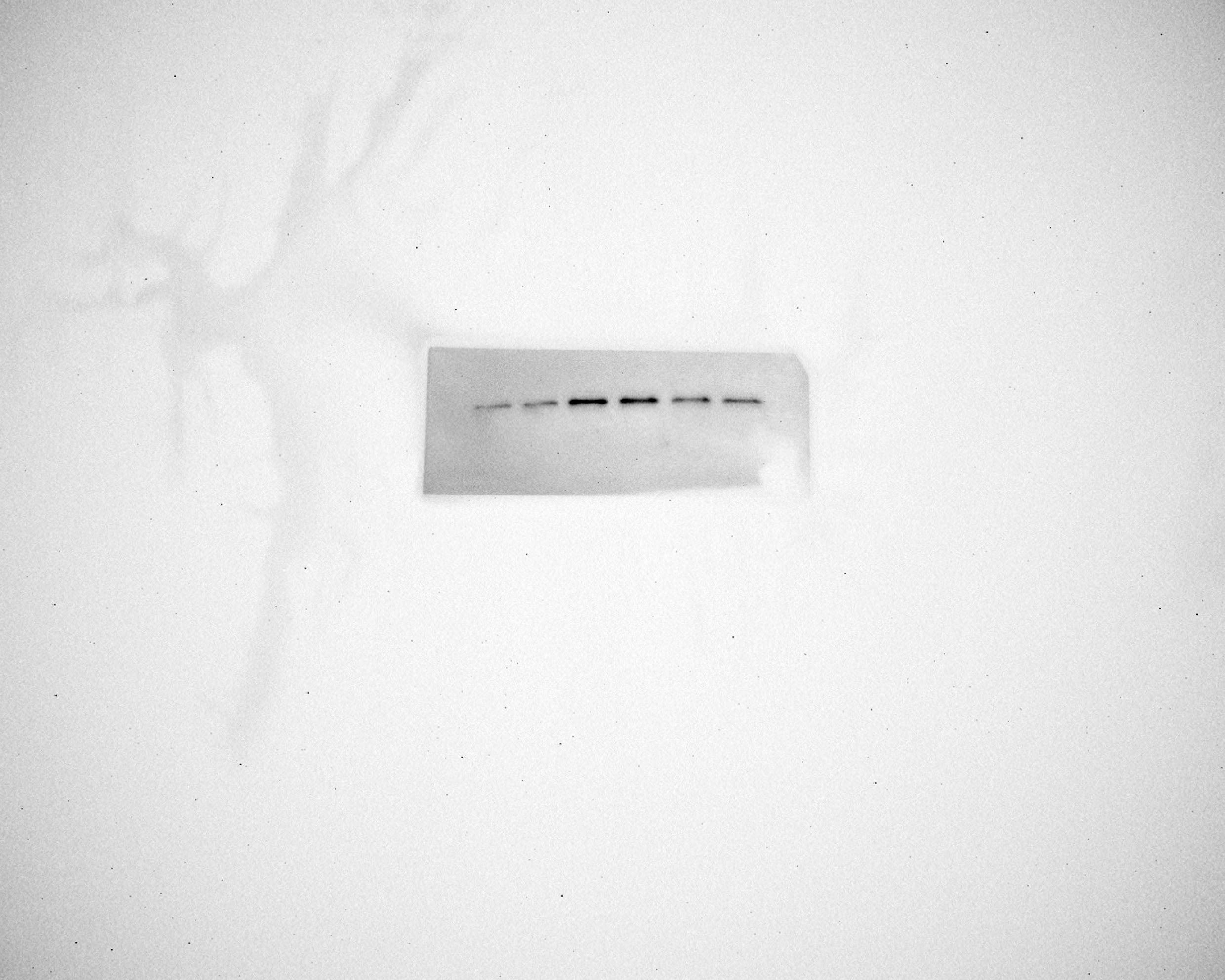

Supplement: Figure 1—figure supplement 2—source data 1. [file elife-85902-fig1-figsupp2-data1.zip › Figure 1-figure supplement 2-source data_/Unlabelled/C PEIF2A.tif]

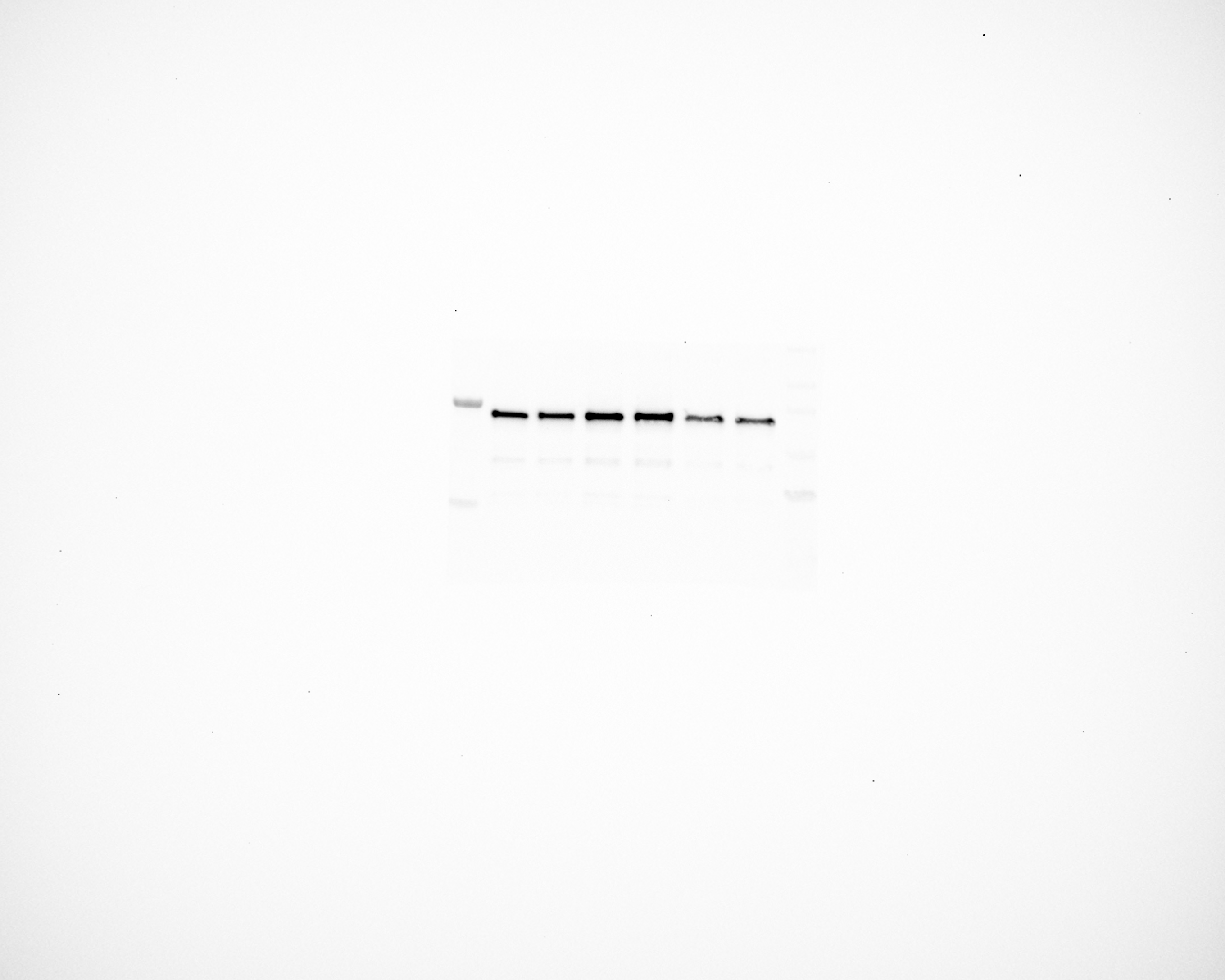

Supplement: Figure 1—figure supplement 2—source data 1. [file elife-85902-fig1-figsupp2-data1.zip › Figure 1-figure supplement 2-source data_/Unlabelled/B PKR.tif]

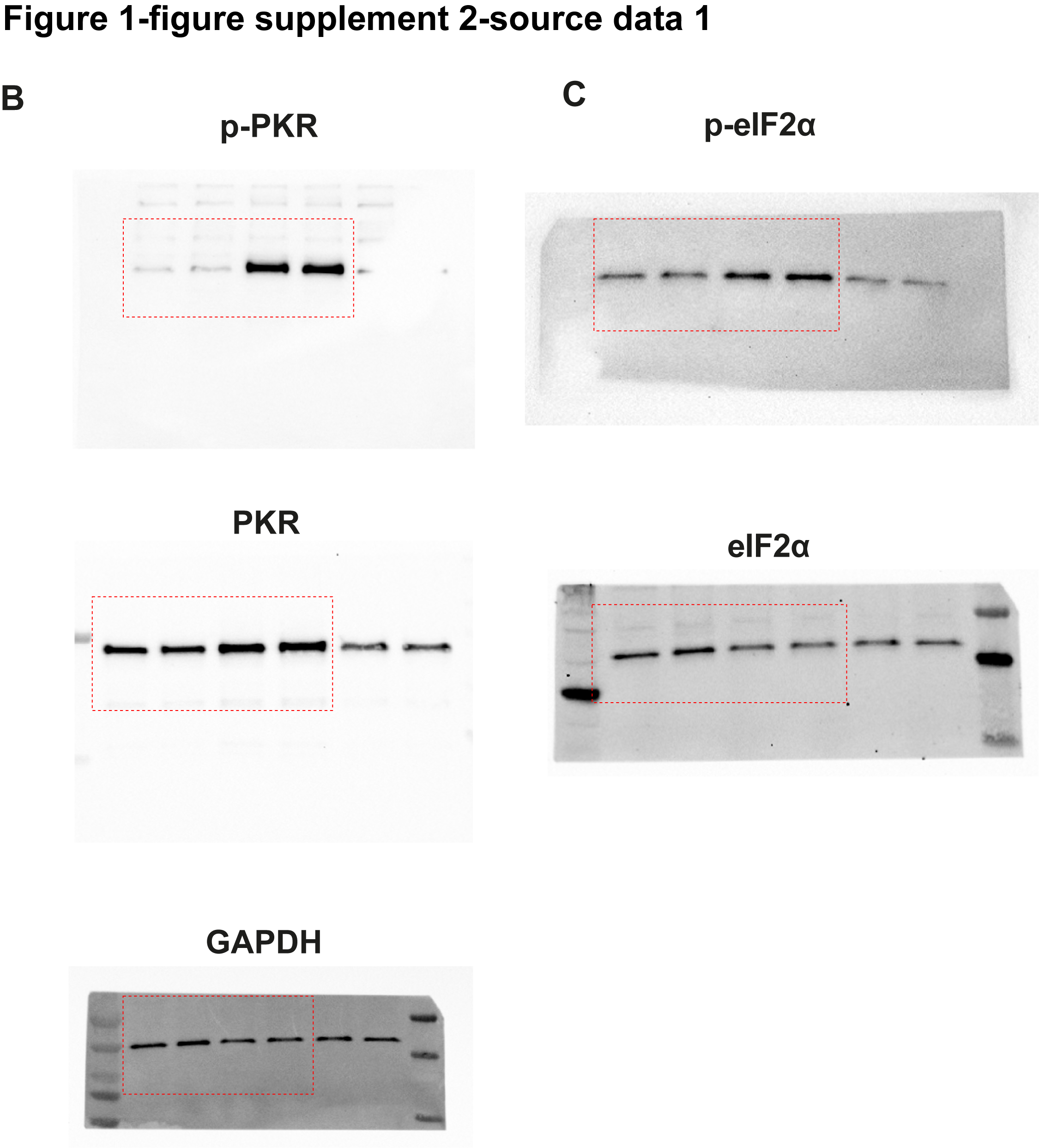

Supplement: Figure 1—figure supplement 2—source data 1. [file elife-85902-fig1-figsupp2-data1.zip › Figure 1-figure supplement 2-source data_/Labelled/Figure 1-figure supplement 2-source data 1.tif]

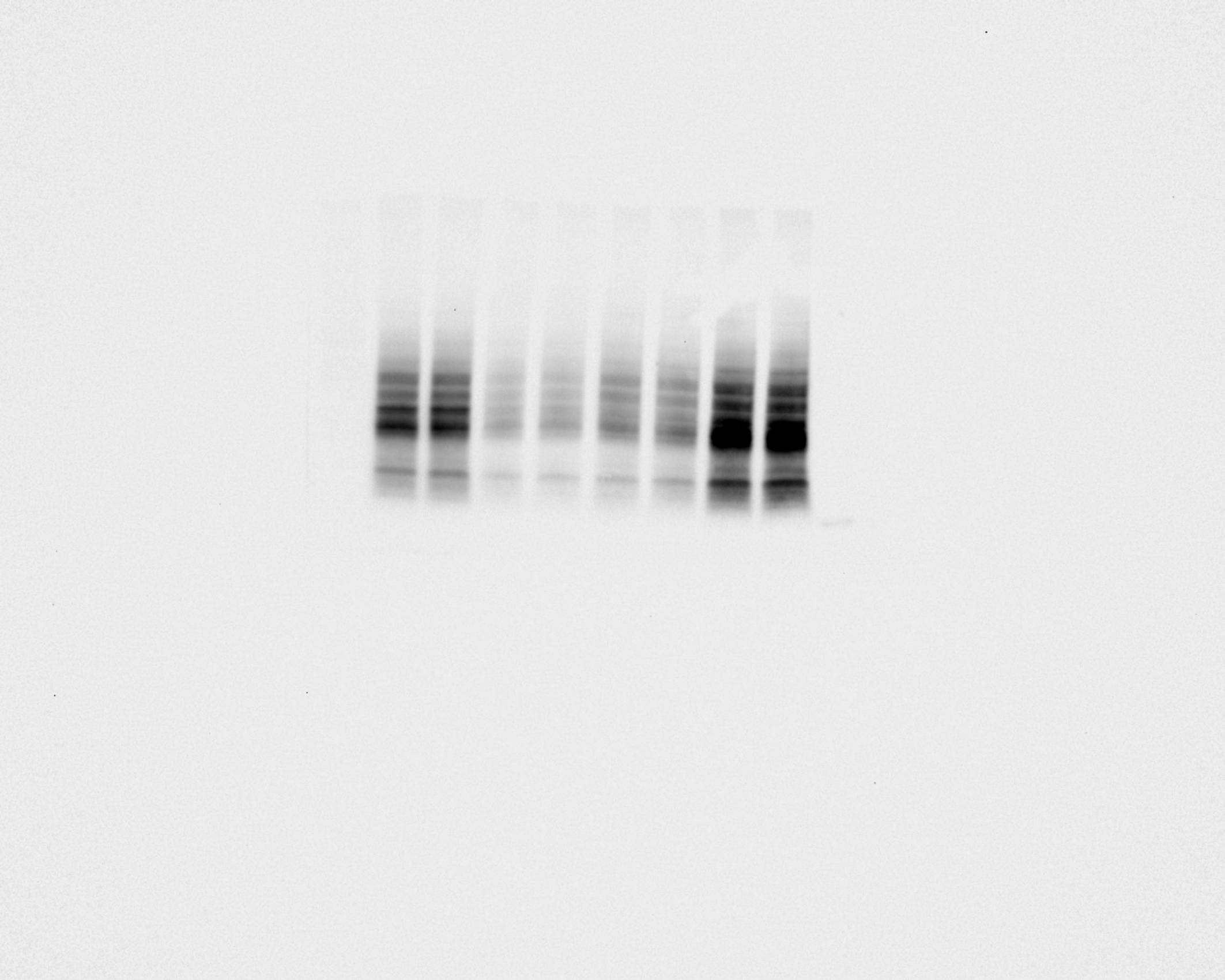

Supplement: Figure 2—source data 1. [file elife-85902-fig2-data1.zip › Figure 2-source data/Unlabelled/2A PUROMYCIN.tif]

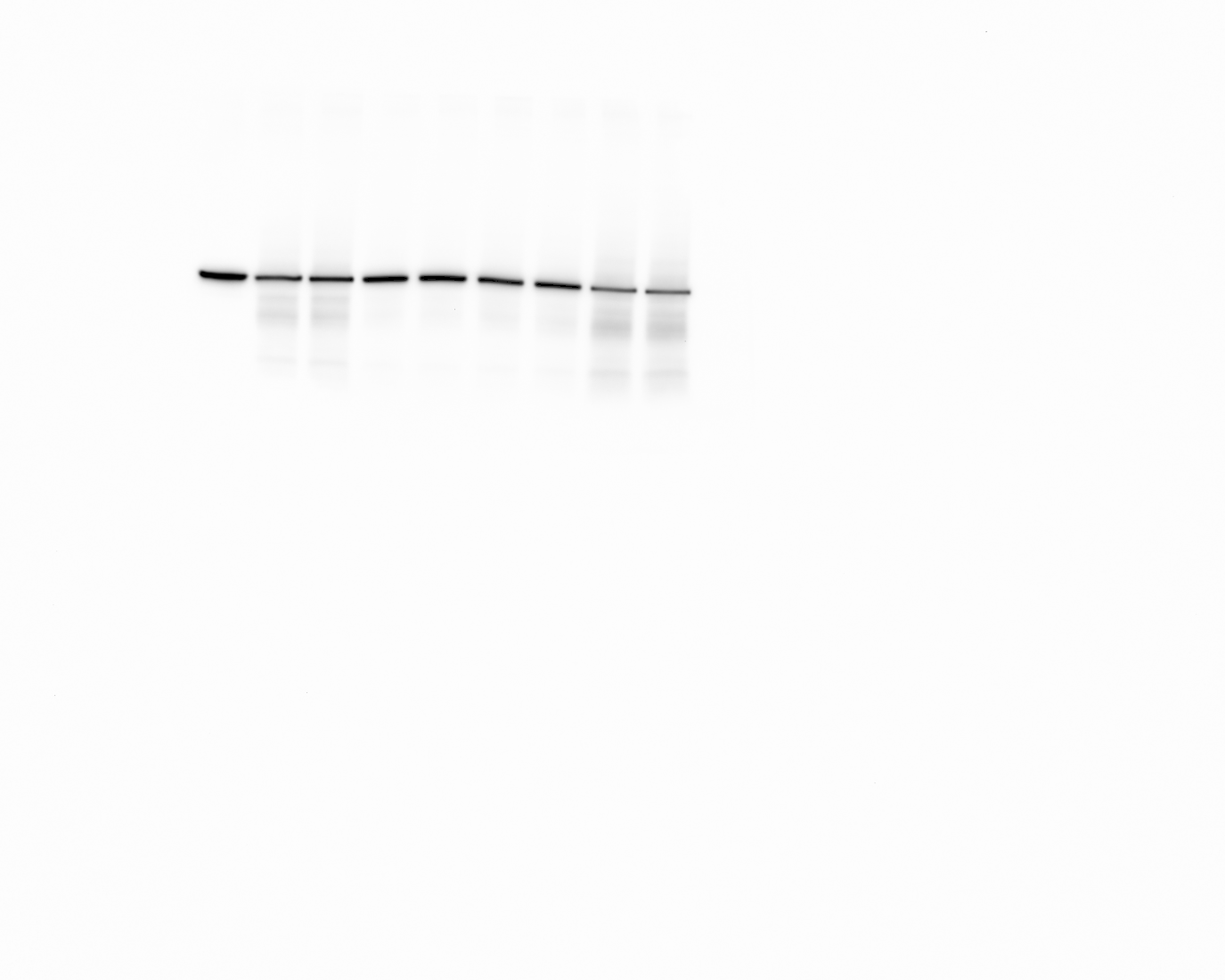

Supplement: Figure 2—source data 1. [file elife-85902-fig2-data1.zip › Figure 2-source data/Unlabelled/2A GAPDH.tif]

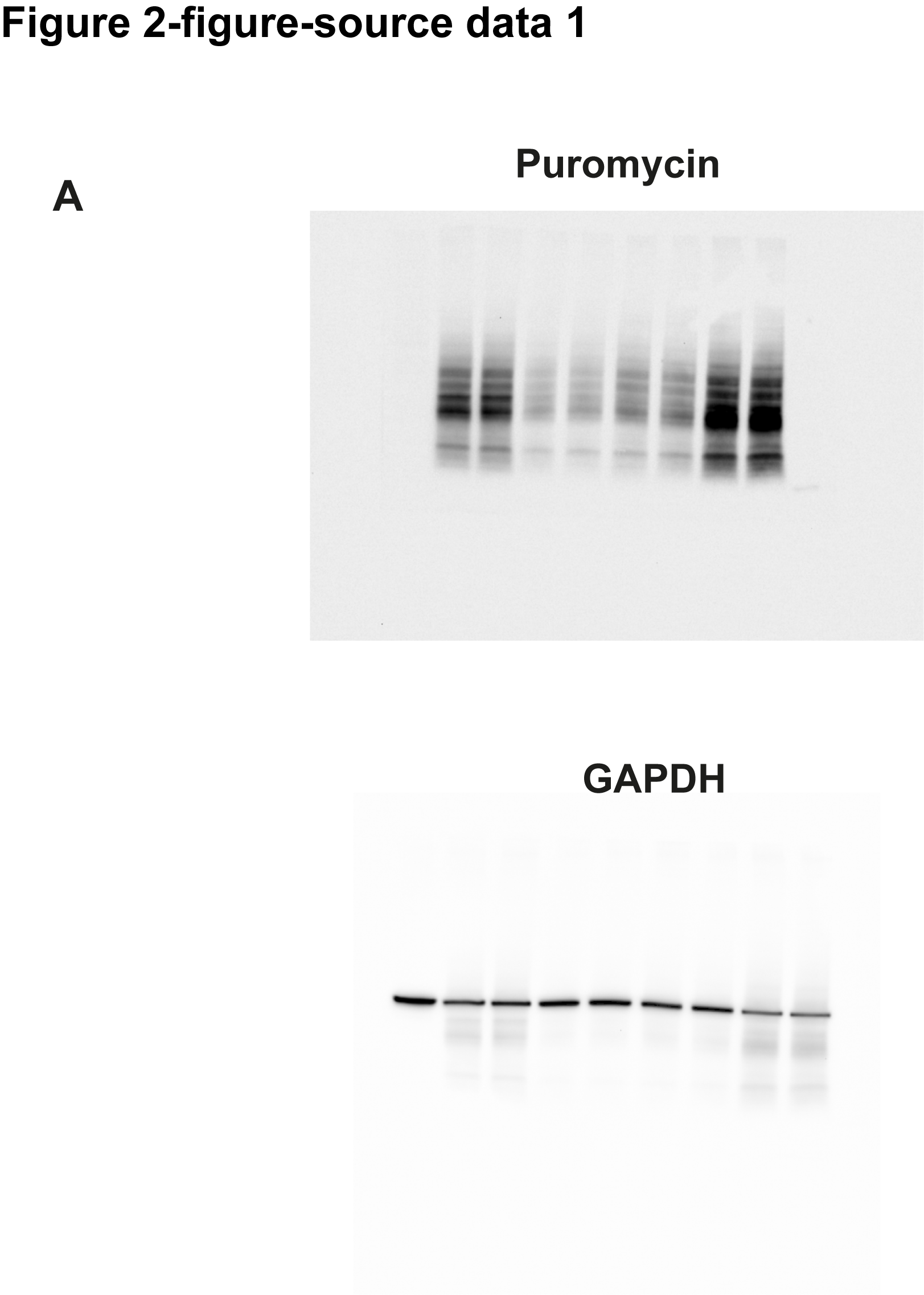

Supplement: Figure 2—source data 1. [file elife-85902-fig2-data1.zip › Figure 2-source data/Labelled/Figure 2-source data 1.tif]

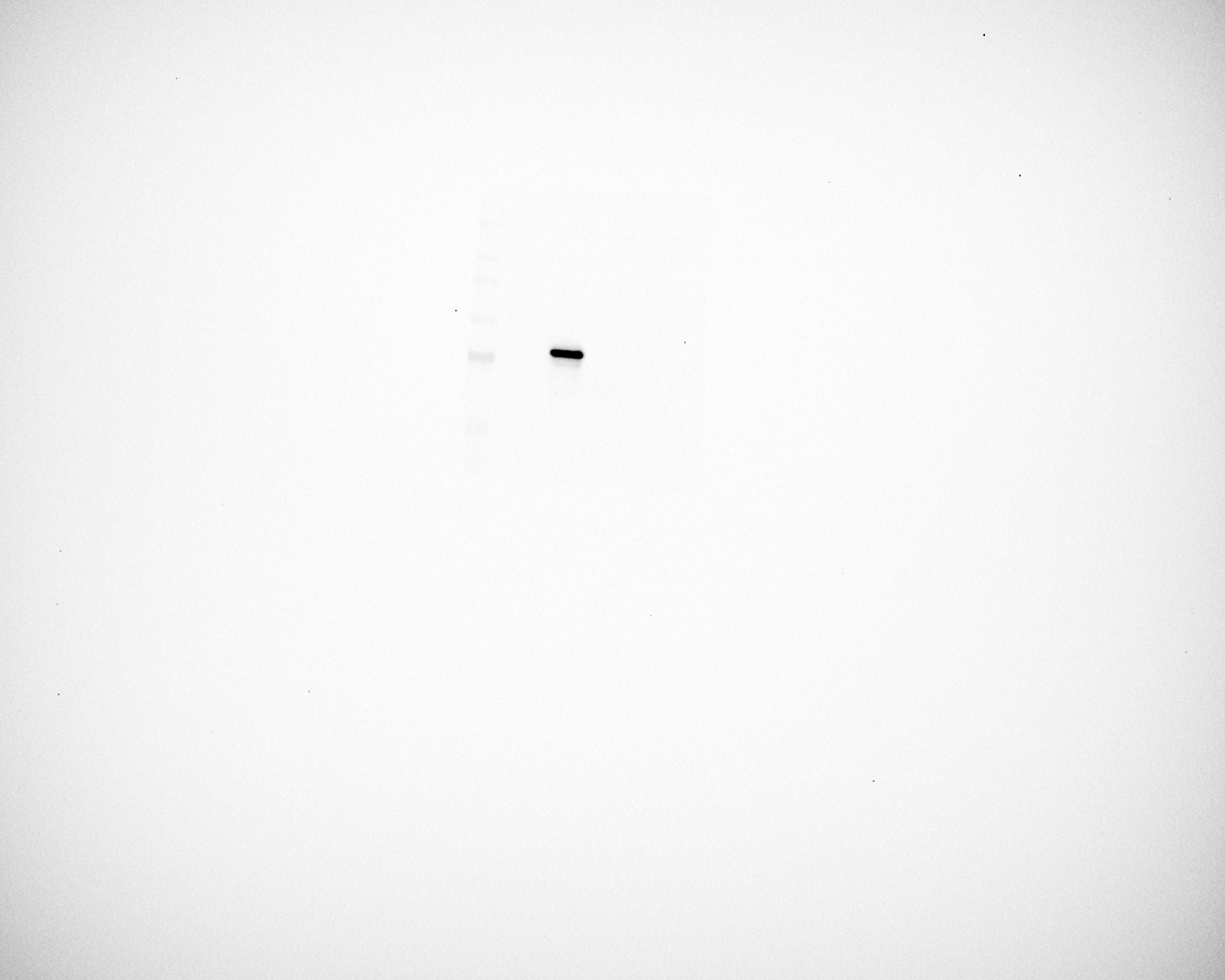

Supplement: Figure 3—source data 1. [file elife-85902-fig3-data1.zip › Figure 3-source data/Unlabelled/3B GP.tif]

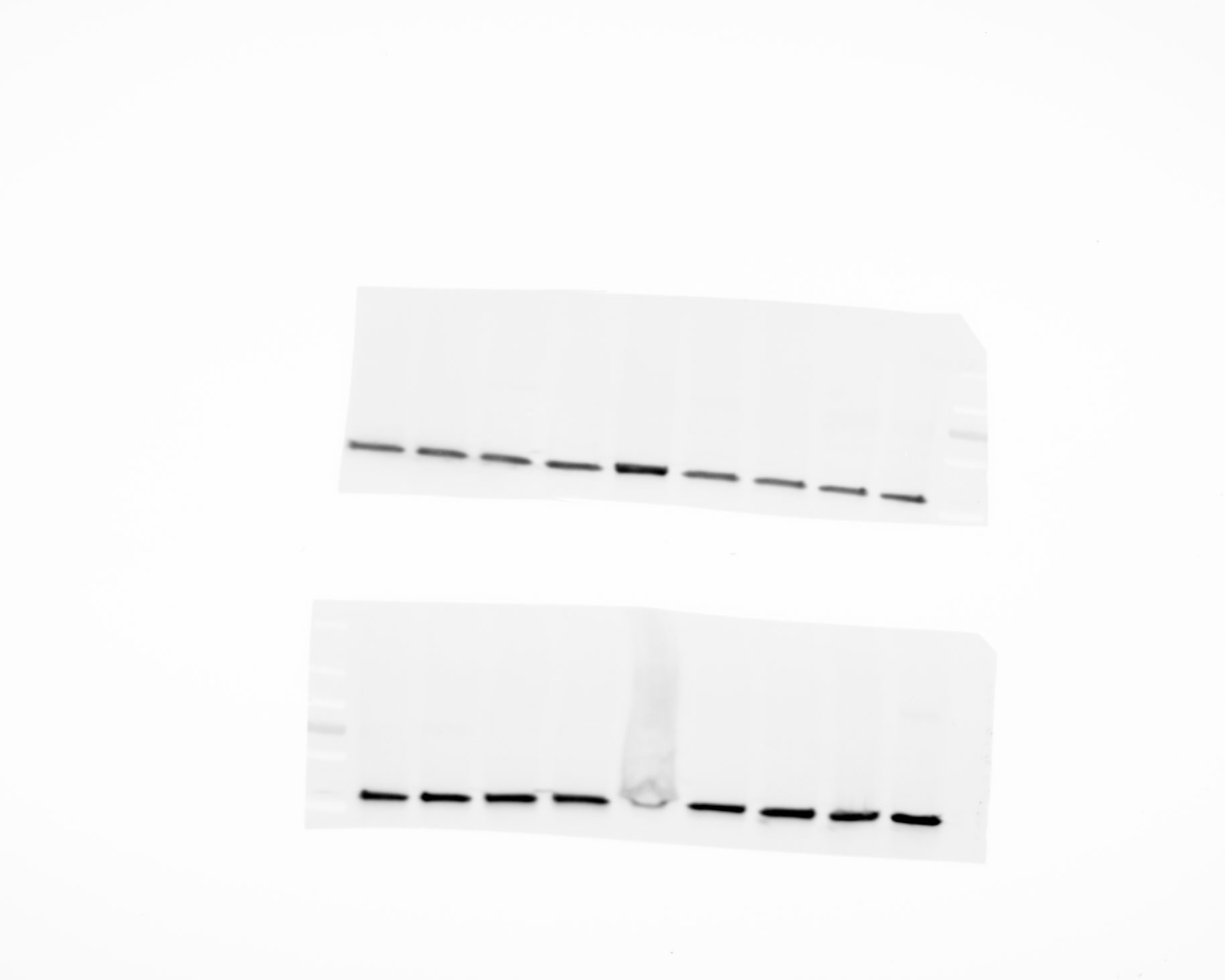

Supplement: Figure 3—source data 1. [file elife-85902-fig3-data1.zip › Figure 3-source data/Unlabelled/GAPDH 3D.tif]

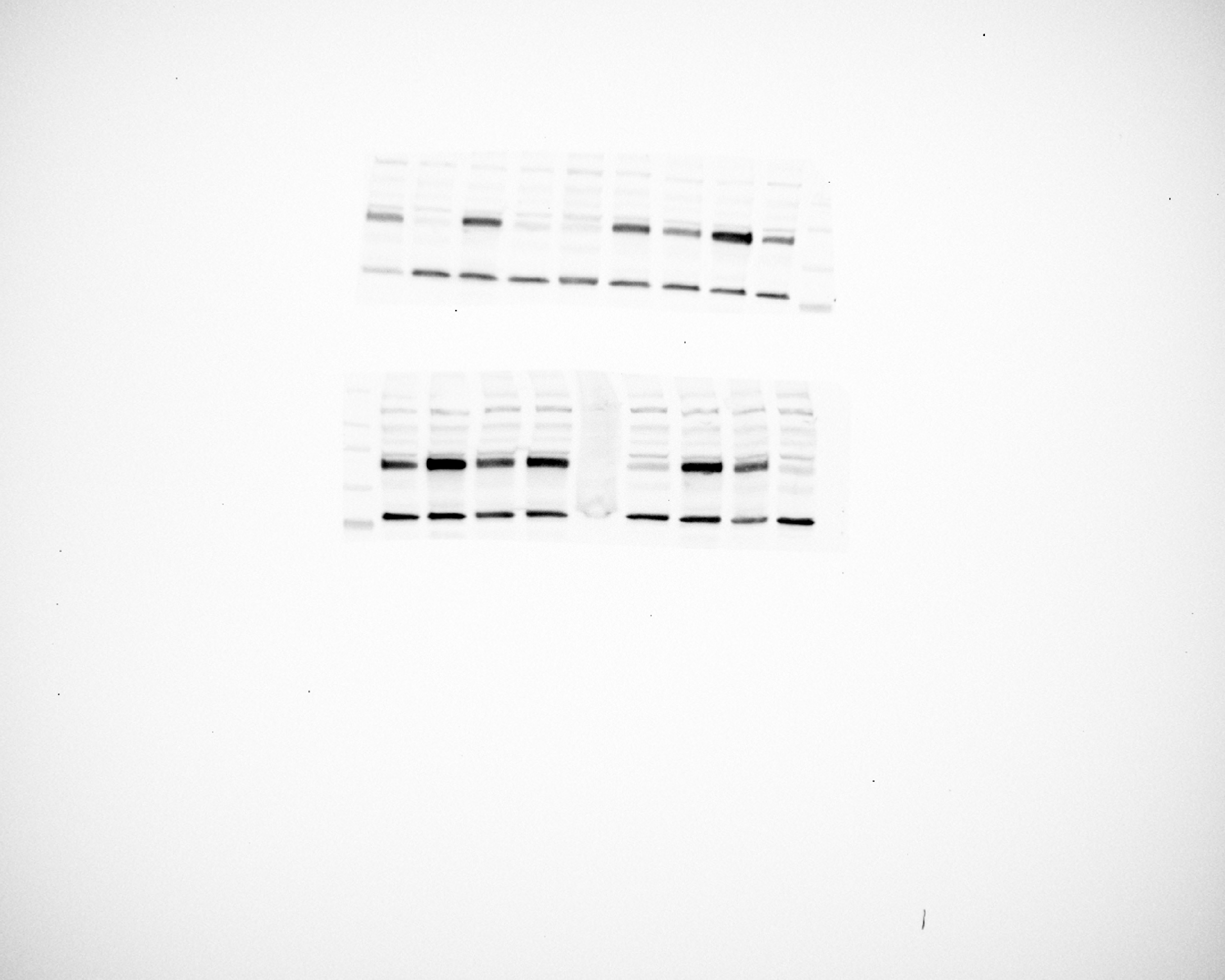

Supplement: Figure 3—source data 1. [file elife-85902-fig3-data1.zip › Figure 3-source data/Unlabelled/EIF2A 3D.tif]

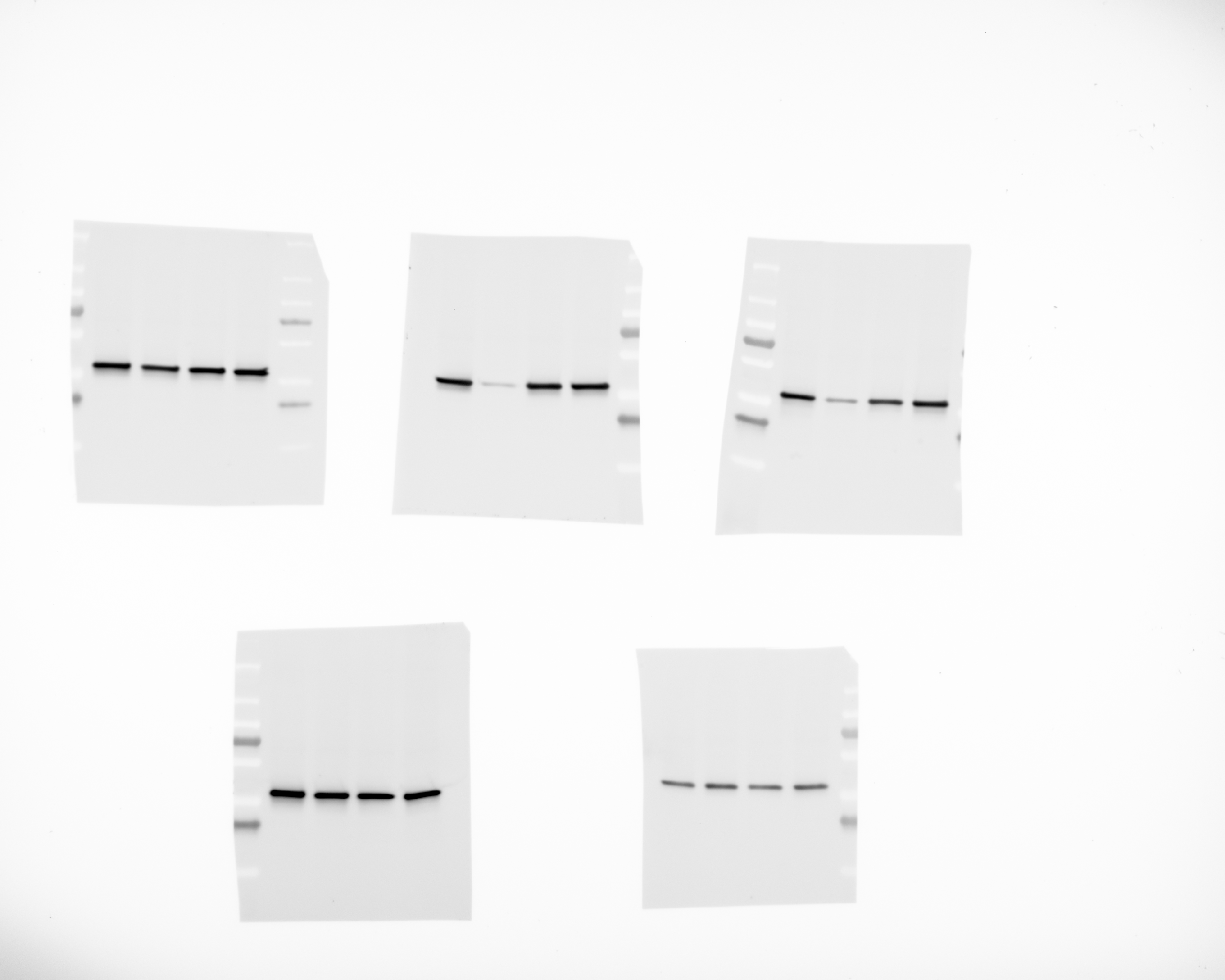

Supplement: Figure 3—source data 1. [file elife-85902-fig3-data1.zip › Figure 3-source data/Unlabelled/3B GAPDH.tif]

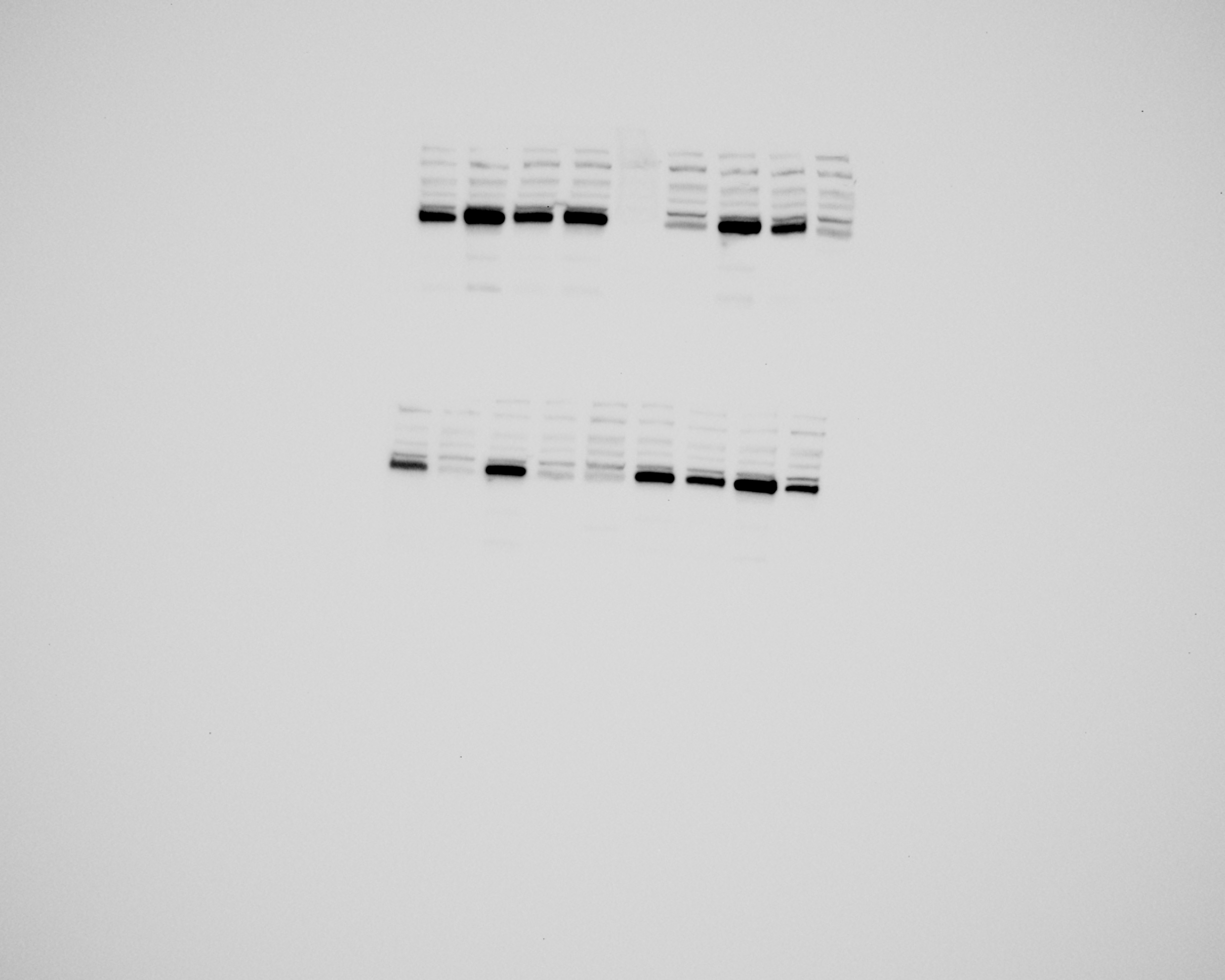

Supplement: Figure 3—source data 1. [file elife-85902-fig3-data1.zip › Figure 3-source data/Unlabelled/pPKR 3D.tif]

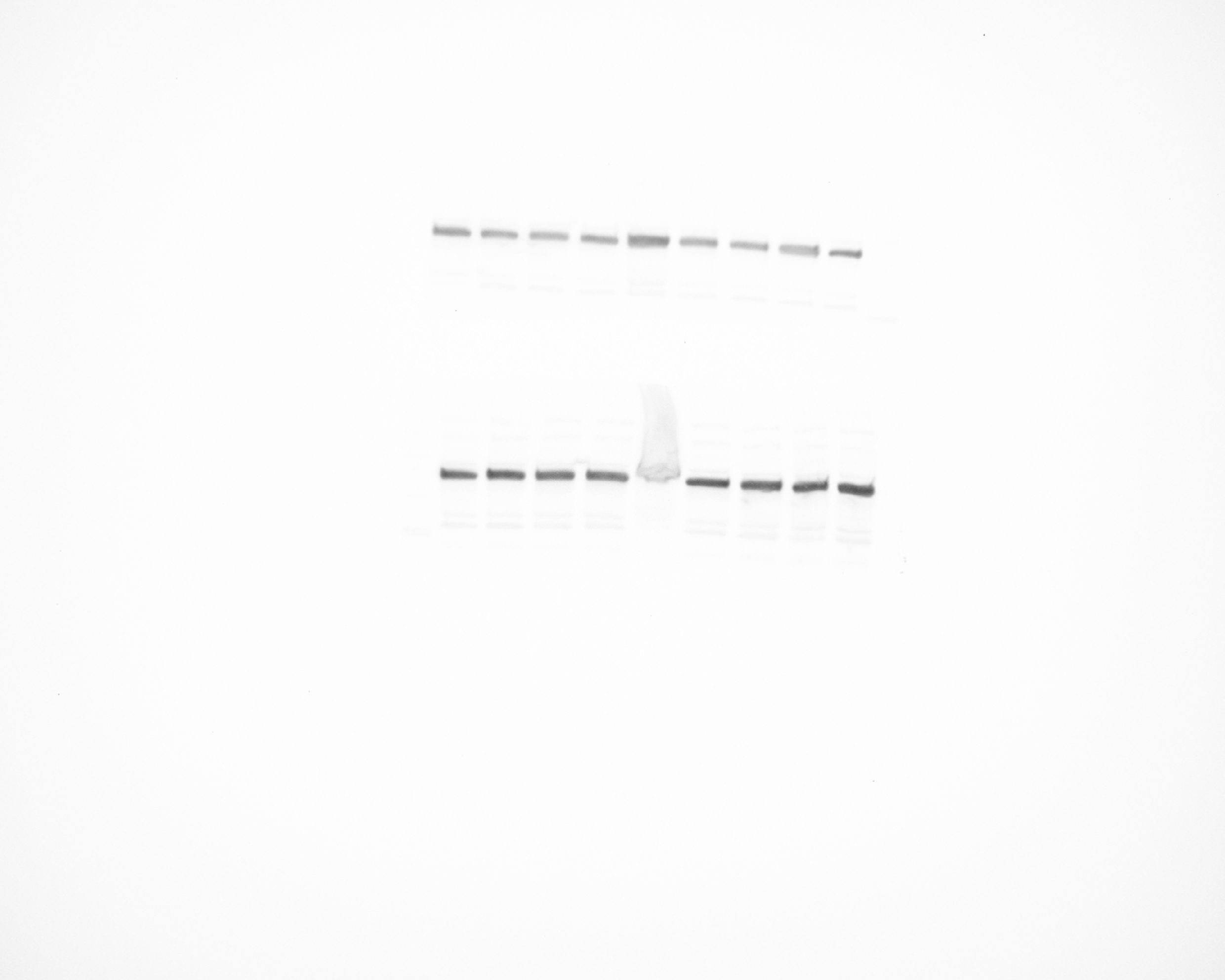

Supplement: Figure 3—source data 1. [file elife-85902-fig3-data1.zip › Figure 3-source data/Unlabelled/PKR 3D.tif]

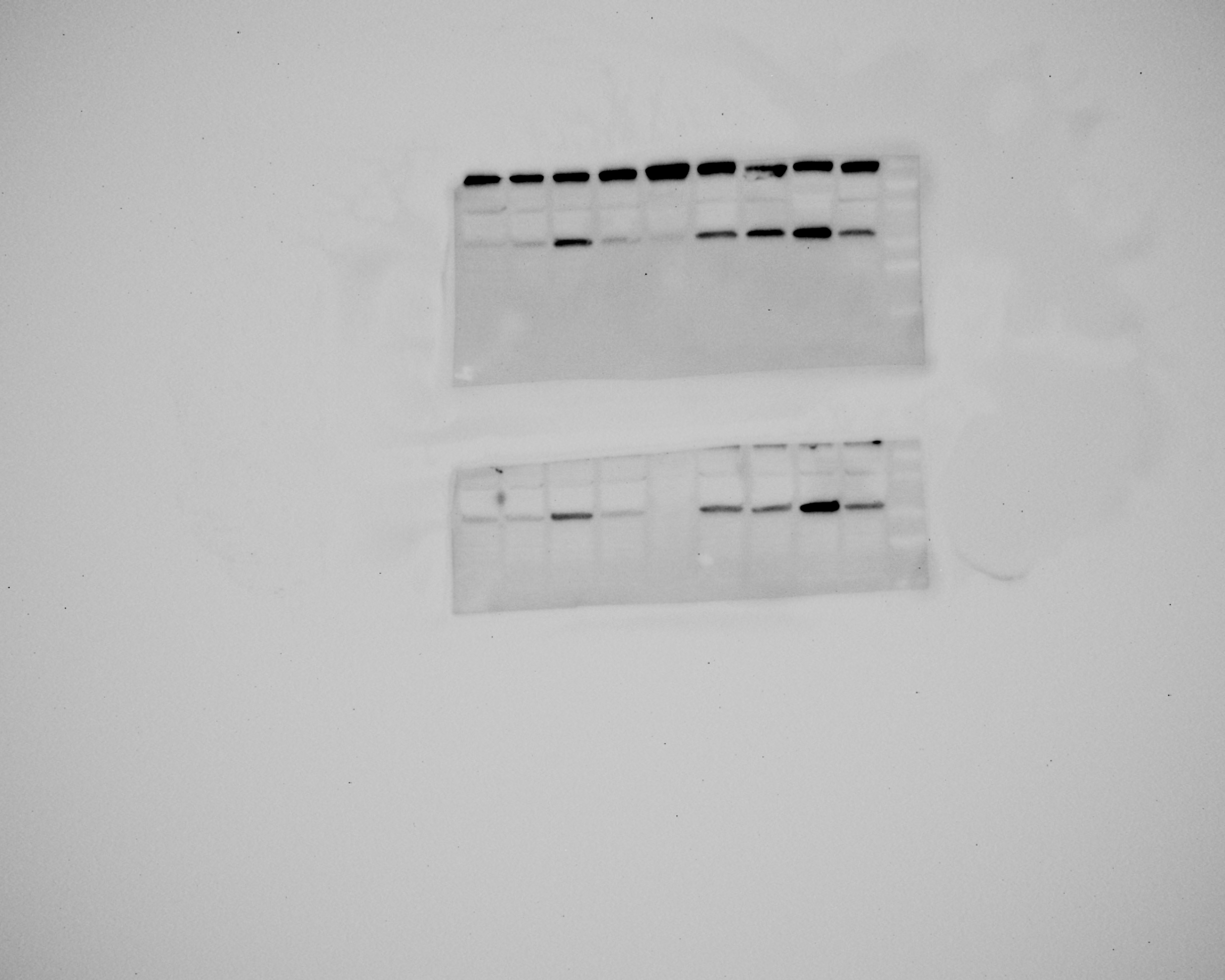

Supplement: Figure 3—source data 1. [file elife-85902-fig3-data1.zip › Figure 3-source data/Unlabelled/pEIF2A 3D.tif]

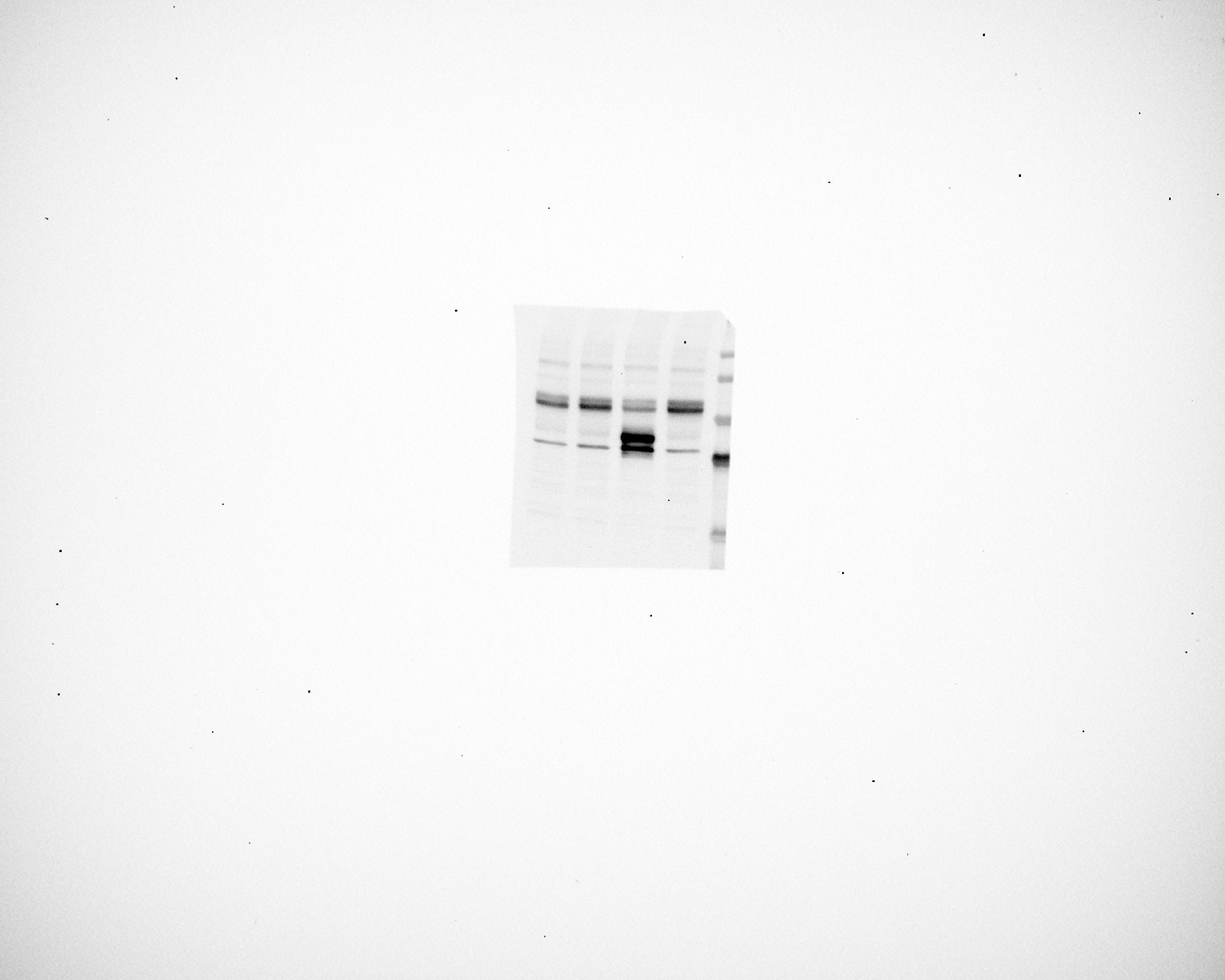

Supplement: Figure 3—source data 1. [file elife-85902-fig3-data1.zip › Figure 3-source data/Unlabelled/3B PR.tif]

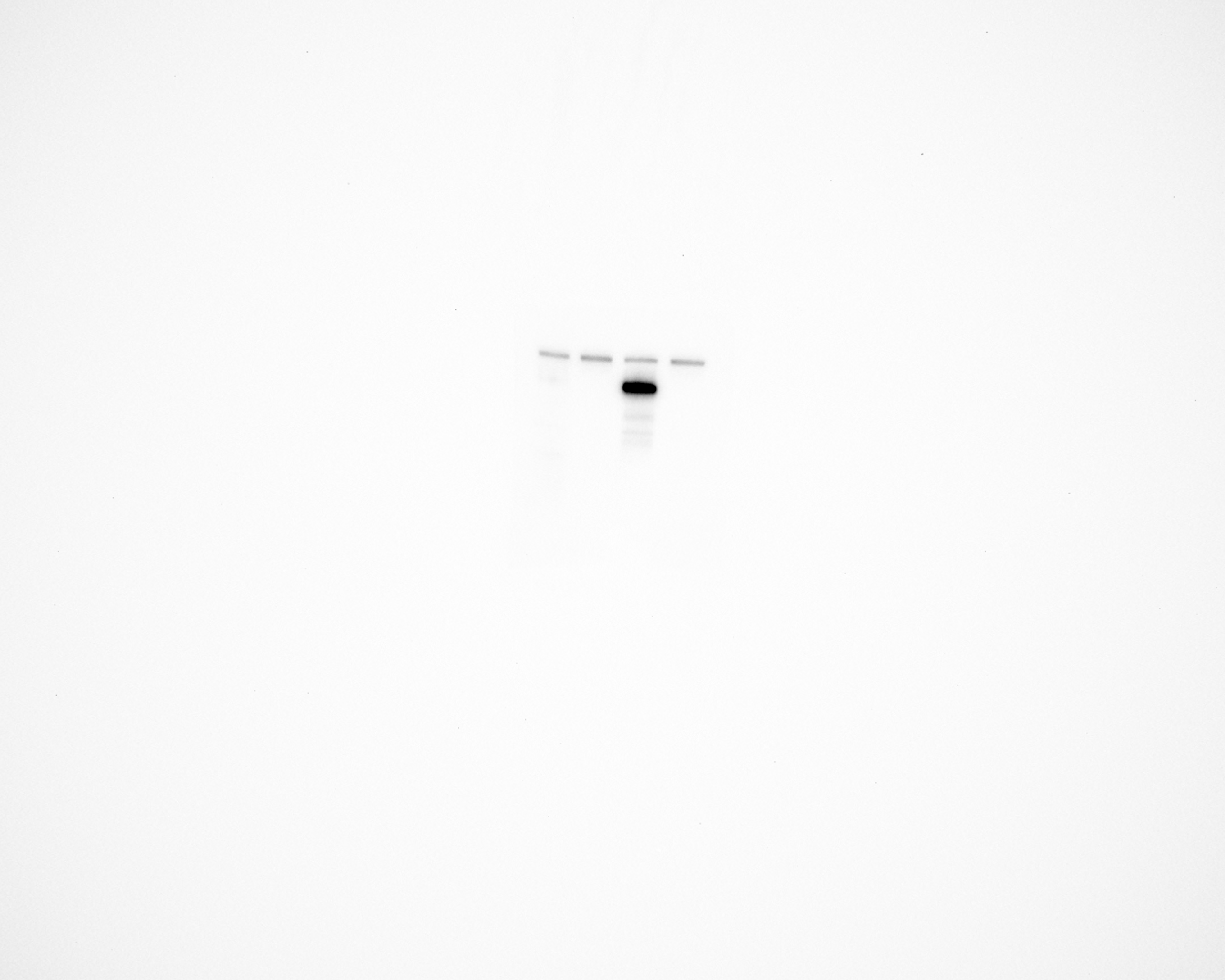

Supplement: Figure 3—source data 1. [file elife-85902-fig3-data1.zip › Figure 3-source data/Unlabelled/3B PA.tif]

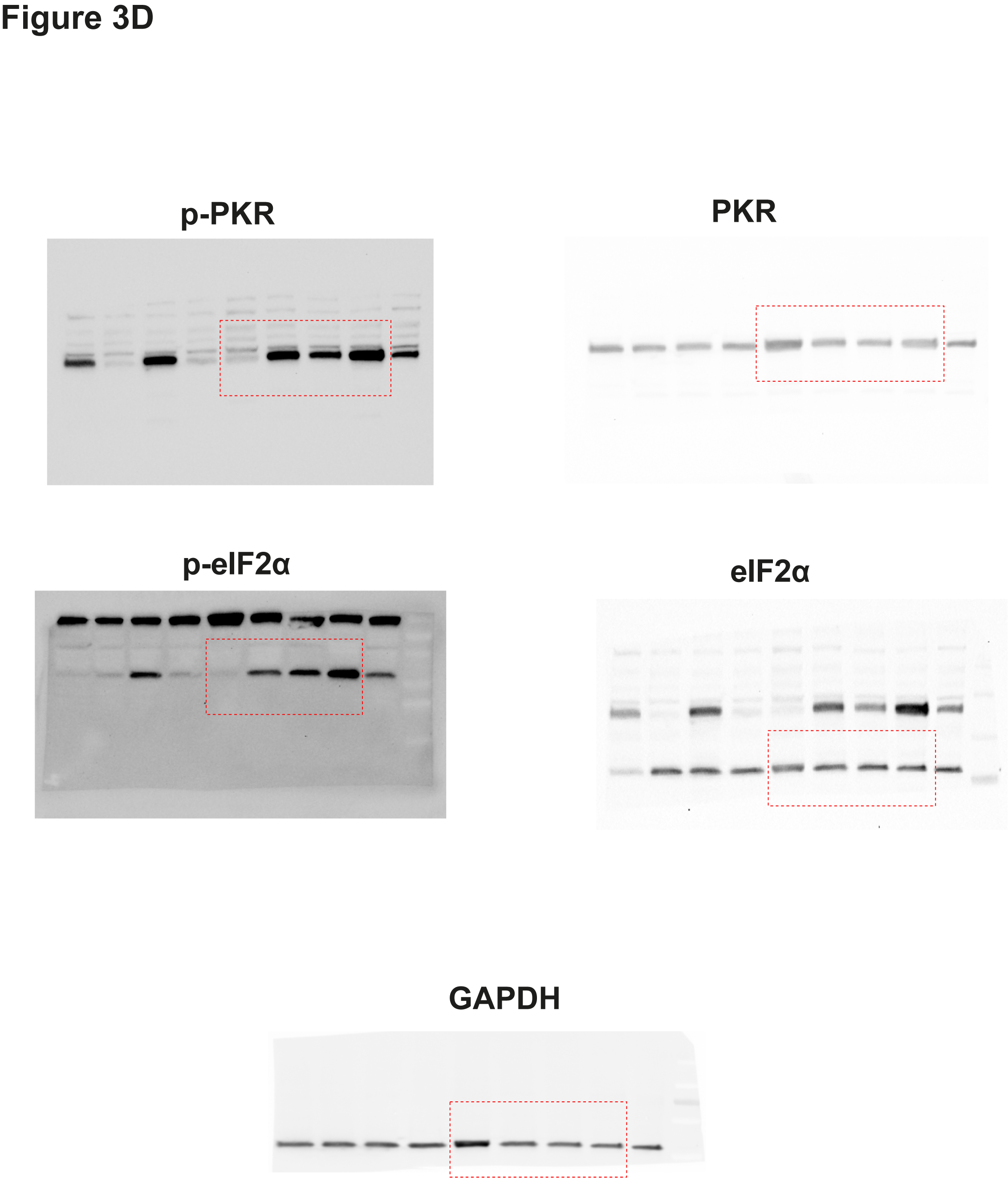

Supplement: Figure 3—source data 1. [file elife-85902-fig3-data1.zip › Figure 3-source data/Labelled/Figure 3-source data 2.tif]

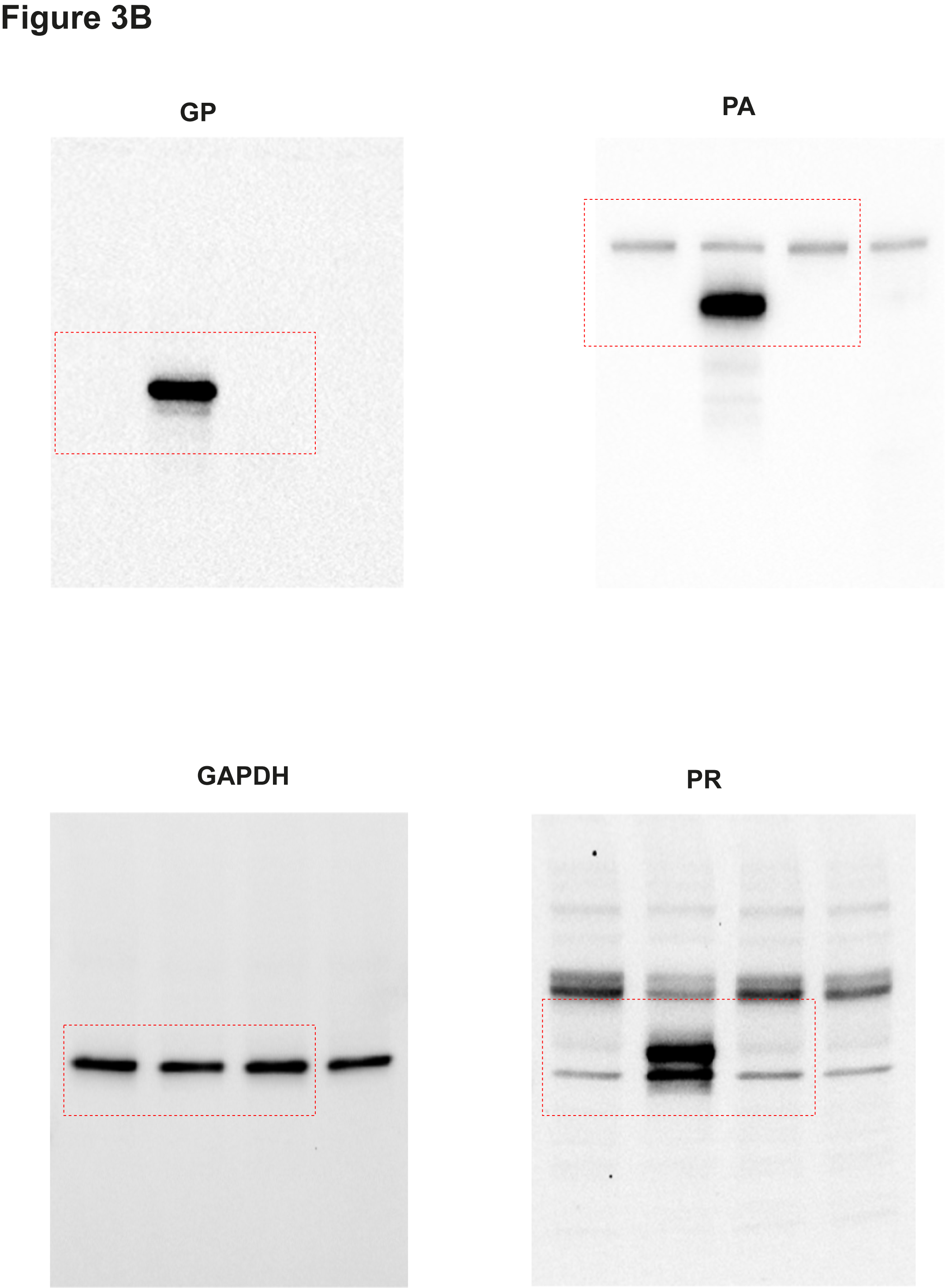

Supplement: Figure 3—source data 1. [file elife-85902-fig3-data1.zip › Figure 3-source data/Labelled/Figure 3-source data 1.tif]

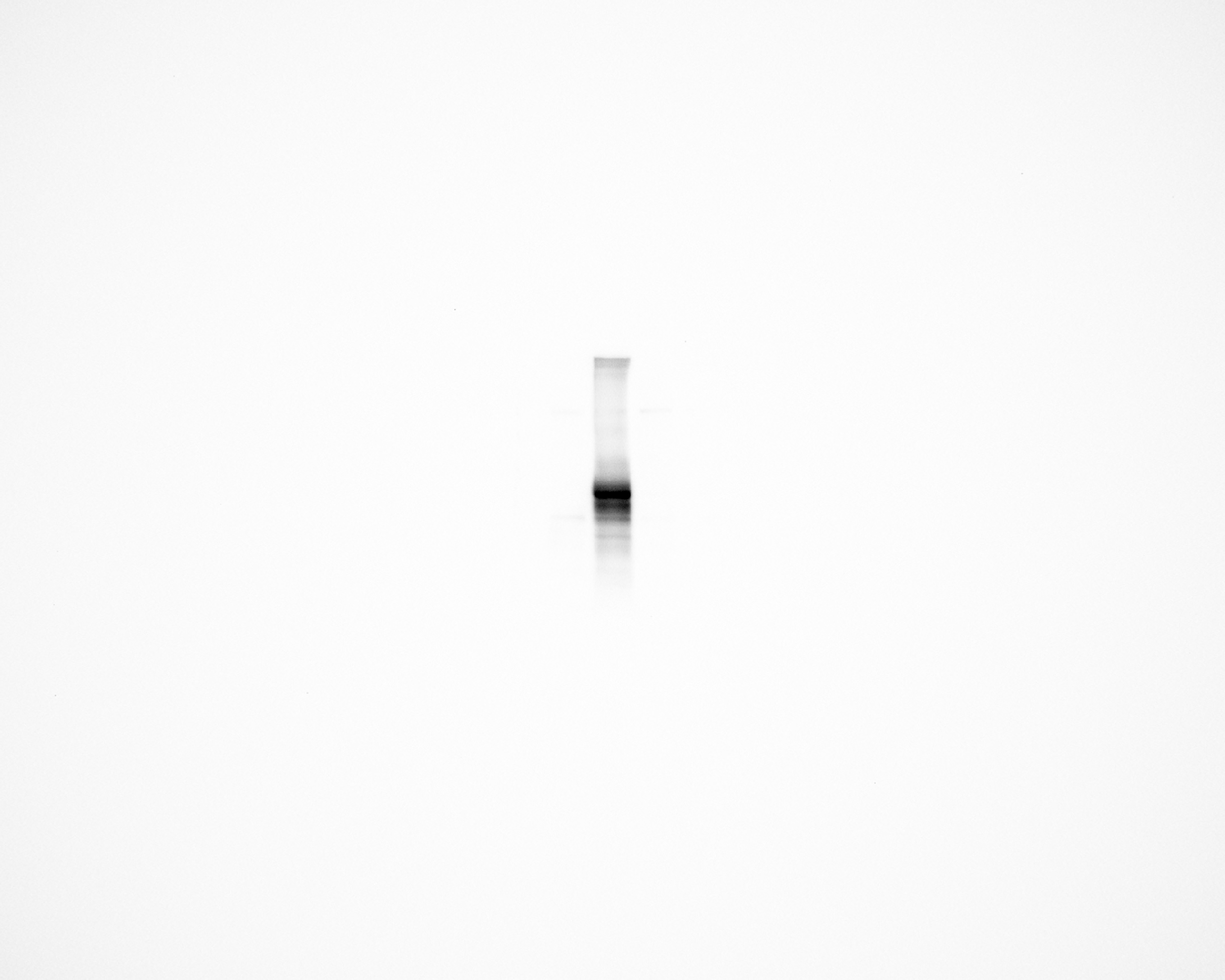

Supplement: Figure 3—source data 2. [file elife-85902-fig3-data2.zip › Figure 3-figure supplement 1-source data_/Unlabelled/D PR.tif]

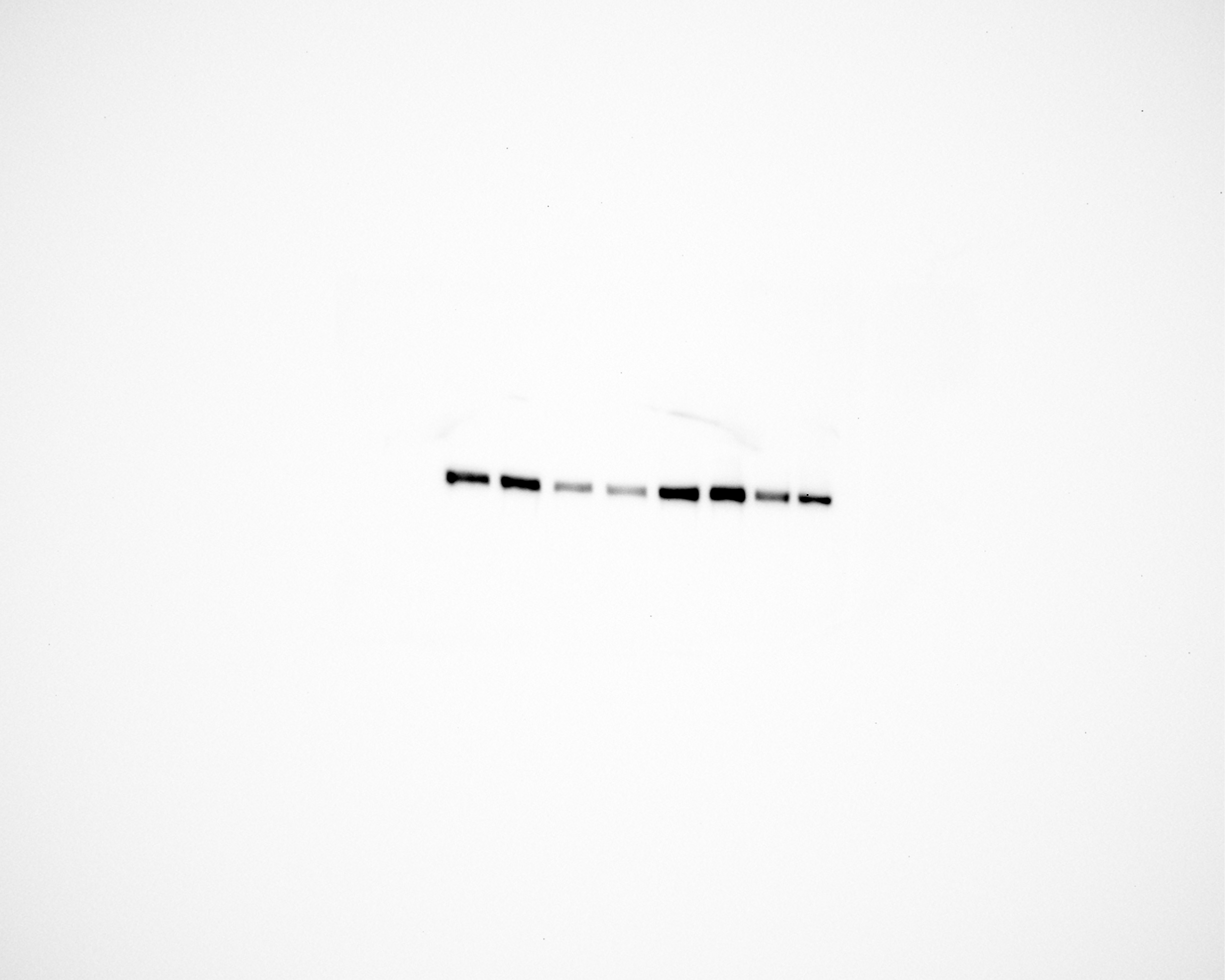

Supplement: Figure 3—source data 2. [file elife-85902-fig3-data2.zip › Figure 3-figure supplement 1-source data_/Unlabelled/F PKR.tif]

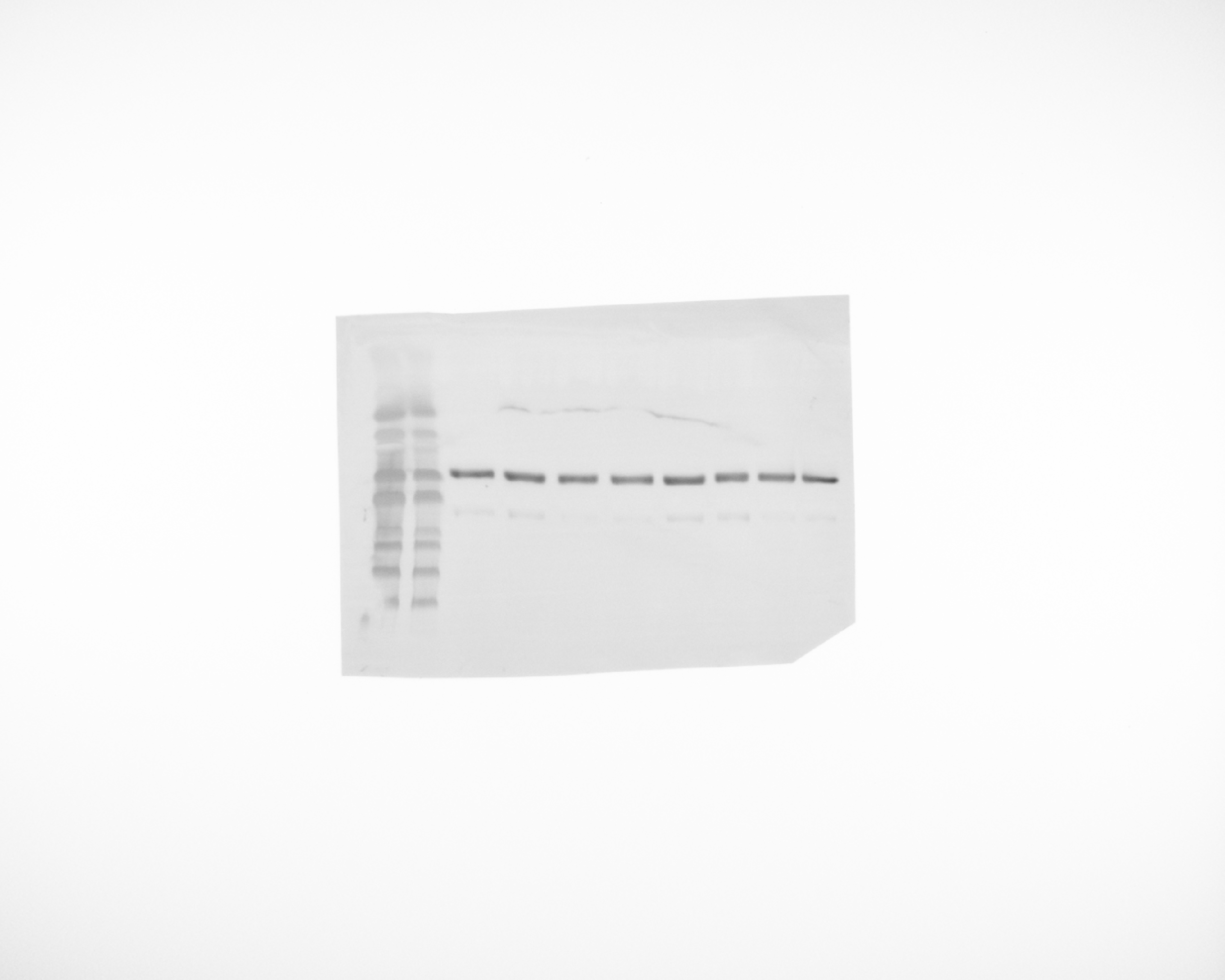

Supplement: Figure 3—source data 2. [file elife-85902-fig3-data2.zip › Figure 3-figure supplement 1-source data_/Unlabelled/F ACTIN.tif]

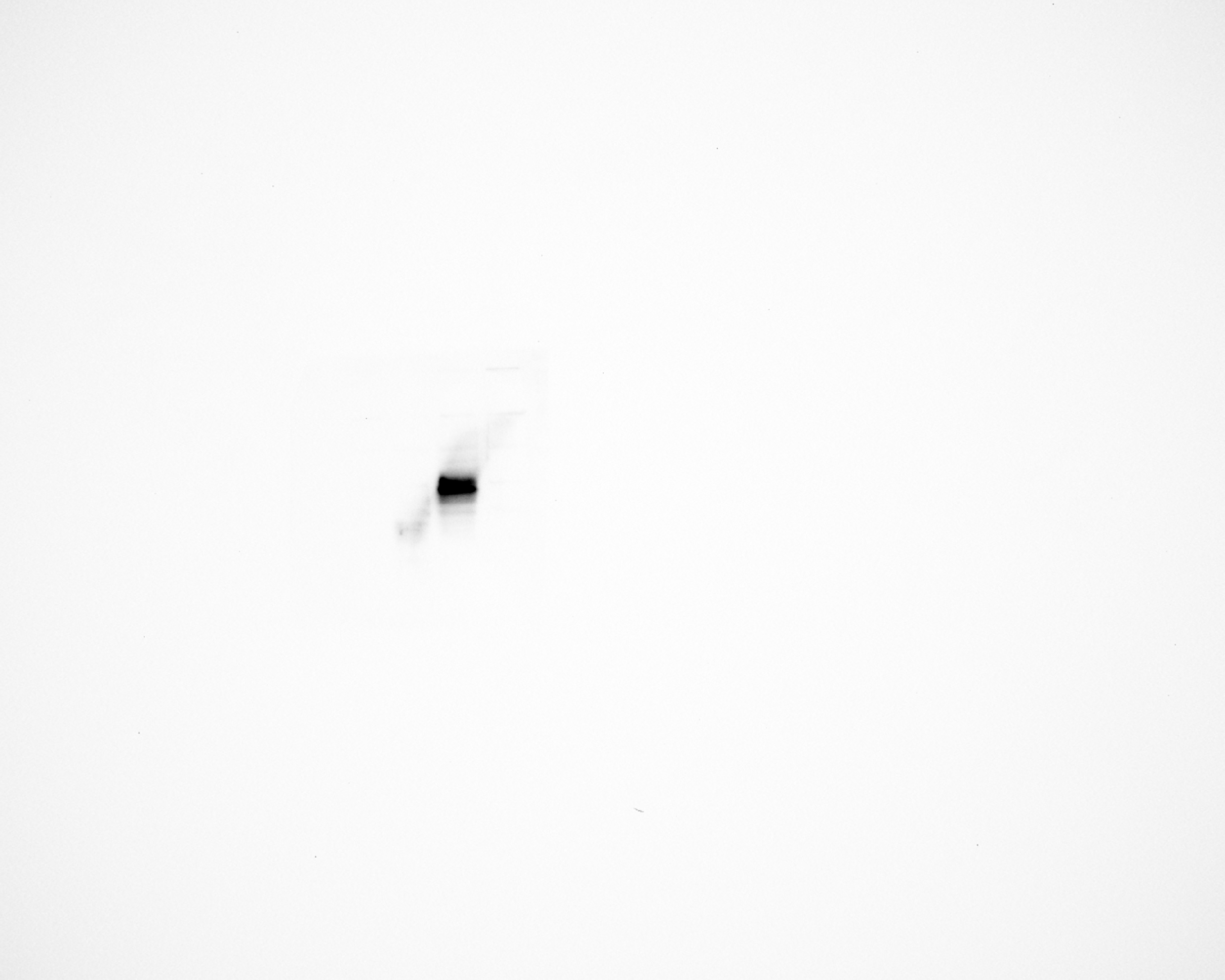

Supplement: Figure 3—source data 2. [file elife-85902-fig3-data2.zip › Figure 3-figure supplement 1-source data_/Unlabelled/D GP.tif]

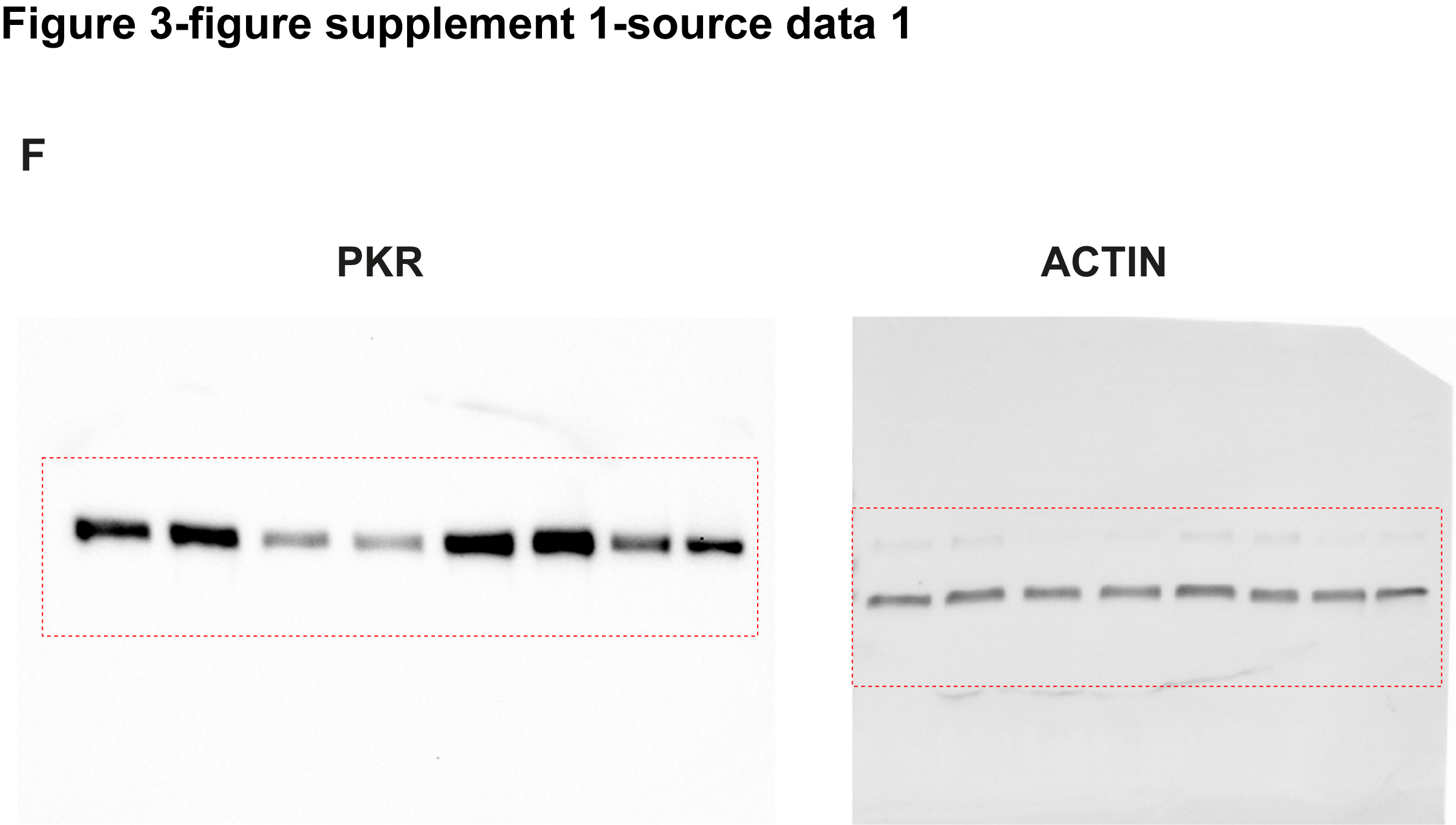

Supplement: Figure 3—source data 2. [file elife-85902-fig3-data2.zip › Figure 3-figure supplement 1-source data_/Labelled/Figure 3-figure supplement 1-source data 2.tif]

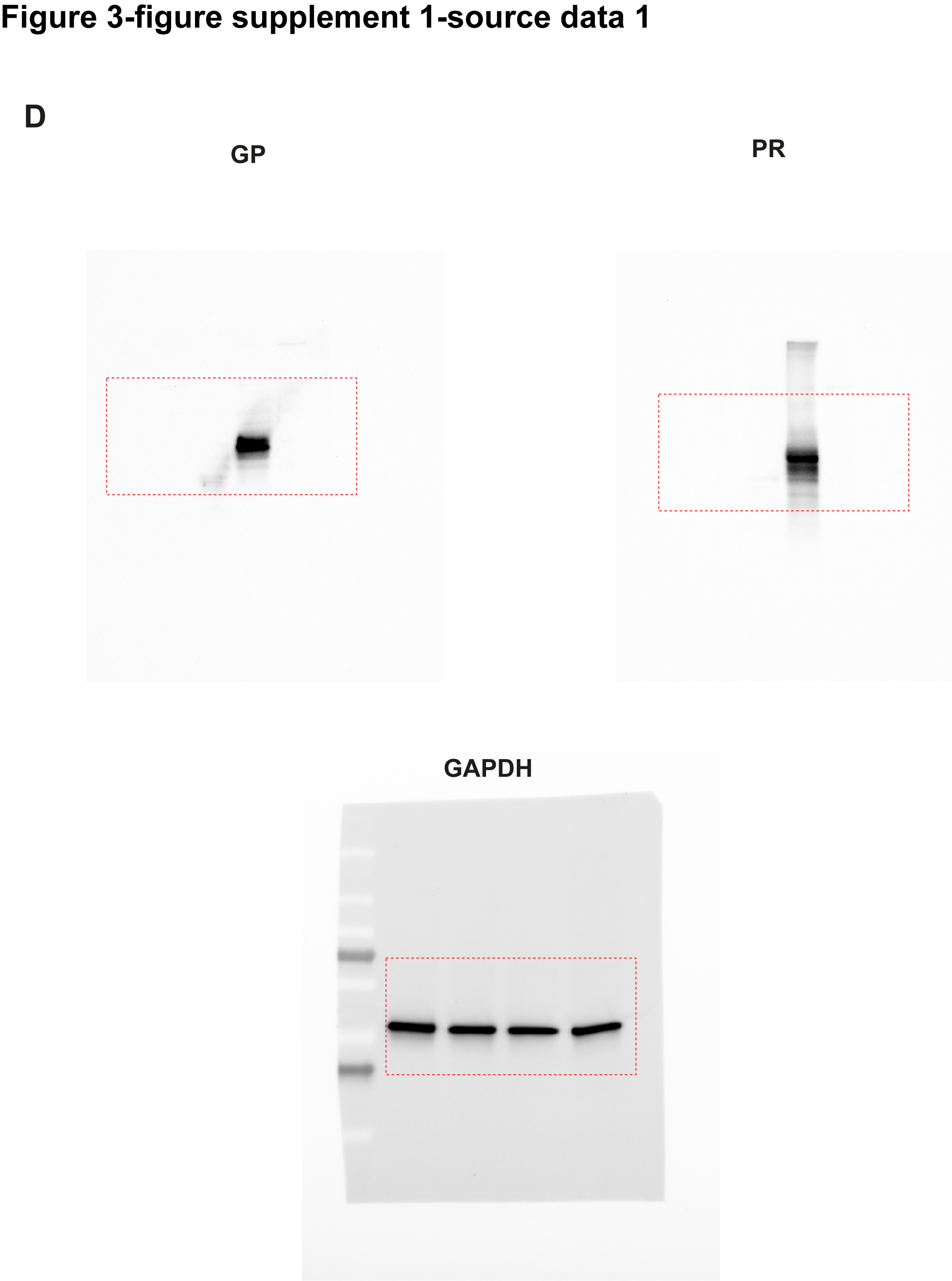

Supplement: Figure 3—source data 2. [file elife-85902-fig3-data2.zip › Figure 3-figure supplement 1-source data_/Labelled/Figure 3-figure supplement 1-source data 1.tif]

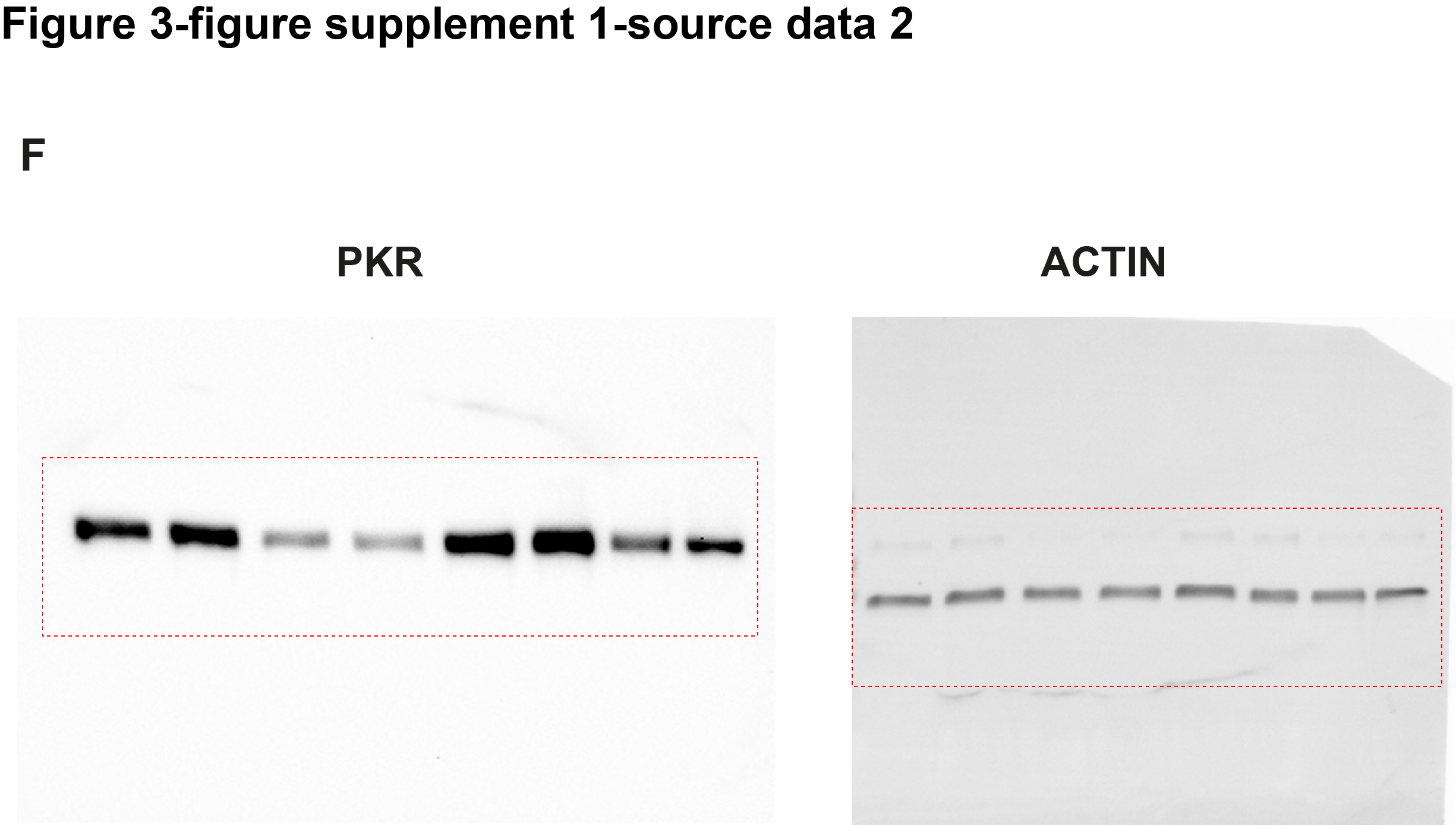

Supplement: Figure 3—figure supplement 1—source data 2. [file elife-85902-fig3-figsupp1-data2.zip › Figure 3-figure supplement 1-source data 2_ /Labelled/Figure 3-figure supplement 1-source data 2.png]

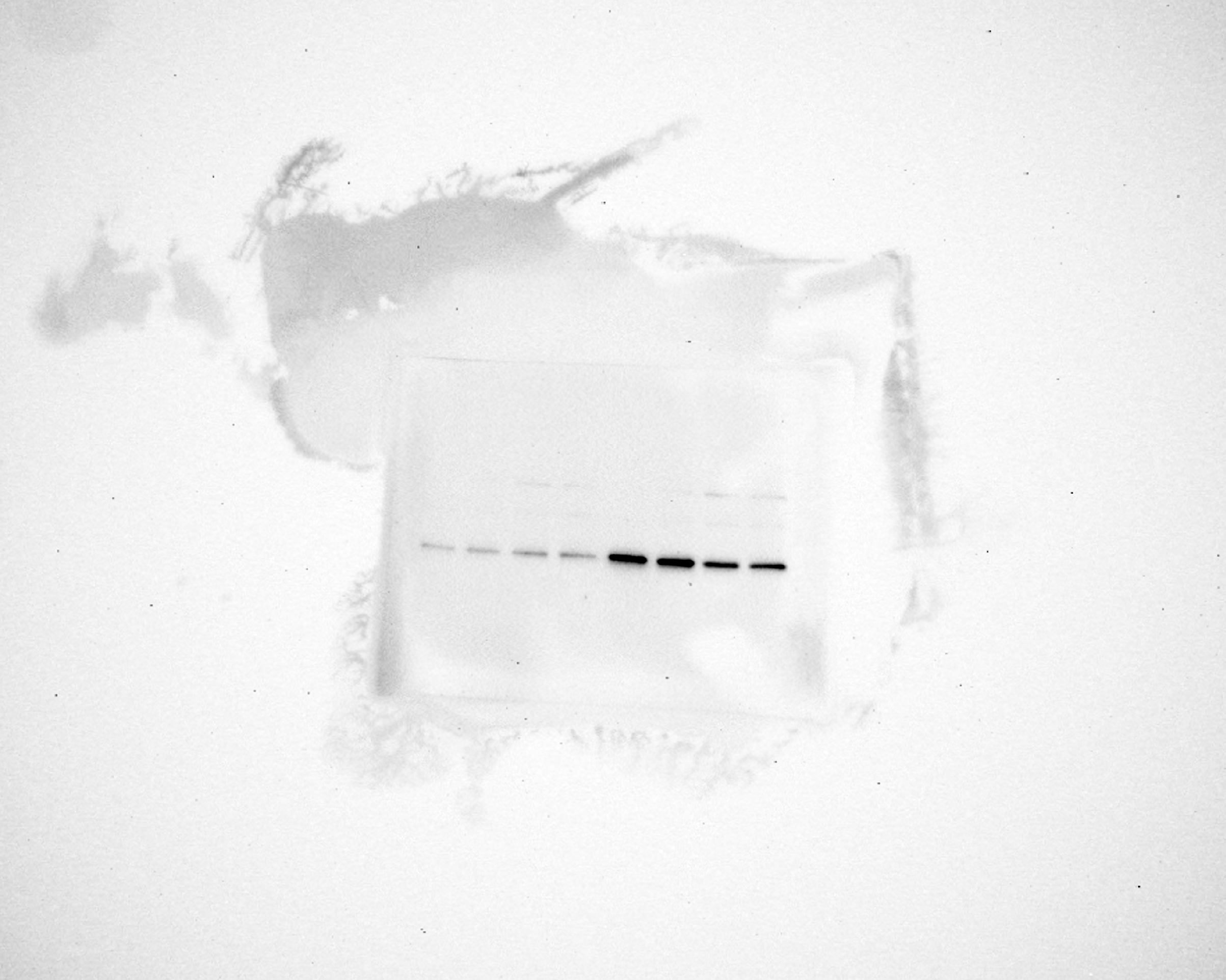

Supplement: Figure 4—source data 1. [file elife-85902-fig4-data1.zip › Figure 4-source data/Unlabelled/4D Peif2a.tif]

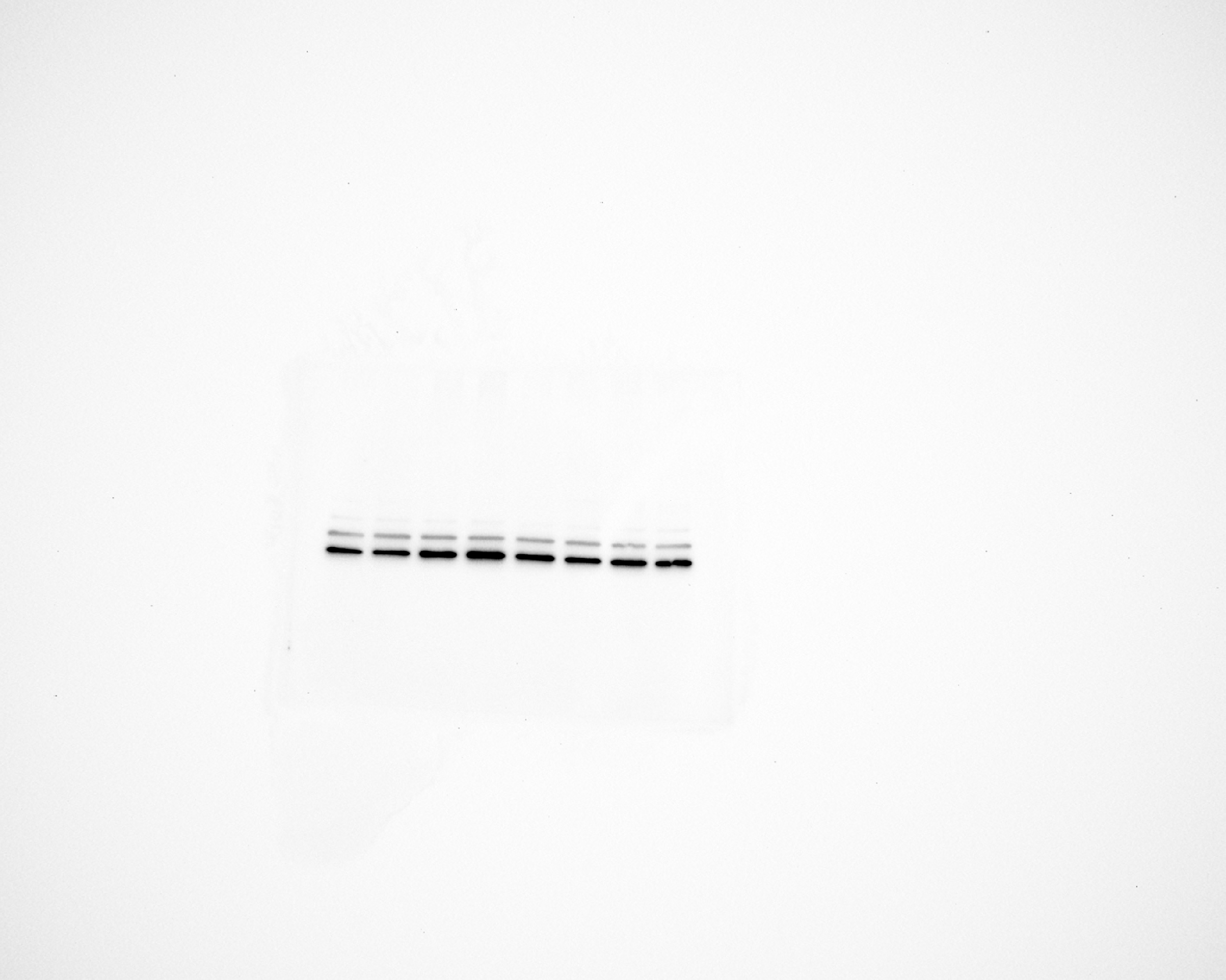

Supplement: Figure 4—source data 1. [file elife-85902-fig4-data1.zip › Figure 4-source data/Unlabelled/4D eif2a.tif]

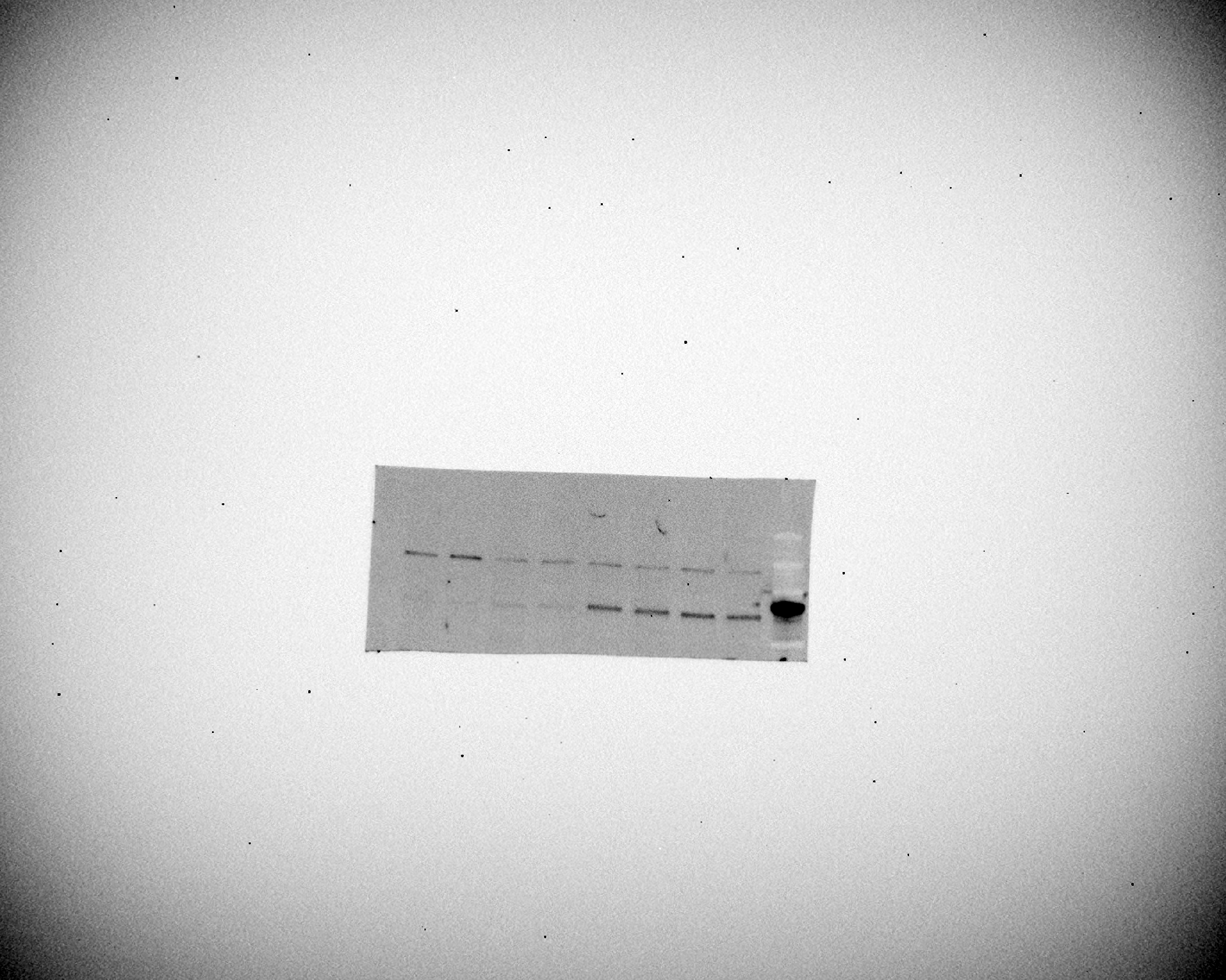

Supplement: Figure 4—source data 1. [file elife-85902-fig4-data1.zip › Figure 4-source data/Unlabelled/4D PKR.jpg]

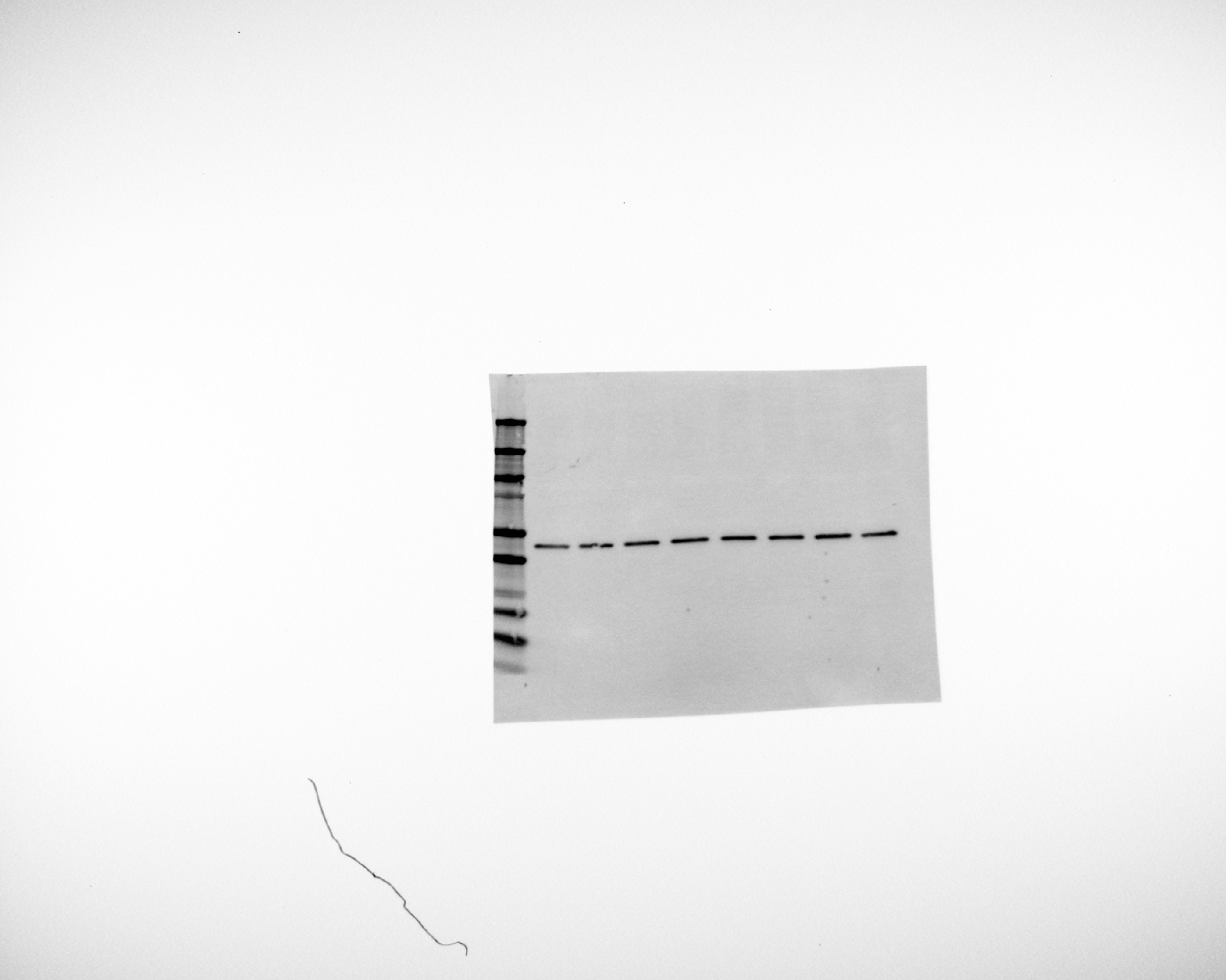

Supplement: Figure 4—source data 1. [file elife-85902-fig4-data1.zip › Figure 4-source data/Unlabelled/4D GAPDH.tif]

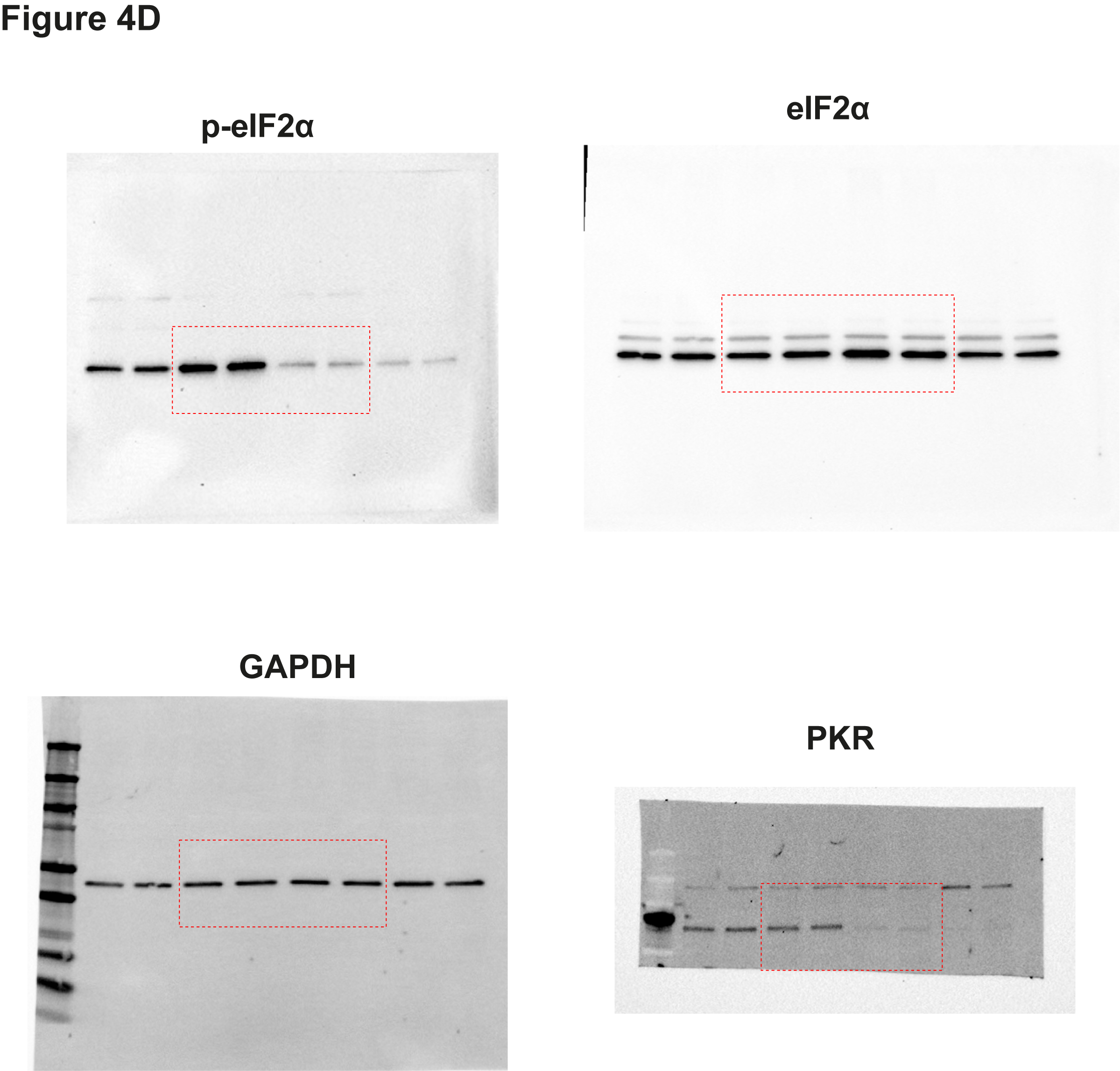

Supplement: Figure 4—source data 1. [file elife-85902-fig4-data1.zip › Figure 4-source data/Labelled/Figure 4-source data 1.tif]

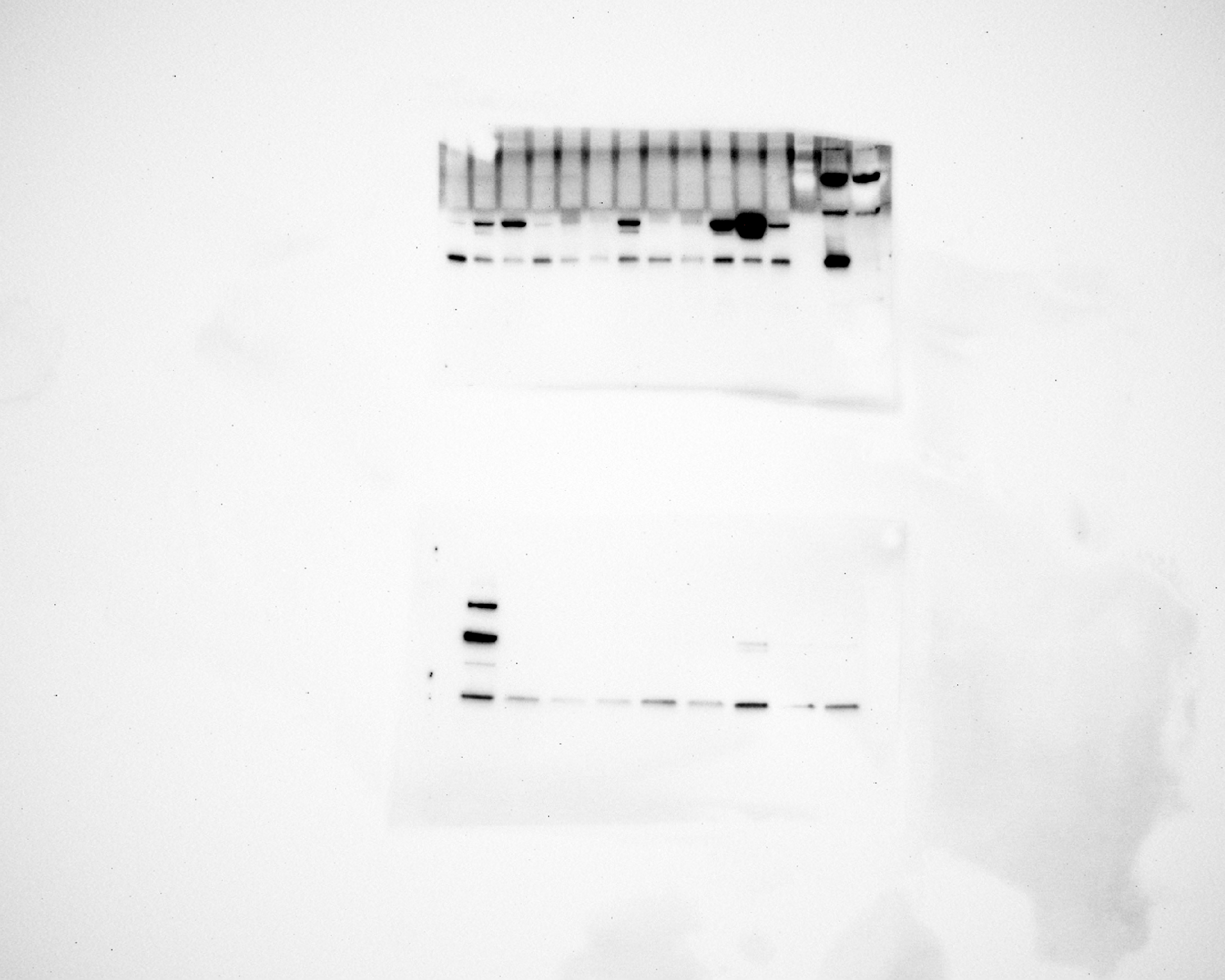

Supplement: Figure 5—source data 1. [file elife-85902-fig5-data1.zip › Figure 5-source data/Unlabelled/6B pEIF2A.tif]

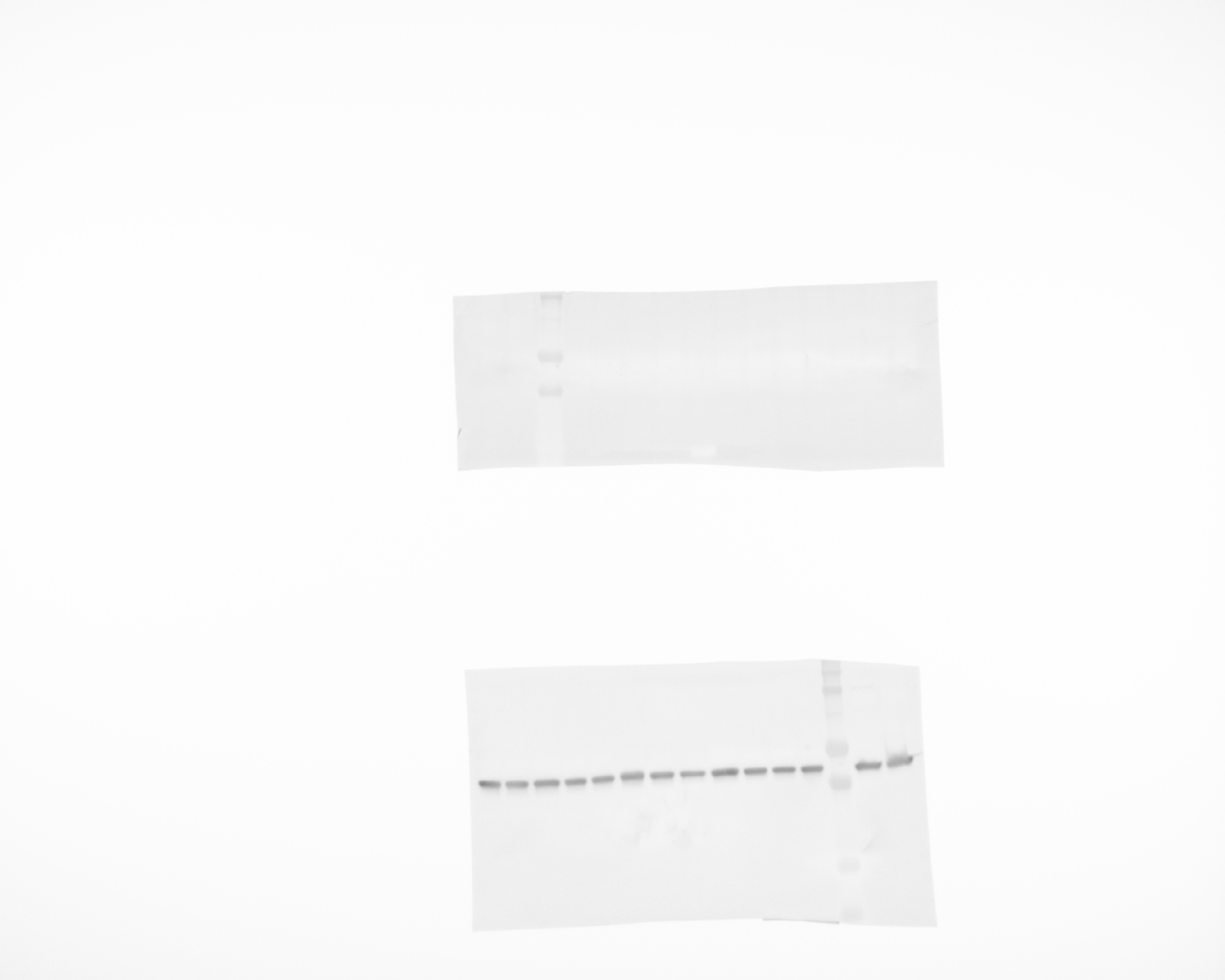

Supplement: Figure 5—source data 1. [file elife-85902-fig5-data1.zip › Figure 5-source data/Unlabelled/6B GAPDH.tif]

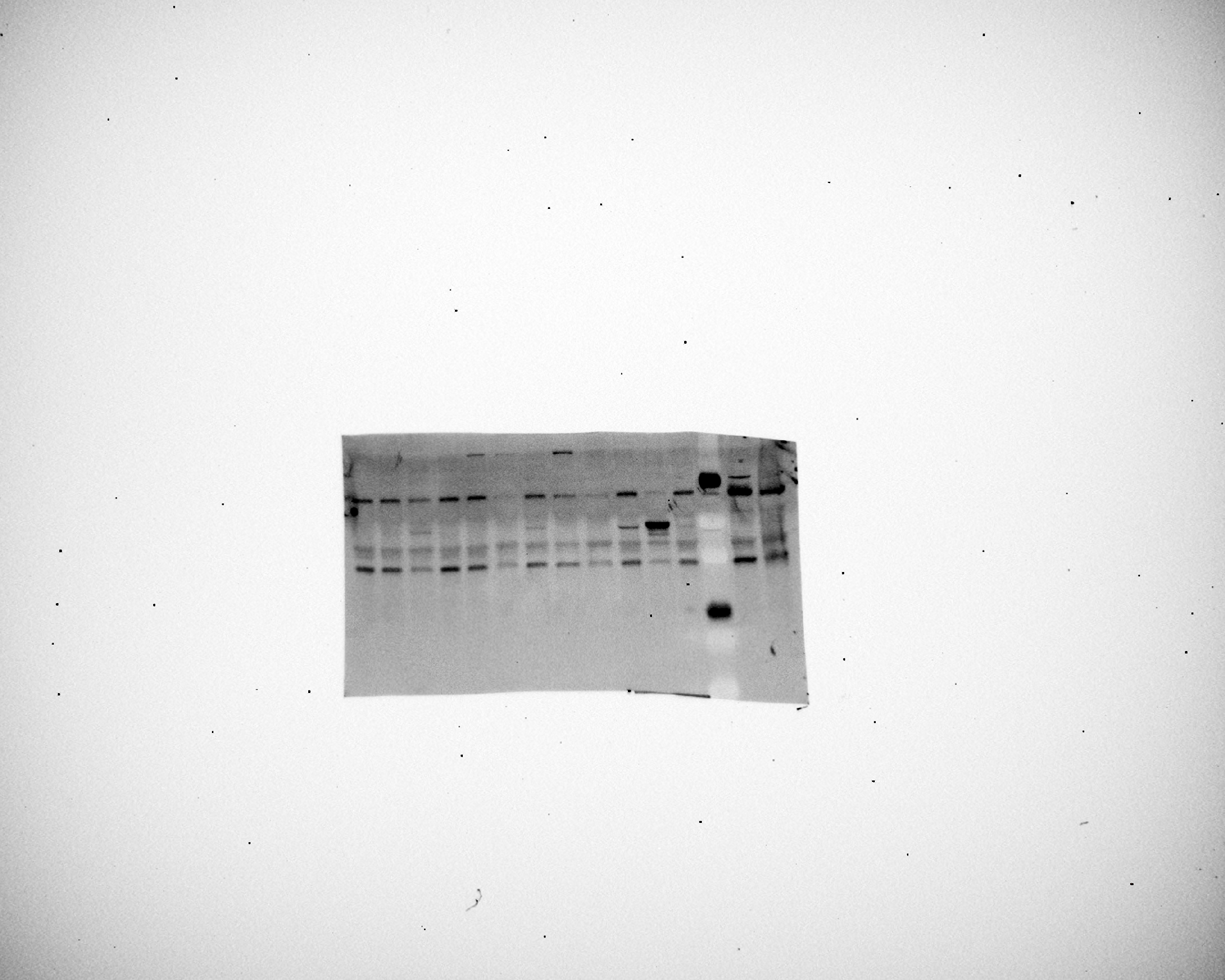

Supplement: Figure 5—source data 1. [file elife-85902-fig5-data1.zip › Figure 5-source data/Unlabelled/6B EIF2A.tif]

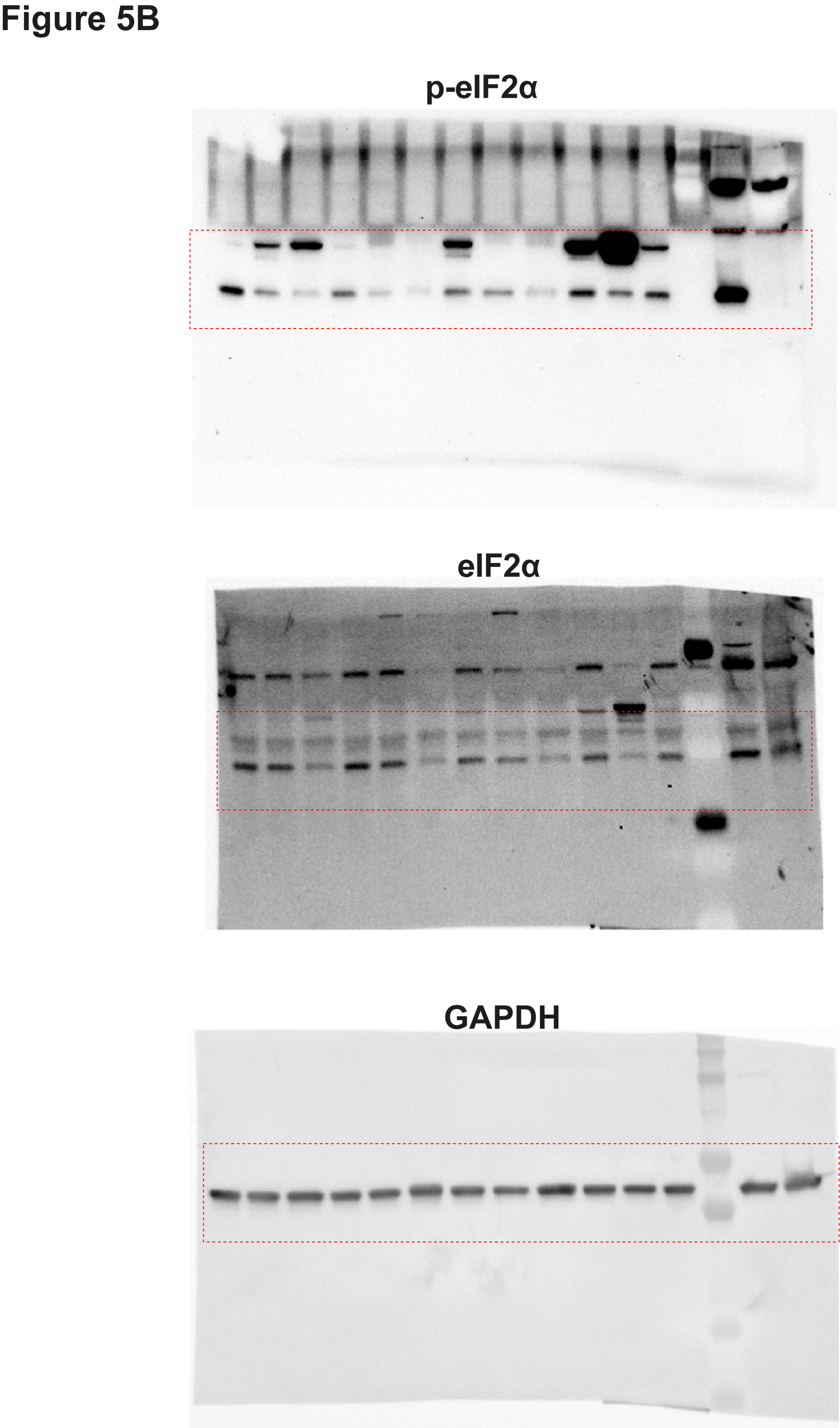

Supplement: Figure 5—source data 1. [file elife-85902-fig5-data1.zip › Figure 5-source data/Labelled/Figure 5-source data 1.tif]

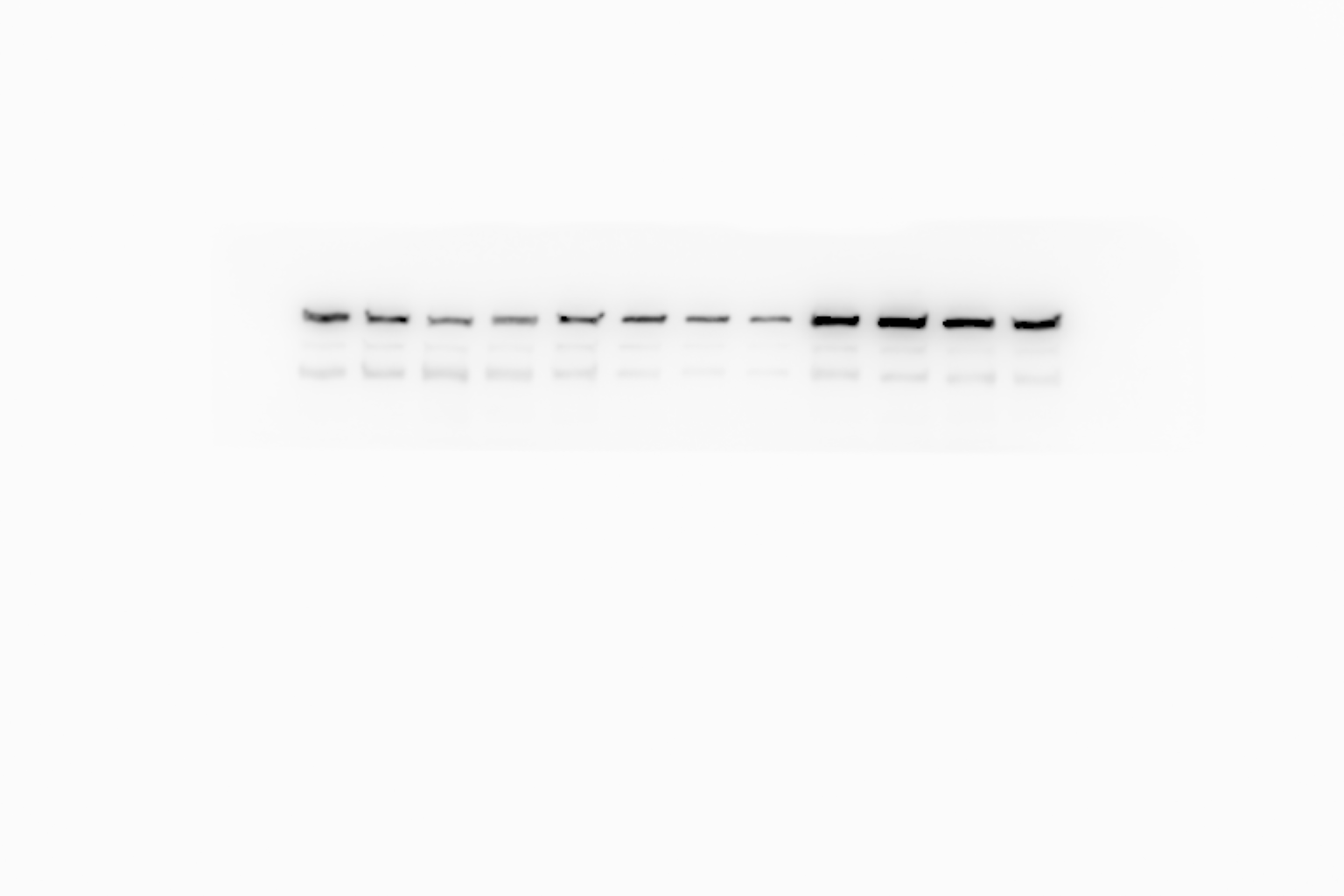

Supplement: Figure 5—figure supplement 1—source data 1. [file elife-85902-fig5-figsupp1-data1.zip › Figure 5-figure supplement 1-source data/Unlabelled/A phospho-PKR-high exposure_3.tif]

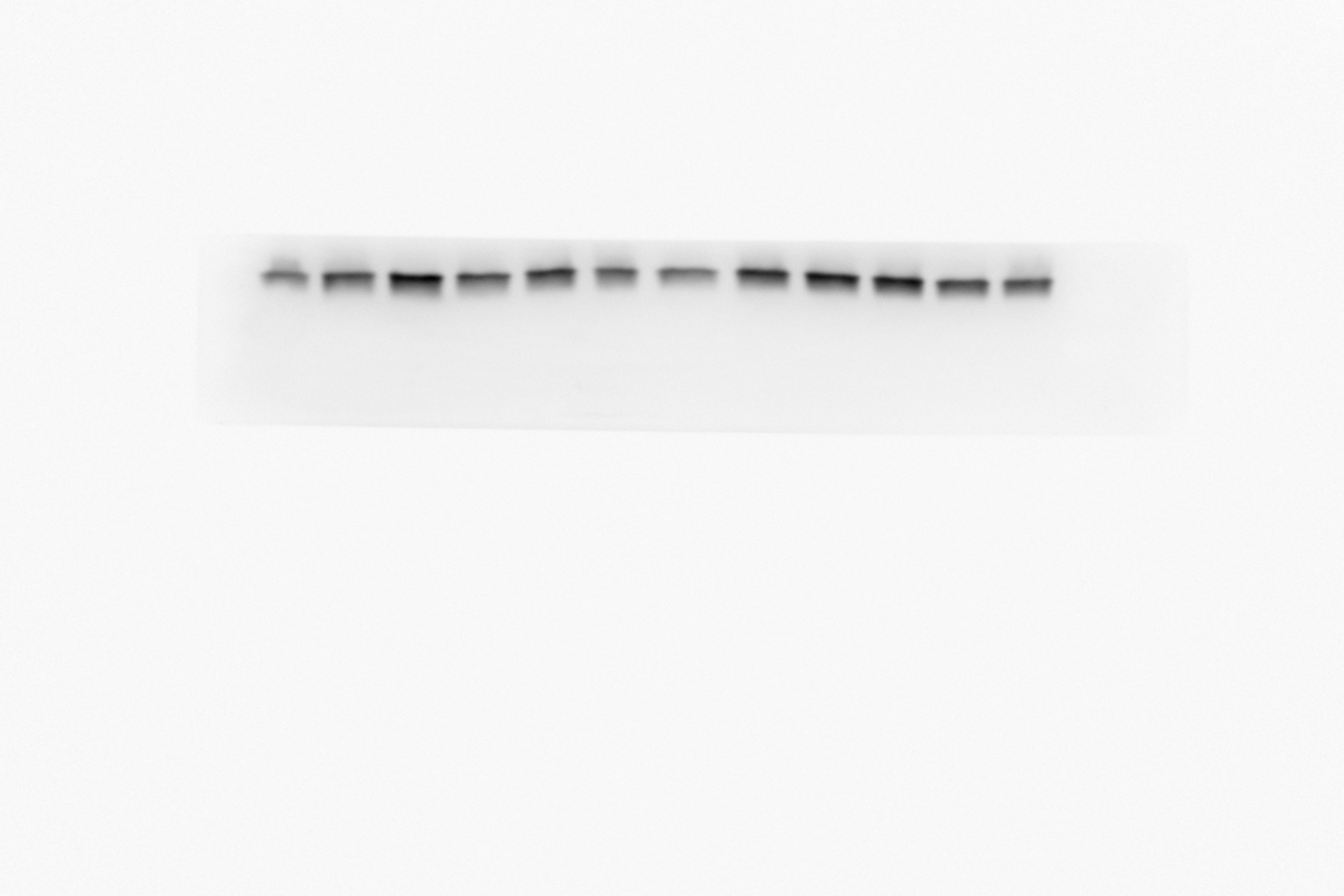

Supplement: Figure 5—figure supplement 1—source data 1. [file elife-85902-fig5-figsupp1-data1.zip › Figure 5-figure supplement 1-source data/Unlabelled/A eif2a_12.tif]

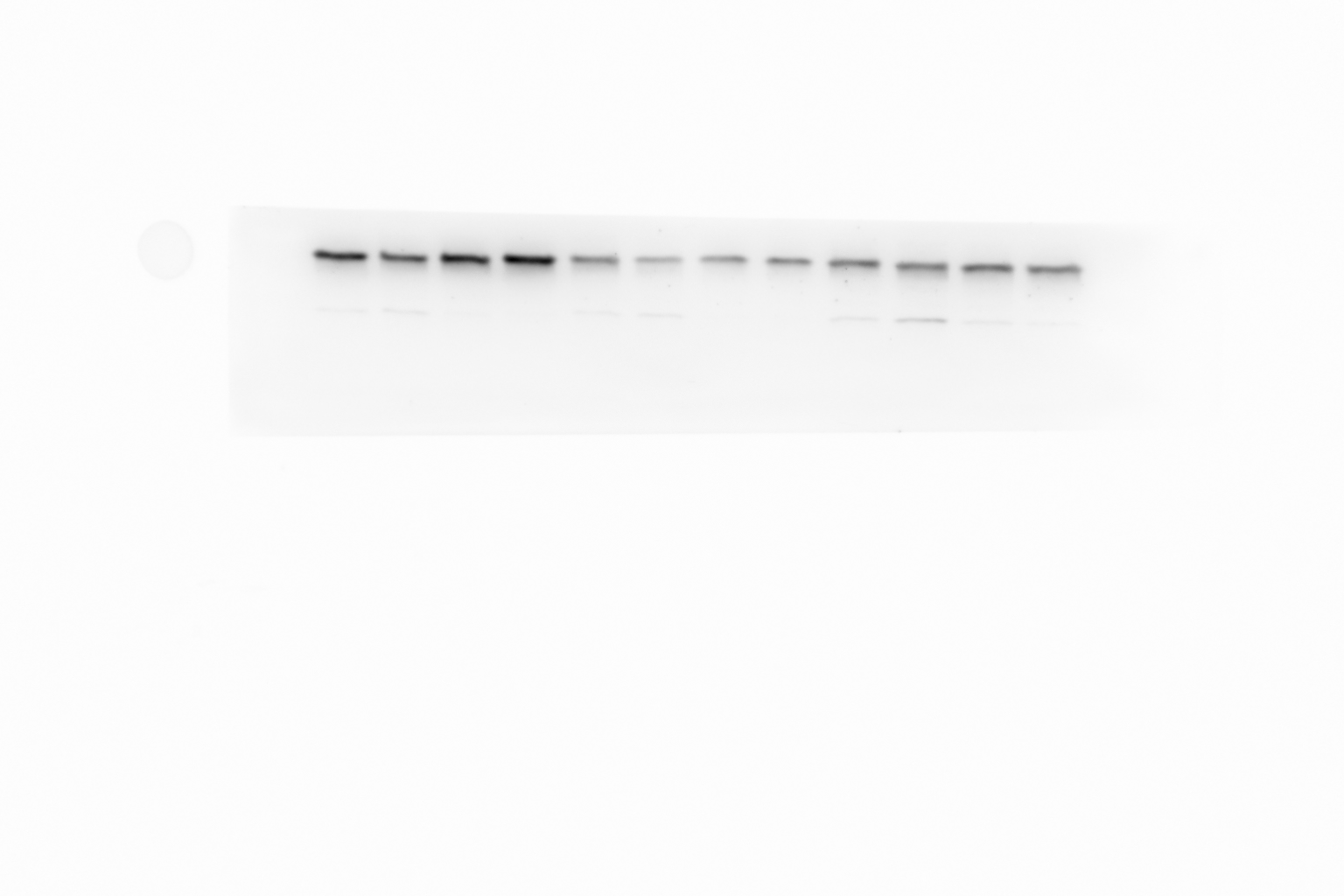

Supplement: Figure 5—figure supplement 1—source data 1. [file elife-85902-fig5-figsupp1-data1.zip › Figure 5-figure supplement 1-source data/Unlabelled/phospho-eif2a.tif]

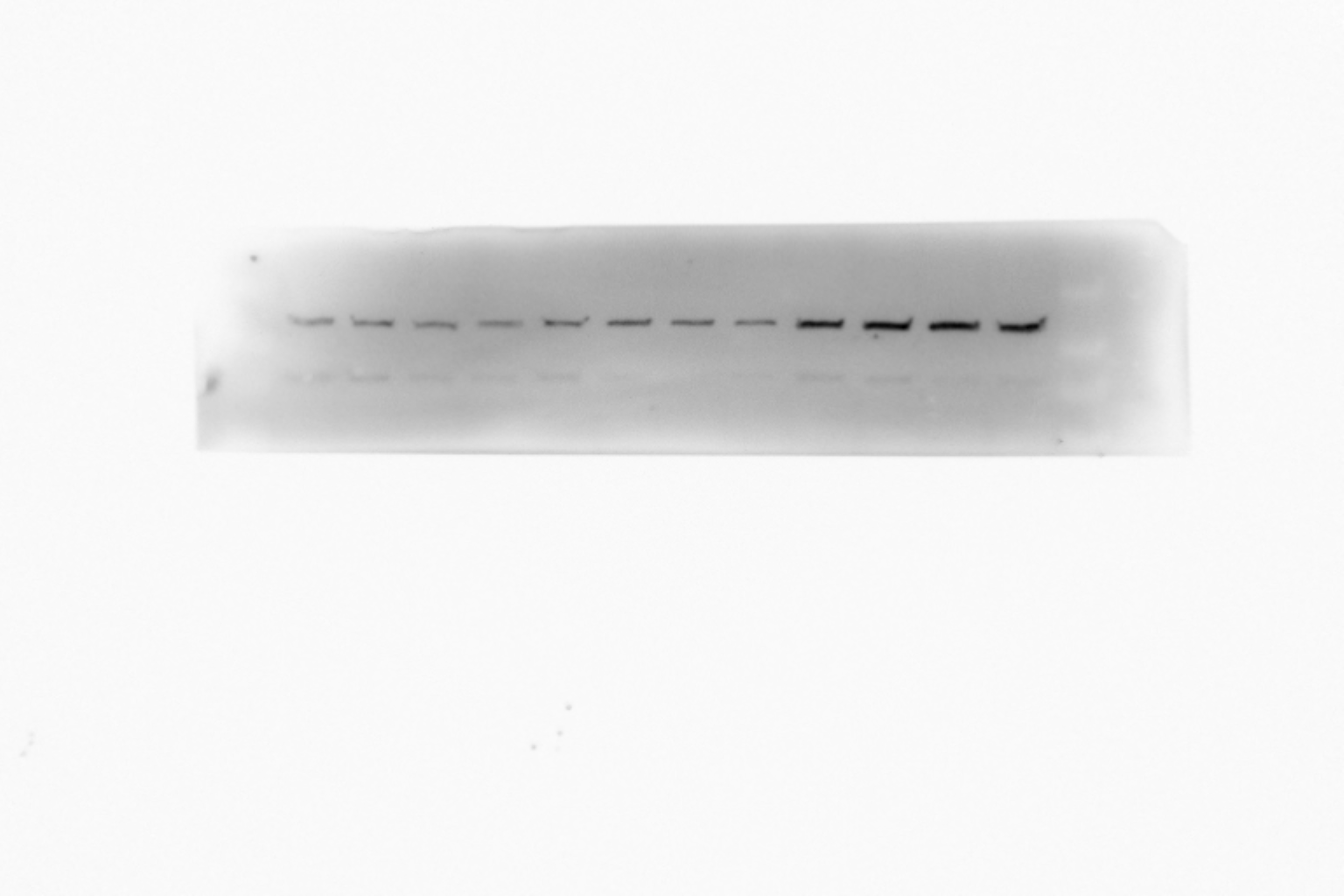

Supplement: Figure 5—figure supplement 1—source data 1. [file elife-85902-fig5-figsupp1-data1.zip › Figure 5-figure supplement 1-source data/Unlabelled/A Blot2-PKR.tif]

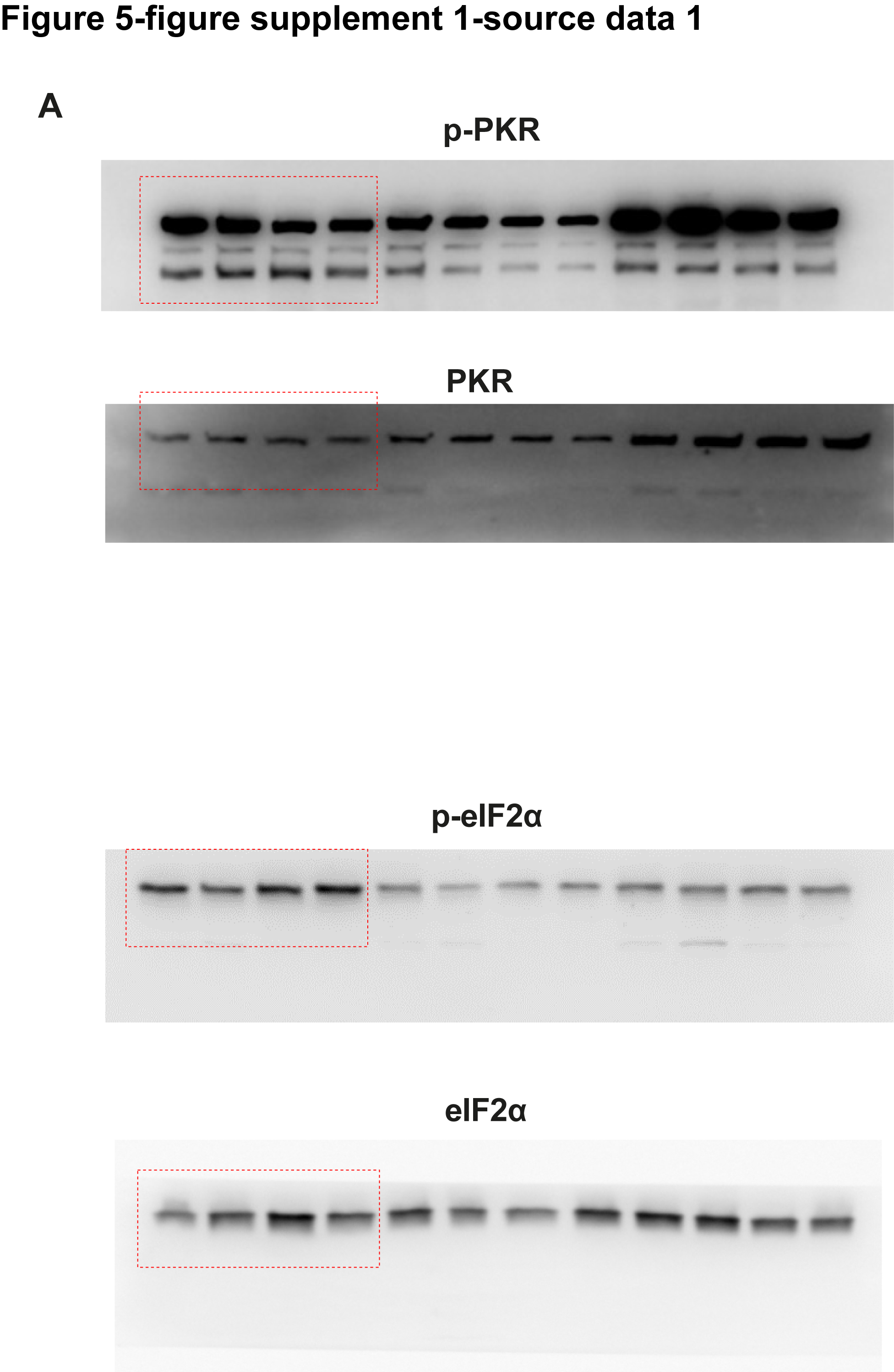

Supplement: Figure 5—figure supplement 1—source data 1. [file elife-85902-fig5-figsupp1-data1.zip › Figure 5-figure supplement 1-source data/Labelled/Figure 5-figure supplement 1-source data 1.tif]

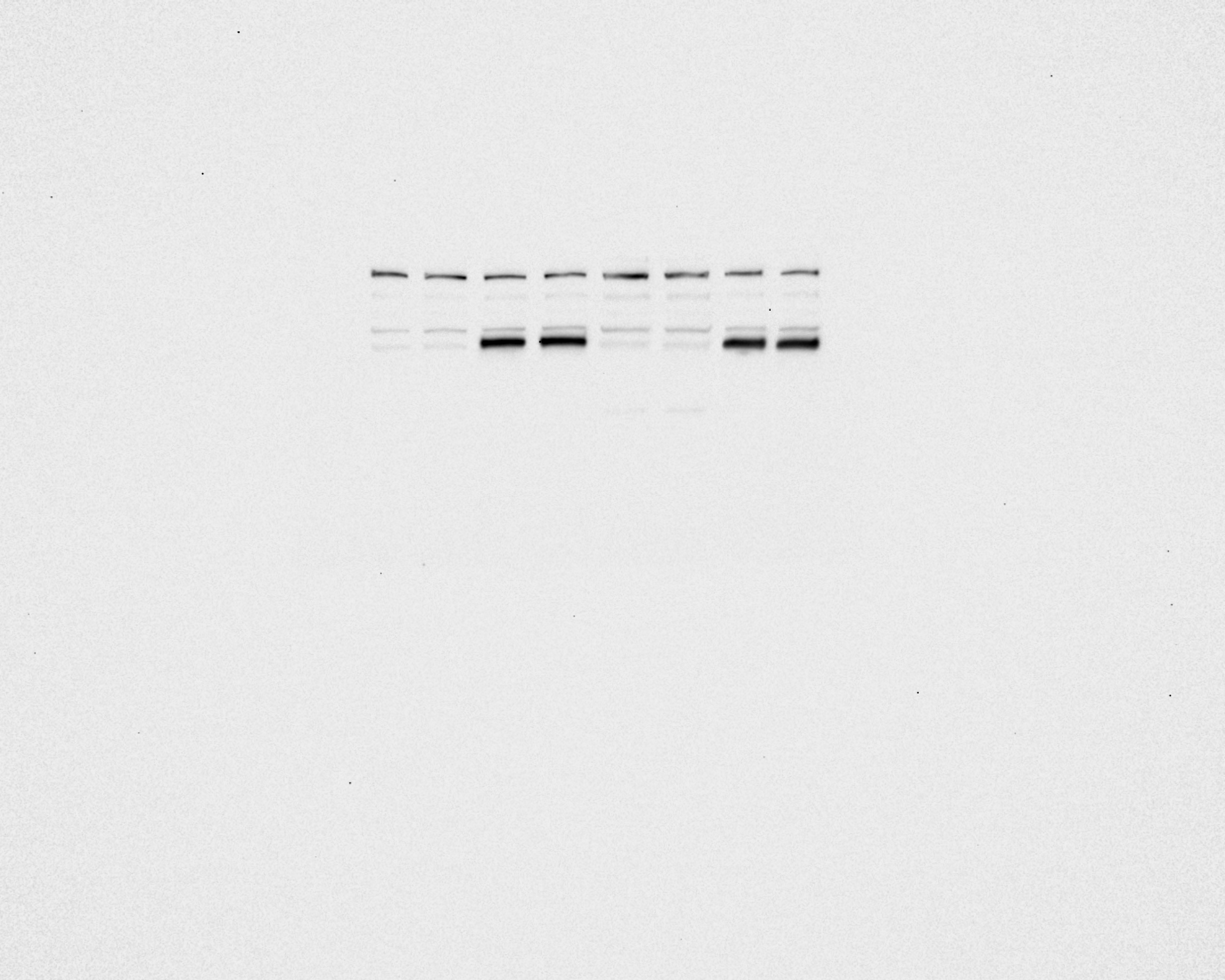

Supplement: Figure 6—source data 1. [file elife-85902-fig6-data1.zip › Figure 6-source data/Unlabelled/6A pPKR.tif]

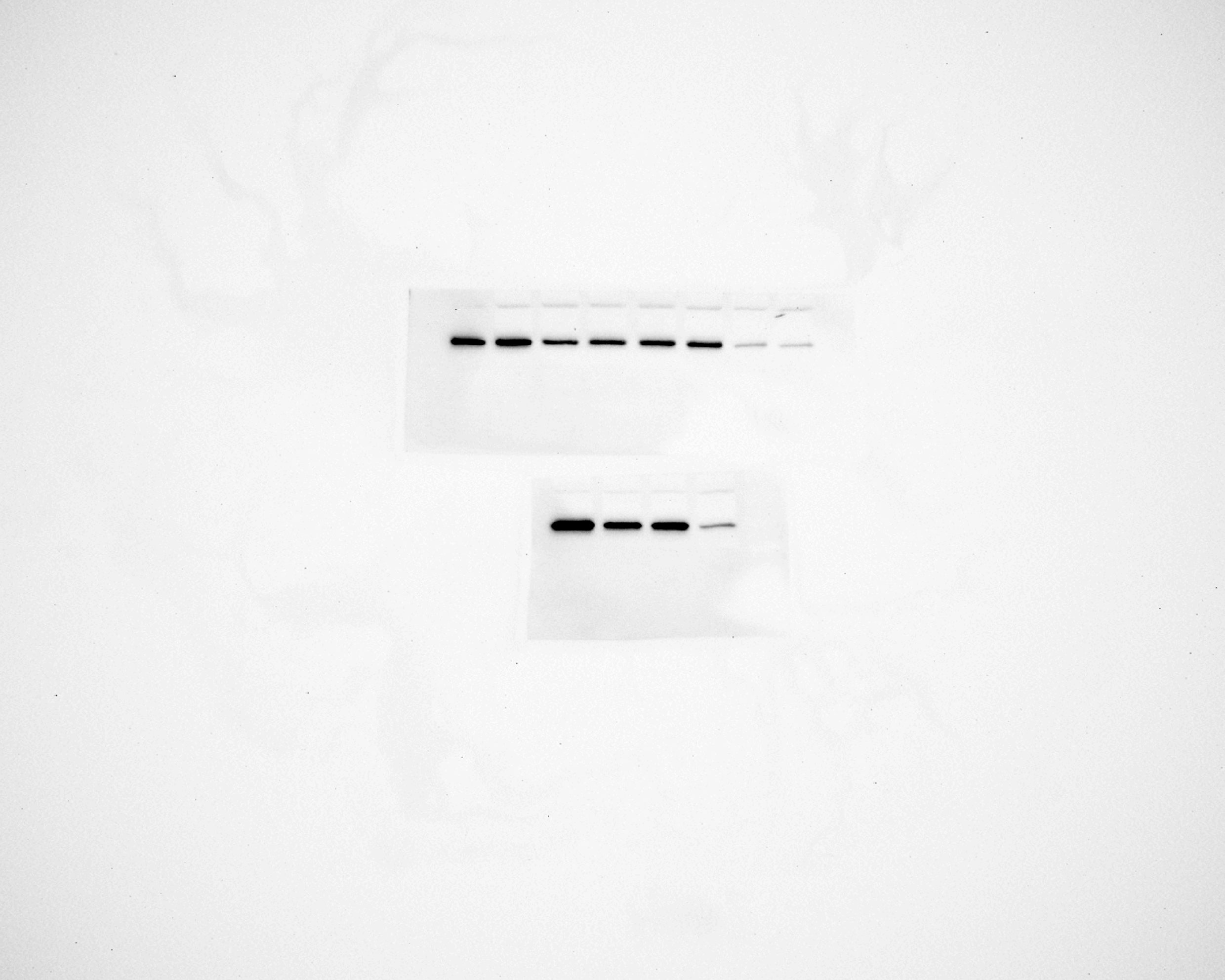

Supplement: Figure 6—source data 1. [file elife-85902-fig6-data1.zip › Figure 6-source data/Unlabelled/6E peif2a.tif]

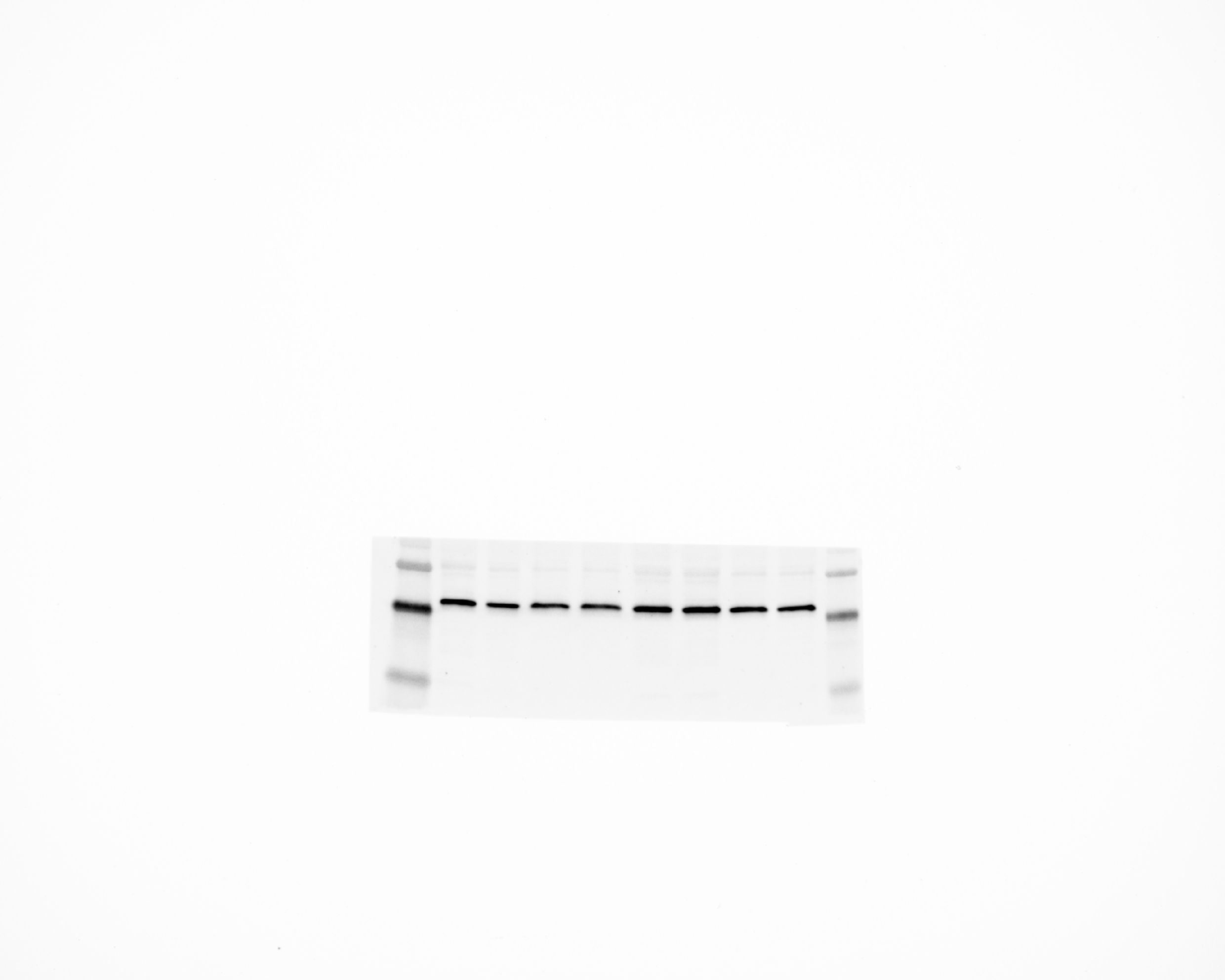

Supplement: Figure 6—source data 1. [file elife-85902-fig6-data1.zip › Figure 6-source data/Unlabelled/6A eif2a.tif]

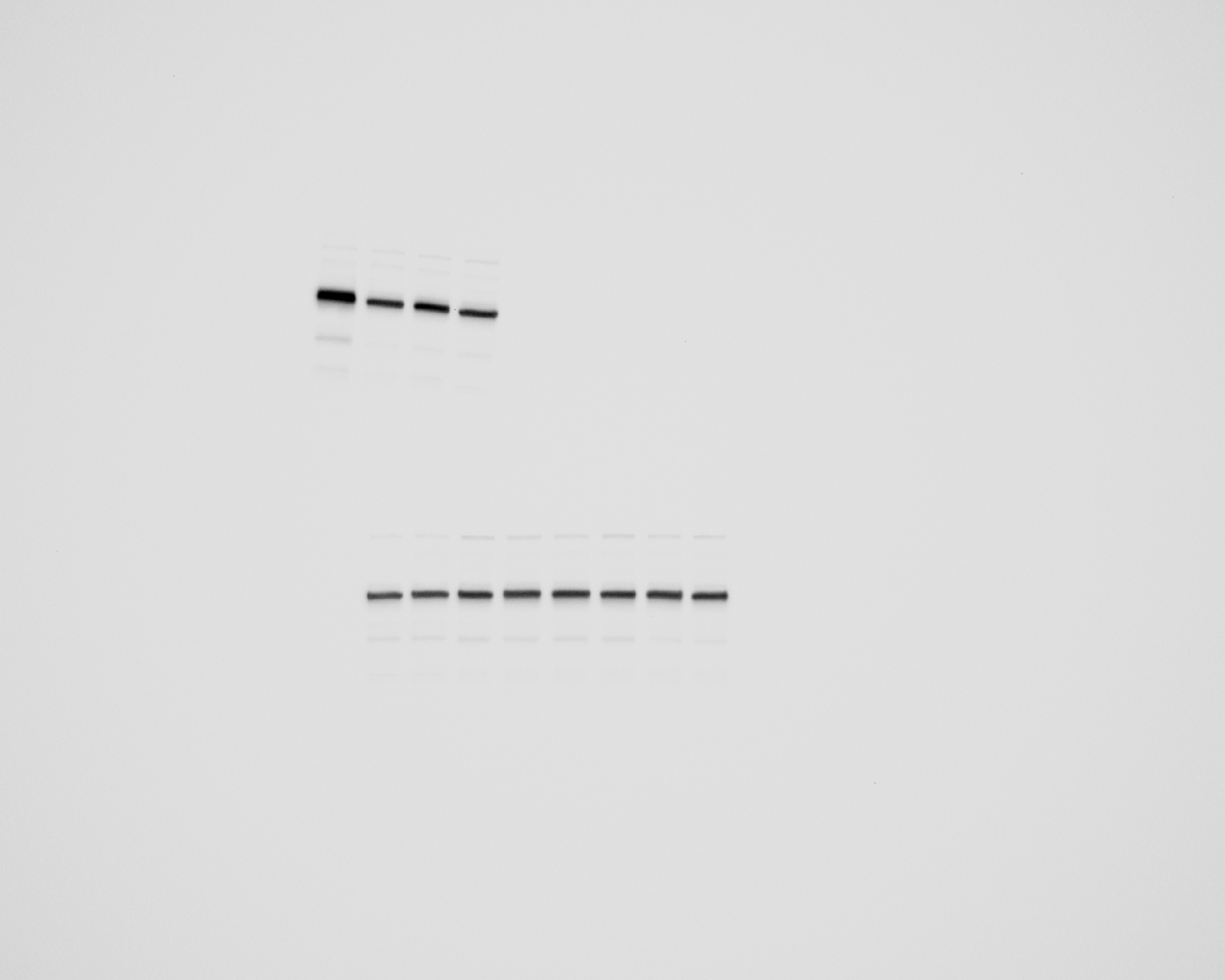

Supplement: Figure 6—source data 1. [file elife-85902-fig6-data1.zip › Figure 6-source data/Unlabelled/6E PKR.tif]

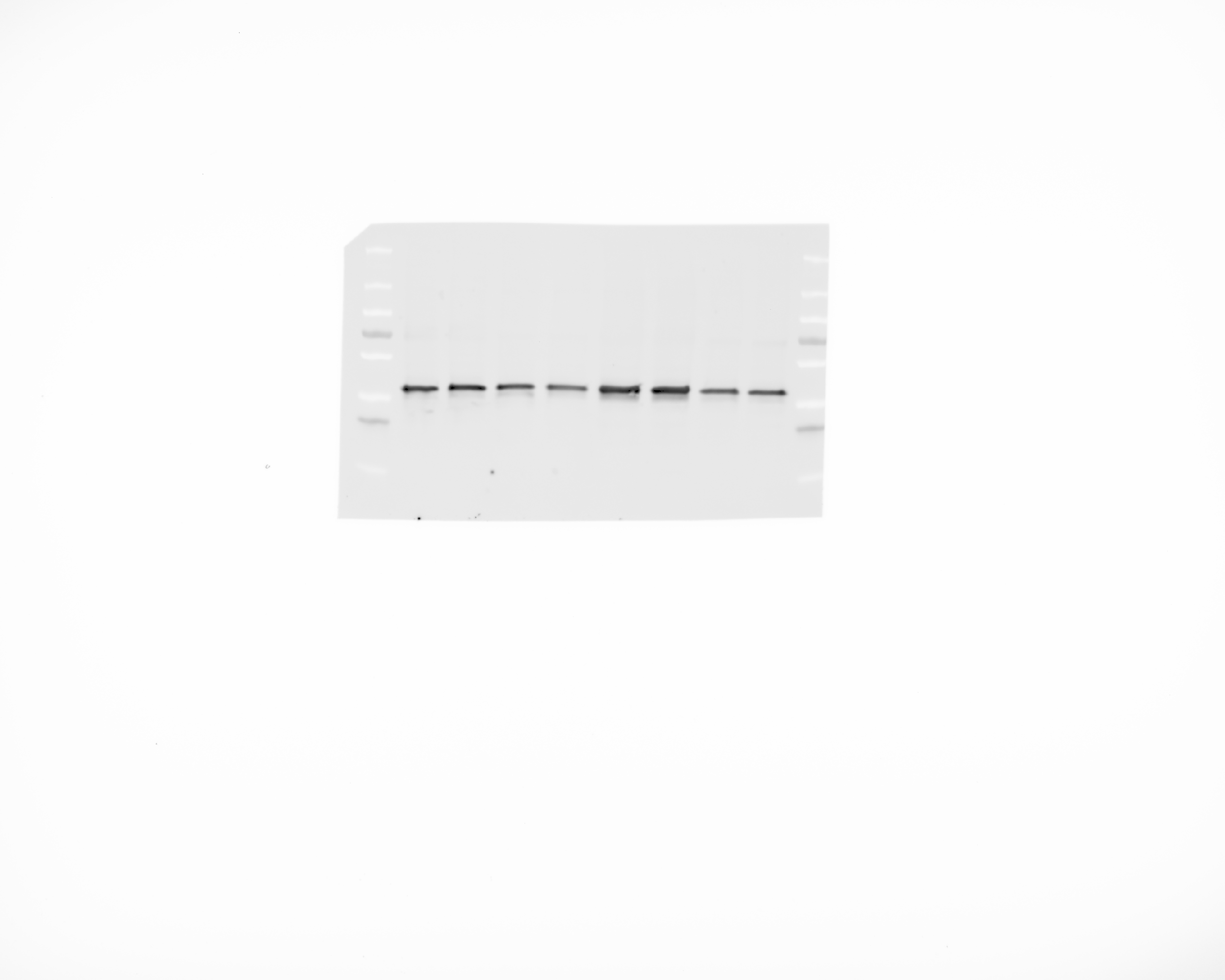

Supplement: Figure 6—source data 1. [file elife-85902-fig6-data1.zip › Figure 6-source data/Unlabelled/6A GAPDH.tif]

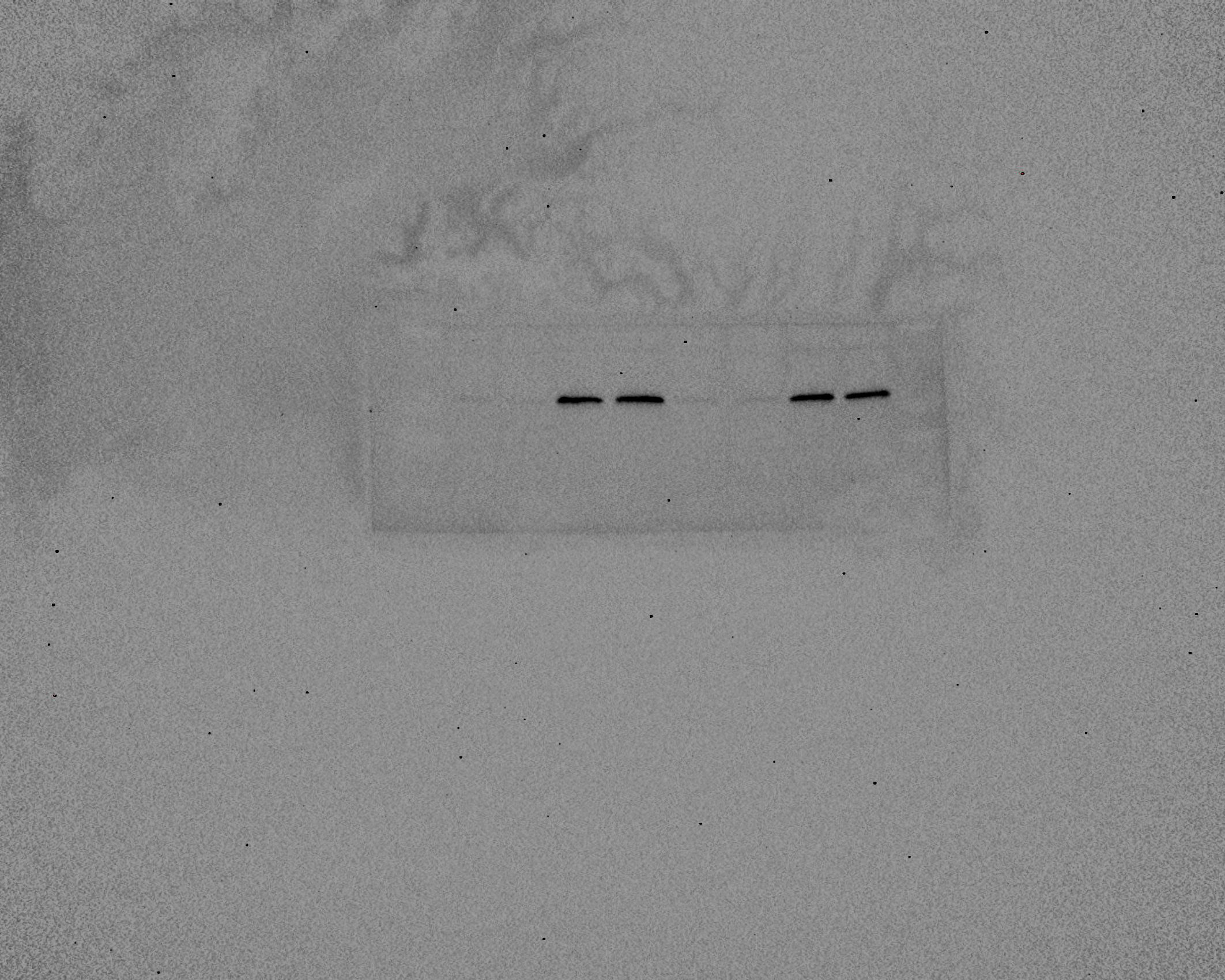

Supplement: Figure 6—source data 1. [file elife-85902-fig6-data1.zip › Figure 6-source data/Unlabelled/6A peif2a.tif]

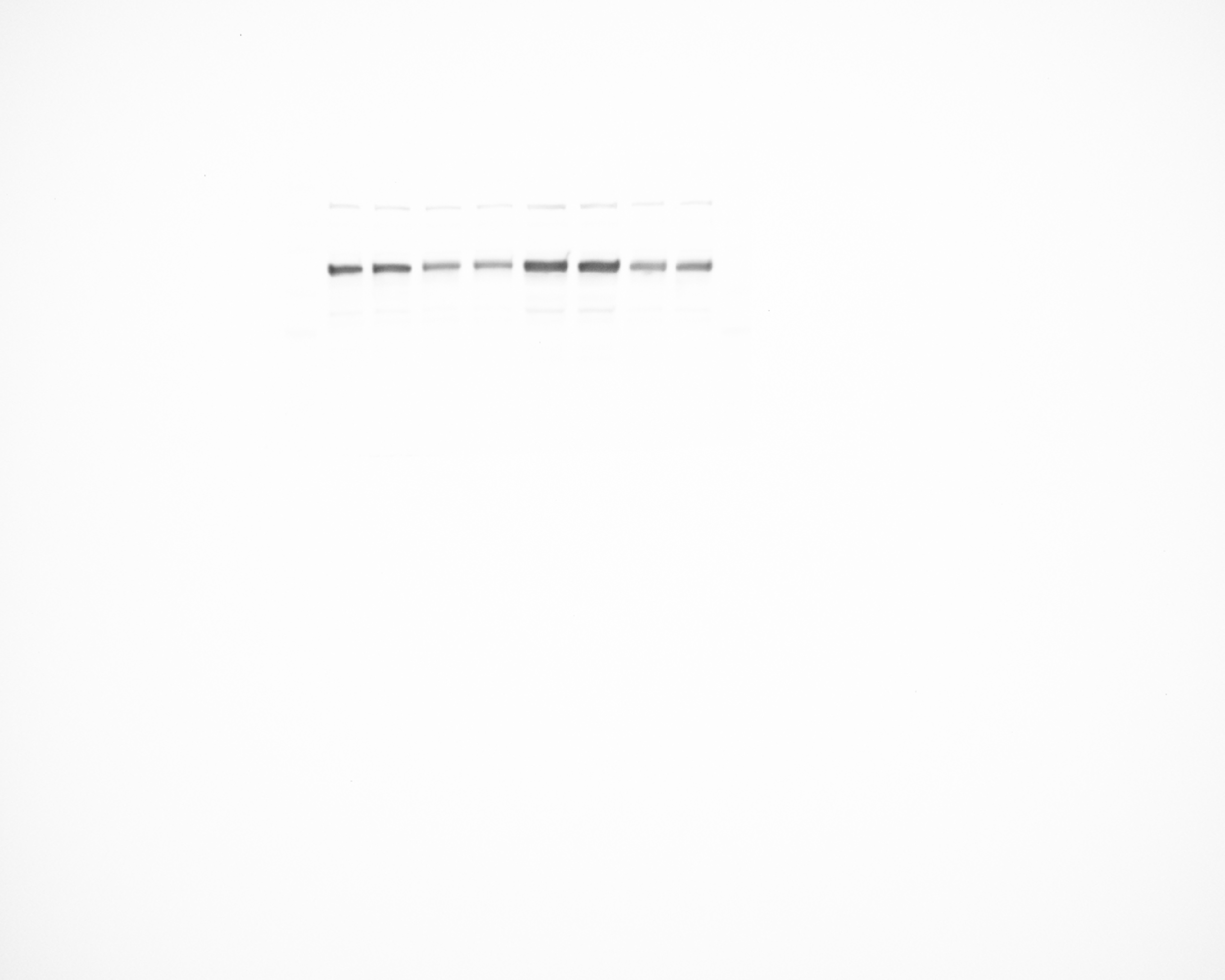

Supplement: Figure 6—source data 1. [file elife-85902-fig6-data1.zip › Figure 6-source data/Unlabelled/6A PKR.tif]

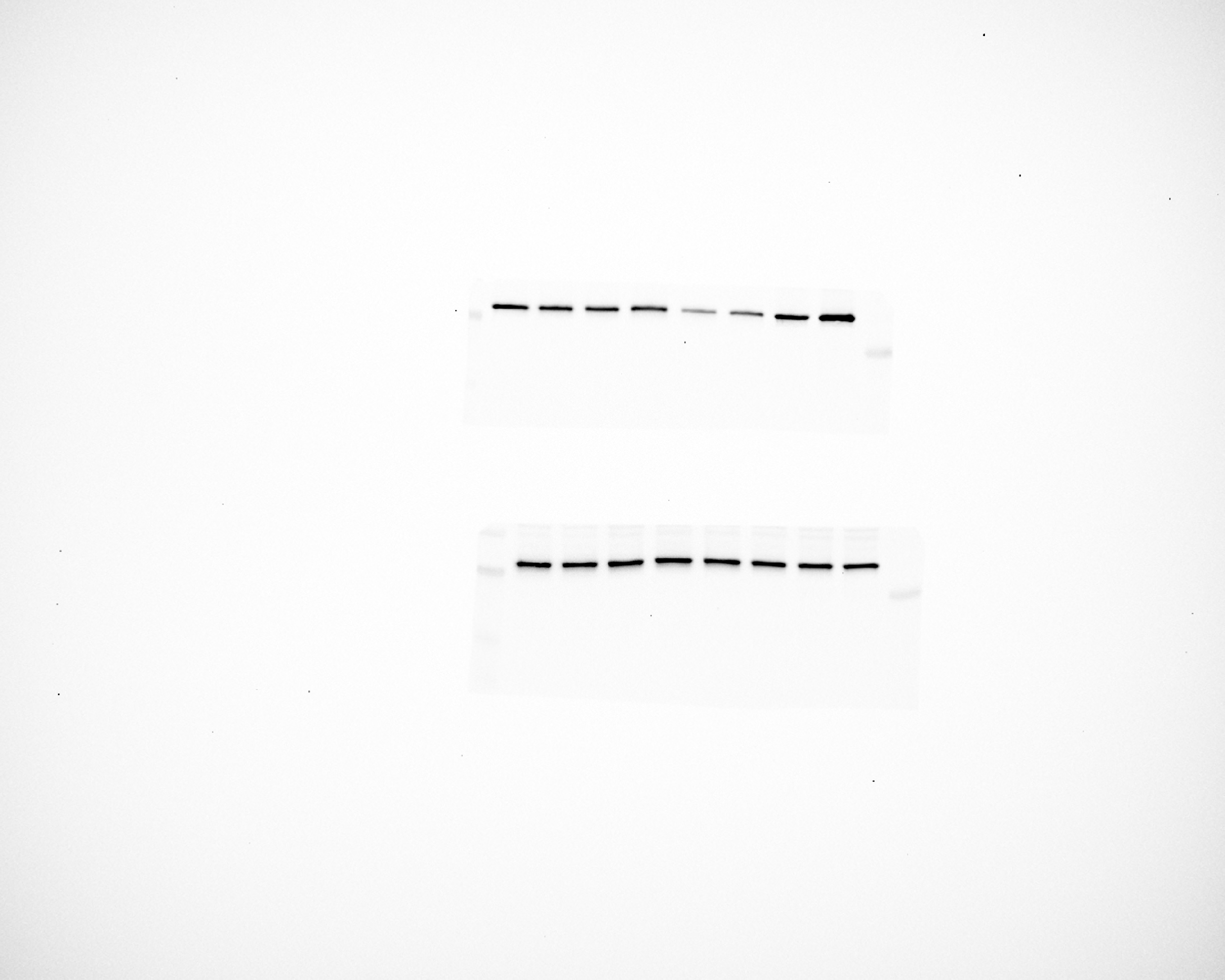

Supplement: Figure 6—source data 1. [file elife-85902-fig6-data1.zip › Figure 6-source data/Unlabelled/6E eif2a.tif]

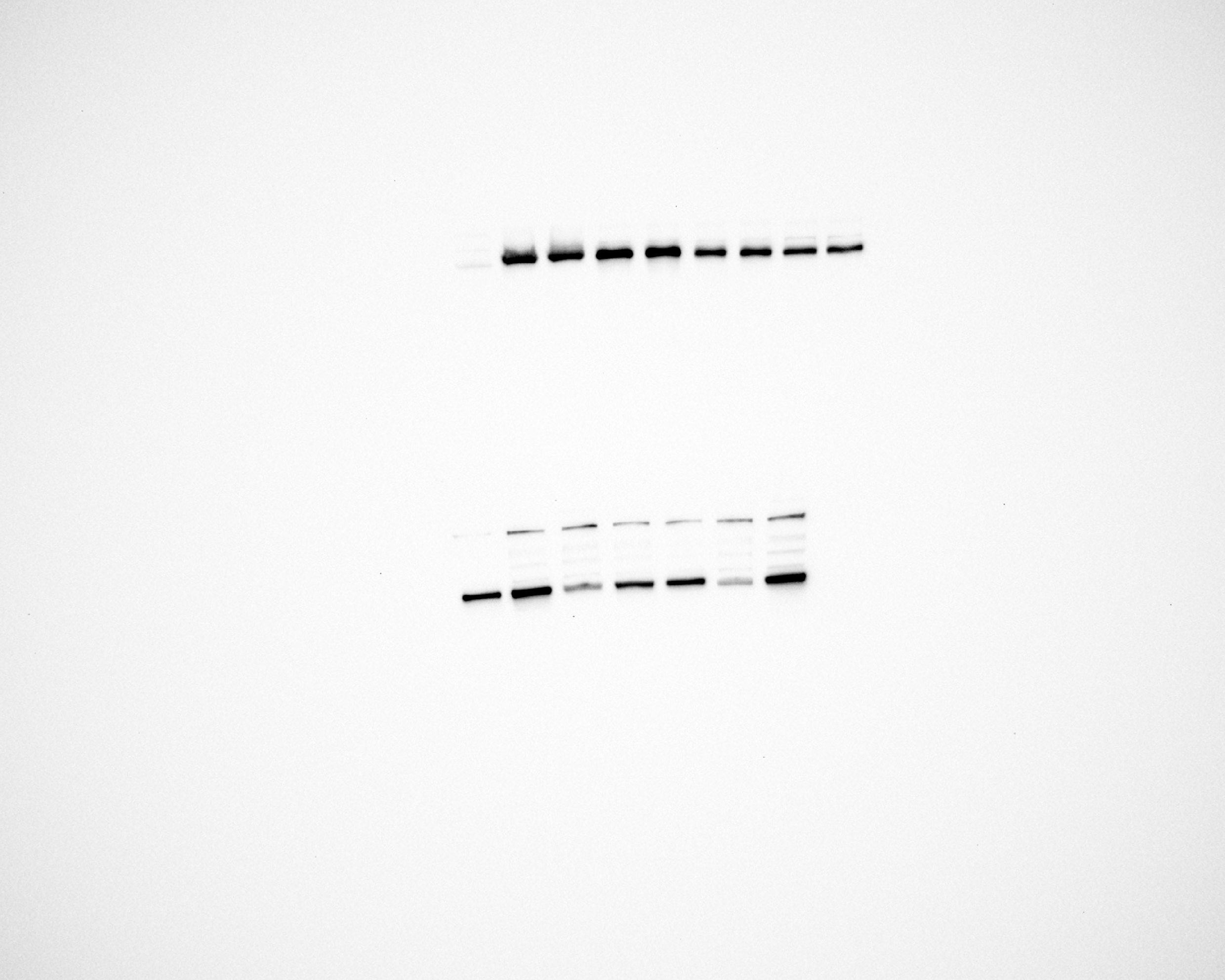

Supplement: Figure 6—source data 1. [file elife-85902-fig6-data1.zip › Figure 6-source data/Unlabelled/6C pPKR.tif]

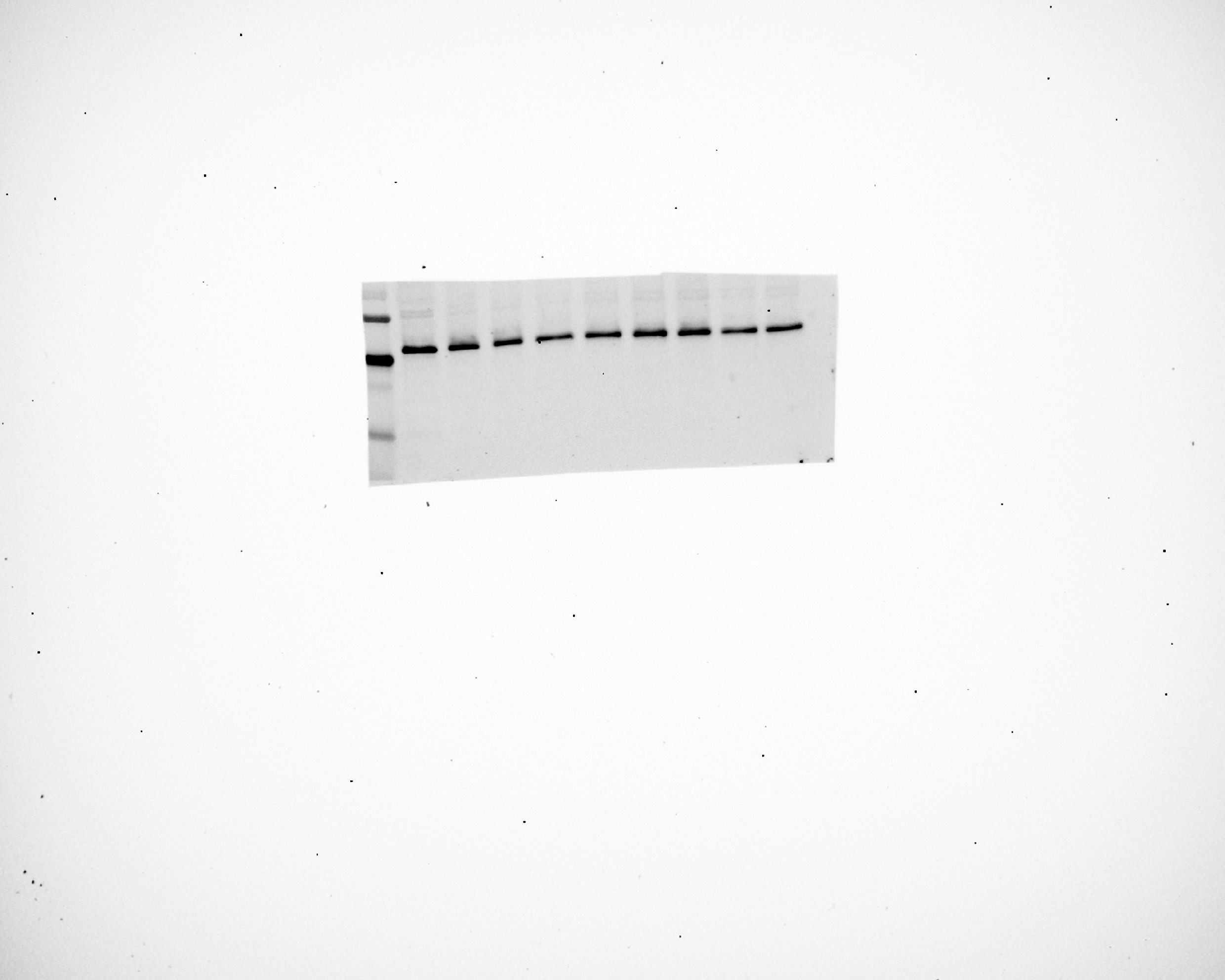

Supplement: Figure 6—source data 1. [file elife-85902-fig6-data1.zip › Figure 6-source data/Unlabelled/6C eif2a.tif]

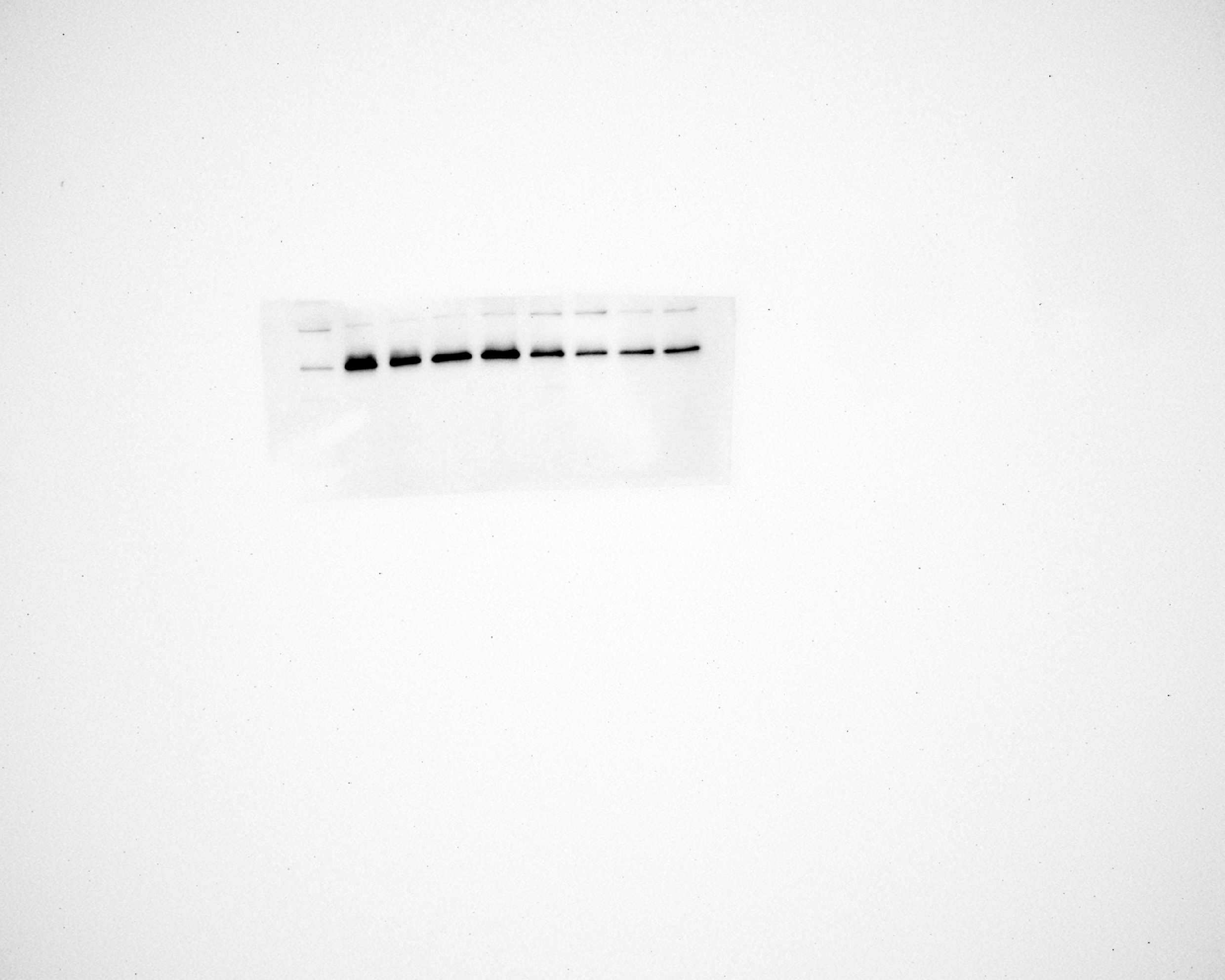

Supplement: Figure 6—source data 1. [file elife-85902-fig6-data1.zip › Figure 6-source data/Unlabelled/6C peif2a.tif]

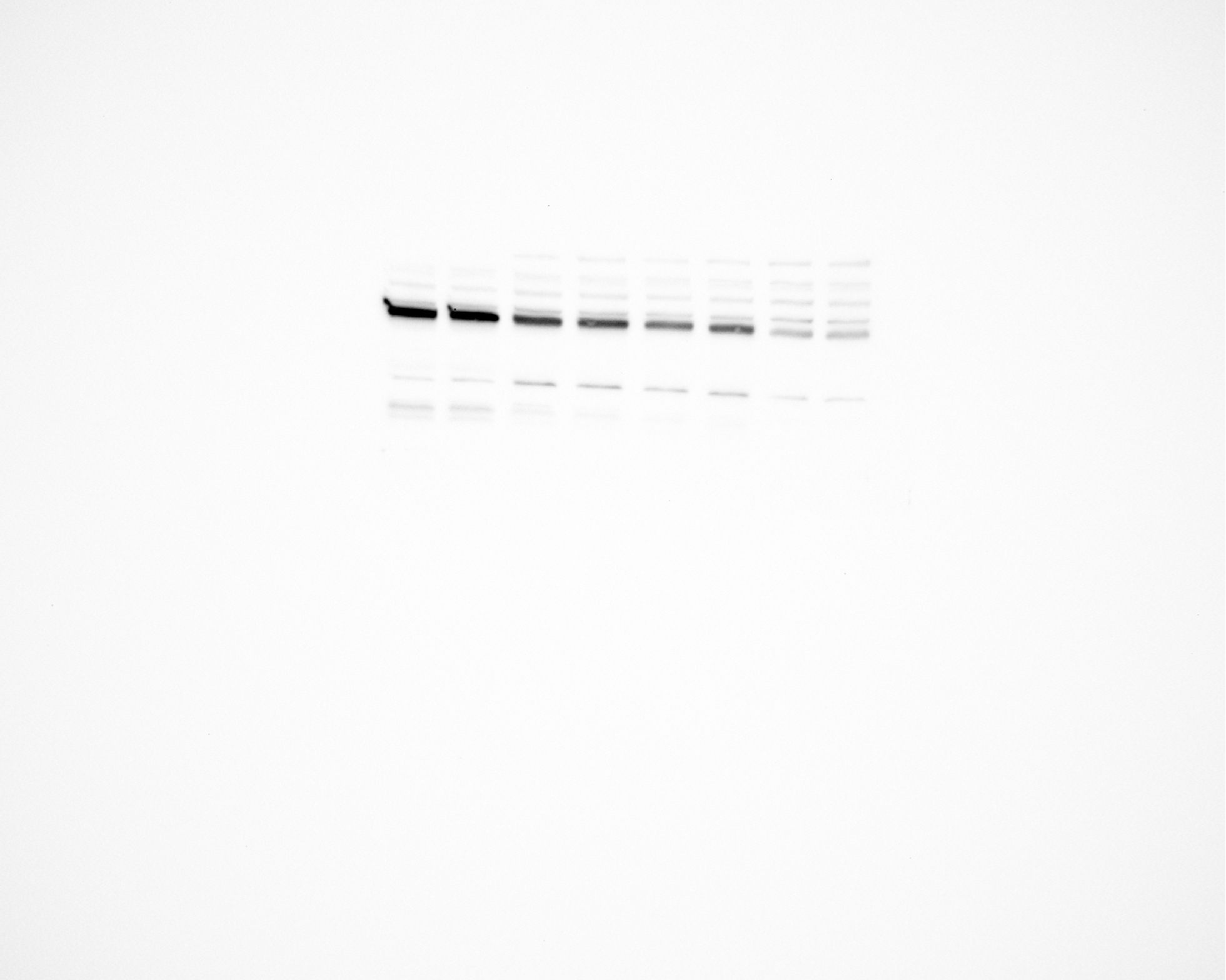

Supplement: Figure 6—source data 1. [file elife-85902-fig6-data1.zip › Figure 6-source data/Unlabelled/6E pPKR.tif]

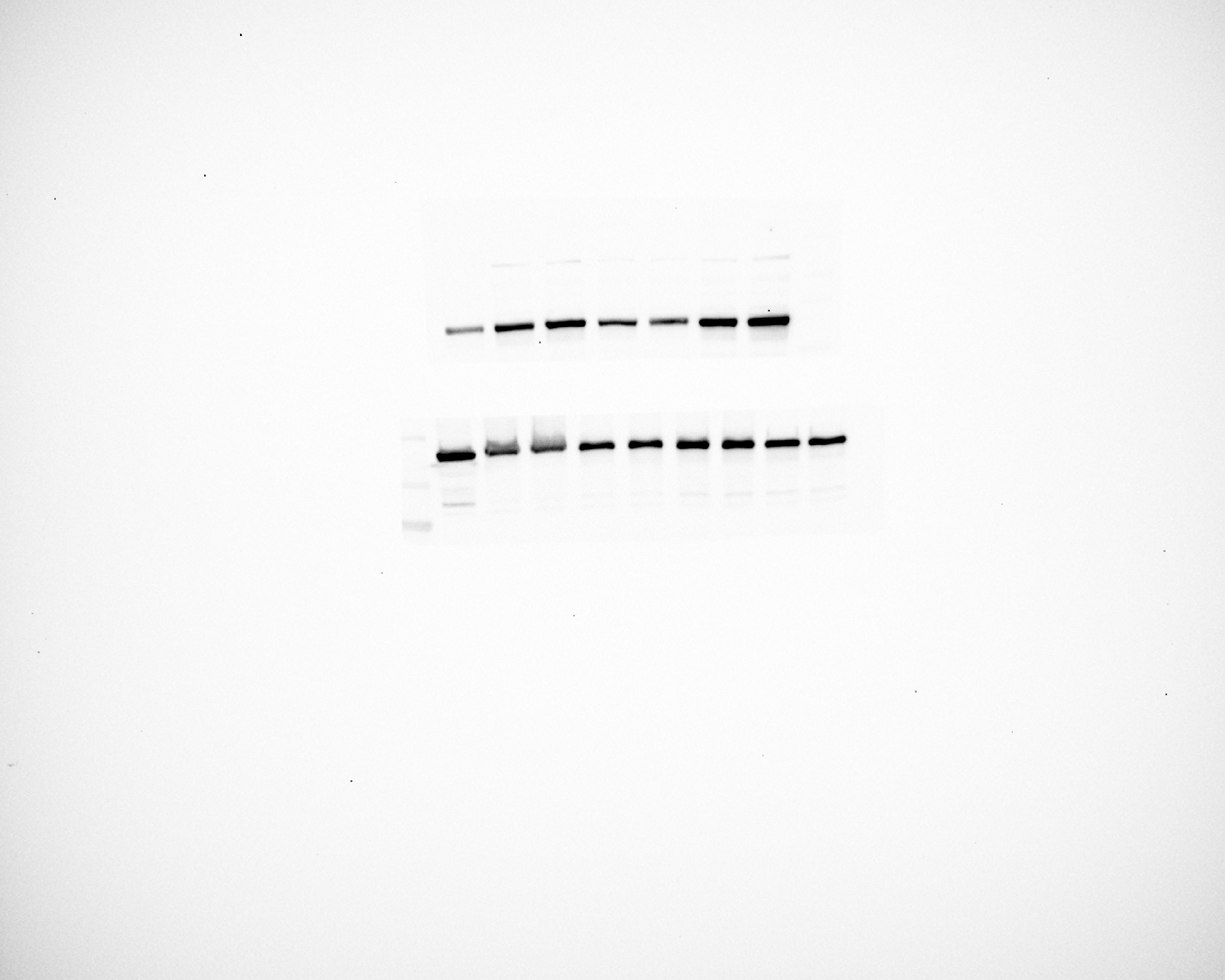

Supplement: Figure 6—source data 1. [file elife-85902-fig6-data1.zip › Figure 6-source data/Unlabelled/6C PKR.tif]

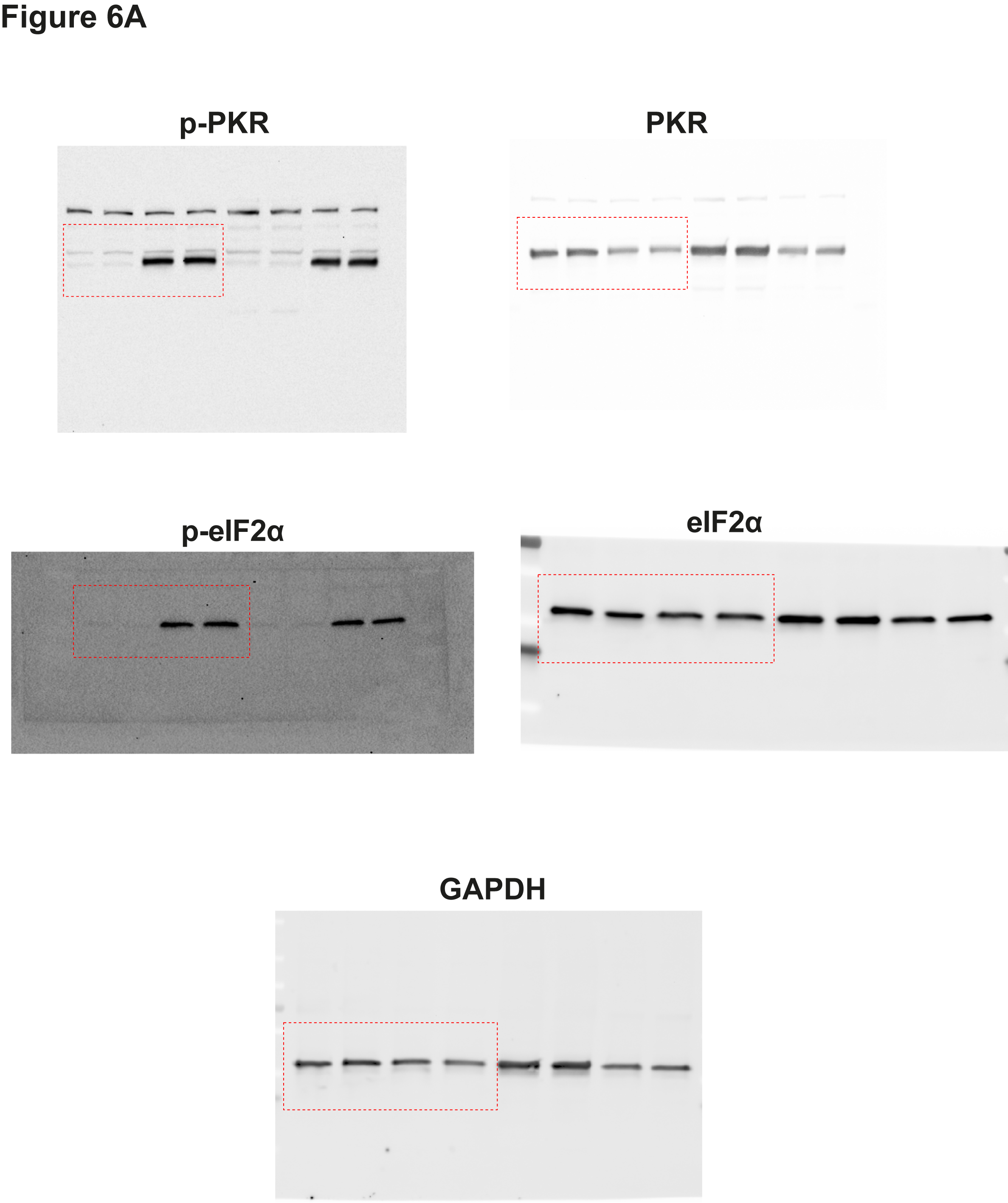

Supplement: Figure 6—source data 1. [file elife-85902-fig6-data1.zip › Figure 6-source data/Labelled/Figure 6-source data 1.tif]

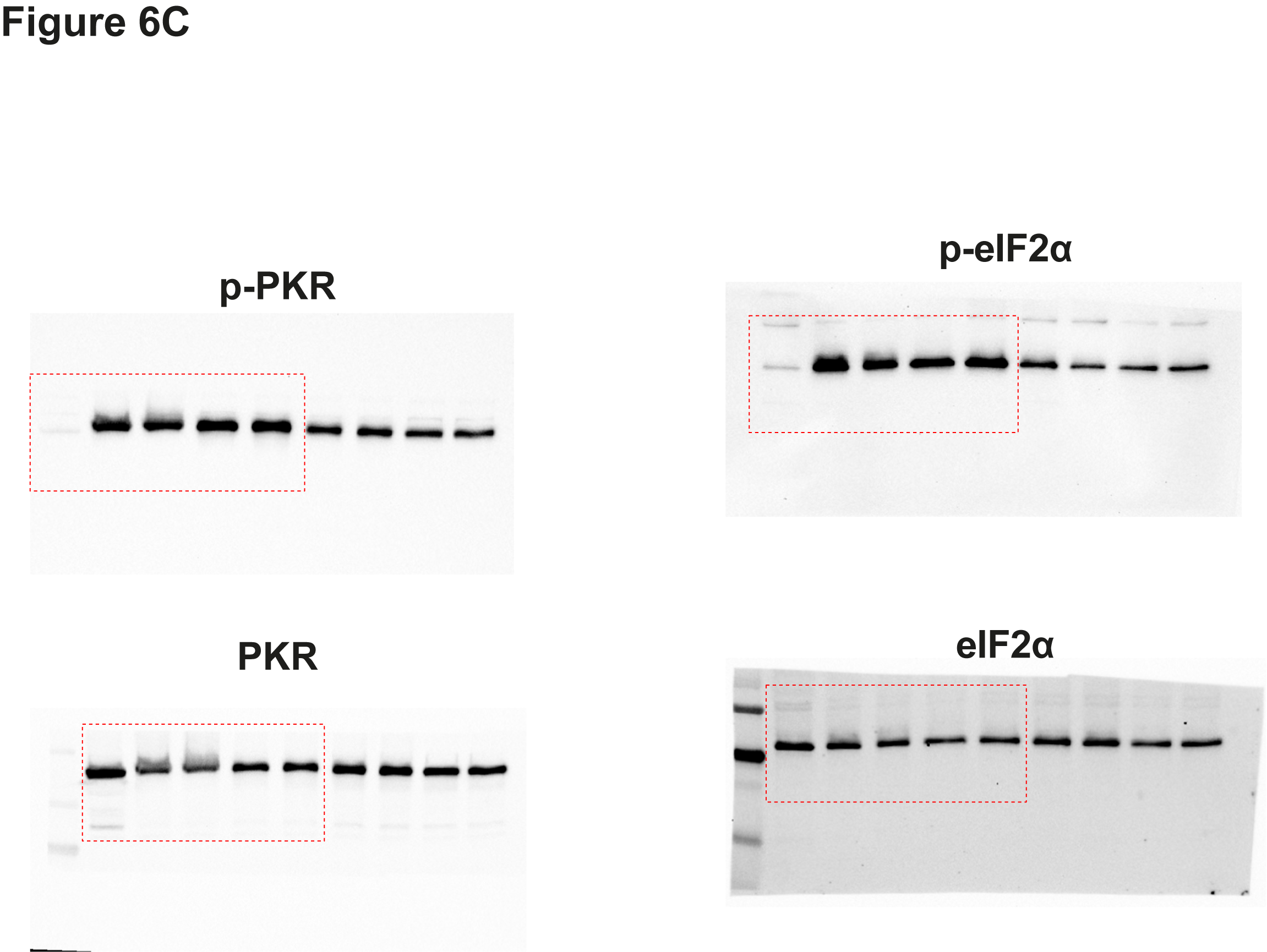

Supplement: Figure 6—source data 1. [file elife-85902-fig6-data1.zip › Figure 6-source data/Labelled/Figure 6-source data 2.tif]

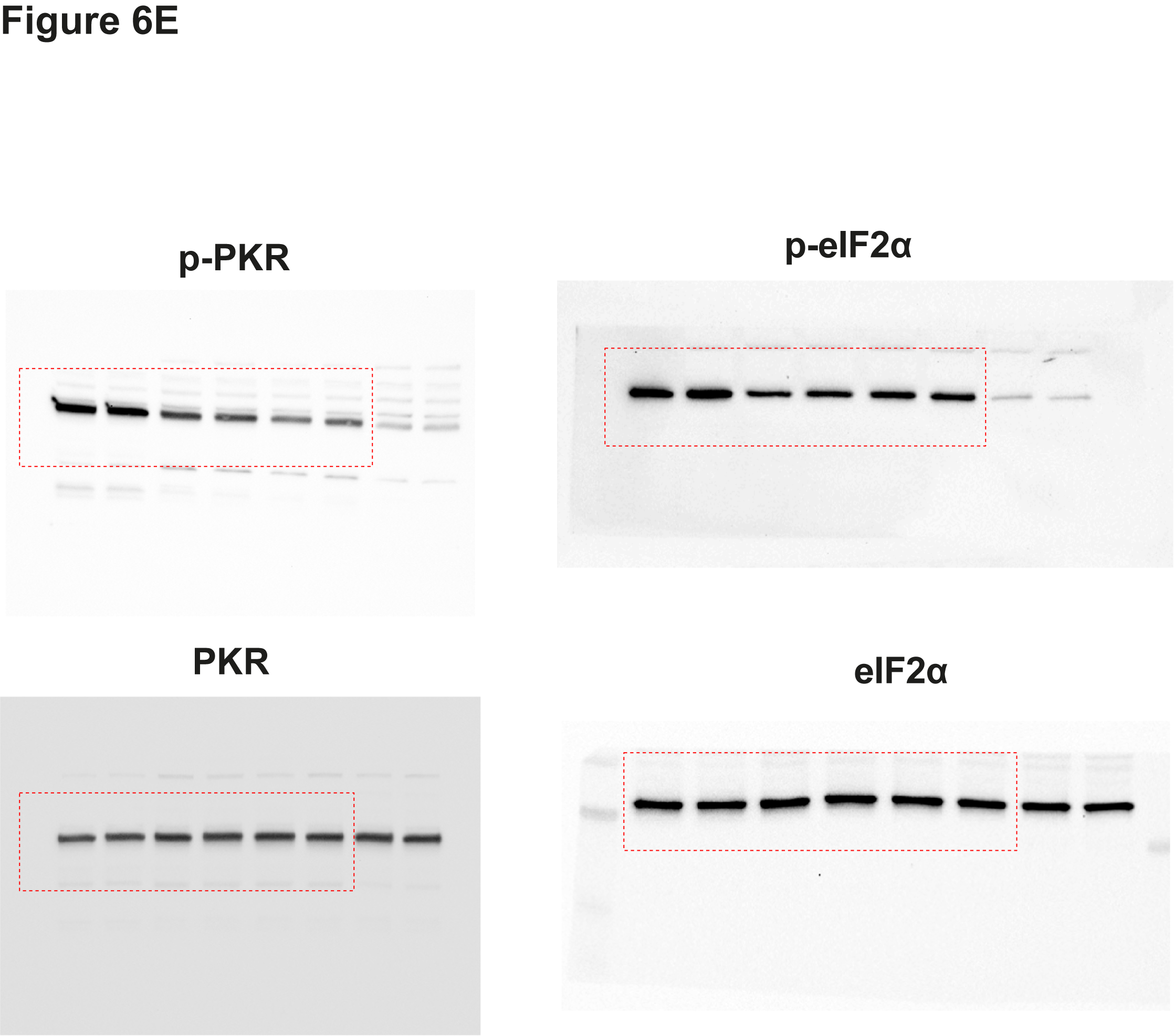

Supplement: Figure 6—source data 1. [file elife-85902-fig6-data1.zip › Figure 6-source data/Labelled/Figure 6-source data 3.tif]

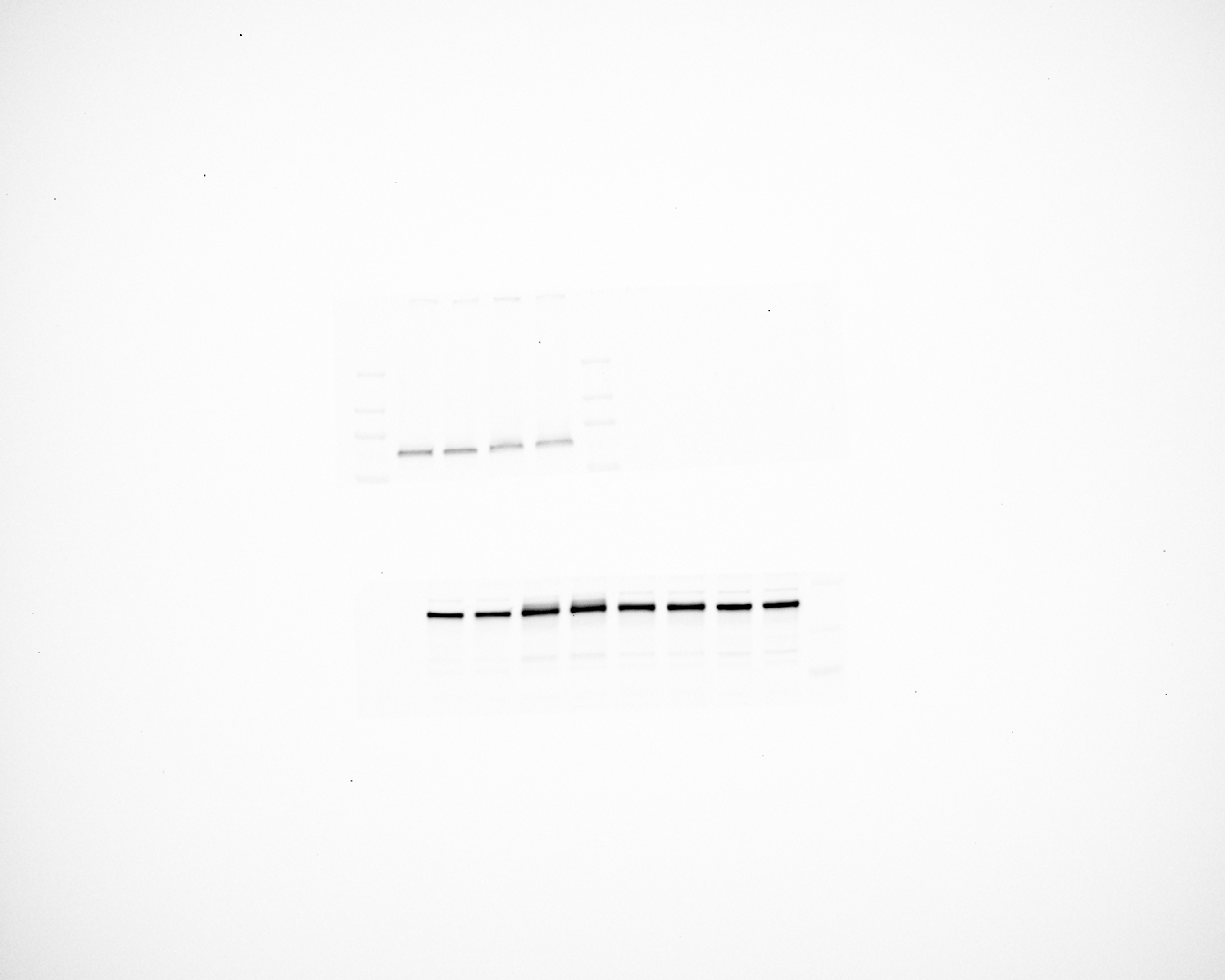

Supplement: Figure 6—source data 2. [file elife-85902-fig6-data2.zip › Figure 6-figure supplement 1-source data_/Unlabelled/F PKR.tif]

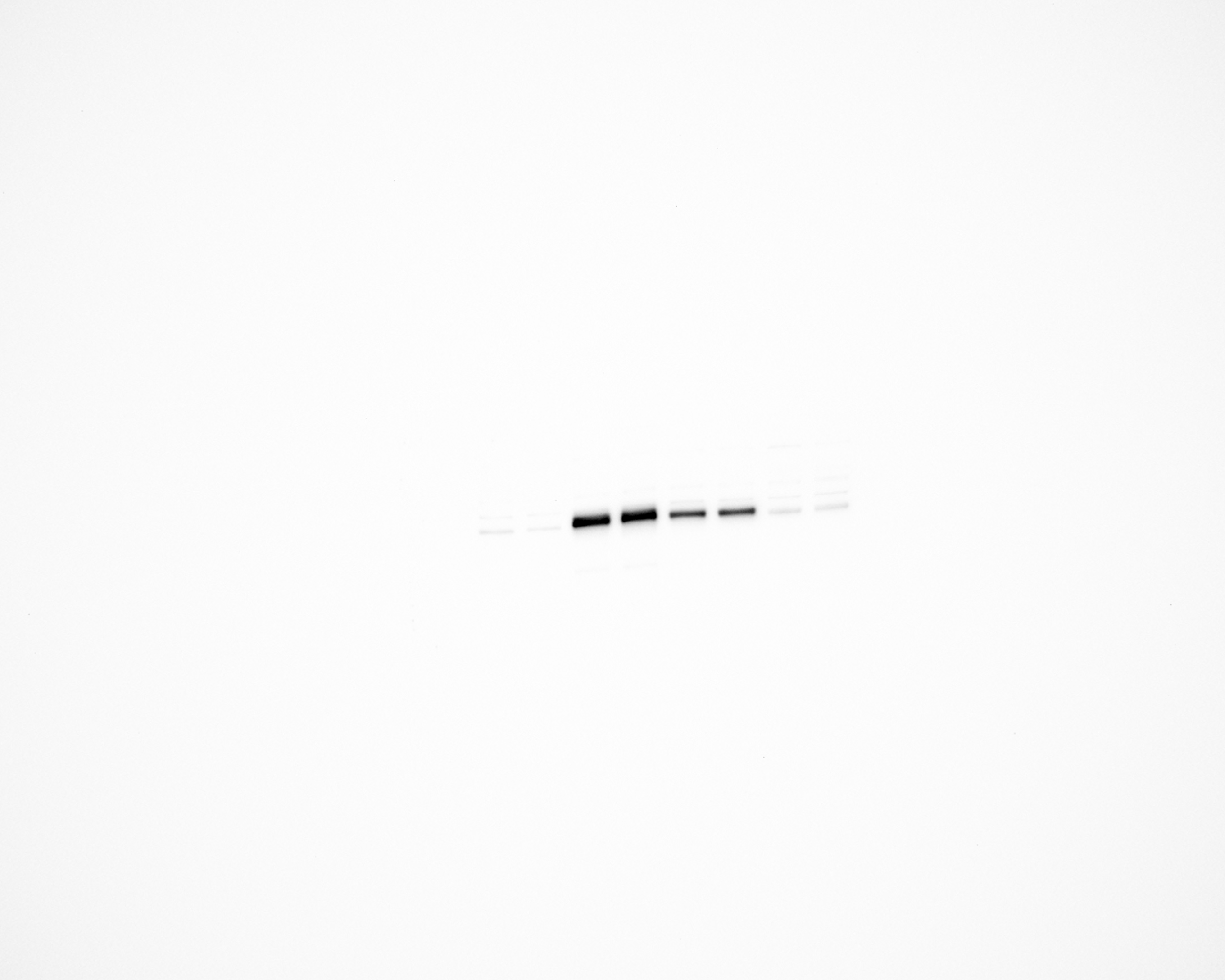

Supplement: Figure 6—source data 2. [file elife-85902-fig6-data2.zip › Figure 6-figure supplement 1-source data_/Unlabelled/F pPKR.tif]

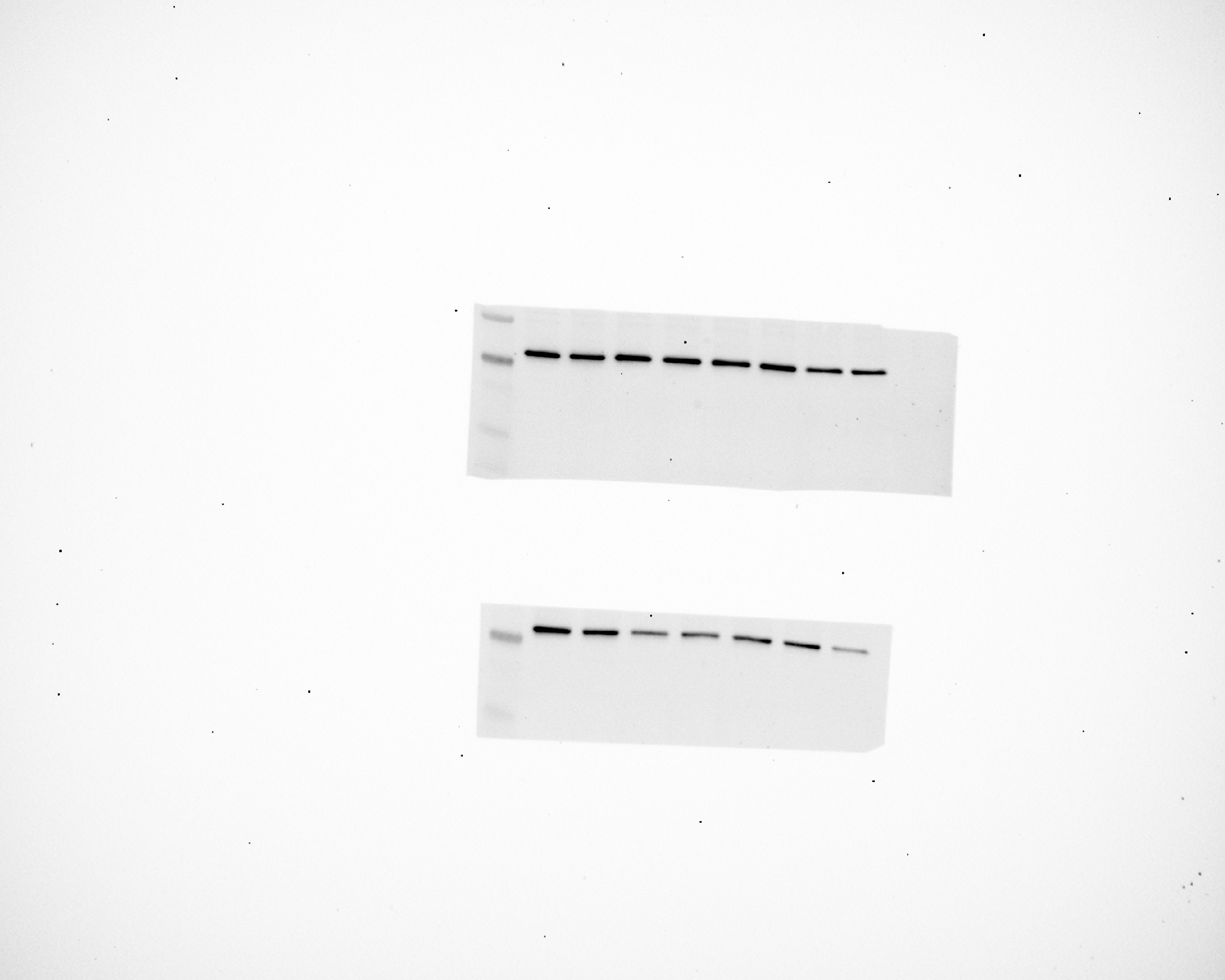

Supplement: Figure 6—source data 2. [file elife-85902-fig6-data2.zip › Figure 6-figure supplement 1-source data_/Unlabelled/F EIF2A.tif]

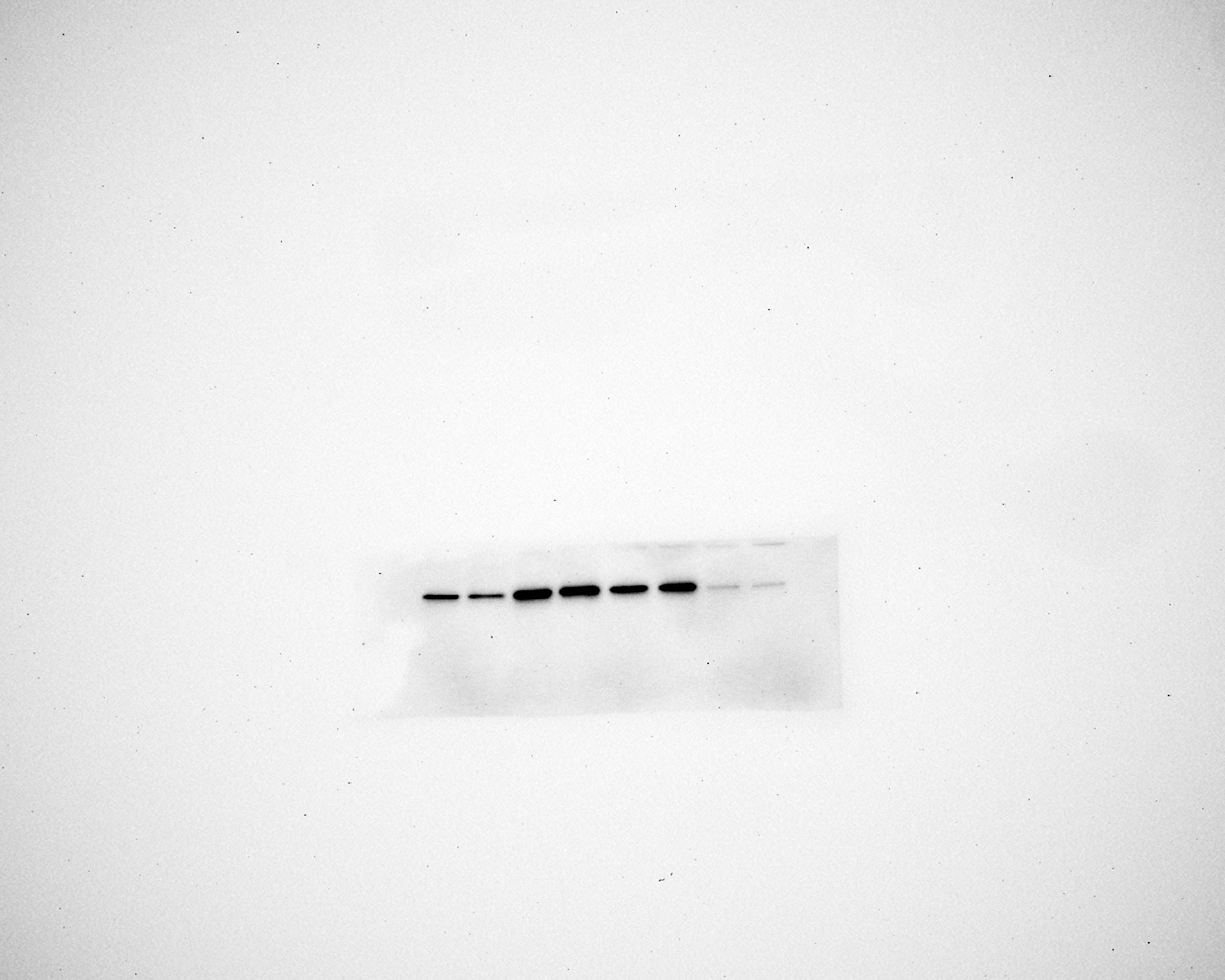

Supplement: Figure 6—source data 2. [file elife-85902-fig6-data2.zip › Figure 6-figure supplement 1-source data_/Unlabelled/F PEIF2A.tif]

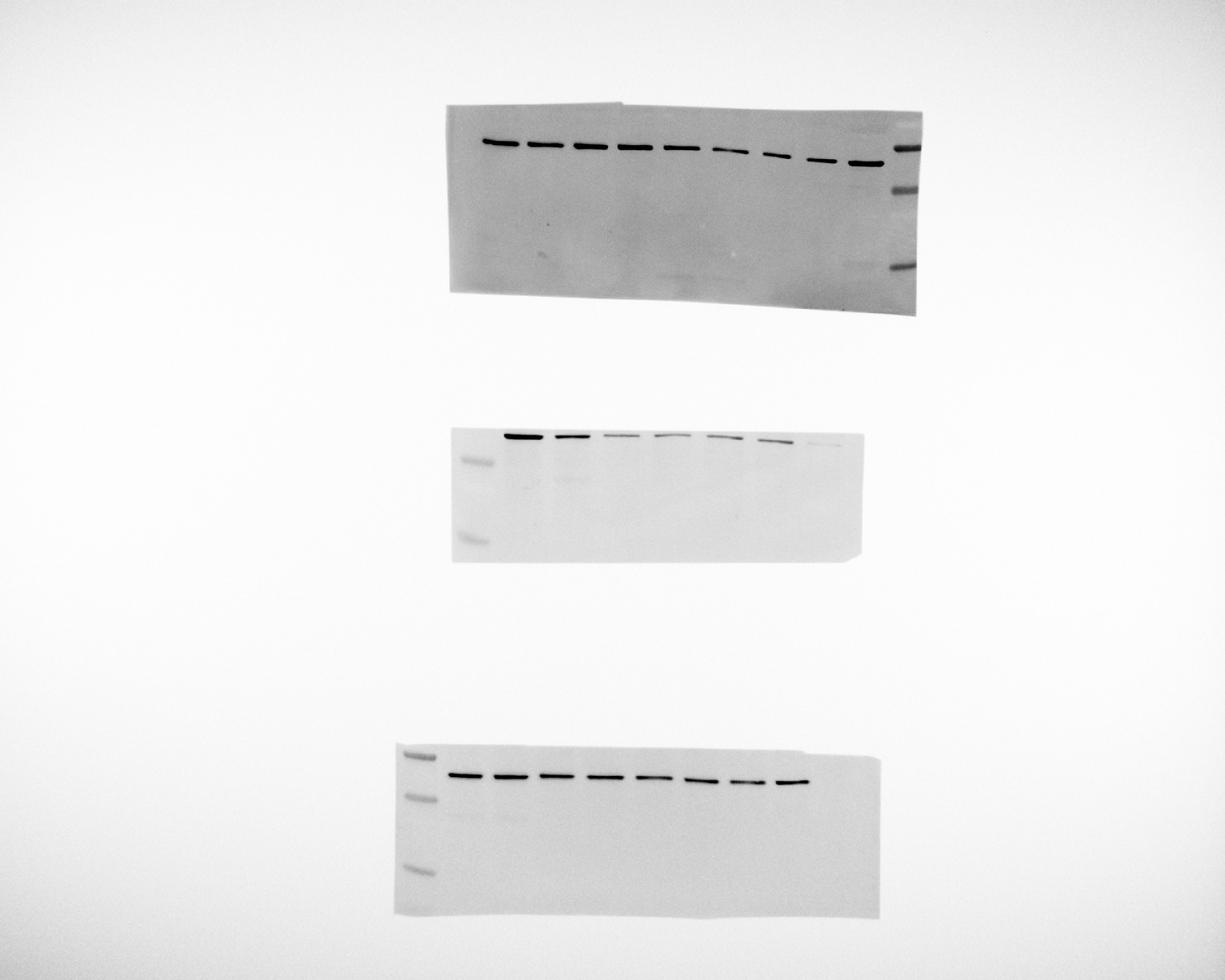

Supplement: Figure 6—source data 2. [file elife-85902-fig6-data2.zip › Figure 6-figure supplement 1-source data_/Unlabelled/F GAPDH.tif]

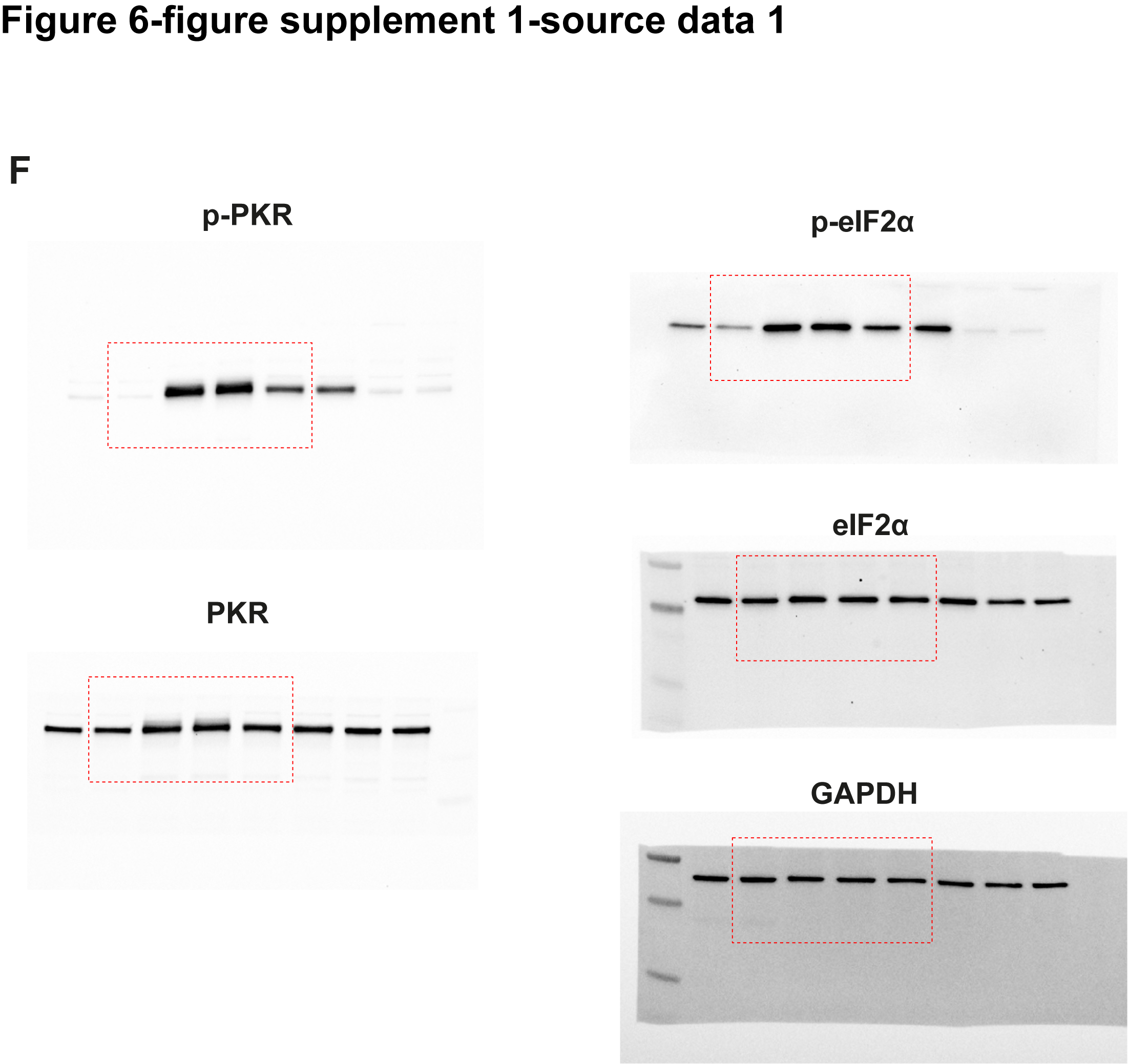

Supplement: Figure 6—source data 2. [file elife-85902-fig6-data2.zip › Figure 6-figure supplement 1-source data_/Labelled/Figure 6-figure supplement 1-source data 1.tif]

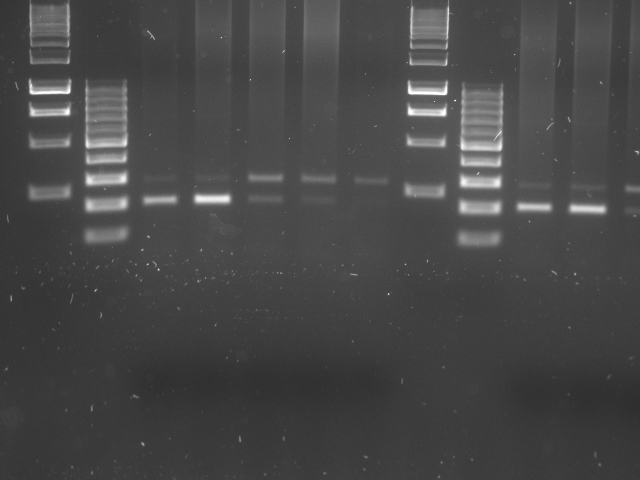

Supplement: Figure 7—figure supplement 1—source data 1. [file elife-85902-fig7-figsupp1-data1.zip › Figure 7-supplement 1-source data/Unlabelled/PKR-left.tif]

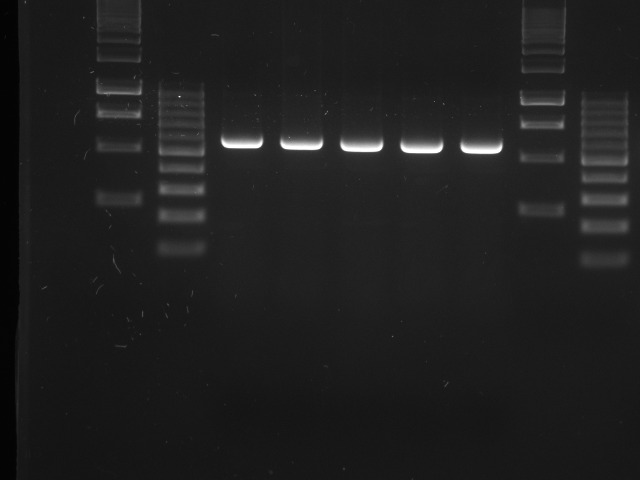

Supplement: Figure 7—figure supplement 1—source data 1. [file elife-85902-fig7-figsupp1-data1.zip › Figure 7-supplement 1-source data/Unlabelled/GAPDH-left.tif]

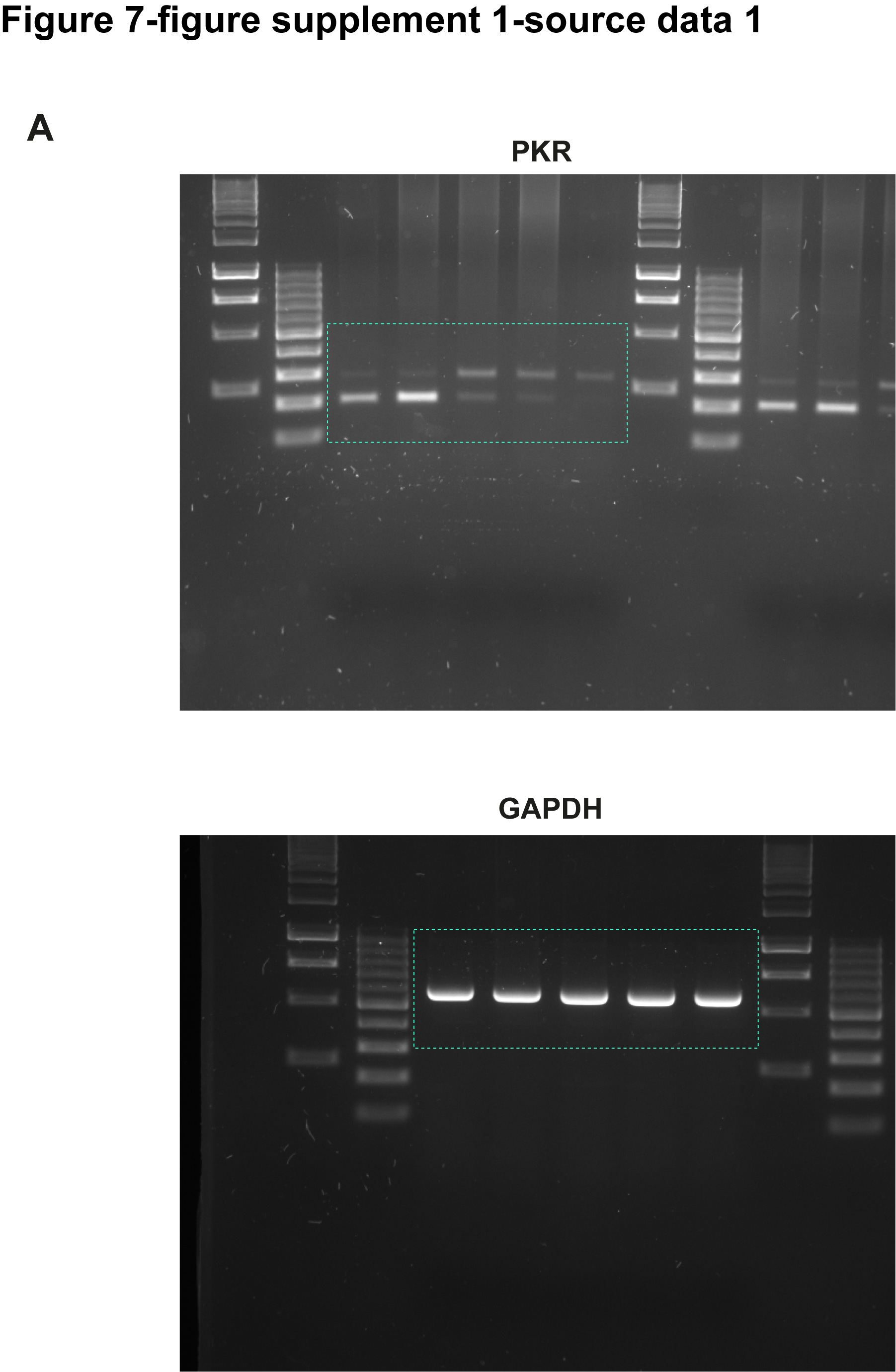

Supplement: Figure 7—figure supplement 1—source data 1. [file elife-85902-fig7-figsupp1-data1.zip › Figure 7-supplement 1-source data/Labelled_/Figure 7-figure supplement 1-source data 1.tif]
